# Adsorption of Polycyclic Aromatic Hydrocarbons and C_60_ onto Forsterite: C–H Bond Activation by the Schottky Vacancy

**DOI:** 10.1021/acsearthspacechem.2c00084

**Published:** 2022-07-27

**Authors:** Dario Campisi, Thanja Lamberts, Nelson Y. Dzade, Rocco Martinazzo, Inge Loes ten Kate, Alexander G. G. M. Tielens

**Affiliations:** † Leiden Observatory, Leiden University, Niels Bohrweg 2, Leiden 2333 CA, The Netherlands; ‡ Leiden Institute of Chemistry, Leiden University, Einsteinweg 55, Leiden 2300 RA, The Netherlands; § Cardiff University, Main Building, Park Place, Cardiff CF10 3AT, U.K.; ∥ Department of Chemistry, Università degli Studi di Milano, Via Golgi 19, Milan 20133, Italy; ⊥ Department of Earth Sciences, Faculty of Geosciences, Utrecht University, Princetonlaan 8a, Utrecht 3584 CB, The Netherlands

**Keywords:** periodic DFT-D4, PAHs, fullerene, forsterite, astrochemistry, cosmochemistry, catalysis

## Abstract

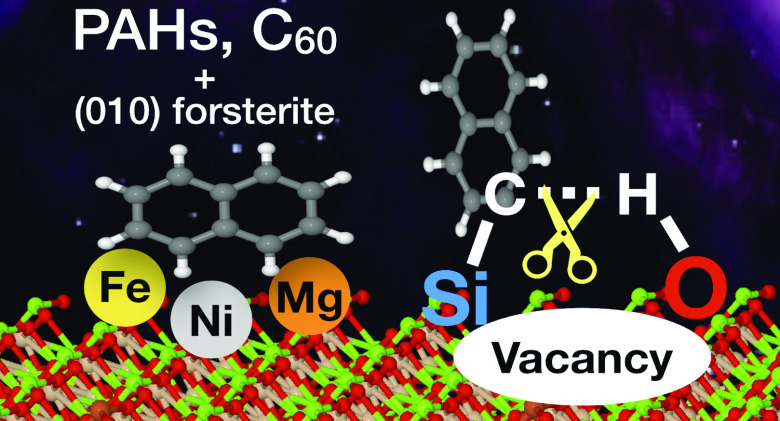

Understanding how to catalytically break the C–H bond of aromatic molecules, such as polycyclic aromatic hydrocarbons (PAHs), is currently a big challenge and a subject of study in catalysis, astrochemistry, and planetary science. In the latter, the study of the breakdown reaction of PAHs on mineral surfaces is important to understand if PAHs are linked to prebiotic molecules in regions of star and planet formation. In this work, we employed a periodic density functional theory along with Grimme’s D4 (DFT-D4) approach for studying the adsorption of a sample of PAHs (naphthalene, anthracene, fluoranthene, pyrene, coronene, and benzocoronene) and fullerene on the [010] forsterite surface and its defective surfaces (Fe-doped and Ni-doped surfaces and a MgO-Schottky vacancy) for their implications in catalysis and astrochemistry. On the basis of structural and binding energy analysis, large PAHs and fullerene present stronger adsorption on the pristine, Fe-doped, and Ni-doped forsterite surfaces than small PAHs. On a MgO-Schottky vacancy, parallel adsorption of the PAH leads to the chemisorption process (C–Si and/or C–O bonds), whereas perpendicular orientation of the PAH leads to the catalytic breaking of the aromatic C–H bond via a barrierless reaction. Spin density and charge analysis show that C–H dissociation is promoted by electron donation from the vacancy to the PAH. As a result of the undercoordinated Si and O atoms, the vacancy acts as a Frustrated Lewis Pair (FLP) catalyst. Therefore, a MgO-Schottky vacancy [010] forsterite surface proved to have potential catalytic activity for the activation of C–H bond in aromatic molecules.

## Introduction

The study of the reactivity of organic molecules as feedstock for the formation of precursors of complex molecular species is the focus of organic chemistry, materials science, catalysis, astrochemistry, and planetary science.^1−4^ The orbital interaction of an sp^2^ carbon with an s orbital of a hydrogen atom forms a strong C–H bond with a bonding energy of about 4.6 eV, which we can find in aromatic species such as benzene^5^ and polycyclic aromatic hydrocarbons (PAHs).^6^ Shedding light on the activation of the aromatic C–H bond is the first step to understand the reactivity of aromatic molecules.^7^ PAHs lock up about 20% of carbon in the universe, whereas 80% is locked up in other species. PAHs might be linked to other organic molecules because they can act as a carbon feedstock; therefore, studying their reactivity is also important for understanding the chemical evolution of our universe.^4^

Finding Earth-abundant materials that are able to activate and catalyze the C–H bond breaking of aromatic species is still a big challenge because of its high bond energy and high stability. Synthetic transition-metal catalysts such as Wilkinson’s (rhodium-based),^8^ palladium,^9,10^ and iridium catalysts^11^ are able to break the aromatic C–H bond. However, these catalysts are harmful to human health and, moreover, very expensive.^12^ Recently, metal-free catalysts such as frustrated Lewis pairs^13^ (FLPs), formed by a Lewis acid and bases that cannot bind together as a result of steric hindrances, have been studied because they can activate aromatic hydrocarbons.^14^

Mineral-bearing materials are organized periodic systems and have been considered as cheap and safe alternatives to synthetic catalysts.^15^ In fact, mineral surfaces have active inorganic interfaces that can host molecular species and possibly activate them.^16^ Hence, minerals catalyze processes that are thermodynamically favorable but kinetically hindered by high energy barriers.^17^

Forsterite (Mg_2_SiO_4_) is one of the most abundant olivinic minerals available on Earth and in space.^18,19^ Experimental studies have reported the potential catalytic role of forsterite and Fe-doped forsterite for the conversion of methanol to olefine and PAHs.^20,21^ However, theoretical and mechanistic studies on the catalytic activity of forsterite are currently lacking. Only a few studies have addressed the interaction of organic species with a forsterite surface using first-principle methods.^22−24^

In this work, we use periodic density functional theory (DFT) along with Grimme’s D4 method^25^ (DFT-D4) to study the adsorption and C–H activation of PAHs (naphthalene, anthracene, fluoranthene, pyrene, coronene, and benzocoronene) on the pristine [010] forsterite surface ([010]-fo).^19,22,26,27^ Along with the pristine [010]-fo surface, we also study the defective surfaces of [010] forsterite^28^ containing Fe dopant (Fe-[010]-fo),^29,30^ Ni dopant (Ni-[010]-fo),^22^ and MgO-Schottky vacancy (V_MgO_-[010]-fo).^31^ Defects are ubiquitous, at different concentrations, in all crystalline mineral structures.^32^ They are often promoted by the radiation of isotopic decay, or by high temperatures during crystal growth, and can be of different natures, that is, bulk, planar, line, and point defects.^32^ A small amount of Fe and Ni in olivine is generally present,^28,33,34^ whereas the presence of a MgO-Schottky vacancy might have been the result of the reaction of olivine with a small amount of water.^35^

We also include the adsorption of C_60_ onto forsterite as this molecule is abundant in space.^36,37^ We analyze the geometrical parameters and adsorption energies for the adsorption of PAHs and C_60_ as well as the energy barriers and the electronic structure associated with the C–H bond dissociation of PAHs only on the vacancy forsterite surface. As it is not fully understood whether astronomically PAHs might be formed in situ on forsterite or adsorbed on forsterite after they are formed in the gas phase,^18^ we report, along with the adsorption energies, the binding energies that might be used for astronomical modeling to predict their abundance and formation. This study aims to understand the surface chemistry of aromatic molecules in interstellar space and on solar system bodies as well as the role of minerals as potential catalysts.

## Theoretical Methods

### Forsterite and PAH Structures

**Figure 1:**
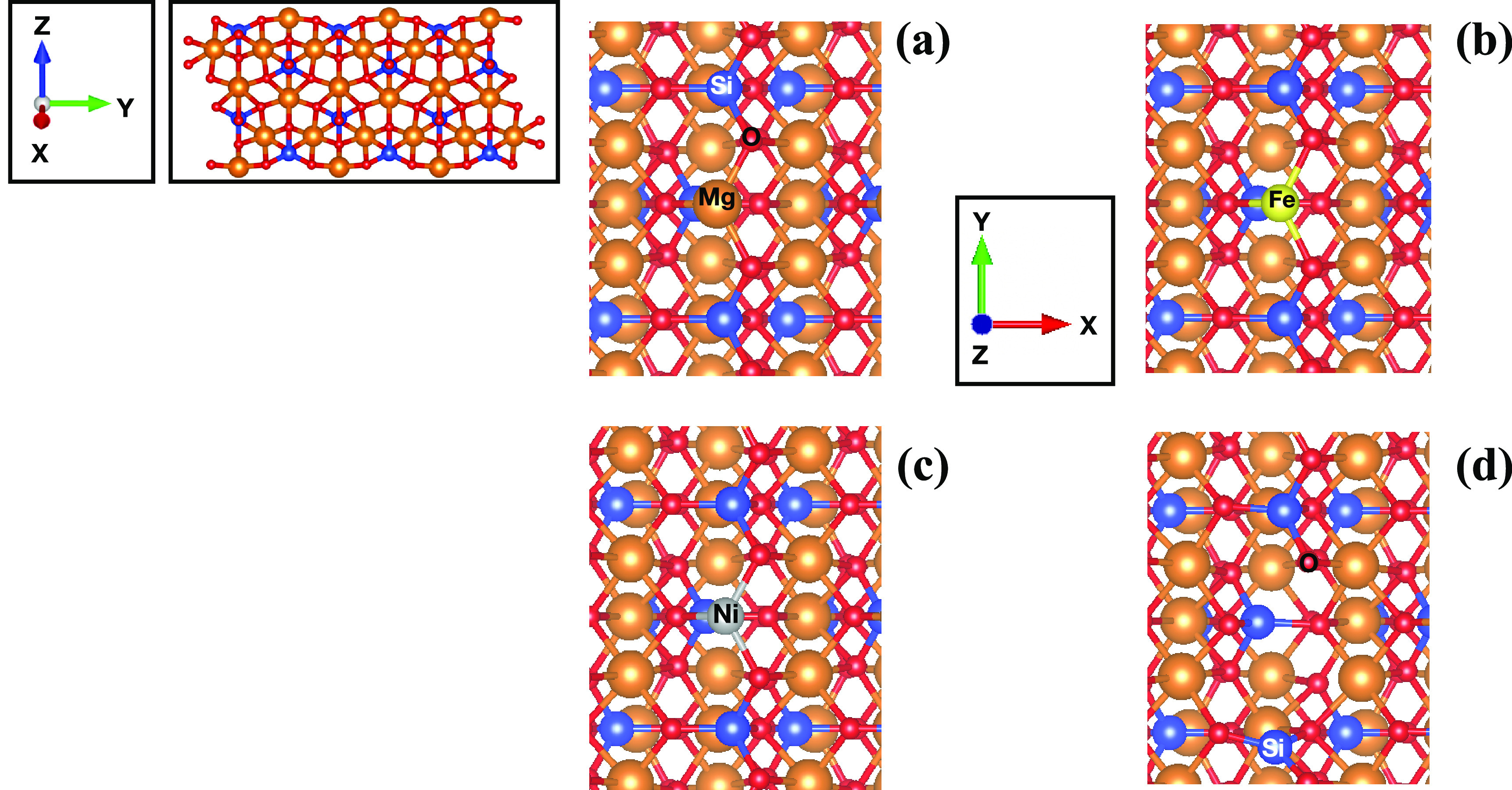
Optimized slab models of [010] forsterite at two different orientations: (a) [010]-fo, (b) Fe-[010]-fo, (c) Ni-[010]-fo, and (d) V_MgO_-[010]-fo (the location of the vacancy is indicated by the Si and O labels reported on the corresponding undercoordinated atoms). The vacuum region is located along the *z*-axis, and the atomic labels of the corresponding atoms (Si, Mg, O, Ni, and Fe) are reported.

In this study, we model the crystalline nonpolar [010]-fo surface (Mg_96_O_192_Si_48_) and its defective surfaces under dry conditions, shown in Figure 1.

All models have a 19 × 17.94 × 35.20 Å (α = β = γ = 90°) supercell (4 × 3 × 1) with a slab thickness of 9.25 Å. The [010] slab surfaces were generated by cutting the optimized bulk of forsterite from a prior study^22^ using the METADISE code.^38^ The bottom layers (about 4 Å thickness) have been constrained during the whole optimization procedure, whereas the topmost layers were allowed to relaxed unconstrainedly. The presence of defects has proved to increase the reactivity and catalytic activity of several materials.^39−41^ Therefore, we decided to model the defective surfaces of [010]-fo by replacing an undercoordinated Mg atom on the surface with only one transition metal (e.g., Fe and Ni for this work) and optimizing the resulting slab model. Furthermore, V_MgO_-[010]-fo is created by removing a MgO unit from the [010]-fo surface, again followed by a reoptimization of the slab. Hence, the pristine surface is characterized by 12 Mg atoms facing the vacuum region, whereas the defective surfaces are characterized by only 11 Mg atoms facing the vacuum region.

The PAHs taken into consideration in this study are naphthalene, anthracene, pyrene, fluoranthene, coronene, benzocoronene, and fullerene. These species (Figure 3 as well as Figures S7–S13 in the Supporting Information) are characterized by honeycomb structures of fused hexagonal carbon rings bound with hydrogen atoms on their edges. Fluoranthene is the only PAH with a five-membered ring. The fullerene, C_60_, is characterized by a spherical shape (buckyball) with hexagonal and pentagonal rings bound together, forming [6,6] and [5,6] ring junctions (Figure S13), which are bridge C atoms of two hexagonal rings and hexagonal-pentagonal rings, respectively. The gas-phase model of PAHs has been optimized in a cubic unit cell with sides of 15 Å.

### Calculation Setup

The slab models were optimized using periodic density functional theory implemented in the SIESTA code,^42^ which makes use of localized atomic orbitals basis sets (LCAO). The details of the method have been described as well as benchmarked in a prior work^22^ and are summarized here.

The Perdew–Becke–Ernzerhof^43^ (PBE) exchange-correlation functional has been employed along with the DZP basis set using the core pseudopotentials and setting the radii of the split valence type for the hydrogen atoms to 0.5. The counterpoise correction^44,45^ is taken into account for all calculations to correct for the basis set superposition error.

The dispersion energy is taken into account a posteriori, on the optimized geometry at the PBE/DZP level, employing Grimme’s DFT-D4 code.^25,46,47^ The Brillouin zone is sampled using a 2 × 2 × 1 Monkhorst–Pack^48^*k*-point grid for all surfaces with a unit cell of 18.996 × 17.938 Å and 1 × 1 × 1 Monkhorst–Pack *k*-point grid for the gas-phase models (reagents). The mesh cutoff is set to 200 Ry (see the Supporting Information of our previous work^22^). All calculations are run with the spin-unrestricted formalism, and convergence is reached when the forces are lower than 0.01 eV Å ^–1^. All structures are optimized using the limited-memory Broyden–Fletcher–Goldfarb–Shanno (L-BFGS) algorithm.^49^ An electric field along the *z*-axis is set to override the resulting dipole moment along the *z*-axis of the slabs during the optimization procedure. In a prior study,^22^ PBE-D4/DZP was proven to be a robust theory level to study the interaction of aromatic molecules on mineral surfaces.

We analyzed the electronic structure of the vacancy surface with the spin-density isosurfaces and the population analysis charge based on the Voronoi scheme.^50^ These techniques allow us to define the spin-up and spin-down population (spin density) of each atom as well as the electronic donation and acceptance between the atoms (partial atomic charges). The spin density and charge analysis have been calculated at the PBE+U level of theory with a single-point calculation using TZP on the optimized DZP geometry with supplementary 4 × 4 × 1 Monkhorst–Pack’s *k*-points. The Hubbard U value, using the LDA+U method,^51^ is set to 15.3 eV to describe the strong on-site Coulomb interaction of the localized electrons of the 2p orbitals of the oxygen atoms to reproduce the experimental band gap (8.4 eV).^52^ A threshold tolerance of 10^–2^ and a tolerance population of 4 × 10^–4^ are selected for the LDA+U method. The radii for the split-valence type of TZP for all atoms, excluding hydrogen, are set up to be 0.30. The reaction barriers (e.g., the characterization of a saddle point) and the minimum energy path are calculated using the climbing-image nudged elastic band method^53^ (CI-NEB) implemented in the atomistic simulation environment^54,55^ python module employing SIESTA as a calculator. The minimum energy path is optimized through linear interpolation of the reagent and the product by six images. Each image can be thought of as a representation along the reaction path with all images interconnected by springs. The optimization focuses on finding the lowest energy possible for each image, and the maximum one only for one image, while maintaining the constraint that they are equally spaced with respect to neighboring images. Note that, after optimization of the CI-NEB path, the energies of the images represent the minimum energy path from the initial to the final geometry. The path is optimized using the Fast Inertial Relaxation Engine (FIRE)^56^ algorithm, which provides robust results compared to those from quasi-Newton methods.^57^ The spring constant is set to 0.1 eV/Å, and the six images between the reagent and the product are considered optimized when the forces are lower than 0.025 eV/Å.

In this work, the binding energy (*E*_bind_) is defined as
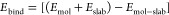
1*E*_mol_ is the energy of the optimized molecule in the gas phase, *E*_slab_ is the energy of the optimized forsterite slab, and *E*_mol–slab_ is the energy of the optimized adsorbed molecule on the forsterite slab. Positive and negative binding energy values show exoergic and endoergic adsorption processes, respectively.

The adsorption energy (*E*_ads_) is defined as
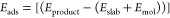
2*E*_product_ is the energy of the optimized product. For this definition, *E*_ads_*= −E*_bind_. Therefore, negative and positive values of *E*_ads_ show exoergic and endoergic processes, respectively.

Harmonic frequencies of PAHs adsorbed onto a vacancy surface are computed to compare different adsorption configurations that differ from the position of an H atom. The zero-point energy correction (*E*_ΔZP_) is calculated as
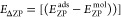
3*E*_ZP_^ads^ is the zero-point energy correction of PAH adsorbed onto a vacancy forsterite surface where only the PAH was allowed to vibrate and *E*_ZP_^mol^ is the zero-point energy of the PAH in the gas phase. The resulting corrected zero-point adsorption energy is the sum of *E*_ads_ and *E*_ΔZP_.

We define contact points (C.P.) as the sum of orbital interactions between the p orbital of a C atom with the 3p orbital of an Mg atom and 3d orbital of Fe and Ni atoms. The surface area of each PAHs is calculated as the sum of the area of the single hexagons and/or pentagons using the carbon backbone, that is, excluding the bound H atoms.

## Results and Discussion

### Structure of the [010] Forsterite Surfaces

The [010]-fo surface has an orthorhombic structure with the *Pbnm* space group, and differs from the bulk because of the presence of undercoordinated Mg atoms bound with three neighboring O atoms on the external layer that faces the vacuum region.

**Figure 2:**
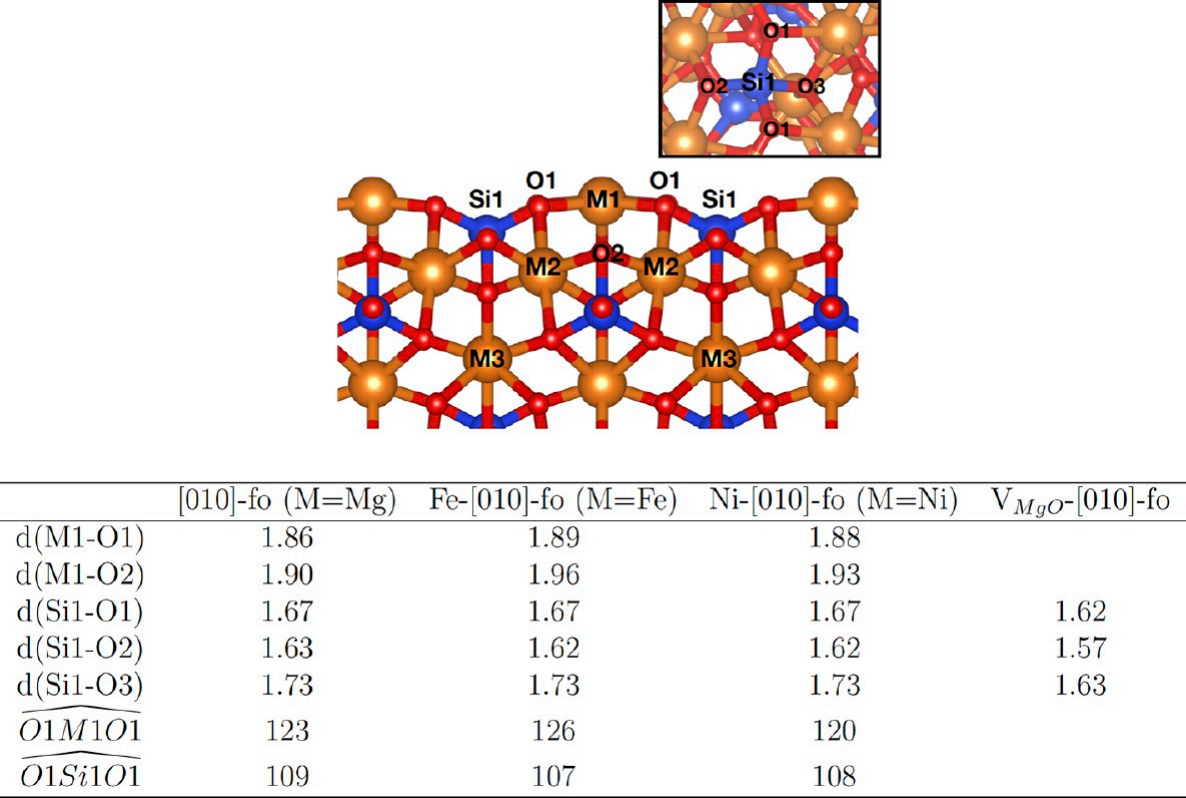
Side-view figure that shows the labels of the atomic positions of the [010]-fo forsterite. M shows the location of an Mg, Fe, and Ni atoms or MgO vacancy. The table reports distances (*d*), in Angstrom (Å), and angles, in degrees (°), of the atoms found in the top figure.

The surface structure of [010]-fo is characterized by undercoordinated Mg atoms (M1) shown in Figure 2 and fully coordinated Mg atoms (M2) located in the underlying layer. The Si (Si1) atoms on the surface are fully coordinated (tetrahedral structures). The bond distances, only for the atoms located on the surface, are reported in the table included in Figure 2 and show Mg–O and Si–O distances ranging between 1.63 and 1.90 Å. Mg, O, and Si atoms, on the surface, form zigzag edges following an alternating unit scheme: −O–Si–O–Mg–O–. The Mg–O bond lengths of the undercoordinated Mg atoms on the surface (M1) are shorter than those of the octahedral Mg–O (M2–O2) with a bond distance that ranges between 2 and 2.16 Å. All calculated bond lengths are in agreement with the DFT study carried out by Navarro-Ruiz et al.^58^ using LCAO methods and by Brodholt using plane wave methods.^59^

We also studied the presence of point defects on the [010]-fo surface, shown in Figure 1b–d. The Fe-[010]-fo surface is characterized by an undercoordinated Fe^2+^ atom bound to three oxygen atoms. When the Fe is located in the M1 site, it is more stable by 0.30 eV with respect to the M2 site and 0.22 eV with respect to the M3 site. Hence, M1 is the preferred site (Figure 2). The table reported in Figure 2 shows the bond distances and angles of the Fe-[010]-fo, which are in close agreement with the geometrical parameters of the pristine surface.

Along with Fe-[010]-fo, we considered the presence of undercoordinated Ni as a point defect on the surface, Ni-[010]-fo (Figure 1 c). The location of the Ni point defect in the M1 site is slightly more stable by about 0.04 and 0.01 eV compared to those of M2 and M3, respectively. However, this difference is not significant considering the accuracy of the functional used.^22^ Once the transition metal coordinates with an aromatic molecule such as naphthalene, the Ni point defect in the M1 site is stabilized by 0.30 and 1.61 eV with respect to M2 and M3. The table in Figure 2 shows bond distances and angles for Ni-[010]-fo, which are in close agreement with those for Fe-[010]-fo and [010]-fo. Overall, the presence of transition metals does not significantly change the forsterite structure as compared to the pristine [010]-fo structure. The stability of the transition metals on the surface, as reported by the study of Navarro-Ruiz et al.,^29^ is due to the need to stabilize the high-spin state (quintet for Fe and triplet for Ni), reducing the Fe or Ni–O electronic repulsion in the bulk caused by the size of these atoms.

Another type of defect occurring in crystalline structures is the MgO-Schottky defect, which can be formed when a cation–anion pair is removed, resulting in a vacancy.^60^ The energy required to form the Schottky-MgO vacancy defect is about 7 eV for the bulk^35^ and 11.85 eV on the surface, as computed by us. For comparison, SiO_2_ vacancies are less favorable because the formation energy is about 20 eV for the bulk^35^ and 14.23 eV for SiO removed from the surface, as computed by us. As the formation energies of these vacancies are large, we expect that the abundance must be low, which justifies the choice of modeling only one MgO vacancy on the surface (further discussion is reported in “Astrochemical and Cosmochemical Implications”). The presence of the Schottky MgO vacancy (V_MgO_-[010]-fo), shown in Figure 1d, causes an interruption of the zigzag units, resulting in the formation of a SiO_3_^2–^ unit and O atoms that bridge an Mg and Si atom. The absence of an MgO unit does not allow the formation of a bond between the two distant Si and O atoms on the surface. The distance between the unbound Si and O atoms, the two labeled atoms in Figure 1d, in the vacancy is about 5.12 Å, causing the formation of a cavity. The table in Figure 2 shows that the Si–O distances are slightly shorter than those of other surfaces. Instead, the O atom that lacks a neighboring Mg atom because of the vacancy (Figure 1d) binds with a second neighbor, Si (Si1), which has a distance of 1.61 and 1.97 Å with the Mg located in the M2 site. Therefore, the cavity formation (see the structure in Figure 10a) causes a bond length reorganization to compensate for the absence of an Mg atom. The MgO vacancy is more stable when located on the surface in the M1 site by about 2.90 eV with respect to the M2. Hence, M1 is the most favorable site for all three defects considered in this study.

### Interaction of PAHs and C_60_ with [010] Forsterite

#### Structural Analysis

Previous theoretical studies have shown that forsterite strongly binds with aromatic molecules on its surfaces.^22−24^

**Figure 3:**
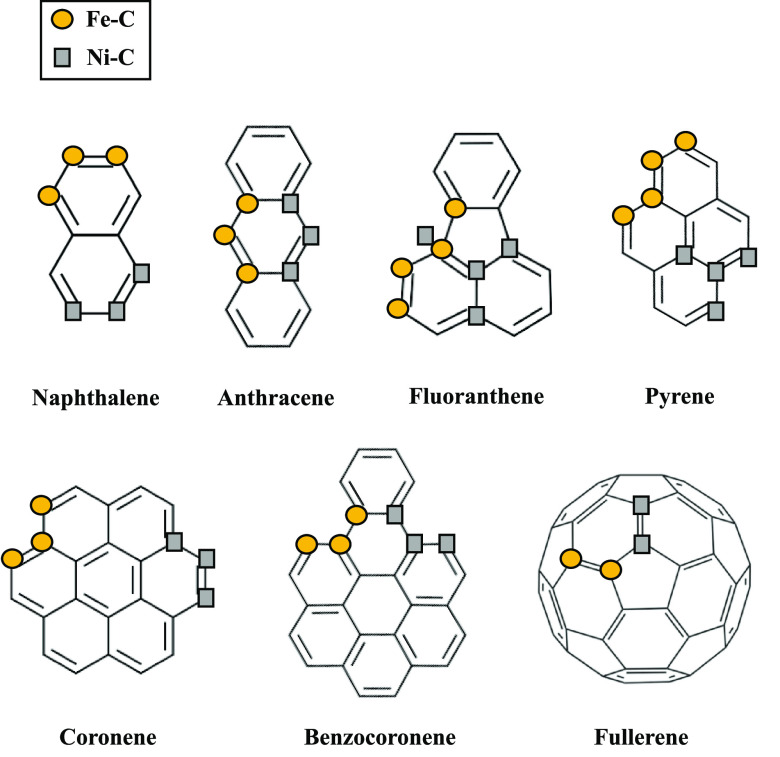
The skeleton structure of PAHs and the location of carbon–transition-metal interactions less than 3 Å (see the Supporting Information), on the PAH structure, when they adsorb on the Fe and Ni-[010]-fo. The legend shows the symbol related to C–Fe (circles) and C–Ni (squares) interactions.

Therefore, we have selected a sample of PAHs that span a range in the surface area (Table 1) and that contain catacondensed PAHs (e.g., acenes, naphthalene, and anthracene), pericondensed PAHs (pyrene and coronene), and irregular PAHs (benzocoronene). In addition, we also selected the nonalternant aromatic species fluoranthene and fullerene. The skeleton structure of the mentioned PAHs is reported in Figure 3.

**Table 1:** Surface Area of PAHs and the [010] Forsterite Surfaces ([010]-fo, Fe-[010]-fo, Ni-[010]-fo, and V_MgO_-[010]-fo) Optimized in This Study^a^

	surface area (Å^2^)
naphthalene	10.18
anthracene	15.28
fluoranthene	18.34
pyrene	20.37
coronene	35.64
benzocoronene	40.73
forsterite surfaces (4 × 3 × 1)	340.74

a The average sizes of the hexagons and pentagons are 1.40 Å, which is the distance of a single C–C bond.

**Figure 4:**
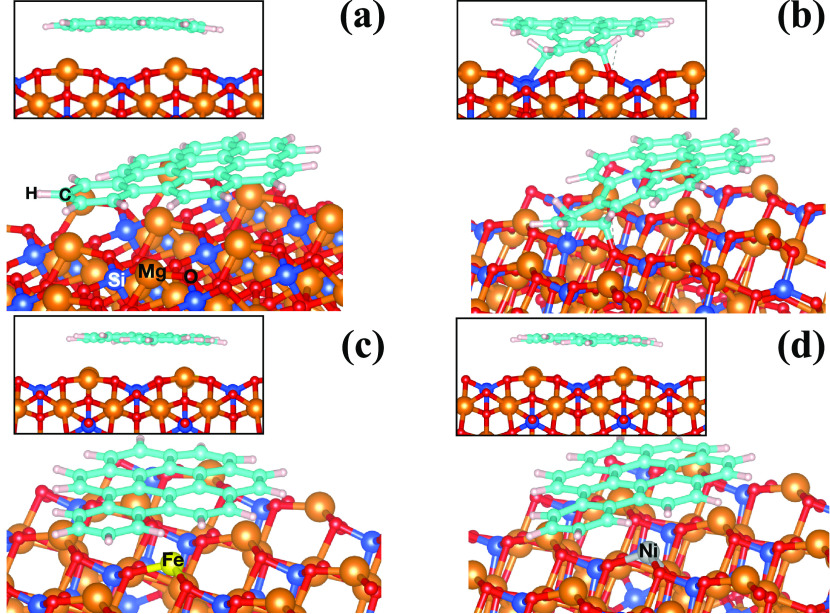
Side views using two different perspectives (second perspective in thumbnail) of the optimized geometries of benzocoronene adsorbed on (a) [010]-fo, (b) V_MgO_-[010]-fo, (c) Fe-[010]-fo, and (d) Ni-[010]-fo. Atomic labels are reported on the corresponding atoms.

We optimized all structures for a parallel adsorption of the molecular plane with respect to the surface. The adsorption of PAHs on the [010] forsterite surfaces causes a slight deviation of the PAH structure from their planarity (Figure 4).

**Figure 5:**
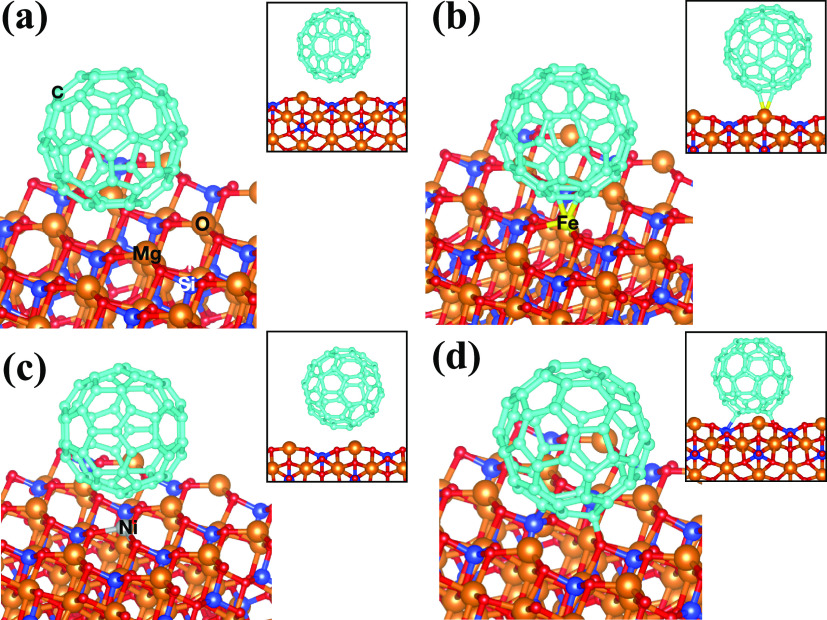
Side views using two different perspectives (second perspective in thumbnail) of the optimized geometries of fullerene adsorbed on (a) [010]-fo, (b) Fe-[010]-fo, (c) Ni-[010]-fo, and (d) V_MgO_-[010]-fo. Atomic labels are reported on the corresponding atoms.

This is in-line with the average dihedral angles of the C atoms of the PAH skeleton, reported in Table 2. The average dihedral angles do not show characteristic planar structures of 180°. With the exception of pyrene, PAH adsorption on [010]-fo and Fe-[010] causes a larger deviation from planarity than that on Ni-[010]-fo in which the adsorbed molecules have dihedral angles close to 180°. The average angles indicate a perfect 120°, characteristic of sp^2^ carbons for all PAHs with the exception of fluoranthene. The latter show larger angle deviation from 120° with respect to the other PAHs because of the presence of a five-membered ring that causes major bending of its structure. The major flexibility due to the presence of a five-membered ring has been identified also in other materials with PAH-like structures.^39^ The  and dihedral Θ angles reported in Table 2 indicate that PAHs adsorbed on the surface have the 120° angles expected for planar PAHs, whereas the fullerene (C_60_) deviates from this by about 6°, as expected for a buckyball-like structure (Figure 5a,b,c).^61^ Thus, we conclude that the adsorption process does not induce significant changes in the conformational structure of the PAHs relative to the gas phase, apart from a moderate loss in planarity.

**Table 2:** Geometrical Parameters of PAHs Adsorbed on Forsterite Surfaces^a^

	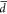 (C–Mg)	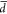 (C–M)	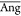	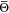	[nC–nMg]	[nC–nM]	C.P.
naphthalene:							
[010]-fo^22^	2.61		120.21	176.18	5–2		5
Fe-[010]-fo^22^	2.51	2.43	120.02	172.04	2–1	3–1	5
Ni-[010]-fo^22^	2.64	2.42	120.04	178.14	2–1	3–1	5
anthracene:							
[010]-fo	2.65		120.82	173.06	6–2		6
Fe-[010]-fo	2.70	2.40	120.47	176.65	3–2	3–1	6
Ni-[010]-fo	2.73	2.41	120.38	177.05	3–2	3–1	6
fluoranthene:							
[010]-fo	2.68		125.34	174.68	7–3		7
Fe-[010]-fo	2.62	2.56	125.73	176.99	3–2	4–1	7
Ni-[010]-fo	2.52	2.62	126.02	177.83	2–1	4–1	6
pyrene:							
[010]-fo	2.72		120.79	179.15	5–2		5
Fe-[010]-fo	2.64	2.61	121.19	175.30	3–1	4–1	7
Ni-[010]-fo	2.66	2.50	120.86	177.64	3–1	4–1	7
coronene:							
[010]-fo	2.73		120.92	175.63	11–4		11
Fe-[010]-fo	2.63	2.51	120.97	174.78	7–3	3–1	10
Ni-[010]-fo	2.68	2.46	120.80	177.90	5–3	3–1	8
benzocoronene:							
[010]-fo^22^	2.71		120.61	176.29	11–4		11
Fe-[010]-fo^22^	2.66	2.47	120.46	178.90	8–4	3–1	11
Ni-[010]-fo^22^	2.67	2.48	120.43	179.18	8–4	3–1	11
fullerene:							
[010]-fo	2.74		113.49	139.00	4–2		4
Fe-[010]-fo		2.10	106.82	133.63		2–1	2
Ni-[010]-fo	2.81	2.09	111.65	143.19	1–1	2–1	3

a Reported are the average distances  in Angstrom (Å), average angles  and dihedral angles  in degrees (deg), number (*n*) of atoms (C, Mg, or M) interacting with each other (e.g., [ nC–nMg ]), and the number of contact points (C.P.), which is the sum of C atoms interacting with either an Mg or transition metal. The average values are calculated from the geometrical parameters reported in the Supporting Information. M is the transition metal (e.g., Fe or Ni) of the corresponding surface (e.g., Fe for Fe-[010]-fo). Missing values shows no interaction between the atoms.

The larger the surface area of the PAHs (Table 1), the larger the number of interactions of the C atoms either with an Mg or with transition-metal dopant atoms (contact points), as shown in Table 2. Although pyrene has a larger surface area than fluoranthene, the number of contact points in pyrene is roughly the same as that in fluoranthene. This could be attributed to the more compact structure of the pyrene skeleton as compared to that of fluoranthene. With the exception of pyrene and fullerene, the number of Mg atoms interacting with the C atoms at a distance less than 3 Å increases with increasing surface area of the PAH. Therefore, the larger the surface area, the higher the number of contact points. Distances larger than 3 Å do not present orbital interactions (see Population Analysis section in the Supporting Information).

#### Interaction of PAHs and C_60_ with Doped Forsterite

Figure 3 shows the preferred carbon sites of PAHs and fullerene that interact with Fe or Ni on the surface. Naphthalene, anthracene, coronene, and benzocoronene have three transition-metal interactions located in the edge carbons of the PAHs. Fluoranthene and pyrene, instead, have four C–Fe and C–Ni interactions. Furthermore, for fluoranthene and pyrene, we noticed a different behavior from the Ni–C interactions because the optimization of the geometry led to a structure in which the inner carbon of these PAHs can interact with Ni atoms at distances smaller than 3 Å (see Geometrical Parameters section in the Supporting Information for details). Instead, fullerene has two Fe–C and Ni–C interactions located in the [6,6] ring junction.

PAHs do not adsorb with the center of the π-ring on the transition-metal-doped surfaces (Fe and Ni-[010]-fo), as seen for benzene–cation complexes (gas-phase configuration) with the same spin configuration of the surfaces (quintet for Fe^2+^ and triplet for Ni^2+^). The gas-phase configuration shows carbon–transition-metal (C–M) bond distances of about 2.28 Å for Fe^2+^ and 2.17 Å for Ni^2+^ (values calculated from the optimized geometries reported in a prior study^22^ and in agreement with published theoretical studies of Fe–PAH complexes in the gas phase^62^). The PAHs on forsterite surfaces have, as shown in Table 2, slightly larger C–M bond distances with respect to the gas-phase configuration. On a regular forsterite surface, the PAH will orient itself on the surface to interact closely with as many Mg atoms as possible. For doped surfaces, binding of PAHs is a competition of favorable orientations to interact with as many Mg atoms as possible and to optimize the interaction with the transition metal. Table 2 summarizes this competition, illustrating that, on doped surfaces, the number of C–Mg interactions decreases relative to the pristine surface but this is compensated for by the interactions with the transition metal. In general, but not always, the number of contact points remains the same. We note that the difference between the Fe–C and Ni–C bond distances of the PAHs on forsterite surfaces with respect to the gas-phase configuration, considering the optimized distance, is about 0.12–0.33 Å for Fe and 0.24–0.45 Å for Ni. Hence, PAHs can optimize the Fe–C distance on an Fe-doped surface better than that on a Ni-doped surface.

Fullerene shows smaller (Table 2) C–Fe and C–Ni distances than PAHs, resulting in a stronger interaction.

#### Interaction of PAHs and C_60_ with a Vacancy

**Figure 6:**
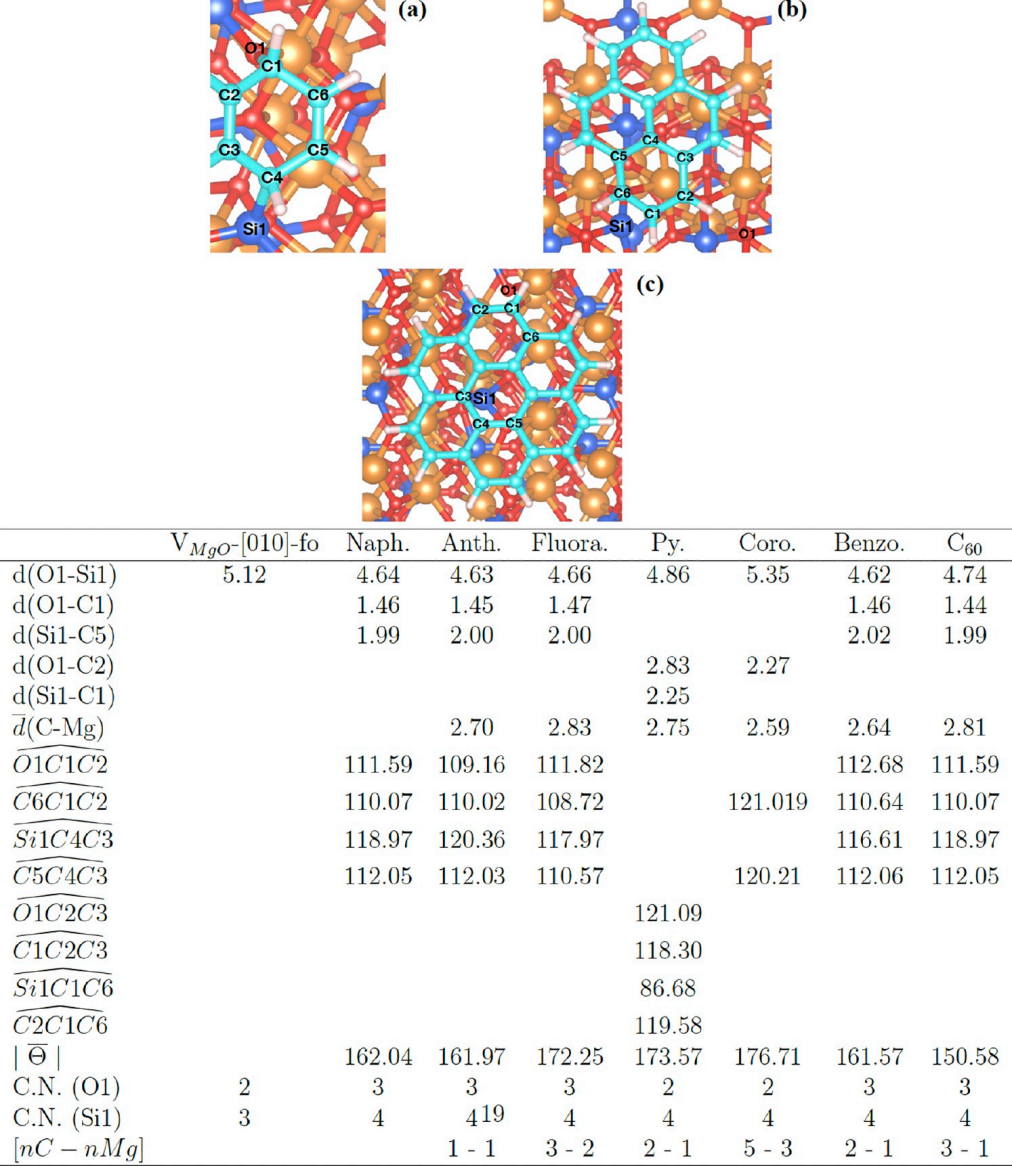
For the PAHs adsorbed on the V_MgO_-[010]-fo surface, we report a schematic representation of the atomic labels of the hexagonal rings chemisorbed with the undercoordinated Si and O for (a) naphthalene, anthracene, fluoranthene, benzocoronene, and fullerene, (b) pyrene, and (c) coronene. A table reporting the average distance  and distance (*d*) in Angstrom (Å), angles  and average dihedral angles , in degrees (deg), coordination number (C.N.) for the specified atom, and the number (*n*) of atoms (C and Mg) interacting with each other ([*n*C–*n*Mg ]) for the vacancy surface, PAHs, and C_60_. Missing values indicate no interaction between the atoms. The average values are calculated from the geometrical parameters reported in the Supporting Information.

The geometrical structures of all species adsorbed on V_MgO_-[010]-fo are reported separately in a table in Figure 6. The vacancy has peculiar structures with respect to the pristine and transition-metal-doped ones. Specifically, the adsorption of naphthalene, anthracene, fluoranthene, benzocoronene, and fullerene have characteristic  angles for the rehybridization of the C atoms of PAHs from sp^2^ to sp^3^ (about 109°). The C–Si bond has a short distance, about 1.9 Å, and the  angles, about 118–120°, are close to the geometrical parameters of silane complexes (about 120°).^63^ Therefore, the adsorption of these species on the vacancy surface causes the breaking of one aromatic ring (i.e., a chemisorption process). The  angles, in the table in Figure 6, show a perfect 109° for anthracene, whereas the other PAHs slightly diverge from this value. Hence, the surface area of anthracene allows a perfect interaction with the vacancy. Pyrene and coronene do not show covalent bond formation. In fact, species only with a terminal aromatic ring with four hydrogen atoms (naphthalene, anthracene, fluoranthene, and benzocoronene) and the six-membered ring of fullerene form covalent bonds with the undercoordinated Si and O atoms (see C.N. in the table in Figure 6). This occurs as a result of their large structural flexibility with respect to more compact structures such as pyrene and coronene. The coordination number of O and Si increases once PAHs adsorb on the vacancy forsterite surface, in-line with the formation of C–O and C–Si bonds. For the case of pyrene, the molecule binds only with the Si atom, leaving the O atom with a lower coordination number (2) with respect to the adsorption of the other PAHs and C_60_. However, the Si–C distance, for the case of pyrene, is larger by about 0.25 Å as compared to that for the other PAHs chemisorbed on the vacancy. The adsorption structure of pyrene on the vacancy is shown in Figure 6b. All species have O–C and Si–C bond distances in good agreement with the average experimental values (1.40 and 1.87 Å, respectively).^64^

**Figure 7:**
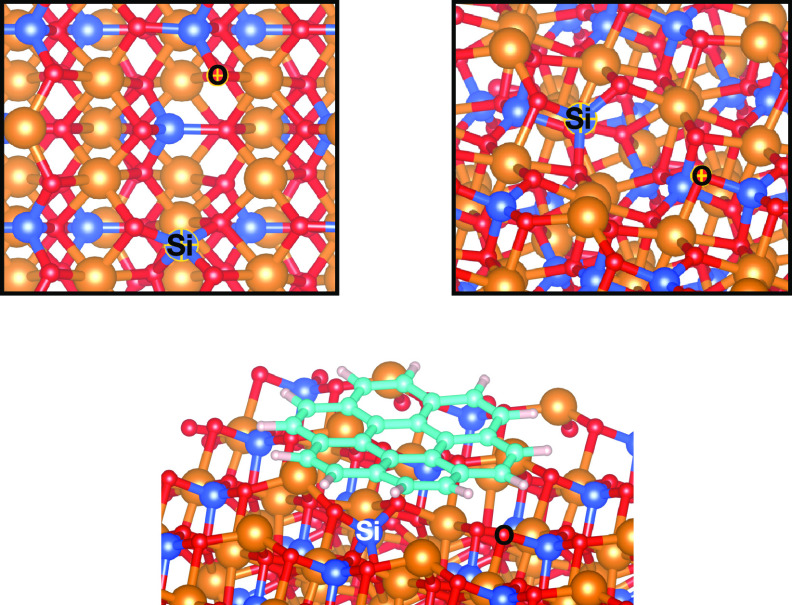
Vacancy reconstruction caused by the adsorption of coronene on V_MgO_-[010]-fo. In the thumbnail is reported the close-up perspective of the reconstruction. The atomic labels on the corresponding atoms indicate the reconstructed atoms.

The distance of the undercoordinated Si and O atoms on the vacancy structure is reduced once their coordination is completed by the chemisorption of the PAHs (see the O1–Si1 distance in the table reported in Figure 6). However, for the case of coronene, the distance O1–Si1 increases with respect to the noncoordinated surface. Angles and dihedral values of coronene adsorbed on the vacancy do not show the formation of C sp^3^, but a quasi-planar structure, in agreement with the values shown in the adsorption on the pristine and transition-metal-doped surfaces. The coordination number of O and Si of the vacancy, when coronene is adsorbed on it, is completed. Nevertheless, for the case of coronene adsorbed on the vacancy, there is no covalent bond formation; the vacancy enlarged its structure to accommodate the large surface area of coronene, which in turn causes the formation of a Si reconstruction, as shown in Figure 7.

The vacancy reconstruction, caused by the adsorption of coronene on the vacancy, is characterized by the formation of the Si–O bond located in the underlying surface, moving the Si atom between the surface layer and the layer below the surface, completing the coordination of the Si atom. Also, the O atom on the vacancy completes the coordination with a Si located in the underlying surface.

Because of the interaction of the PAHs with the vacancy, the number of Mg atoms interacting with C atoms is much lower with respect to those of the pristine and metal-doped surfaces, comparing the values between the Table 2 and the table reported in Figure 6, respectively.

#### Binding Energy Analysis

**Figure 8:**
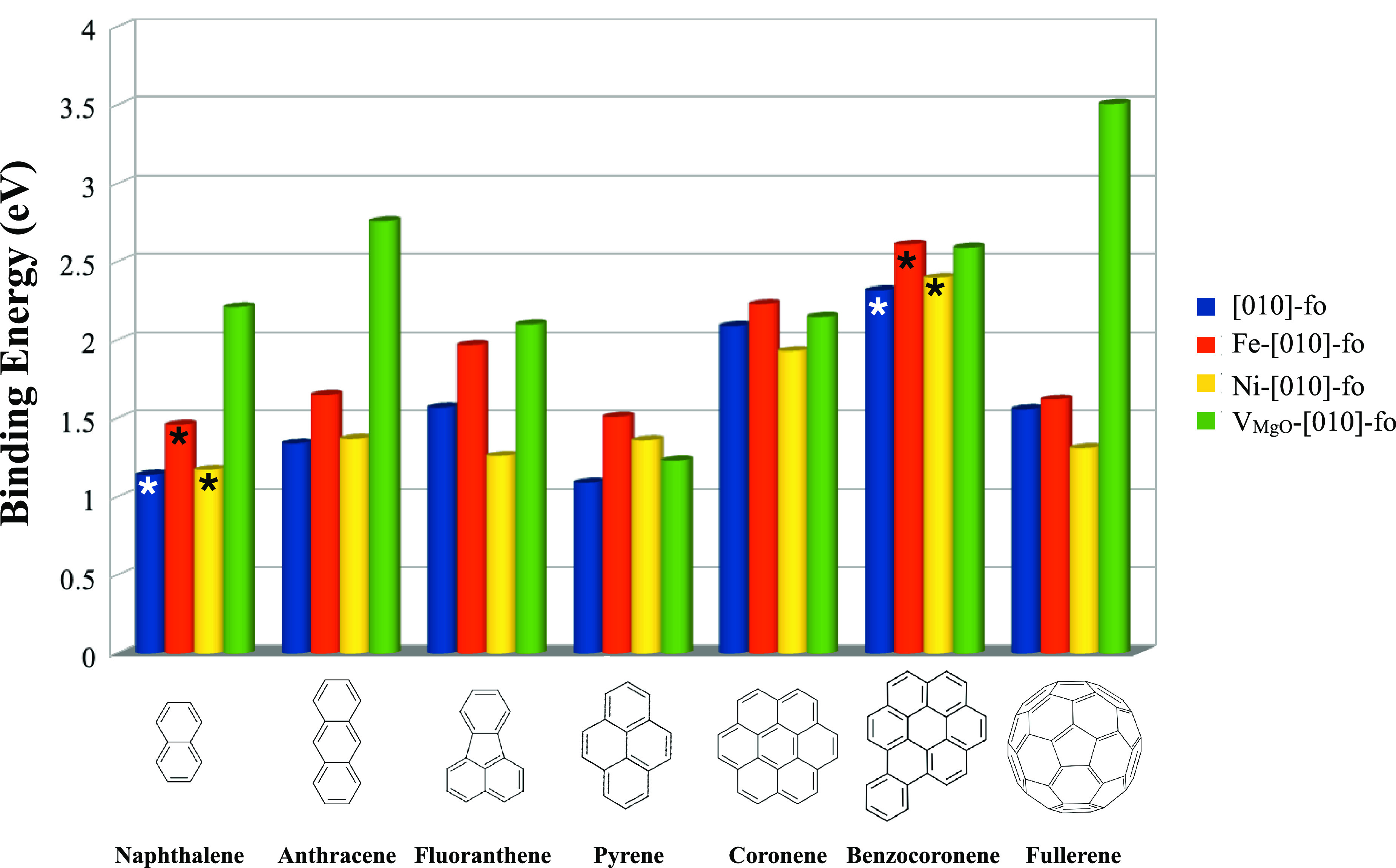
Binding energies of the adsorption of PAHs on [010] forsterite surfaces modeled in this study. The surfaces follow the order shown in the legend for all PAHs. Numerical values are reported in the Supporting Information. The asterisks (*) on the bars show data reported from a prior work.^22^

Figure 8 summarizes the binding energies for the PAHs adsorbed on the four different surfaces considered in this study. Disregarding for the moment the interaction with the Schottky defect, there is a general trend of increasing binding energy with the PAH size/surface area, but those of pyrene and the fullerene form an exception to this, not surprising, given their geometries.

The general increase of the binding energy is attributed to the increasing number of contact points (see Table 2), which is related to the size of the surface area (see Table 1). All PAHs are slightly more stabilized (larger binding energy) on Fe-[010]-fo than on Ni-[010]-fo, which is opposite these species (Fe^2+^ and Ni^2+^) on benzene in the gas phase (gas-phase configuration).^22^ As discussed in the “Interaction of PAHs and C_60_ with Doped Forsterite” section, this reflects subtle geometrical differences in the balance between Mg–PAH and Ni–PAH interactions. PAH adsorption on Fe-doped forsterite shows consistently higher binding energies than that on a pure surface, reflecting that PAHs can optimize their interaction on this surface better than on a Ni-doped surface. The binding energy trend between Fe and the pristine surface is, instead, in agreement with the binding energy trend observed for the binding of Fe^2+^ and Mg^2+^ with benzene in the gas phase.^65^

As shown in Figure 8, all molecules bind strongly on the vacancy surface with binding energies larger than 2 eV. We note that anthracene has larger binding energies than naphthalene, in-line with the reactivity studies conducted by Bonfanti et al.^66^ and Rasmussen.^67^ Anthracene adsorbs on the vacancy surface with the formation of perfect C–Si and C–O bonds and  and  angles of 109° and 120°, respectively. Table 3 shows the binding energy of PAHs and fullerene as a function of the  and  angles (see labels in Figure 6). Excluding the special case of fullerene, the binding energy of PAHs decreases as a function of the angle deviations from 109° and 120°, for  and , respectively. Therefore, anthracene is the PAH that benefits the most from the adsorption process on the vacancy surface. Here, we focus only on the localized effect on the vacancy site as the latter is responsible for chemisorption processes of PAHs, and we will study the effect of the binding of PAHs on the pristine part of the vacancy surface in a follow-up study.

**Table 3:**
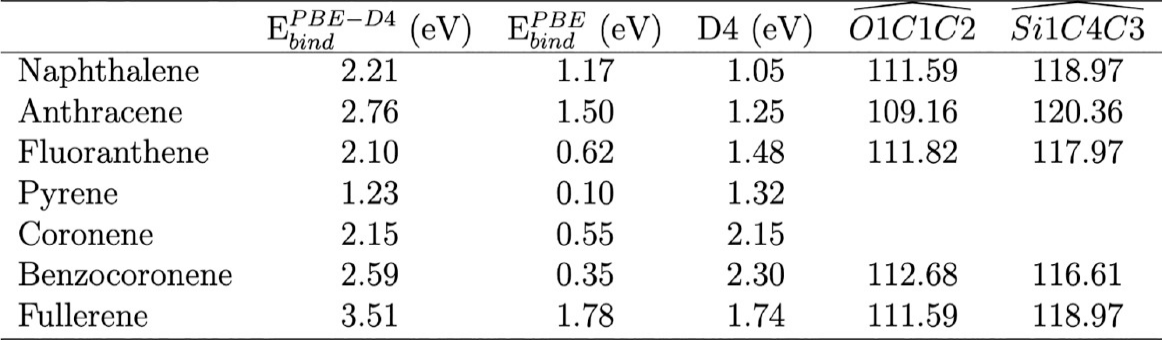
Binding Energies (*E*_bind_), at PBE-D4/DZP and PBE/DZP Levels, Dispersion Energy (D4), and Angles (^) Only for the Chemisorbed Molecules, of the Interaction of PAHs with the V_MgO_-[010]-fo Surface

Fullerene is an exception because it benefits from the formation of covalent bonds with the O and Si atoms on the vacancy and, therefore, from the rehybridization of the C atoms (from tensioned sp^2^ to sp^3^ carbons)^61^ that break the symmetry of the icosahedral structure. Despite the fact that benzocoronene has larger geometrical deviations than other PAHs (see angles in Table 3), its binding energy is closer to the anthracene one because of the higher dispersion energy (see Table 3).

The binding energy of pyrene and coronene deviates with respect to the other PAHs because of their physisorption state (see the discussion in the “Structural Analysis” section and the table in Figure 6).

The difference in binding energies between the vacancy surface and the pristine surfaces is much larger for naphthalene, anthracene, and fullerene than for the other PAHs. The smaller number of contact points for naphthalene and anthracene as compared to other PAHs makes the contribution from the covalent bond formation on the vacancy surface relatively more important. The propensity of fullerenes for addition reactions through the carbons of the six-membered ring facilitates covalent bond formation on the vacancy surface and results in a relatively high binding energy.

#### Adsorption Mechanism

**Figure 9:**
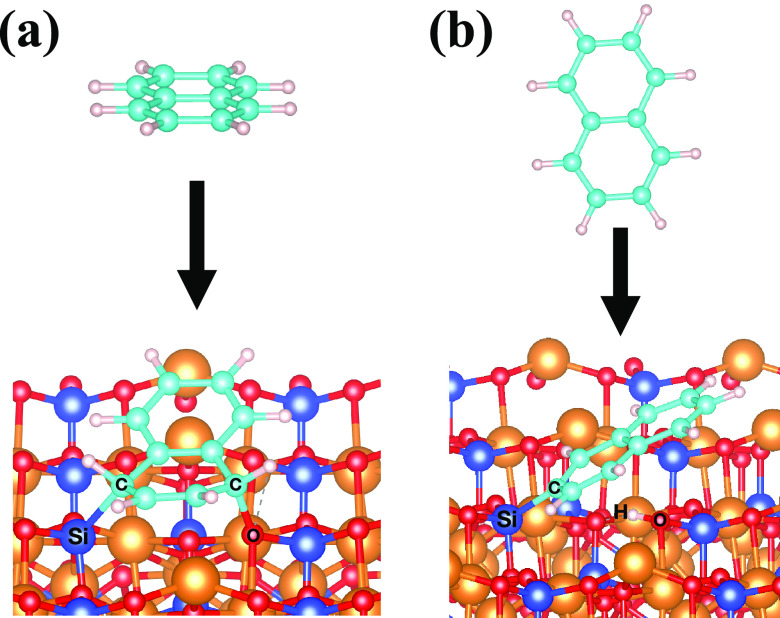
A schematic representation of the (a) parallel and (b) perpendicular adsorption of naphthalene on V_MgO_-[010]-fo. Atomic labels depict the bond formation due to the adsorption.

In the previous section, we saw that PAHs strongly bind onto the vacancy surface with the formation of C–O and C–Si bonds. To evaluate the possible presence of activation barriers associated with the formation of covalent bonds (see the “Structural Analysis” section), we calculated the minimum energy path by employing the CI-NEB approach to locate possible saddle points (transition states); see section “C–H Activation” in the Supporting Information. Because of technical and computational difficulties in optimizing these large molecular interactions with CI-NEB, we considered only the adsorption of naphthalene and benzocoronene on the vacancy surface to address potential differences between small and large PAHs in the chemisorption process. For the PAH approaching the surface in parallel (Figure 9a), the adsorption is studied for both naphthalene and benzocoronene. For a perpendicular orientation (Figure 9b), we studied only naphthalene because of technical difficulties.

#### Parallel Adsorption

When the molecule is oriented parallel to the surface (Figure 9a), the adsorption process is barrierless and has large exoergic adsorption energy of about −2.2 eV for naphthalene. We also reported the minimum energy path for benzocoronene (red curve in Figure S3 in Supporting Information) to check how the path changes for large surface area molecules. Benzocoronene also has a large adsorption energy of about −2.59 eV with a barrierless and exoergic adsorption.

#### Perpendicular Adsorption

When the molecule is oriented perpendicular to the surface (Figure 9b), the adsorption on the V_MgO_-[010]-fo leads, during the optimization process, to the dissociation of the C–H bond of the PAH. In contrast, this process on the pristine and transition-metal surfaces does not lead to dissociation but only adsorption (see Figure 4). On the vacancy, the optimized minimum energy path (Figure S4), with the CI-NEB method, shows a barrierless and exoergic reaction of −3.5 eV. Therefore, the dissociation (perpendicular adsorption) is more favorable than the adsorption (parallel adsorption) by about −1.3 eV.

The electron donation of the C–Si interaction causes, at the same time, the transposition of the hydrogen to the neighboring oxygen atom on the vacancy, forming an O–H bond with a distance of about 0.99 Å, typical for hydroxyl groups.^64^ The last step implies the adsorption of the remaining carbon skeleton on the surface (Figure 9b).

**Figure 10:**
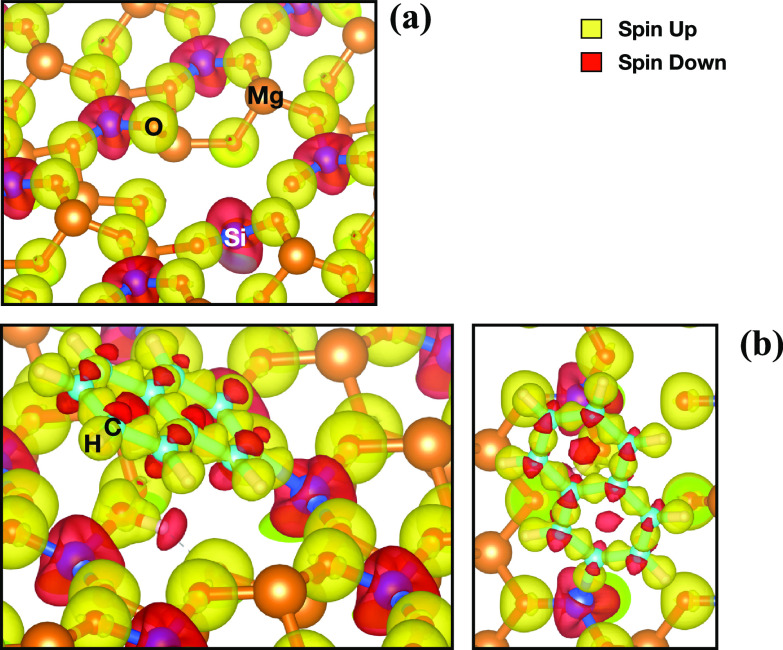
Spin-density isosurface (isovalue 0.007 e/A^3^) of V_MgO_-[010]-fo (a) and the dissociation of the C−H bond of naphthalene on the V_MgO_-[010]-fo (b). Yellow density indicates the spin-up population and red density the spin-down one.

The spin-density isosurfaces show a large population of spin down localized on the Si atoms and spin up localized on the O atoms of the V_MgO_-[010]-fo surface (Figure 10). The Voronoi charge analysis shows a partial positive charge on the Si atom (0.897) and a partial negative charge on the O atom (−0.625). After the dissociation, the partial electronic charge is reduced to 0.625 for the Si atoms, gaining electrons, and −0.356 for the O atom, donating electrons. The Voronoi charge analysis shows a donation of the electrons from the 2p orbital of the C atom of naphthalene to the 3p orbital of the undercoordinated Si atom on the vacancy, whereas the O atom donates electron density to the proton (H) dissociated from the PAH. This behavior is similar to the catalytic effect of frustrated Lewis pairs (FLPs), which are a Lewis acid and base that are not able to bind because of geometrical hindrance. Therefore, the geometrical hindrance causes the increase of the strength of the Lewis acid and base. The simultaneous electron transfer is responsible for the cleavage of the C–H bond through a barrierless reaction promoted by an O and undercoordinated Si atoms, similar to the electron-transfer mechanism of the FLPs.^68^ The formation of a polar cavity, caused by the electric field generated by the presence of FLPs, polarizes the C–H bond (see “C–H Activation” section in the Supporting Information,) allowing the cleavage of the bond. This activation mechanism was first suggested by Grimme et al.^69^ for the cleavage of molecular hydrogen, which goes through a barrierless process once the molecule is inside the cavity and is subjected to the electric field caused by the presence of the FLPs. The presence of a barrier is caused only by the entrance of the molecule into the cavity because of steric hindrance.^69^ As the cavity space between Si and O atoms is almost 5 Å, a terminal C–H bond of a PAH has enough space to enter the cavity. Hence, the C–H cleavage is a barrierless process for this case study. In addition, the O has higher electronic affinity (1.46 eV)^70^ than C (1.26 eV)^71^ with respect to the H (0.75 eV),^72^ and Si has higher electronic affinity (1.39 eV)^70^ than H. Hence, the loss of stability caused by the cleavage of the C–H bond is compensated by the formation of stable C–Si and H–O bonds.

Table 4 reports the zero-point energy corrected adsorption energy for all PAHs on the V_MgO_-[010]-fo based on the two studied mechanisms (perpendicular and parallel). For the parallel adsorption, naphthalene, anthracene, fluoranthene, and benzocoronene show comparable adsorption energies, whereas pyrene show smaller exoergic adsorption energies than other PAHs as discussed in “Binding Energy Analysis” section. For the perperndicular adsorption, the exoergicity of benzocoronene is even larger (−5.48 eV) with respect to those of all PAHs. Anthracene has a slightly lower adsorption energy with respect to naphthalene and fluoranthene. This might reflect the electron density is mainly localized in the central ring of anthracene and is lower in the terminal ring (the ring bound with the surface).^67,73,74^ However, on the vacancy surface, anthracene prefers to bind with the terminal ring, not with the central one, as the latter would require major structural changes. As a result, the perpendicular adsorption energy of anthracene is not as large as those for naphthalene and fluoranthene.

**Table 4:** Zero-Point Energy Corrected Adsorption Energies for the Adsorption of PAHs on V_MgO_-[010]-fo through a Perpendicular (*E*_ads_^⊥^) and Parallel (*E*_ads_^=^) Adsorption

	*E*_ads_^⊥^ (eV)	*E*_ads_^=^ (eV)
naphthalene	–3.73	–2.44
anthracene	–3.51	–2.97
fluoranthene	–3.84	–2.33
pyrene	–4.18	–1.49
coronene	–4.19	–2.43
benzocoronene	–5.48	–2.83

The adsorption energy trend, for the perpendicular adsorption, is the reverse of the parallel adsorption, in which anthracene is more stabilized with respect to other PAHs because of the perfect geometrical fit with the vacancy surface (see the discussion in the “Interaction of PAHs and C_60_ with a Vacancy” section and Table 3).

The C–H dissociation through a perpendicular adsorption mechanism is more energetically favorable and, therefore, more likely to happen with respect to the parallel adsorption (Table 4). On the basis of the Bell–Evans–Polanyi principle,^75,76^ higher adsorption energies are associated with low energy barriers, and because of the large exoergicity, we would expect a barrierless process also for anthracene, fluoranthene, pyrene, and coronene. Furthermore, a perpendicular adsorption does not lead to aromaticity loss as this effect occurs only for the parallel adsorption. The C atom of the PAH bound with a Si atom loses an H atom, which is bound with the neighboring O atom, maintaining the sp^2^ hybridization of the C.

## Astrochemical and Cosmochemical Implications

The study of the interaction of silicate minerals with PAHs in star-forming regions is important because it may relate to the organic composition of comets and asteroids. The latter locks up a variety of organic molecules including the prebiotic building blocks of life as well as PAHs.^18^ During the star formation evolution, silicate dust grains surrounding the protostar coagulate to form planetesimals and cometesimals that eventually will lead to planet formation. Therefore, the cometary and asteroidal composition locks up valuable information about the processes that form molecules in space.^4,18^

In this study, we focused on crystalline forsterite as the main component of silicate grains, asteroids, and meteorites.^77,78^ Silicate minerals in protoplanetary disks and the solar system often have a crystalline nature, as revealed by observations of dust disks around young stellar objects, comets, and asteroids.^79^ In the interstellar medium (ISM), silicates have strong infrared Si–O stretching mode and O–Si–O bending mode absorption features around 9.7 and 18 μm, respectively.^80^ Although IR observations reveal that interstellar silicate grains lack large-scale order,^81^ X-ray absorption studies reveal short-scale crystalline structures.^82^ Possibly, interstellar silicates are polycrystalline, and in that case, their adsorption behavior would be well described by the study presented here. However, in the ISM, there is no direct evidence of the presence of crystalline silicate, and thus, this issue is still under investigation.^77,81^

The composition of the dust, asteroids, and meteorites (silicate materials) is largely dominated by silicate structures belonging to the olivine and pyroxene’s families with stoichiometric formulas Mg_2*x*_Fe_2–2*x*_SiO_4_ and Mg_*x*_Fe_1–*x*_SiO_3_, respectively. The composition of silicate materials in the universe is usually very rich in Mg and poor in Fe (Mg/Fe ratios less than a few percent).^83^ The trace of transition metals is embedded in forsterite as point defects^28^ (the presence of one single atom per surface area), distortionary rearrangement of a perfect crystal lattice, and Mg-, Si-, and MgO-Schottky vacancy.^27,31^ Point defects might already have formed in the envelope of asymptotic giant branch (AGB) stars or may reflect energetic processing of silicate grains in the ISM by, for example, cosmic rays,^84,85^ whereas thermal annealing and irradiation by the stars are the processes taking place in protoplanetary disks.^86^ Molecular dynamics simulations suggest the vacancy formation has been preferentially formed around the AGB stars through O-loss or Schottky defect creation.^31^

Part of the condensed dust in the ISM is formed by PAHs along with other aromatic carbonaceous molecules because of the formation of cluster agglomerates by the π-stacking of their aromatic skeleton.^87,88^ In space, PAHs are important for maintaining the energy and ionization balance in some regions of the interstellar medium as well as for producing the most important molecule in the universe, H_2_.^40,73^ In carbonaceous meteorites, PAHs were hypothesized to be formed by the conversion of aliphatic compounds to aromatic ones through post-Fischer–Tropsch synthesis at low temperature catalyzed by minerals at 360–400 K. However, because of the large abundance of nonalkylated PAHs with temperatures between 400 and 800 K, the hypothesis of PAH formation post-Fischer–Tropsch synthesis was rejected.^89^ Therefore, the origin of PAHs in asteroidal settings is still to be clarified, whereas it is clear that circumstellar regions and the ISM are the most likely places for the formation of these aromatic compounds.^90^

The silicates found in meteorites, specifically carbonaceous chondrites, have a characteristic composition of nonvolatile elements, for example, Fe, Ni, and Mg, in which the abundance is comparable to the abundance of elements observed in the Sun.^91,92^

The abundance of PAHs in the Murchinson carbonaceous meteorite is about 1.82 mmol/g with a large abundance of PAHs with four aromatic units.^93^ PAHs with more than six aromatic units such as coronene (C_24_H_12_) and benzocoronene (C_28_H_14_) have not yet been identified in meteorites. The presence of fullerene in meteorites is suspected and still needs further clarifications.^94^ Therefore, we have considered studying the interaction of coronene and an analogous molecule with an extra hexagonal ring (benzocoronene) along with fullerene to clarify as to why large organic species have not yet been detected.

The potential catalytic activity of forsterite and its defective surfaces, revealed by this study, provides a new view on why large PAHs might not be present in carbonaceous meteorites. The degradation of PAHs, as we saw for the C−H activation on the vacancy surface, is favorable for PAHs up to seven aromatic units. In the ISM the barrier for H-loss from PAHs in the gas phase is 4.7 eV,^95^ and it can be easily promoted by energetic UV-photons (about 12 eV). Astronomical models imply that photodestruction is more rapid for small PAHs than for large PAHs.^6,96^ In our calculations based on the binding and adsorption energies, on a solid surface, we see the opposite trend, and the dissociation rate is more favorable for large PAHs, suggesting that defects in crystalline olivine might catalyze the dissociation of the C–H bond of PAHs in an asteroidal setting. Therefore, this work provides the basis for future astronomical modeling and experimental studies. In particular, the dissociation sequence of each C–H bond of PAHs as well as the estimation of energy barriers for the pristine and metal-doped surfaces needs to be clarified in future works.

## Conclusions

In summary, we present a comprehensive dispersion-corrected density functional theory (DFT-D4) investigation of PAHs with the pristine [010] forsterite surface and its defective surfaces, containing Fe and Ni dopants or an MgO-Schottky vacancy. The lowest-energy adsorption geometries and their binding energetics of naphthalene, anthracene, fluoranthene, pyrene, coronene, benzocoronene, and fullerene on these four [010] forsterite surfaces are unraveled. With the exception of fullerene, all species adsorb on the surfaces maintaining angles and dihedral angles close to the gas-phase configuration. A large surface area allows large contact points (number of atomic interactions) with the surface. Excluding the vacancy surface, we found that all organic species, in this study, present larger binding energy on the Fe-[010]-fo surface with respect to Ni and the pristine surface, which shows the opposite behavior of benzene–cation complexes in the gas phase. These small differences in the binding energy for the adsorption of PAHs on pristine and transition-metal surfaces are caused by differences in the number of contact points. The vacancy surface shows different behaviors with respect to pristine and transition-metal surfaces because of the formation of covalent C–O and C–Si bonds with PAHs. Anthracene has a larger binding energy with respect to other PAHs as a result of the formation of perfect  and  angles of 109° and 120° characteristic of silane complexes. Deviations from these angles are reflected in the differences in binding energy of PAHs on the vacancy surface. Pyrene and coronene physisorb on the vacancy without covalent bond formation. For the case of coronene, the physisorption causes the reconstruction of Si–O bonds on the vacancy. Fullerene adsorption occurs through the formation of C–O and C–Si bonds on the six-membered ring analogous to PAHs chemisorption. The larger binding energy of the chemisorption of fullerene on the vacancy reflects the formation of covalent bonds that break the icosahedral symmetry of the buckyball.

We found that a barrierless adsorption is possible for a parallel orientation of the PAH with respect to the vacancy surface, and a barrierless C−H dissociation is possible in the perpendicular orientation. The latter is energetically more favorable than the parallel adsorption. Therefore, the vacancy has potential catalytic activity for the dissociation of the C−H bond of PAHs. For the C−H dissociation process, the charge analysis has shown an electron donation of the O atoms to the C atoms and electron attraction of the H to the Si atom. This causes splitting of the C–H bond and the formation of C–Si and O–H bonds on the surface. This mechanism is similar to the electron-transfer mechanism of FLPs catalysts.

Hence, forsterite surfaces with a vacancy show potential catalytic activity for the dissociation of aromatic CH. Furthermore, the catalytic potential of forsterite might shed light on the formation of the so-called organic inventory of star-forming regions in space. This study will be followed up with future studies on addressing the selectivity of the vacancy for the dissociation of the hydrogen bound with different carbon sites of the PAHs as well as clarifying the catalytic activity of the pristine surfaces and how to release the fragmented PAH strongly bound to the catalytic site.

## Supporting Information Available

Binding energies, population analysis, C–H activation, geometrical parameters, and cartesian coordinates (PDF)

## References

[ref1] Parshall G. W. Organometallic Chemistry in Homogeneous Catalysis. Science 1980, 208, 1221–1224. 10.1126/science.208.4449.1221. 17830793

[ref2] Nakao Y.; Kashihara N.; Kanyiva K. S.; Hiyama T. Nickel-Catalyzed Alkenylation and Alkylation of Fluoroarenes via Activation of CH Bond over CF Bond. J. Am. Chem. Soc. 2008, 130, 16170–16171. 10.1021/ja807258m. 18998690

[ref3] Segawa Y.; Stephan D. W. Metal-Free Hydrogenation Catalysis of Polycyclic Aromatic Hydrocarbons. Chem. Commun. 2012, 48, 11963–11965. 10.1039/c2cc37190a. 23128319

[ref4] Tielens A. G. G. M. The Molecular Universe. Rev. Mod. Phys. 2013, 85, 1021–1081. 10.1103/RevModPhys.85.1021.

[ref5] Davico G. E.; Bierbaum V. M.; DePuy C. H.; Ellison G. B.; Squires R. R. The C-H Bond Energy of Benzene. J. Am. Chem. Soc. 1995, 117, 2590–2599. 10.1021/ja00114a023.

[ref6] Andrews H.; Candian A.; Tielens A. G. G. M. Hydrogenation and Dehydrogenation of Interstellar PAHs: Spectral Characteristics and H_2_ Formation. Astron. Astrophys. 2016, 595, A23 10.1051/0004-6361/201628819.

[ref7] Arndtsen B. A.; Bergman R. G.; Mobley T. A.; Peterson T. H. Selective Intermolecular Carbon-Hydrogen Bond Activation by Synthetic Metal Complexes in Homogeneous Solution. Acc. Chem. Res. 1995, 28, 154–162. 10.1021/ar00051a009.

[ref8] Thalji R. K.; Ahrendt K. A.; Bergman R. G.; Ellman J. A. Annulation of Aromatic Imines via Directed CH Activation with Wilkinson’s Catalyst. J. Am. Chem. Soc. 2001, 123, 9692–9693. 10.1021/ja016642j. 11572698

[ref9] Jia C.; Piao D.; Oyamada J.; Lu W.; Kitamura T.; Fujiwara Y. Efficient Activation of Aromatic C-H Bonds for Addition to C-C Multiple Bonds. Science 2000, 287, 1992–1995. 10.1126/science.287.5460.1992. 10720319

[ref10] Gorelsky S. I. Origins of Regioselectivity of the Palladium-Catalyzed (Aromatic) CH Bond Metalation–Deprotonation. Coord. Chem. Rev. 2013, 257, 153–164. 10.1016/j.ccr.2012.06.016.

[ref11] Cho J.-Y.; Tse M. K.; Holmes D.; Maleczka R. E.; Smith M. R. Remarkably Selective Iridium Catalysts for the Elaboration of Aromatic C-H Bonds. Science 2002, 295, 305–308. 10.1126/science.1067074. 11719693

[ref12] Egorova K. S.; Ananikov V. P. Which Metals are Green for Catalysis? Comparison of the Toxicities of Ni, Cu, Fe, Pd, Pt, Rh, and Au Salts. Angew. Chem., Int. Ed. 2016, 55, 12150–12162. 10.1002/anie.201603777. 27532248

[ref13] Stephan D.; Erker G. Frustrated Lewis Pairs: Metal-free Hydrogen Activation and More. Angew. Chem., Int. Ed. 2010, 49, 46–76. 10.1002/anie.200903708. 20025001

[ref14] Légaré M.-A.; Courtemanche M.-A.; Rochette É.; Fontaine F.-G. Metal-free Catalytic C-H Bond Activation and Borylation of Heteroarenes. Science 2015, 349, 513–516. 10.1126/science.aab3591. 26228143

[ref15] Abu El-Rub Z.; Bramer E. A.; Brem G. Review of Catalysts for Tar Elimination in Biomass Gasification Processes. Ind. Eng. Chem. Res. 2004, 43, 6911–6919. 10.1021/ie0498403.

[ref16] Rimola A.; Sodupe M.; Ugliengo P. Role of Mineral Surfaces in Prebiotic Chemical Evolution. In Silico Quantum Mechanical Studies; Life: Basel, Switzerland, 2019; Vol. 9, p 10. 10.3390/life9010010 PMC6463156 30658501

[ref17] James Cleaves H. II; Michalkova Scott A.; Hill F. C.; Leszczynski J.; Sahai N.; Hazen R. Mineral–Organic Interfacial Processes: Potential Roles in the Origins of Life. Chem. Soc. Rev. 2012, 41, 5502–5525. 10.1039/c2cs35112a. 22743683

[ref18] Ehrenfreund P.; Charnley S. B. Organic Molecules in the Interstellar Medium, Comets, and Meteorites: A Voyage from Dark Clouds to the Early Earth. Annu. Rev. Astron. Astrophys. 2000, 38, 427–483. 10.1146/annurev.astro.38.1.427.

[ref19] Zamirri L.; Corno M.; Rimola A.; Ugliengo P. Forsterite Surfaces as Models of Interstellar Core Dust Grains: Computational Study of Carbon Monoxide Adsorption. ACS Earth Space Chem. 2017, 1, 384–398. 10.1021/acsearthspacechem.7b00041.

[ref20] Li Q.; Dai W.; Liu B.; Sarre P.; Xie M.; Cheung A.-C. Catalytic Conversion of Methanol to Larger Organic Molecules Over Crystalline Forsterite: Laboratory Study and Astrophysical Implications. Mol. Astrophys. 2018, 13, 22–29. 10.1016/j.molap.2018.09.002.

[ref21] Ferrante R.; Moore M.; Nuth J.; Smith T. Laboratory Studies of Catalysis of CO to Organics on Grain Analogs. Icarus 2000, 145, 297–300. 10.1006/icar.2000.6350.

[ref22] Campisi D.; Lamberts T.; Dzade N. Y.; Martinazzo R.; ten Kate I. L.; Tielens A. G. G. M. Interaction of Aromatic Molecules with Forsterite: Accuracy of the Periodic DFT-D4Method. J. Phys. Chem. A 2021, 125, 2770–2781. 10.1021/acs.jpca.1c02326. 33784098 PMC8154625

[ref23] Rimola A.; Trigo-Rodríguez J. M.; Martins Z. Interaction of Organic Compounds with Chondritic Silicate Surfaces. Atomistic Insights from Quantum Chemical Periodic Simulations. Phys. Chem. Chem. Phys. 2017, 19, 18217–18231. 10.1039/C7CP03504G. 28682400

[ref24] Asaduzzaman A.; Zega T.; Laref S.; Runge K.; Deymier P.; Muralidharan K. A Computational Investigation of Adsorption of Organics on Mineral Surfaces: Implications for Organics Delivery in the Early Solar System. Earth Planet. Sci. Lett. 2014, 408, 355–361. 10.1016/j.epsl.2014.10.029.

[ref25] Caldeweyher E.; Mewes J.-M.; Ehlert S.; Grimme S. Extension and Evaluation of the D4 London-Dispersion Model for Periodic Systems. Phys. Chem. Chem. Phys. 2020, 22, 8499–8512. 10.1039/D0CP00502A. 32292979

[ref26] Watson G. W.; Oliver P. M.; Parker S. C. Computer Simulation of the Structure and Stability of Forsterite Surfaces. Phys. Chem. Miner. 1997, 25, 70–78. 10.1007/s002690050088.

[ref27] de Leeuw N. H. Density Functional Theory Calculations of Hydrogen-Containing Defects in Forsterite, Periclase, and α-Quartz. J. Phys. Chem. B 2001, 105, 9747–9754. 10.1021/jp0109978.

[ref28] Fei H.; Katsura T. Si and O Self-Diffusion in Hydrous Forsterite and Iron-Bearing Olivine from the Perspective of Defect Chemistry. Phys. Chem. Miner. 2016, 43, 119–126. 10.1007/s00269-015-0779-0.

[ref29] Navarro-Ruiz J.; Ugliengo P.; Rimola A.; Sodupe M. B3LYP Periodic Study of the Physicochemical Properties of the Nonpolar (010) Mg-Pure and Fe-Containing Olivine Surfaces. J. Phys. Chem. A 2014, 118, 5866–5875. 10.1021/jp4118198. 24517343

[ref30] Navarro-Ruiz J.; Ugliengo P.; Sodupe M.; Rimola A. Does Fe^2+^ in Olivine-Based Interstellar Grains Play Any Role in the Formation of H_2_? Atomistic Insights from DFT Periodic Simulations. Chem. Commun. 2016, 52, 6873–6876. 10.1039/C6CC02313D. 27103407

[ref31] Quadery A. H.; Pacheco S.; Au A.; Rizzacasa N.; Nichols J.; Le T.; Glasscock C.; Schelling P. K. Atomic-Scale Simulation of Space Weathering in Olivine and Orthopyroxene. J. Geophys. Res. Planets 2015, 120, 643–661. 10.1002/2014JE004683.

[ref32] Schwartz K.; Lang M. In Encyclopedia of Geochemistry: A Comprehensive Reference Source on the Chemistry of the Earth; White W. M., Ed.; Springer International Publishing: Cham, 2016; pp 1–5.

[ref33] Palme H.; Lodders K.; Jones A. In Treatise on Geochemistry, 2nd ed.; Holland H. D., Turekian K. K., Eds.; Elsevier: Oxford, 2014; pp 15–36.

[ref34] Westphal A. J.; et al. Evidence for Interstellar Origin of Seven Dust Particles Collected by the Stardust Spacecraft. Science 2014, 345, 786–791. 10.1126/science.1252496. 25124433

[ref35] Brodholt J. P.; Refson K. An ab Initio Study of Hydrogen in Forsterite and a Possible Mechanism for Hydrolytic Weakening. J. Geophys. Res. Solid Earth 2000, 105, 18977–18982. 10.1029/2000JB900057.

[ref36] Cami J. Can Fullerene Analogues be the Carriers of the Diffuse Interstellar Bands?. Proc. Int. Astron. Union 2013, 9, 370–374. 10.1017/S1743921313016141.

[ref37] Maier J. P.; Campbell E. K. Fullerenes in Space. Angew. Chem., Int. Ed. 2017, 56, 4920–4929. 10.1002/anie.201612117. 28070989

[ref38] Watson G. W.; Kelsey E. T.; de Leeuw N. H.; Harris D. J.; Parker S. C. Atomistic Simulation of Dislocations, Surfaces and Interfaces in MgO. J. Chem. Soc., Faraday Trans. 1996, 92, 433–438. 10.1039/ft9969200433.

[ref39] Cortese R.; Campisi D.; Duca D. Hydrogen Arrangements on Defective Quasi-Molecular BN Fragments. ACS Omega 2019, 4, 14849–14859. 10.1021/acsomega.9b01445. 31552324 PMC6751536

[ref40] Campisi D.; Candian A. Do Defects in PAHs Promote Catalytic Activity in Space? Stone–Wales Pyrene as a Test Case. Phys. Chem. Chem. Phys. 2020, 22, 6738–6748. 10.1039/C9CP06523G. 32167097

[ref41] Radzig V. In Physico-Chemical Phenomena in Thin Films and at Solid Surfaces; Trakhtenberg L. I., Lin S. H., Ilegbusi O. J., Eds.; Thin Films and Nanostructures; Academic Press, 2007; Vol. 34; pp 231–345.

[ref42] Soler J. M.; Artacho E.; Gale J. D.; García A.; Junquera J.; Ordejón P.; Sánchez-Portal D. The SIESTA Method for Ab Initio Order-N Materials Simulation. J. Phys.: Condens. Matter 2002, 14, 2745 10.1088/0953-8984/14/11/302. 21693870

[ref43] Perdew J. P.; Burke K.; Ernzerhof M. Generalized Gradient Approximation Made Simple. Phys. Rev. Lett. 1996, 77, 3865–3868. 10.1103/PhysRevLett.77.3865. 10062328

[ref44] Boys S.; Bernardi F. The Calculation of Small Molecular Interactions by the Differences of Separate Total Energies. Some Procedures with Reduced Errors. Mol. Phys. 1970, 19, 553–566. 10.1080/00268977000101561.

[ref45] Sordo J. A.; Chin S.; Sordo T. L. On the Counterpoise Correction for the Basis Set Superposition Error in Large Systems. Theor. Chem. Acc. 1988, 74, 101–110. 10.1007/BF00528320.

[ref46] Caldeweyher E.; Ehlert S.; Hansen A.; Neugebauer H.; Spicher S.; Bannwarth C.; Grimme S. A Generally Applicable Atomic-Charge Dependent London Dispersion Correction. J. Chem. Phys. 2019, 150, 154122 10.1063/1.5090222. 31005066

[ref47] Caldeweyher E.; Bannwarth C.; Grimme S. Extension of the D3 Dispersion Coefficient Model. J. Chem. Phys. 2017, 147, 034112 10.1063/1.4993215. 28734285

[ref48] Monkhorst H. J.; Pack J. D. Special Points for Brillouin-Zone Integrations. Phys. Rev. B 1976, 13, 5188–5192. 10.1103/PhysRevB.13.5188.

[ref49] Liu D. C.; Nocedal J. On the Limited Memory BFGS Method for Large Scale Optimization. Math. Program. 1989, 45, 503–528. 10.1007/BF01589116.

[ref50] Fonseca Guerra C.; Handgraaf J.-W.; Baerends E. J.; Bickelhaupt F. M. Voronoi Deformation Density (VDD) Charges: Assessment of the Mulliken, Bader, Hirshfeld, Weinhold, and VDD Methods for Charge Analysis. J. Comput. Chem. 2004, 25, 189–210. 10.1002/jcc.10351. 14648618

[ref51] Dudarev S. L.; Botton G. A.; Savrasov S. Y.; Humphreys C. J.; Sutton A. P. Electron-Energy-loss Spectra and the Structural Stability of Nickel Oxide: An LSDA+U Study. Phys. Rev. B 1998, 57, 1505–1509. 10.1103/PhysRevB.57.1505.

[ref52] Shankland T. J. Band Gap of Forsterite. Science 1968, 161, 51–53. 10.1126/science.161.3836.51. 17756514

[ref53] Henkelman G.; Uberuaga B. P.; Jónsson H. A Climbing Image Nudged Elastic Band Method for Finding Saddle Points and Minimum Energy Paths. J. Chem. Phys. 2000, 113, 9901–9904. 10.1063/1.1329672.

[ref54] Larsen A. H.; et al. The Atomic Simulation Environment-A Python Library for Working with Atoms. J. Phys.: Condens. Matter 2017, 29, 273002 10.1088/1361-648X/aa680e. 28323250

[ref55] Bahn S. R.; Jacobsen K. W. An Object-Oriented Scripting Interface to a Legacy Electronic Structure Code. Comput. Sci. Eng. 2002, 4, 56–66. 10.1109/5992.998641.

[ref56] Bitzek E.; Koskinen P.; Gähler F.; Moseler M.; Gumbsch P. Structural Relaxation Made Simple. Phys. Rev. Lett. 2006, 97, 170201 10.1103/PhysRevLett.97.170201. 17155444

[ref57] Garrido Torres J. A.; Jennings P. C.; Hansen M. H.; Boes J. R.; Bligaard T. Low-Scaling Algorithm for Nudged Elastic Band Calculations Using a Surrogate Machine Learning Model. Phys. Rev. Lett. 2019, 122, 156001 10.1103/PhysRevLett.122.156001. 31050513

[ref58] Navarro-Ruiz J.; Ugliengo P.; Rimola A.; Sodupe M. B3LYP Periodic Study of the Physicochemical Properties of the Nonpolar (010) Mg-Pure and Fe-Containing Olivine Surfaces. J. Phys. Chem. A 2014, 118, 5866–5875. 10.1021/jp4118198. 24517343

[ref59] Brodholt J. Ab Initio Calculations on Point Defects in Forsterite (Mg_2_SiO_4_) and Implications for Diffusion and Creep. Am. Mineral. 1997, 82, 1049–1053. 10.2138/am-1997-11-1201.

[ref60] Stoneham A. M. The Theory of Defects in Solids. Contemp. Phys. 1979, 20, 535–545. 10.1080/00107517908210920.

[ref61] Rodríguez-Fortea A.; Irle S.; Poblet J. M. Fullerenes: Formation, Stability, and Reactivity. WIREs Comput. Mol. Sci. 2011, 1, 350–367. 10.1002/wcms.21.

[ref62] Simon A.; Joblin C. Thermochemistry and Infrared Spectroscopy of Neutral and Cationic IronPolycyclic Aromatic Hydrocarbon Complexes of Astrophysical Interest: Fundamental Density Functional Theory Studies. J. Phys. Chem. A 2007, 111, 9745–9755. 10.1021/jp072506a. 17850049

[ref63] Schwieger S.; Wagner C.; Bruhn C.; Schmidt H.; Steinborn D. Synthesis and Structures of Zeise-type Complexes with Vinylsilane Ligands and Quantum Chemical Analysis of the Platinum–Vinylsilane Bonding. Z. Anorg. Allg. Chem. 2005, 631, 2696–2704. 10.1002/zaac.200500110.

[ref64] Johnson R. D. III. NIST Computational Chemistry Comparison and Benchmark Database, NIST Standard Reference Database Number 101, Release 21. 2020.

[ref65] Kolakkandy S.; Pratihar S.; Aquino A. J. A.; Wang H.; Hase W. L. Properties of Complexes Formed by Na^+^, Mg^2+^, and Fe^2+^ Binding with Benzene Molecules. J. Phys. Chem. A 2014, 118, 9500–9511. 10.1021/jp5029257. 25144574

[ref66] Bonfanti M.; Casolo S.; Tantardini G. F.; Ponti A.; Martinazzo R. A few simple rules governing hydrogenation of graphene dots. J. Chem. Phys. 2011, 135, 164701 10.1063/1.3650693. 22047257

[ref67] Rasmussen J. A. Polycyclic Aromatic Hydrocarbons: Trends for Bonding Hydrogen. J. Phys. Chem. A 2013, 117, 4279–4285. 10.1021/jp400287h. 23621608

[ref68] Rokob T. A.; Bakó I.; Stirling A.; Hamza A.; Pápai I. Reactivity Models of Hydrogen Activation by Frustrated Lewis Pairs: Synergistic Electron Transfers or Polarization by Electric Field?. J. Am. Chem. Soc. 2013, 135, 4425–4437. 10.1021/ja312387q. 23432375

[ref69] Grimme S.; Kruse H.; Goerigk L.; Erker G. The Mechanism of Dihydrogen Activation by Frustrated Lewis Pairs Revisited. Angew. Chem., Int. Ed. 2010, 49, 1402–1405. 10.1002/anie.200905484. 20091722

[ref70] Chaibi W.; Peláez R. J.; Blondel C.; Drag C.; Delsart C. Effect of a Magnetic Field in Photodetachment Microscopy. Eur. Phys. J. D 2010, 58, 29–37. 10.1140/epjd/e2010-00086-7.

[ref71] Bresteau D.; Drag C.; Blondel C. Isotope Shift of the Electron Affinity of Carbon Measured by Photodetachment Microscopy. Phys. Rev. A 2016, 93, 013414 10.1103/PhysRevA.93.013414.

[ref72] Lykke K. R.; Murray K. K.; Lineberger W. C. Threshold Photodetachment of H^–^. Phys. Rev. A 1991, 43, 6104–6107. 10.1103/PhysRevA.43.6104. 9904944

[ref73] Campisi D.; Simonsen F. D. S.; Thrower J. D.; Jaganathan R.; Hornekær L.; Martinazzo R.; Tielens A. G. G. M. Superhydrogenation of Pentacene: the Reactivity of Zigzag-Edges. Phys. Chem. Chem. Phys. 2020, 22, 1557–1565. 10.1039/C9CP05440E. 31872819

[ref74] Fukui K.; Yonezawa T.; Shingu H. A Molecular Orbital Theory of Reactivity in Aromatic Hydrocarbons. J. Chem. Phys. 1952, 20, 722–725. 10.1063/1.1700523.

[ref75] Bell R. P. The Theory of Reactions Involving Proton Transfers. Proc. R. Soc. London A 1936, 154, 414–429. 10.1098/rspa.1936.0060.

[ref76] Evans M. G.; Polanyi M. Inertia and Driving Force of Chemical Reactions. Trans. Faraday Soc. 1938, 34, 11–24. 10.1039/tf9383400011.

[ref77] Brucato J. R.; Strazzulla G.; Baratta G.; Colangeli L. Forsterite Amorphisation by Ion Irradiation: Monitoring by Infrared Spectroscopy. Astron. Astrophys. 2004, 413, 395–401. 10.1051/0004-6361:20031574.

[ref78] Reddy V.; Dunn T. L.; Thomas C. A.; Moskovitz N. A.; Burbine T. H. Asteroids IV; University of Arizona Press, 2015; pp 43–63.

[ref79] Draine B. Interstellar Dust Grains. Annu. Rev. Astron. Astrophys. 2003, 41, 241–289. 10.1146/annurev.astro.41.011802.094840.

[ref80] Draine B. T. Interstellar Extinction in the Infrared. In Infrared Spectroscopy in Astronomy; European Space Agency: 1989; p 93.

[ref81] Kemper F.; Vriend W. J.; Tielens A. G. G. M. The Absence of Crystalline Silicates in the Diffuse Interstellar Medium. Astrophys. J. 2004, 609, 826–837. 10.1086/421339.

[ref82] Zeegers S. T.; Costantini E.; Rogantini D.; de Vries C. P.; Mutschke H.; Mohr P.; de Groot F.; Tielens A. G. G. M. Dust Absorption and Scattering in the Silicon K-Edge. Astron. Astrophys. 2019, 627, A16 10.1051/0004-6361/201935050.

[ref83] Molster F.; Kemper C. Crystalline Silicates. Space Sci. Rev. 2005, 119, 3–28. 10.1007/s11214-005-8066-x.

[ref84] Bringa E. M.; Kucheyev S. O.; Loeffler M. J.; Baragiola R. A.; Tielens A. G. G. M.; Dai Z. R.; Graham G.; Bajt S.; Bradley J. P.; Dukes C. A.; Felter T. E.; Torres D. F.; Breugel W. v. Energetic Processing of Interstellar Silicate Grains by Cosmic Rays. Astrophys. J. 2007, 662, 372–378. 10.1086/517865.

[ref85] Carrez P.; Demyk K.; Cordier P.; Gengembre L.; Grimblot J.; D’hendecourt L.; Jones A. P.; Leroux H. Low-Energy Helium Ion Irradiation-Induced Amorphization and Chemical Changes in Olivine: Insights for Silicate Dust Evolution in the Interstellar Medium. Meteorit. Planet. Sci. 2002, 37, 1599–1614. 10.1111/j.1945-5100.2002.tb00814.x.

[ref86] Henning T. Cosmic Silicates. Annu. Rev. Astron. Astrophys. 2010, 48, 21–46. 10.1146/annurev-astro-081309-130815.

[ref87] Rapacioli M.; Calvo F.; Spiegelman F.; Joblin C.; Wales D. J. Stacked Clusters of Polycyclic Aromatic Hydrocarbon Molecules. J. Phys. Chem. A 2005, 109, 2487–2497. 10.1021/jp046745z. 16833550

[ref88] Rapacioli M.; Joblin C.; Boissel P. Spectroscopy of Polycyclic Aromatic Hydrocarbons and Very Small Grains in Photodissociation Regions. Astron. Astrophys. 2005, 429, 193–204. 10.1051/0004-6361:20041247.

[ref89] Wing M. R.; Bada J. L. The Origin of the Polycyclic Aromatic Hydrocarbons in Meteorites. Orig. Life Evol. Biosph. 1991, 21, 375–383. 10.1007/BF01808308.

[ref90] Cherchneff I. The formation of Polycyclic Aromatic Hydrocarbons in evolved circumstellar environments. EAS Publ. Ser. 2011, 46, 177–189. 10.1051/eas/1146019.

[ref91] Lodders K. Solar System Abundances and Condensation Temperatures of the Elements. Astrophys. J. 2003, 591, 1220–1247. 10.1086/375492.

[ref92] Anders E.; Grevesse N. Abundances of the Elements: Meteoritic and Solar. Geochim. Cosmochim. Acta 1989, 53, 197–214. 10.1016/0016-7037(89)90286-X.

[ref93] Huang Y.; Aponte J. C.; Zhao J.; Tarozo R.; Hallmann C. Hydrogen and Carbon Isotopic Ratios of Polycyclic Aromatic Compounds in Two CM2 Carbonaceous Chondrites and Implications for Prebiotic Organic Synthesis. Earth Planet. Sci. Lett. 2015, 426, 101–108. 10.1016/j.epsl.2015.06.027.

[ref94] Hammond M. R.; Zare R. N. Identifying the Source of a Strong Fullerene Envelope Arising from Laser Desorption Mass Spectrometric Analysis of Meteoritic Insoluble Organic Matter. Geochim. Cosmochim. Acta 2008, 72, 5521–5529. 10.1016/j.gca.2008.08.008.

[ref95] Wiersma S. D.; Candian A.; Bakker J. M.; Martens J.; Berden G.; Oomens J.; Buma W. J.; Petrignani A. Photolysis-Induced Scrambling of PAHs as a Mechanism for Deuterium Storage. Astron. Astrophys. 2020, 635, A9 10.1051/0004-6361/201936982.

[ref96] Allain T.; Leach S.; Sedlmayr E. Photodestruction of PAHs in the Interstellar Medium. I. Photodissociation Rates for the Loss of an Acetylenic Group. Astron. Astrophys. 1996, 305, 602–615.

