# Supplementary material for: Adsorption of Polycyclic Aromatic Hydrocarbons and C60 onto Forsterite: C–H Bond Activation by the Schottky Vacancy
Source: ACS Earth Space Chem. 2022 Jul 27;6(8):2009–23. doi: 10.1021/acsearthspacechem.2c00084 (PMC9393896; doi:10.1021/acsearthspacechem.2c00084)
Supplement: Supplementary file 1 — sp2c00084_si_001.pdf [file sp2c00084_si_001.pdf]

# Supporting information (SI) for: Adsorption of PAHs and $C_{60}$ onto Forsterite: C-H Bond Activation by the Schottky Vacancy

Dario Campisi,[*,†,‡] Thanja Lamberts,[¶,†] Nelson Y. Dzade,[§,‖] Rocco Martinazzo,[⊥] Inge Loes ten Kate,[#] and Alexander G. G. M. Tielens[†]

†*Leiden Observatory, Leiden University, Niels Bohrweg 2, 2333 CA Leiden, The Netherlands*

‡*Current Address: Department of Chemistry, The University of Chicago, 5735 S Ellis Ave, Chicago, IL 60637, USA*

¶*Leiden Institute of Chemistry, Leiden University, Einsteinweg 55, 2300 RA Leiden, The Netherlands*

§*Cardiff University, Main Building, Park Place, CF10 3AT Cardiff, U.K.*

‖*Current Address: John and Willie Leone Family Department of Energy and Mineral Engineering, The Pennsylvania State University, 212 Hosler Building, University Park, PA 16802, USA*

⊥*Department of Chemistry, Università degli Studi di Milano, Via Golgi 19, 20133 Milan, Italy*

#*Department of Earth Sciences, Faculty of Geosciences, Utrecht University, Princetonlaan 8a, 3584 CB Utrecht, The Netherlands*

E-mail: campisi@uchicago.edu,campisi@strw.leidenuniv.nl

# Binding Energies

Table S1: Binding energies ($E_{bind}$) of the adsorption of PAHs and fullerene onto [010] forsterite surfaces.

| | $E_{bind}^{[010]-fo}$ (eV) | $E_{bind}^{Fe-[010]-fo}$ (eV) | $E_{bind}^{Ni-[010]-fo}$ (eV) | $E_{bind}^{V_{MgO}-[010]-fo}$ (eV) |
|---|---|---|---|---|
| naphthalene | 1.14[1] | 1.46[1] | 1.17[1] | 2.21 |
| anthracene | 1.34 | 1.65 | 1.37 | 2.76 |
| fluoranthene | 1.57 | 1.97 | 1.26 | 2.1 |
| pyrene | 1.09 | 1.51 | 1.36 | 1.23 |
| coronene | 2.09 | 2.23 | 1.93 | 2.15 |
| benzocoronene | 2.32[1] | 2.61[1] | 2.4[1] | 2.59 |
| fullerene | 1.56 | 1.62 | 1.31 | 3.51 |

# Population Analysis

The electronic structure and the nature of chemical bonding are studied with the projected density of state (PDOS), the crystal orbital overlap (COOP), and hamilton population (COHP).[2] The theoretical level used for the PDOS, COOP, and COHP, consists of a PBE+U/TZP single-point calculation on the PBE/DZP optimized geometry (explained in detail in a previous work[1]).

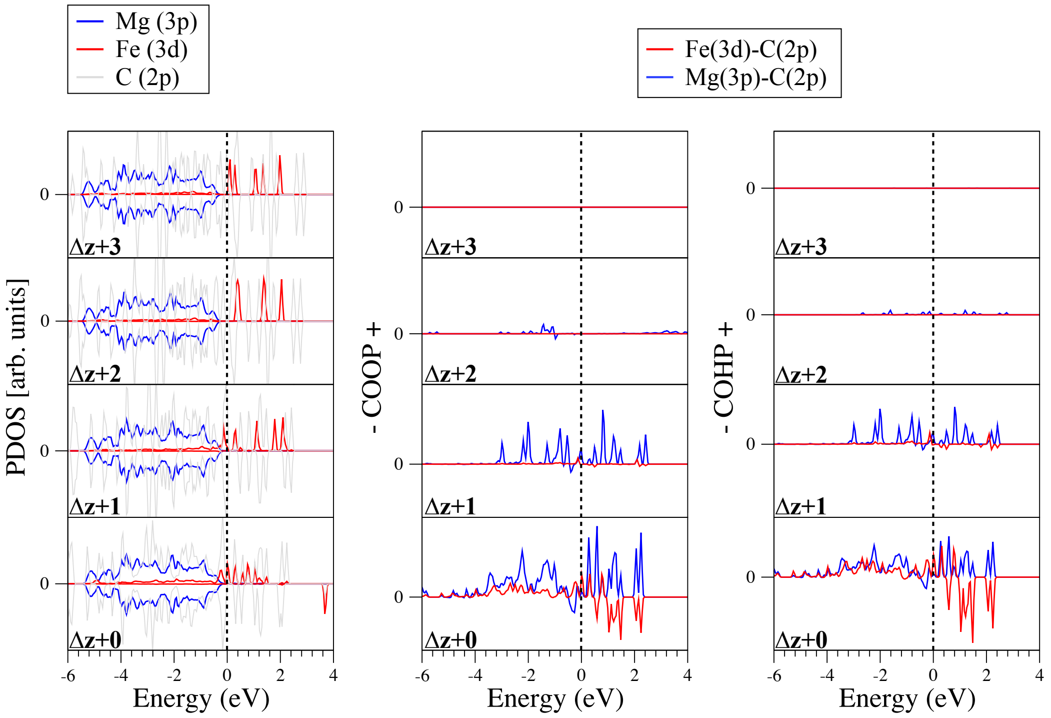


Figure S1: PDOS, COOP, and COHP of benzocoronene adsorbed on Fe-[010]-fo at different distances from the surface (Δz=+0 Å,+1 Å,+2 Å,+3 Å) with the respective atomic legend reported. For the PDOS, the peaks down and up refer to spin down and up, respectively. For the COOP and COHP, positive values are bonding contribution and negative ones are anti-bonding contribution. The Fermi level has been shifted at 0 eV.

The binding energy analysis provides meaningful insights into understanding the strength of the adsorption of the four forsterite surfaces. However, the only binding energy analysis does not provide information on the bonding nature between the molecule and the surface. Therefore, we calculated, as test-case, the PDOS, COOP, and COHP only for the adsorption of the PAH that has large binding energy when adsorbed on the forsterite surfaces: benzocoronene on Fe-[010]-fo and $V_{MgO}$-[010]-fo, for instance. We decided to consider the only benzocoronene in view of its large surface area (40.73 Å), and for the surface, we limit ourselves to Fe-[010]-fo and $V_{MgO}$-[010]-fo since those are the surfaces that show higher binding energy for most of the cases of this study. Specifically, we calculated the PDOS of benzocoronene at different distances from the surface (0, 1, 2 and 3 Å far from the surface). We

reported only the valence band region since we are interested in shedding light on the binding nature of the molecular interaction with the surface. In Fig. S1, we report the PDOS for the benzocoronene adsorbed on the Fe-[010]-fo surface. The valence band is dominated by the electrons of the 2p orbitals of the O atoms, followed by the electrons of the 3p orbitals of the Mg atoms and 3d orbitals of the Fe atoms. When benzocoronene is interacting with the surface ($\Delta z$+0 Å), we notice an overcrowded region of small peaks belonging to orbitals of C and Fe that suggest orbital interaction. Once the molecule moves away from the surface the region of the Fermi level becomes less busy with larger gaps between the peaks of all three atoms reflecting the reduced interaction of the molecule with the surface.

The PDOS provides a good description of the electronic contribution of the atoms to the overall band structure, however, does not provide information about the bonding and antibonding nature of these electrons. Therefore, we partitioned the density of state in the bonding and antibonding contribution of the specific atoms (COOP spectra). Fig. S1 reports the COOP spectra for the orbital interaction between Mg and C atoms as well as Fe and C atoms. The upward and downward peaks reflect bonding and anti-bonding contribution, respectively. Qualitatively, we see that the bonding contribution prevails with respect to the antibonding contribution. Once the molecule is 1 Å away from the surface ($\Delta z$+1 Å), the antibonding contribution disappears since the electron-electron repulsion is lower. Once the molecule is 2 and 3 Å away ($\Delta z$+2 Å and $\Delta z$+3 Å, respectively) from the surface there is not bonding contribution since the electronic interaction becomes weaker.

The COHP is the same as the COOP, but in this case, we partition the band energy rather than the electronic structure. Therefore, the COHP usually is the opposite of the COOP, but for simplicity, we inverted the sign of the peaks for ease of comparison to COOP. As expected, COHP has a similar trend with respect to COOP for the case of benzocoronene on Fe-[010]-fo, shown in Fig. S1.

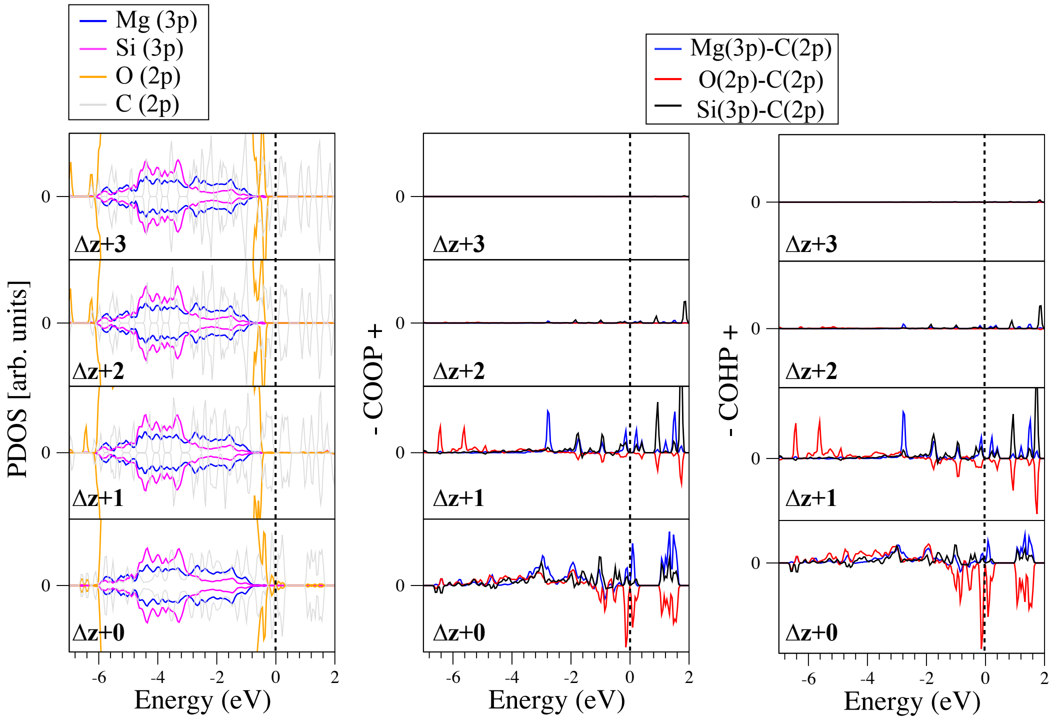


Figure S2: PDOS, COOP, and COHP of benzocoronene adsorbed on $V_{MgO}$-[010]-fo at different distances from the surface (Δz=+0 Å,+1 Å,+2 Å,+3 Å) with the respective atomic legend reported. For the PDOS, the peaks down and up refer to spin down and up, respectively. For the COOP and COHP, positive values are bonding contribution and negative ones are anti-bonding contribution. The Fermi level has been shifted at 0 eV.

For the interaction of benzocoronene with the $V_{MgO}$-[010]-fo, Fig. S2, the PDOS is dominated by the electron of the 2p orbitals of the O atoms followed by 3p orbitals of Mg, 3p orbitals of Si, in line with the PDOS bulk structure of forsterite,[1] and 2p orbitals of the C atoms. Due to the presence of three different types of interaction, it is difficult to analyze substantial variations in the PDOS at three different distances from the surface. Instead, the COOP and COHP show non-bonding peaks as well as larger bonding peaks for the interaction of O-C orbitals. This suggests the presence of a covalent bond formation since the electrons of the 2p orbitals of C atoms come close to the electrons of the 2p orbital of the O atoms repelling each other.

# C-H Activation

Fig. S3 shows the minimum energy path for the adsorption process of naphthalene and benzocoronene leading to the chemisorption of the C atoms, in para (opposite carbon sites of the hexagonal ring), forming C-Si and C-O bonds (image 8 in Fig. S3). Image 1 represents an optimized geometry of the PAH at a distance of about 5.6 Å from the surface. Image 8 is the optimized PAH chemisorbed on the vacancy, which is the final product. The benzocoronene

Figure S3: Optimized minimum energy path of the adsorption of naphthalene (black curve) and benzocoronene (red curve) on $V_{MgO}$-[010]-fo. The optimized geometry, only for naphthalene, of all images is reported below the plot. Image zero is the surface and the molecule at infinite distance.

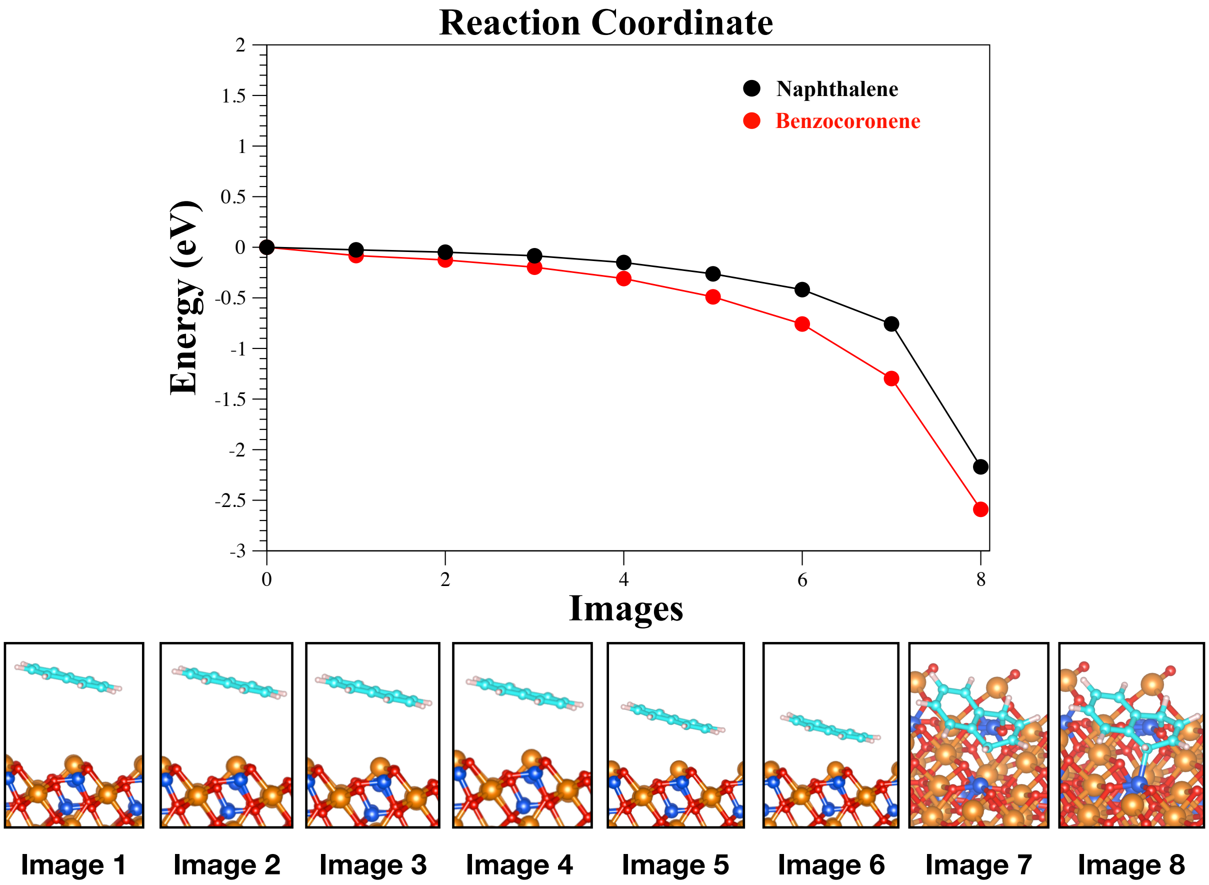


images are lower in energy with respect to the naphthalene ones due to the larger dispersion energy with respect to naphthalene.

Fig. S4 shows the minimum energy path for the perpendicular adsorption of naphthalene

Figure S4: Optimized minimum energy path of the CH dissociation of naphthalene on $V_{MgO}$-[010]-fo. The optimized geometry of all images is reported below the plot. Image zero is the surface and the molecule at infinite distance.

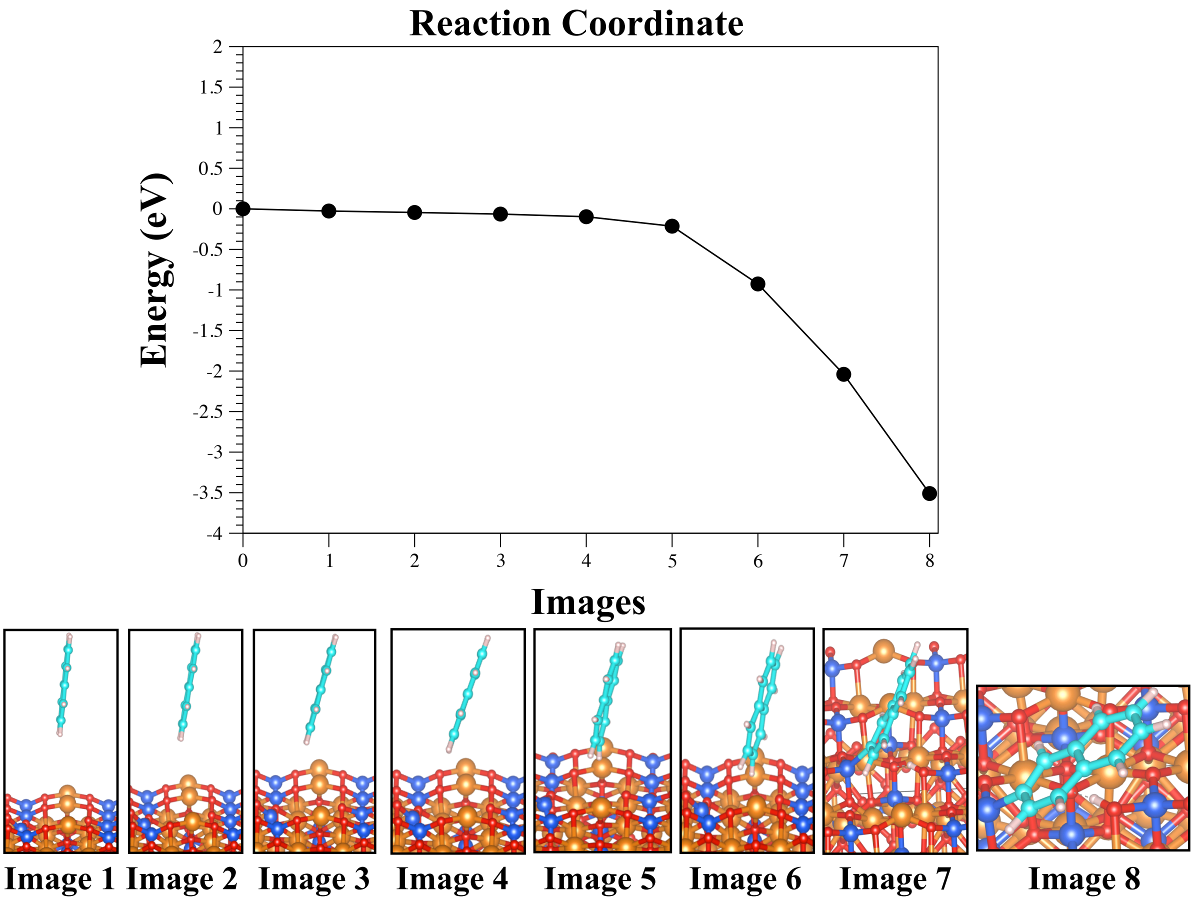

on $V_{MgO}$-[010]-fo. The reagent (image 1) has been optimized perpendicularly and distant about 6.5 Å with respect to the vacancy surface. Along the reaction path, the molecule approaches the surface, chemisorbing a carbon atom on the under-coordinated Si atom on the vacancy (C-Si interaction). This resulted in a C-Si bond length of about 2.13 Å compared to typical C-Si bond length in silane species: 1.87 Å.[3] Both parallel and perpendicular adsorptions lead to barrierless reaction. To confirm the absence of barriers, we recalculated the minimum energy path considering Image 6 and 7 of Fig. S4 as reagent and product, respectively, interpolating them with further 6 images and optimizing them. The resulting optimized images were further interpolated by 6 images to focus only on the C-H activation region and the resulting optimized path is reported in Fig. S5. The spin density and electrostatic potential isosurfaces of all images are reported in Fig. S6, which show polar conditions of the vacancy structure that polarizes the C-H bond as shown also by the Voronoi charges at different C-Si and O-H distances in Table S2.

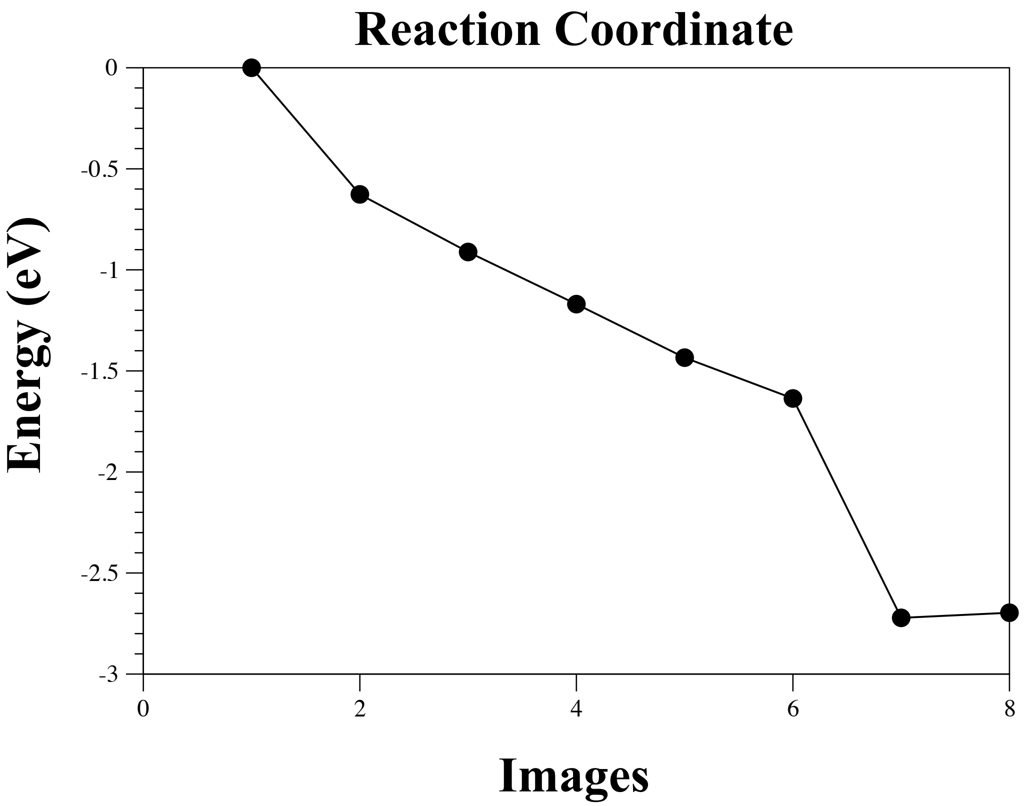


Figure S5: Optimized minimum energy path of the CH dissociation of naphthalene on $V_{MgO}$-[010]-fo. The optimized geometry of all images is reported in Fig. S6.

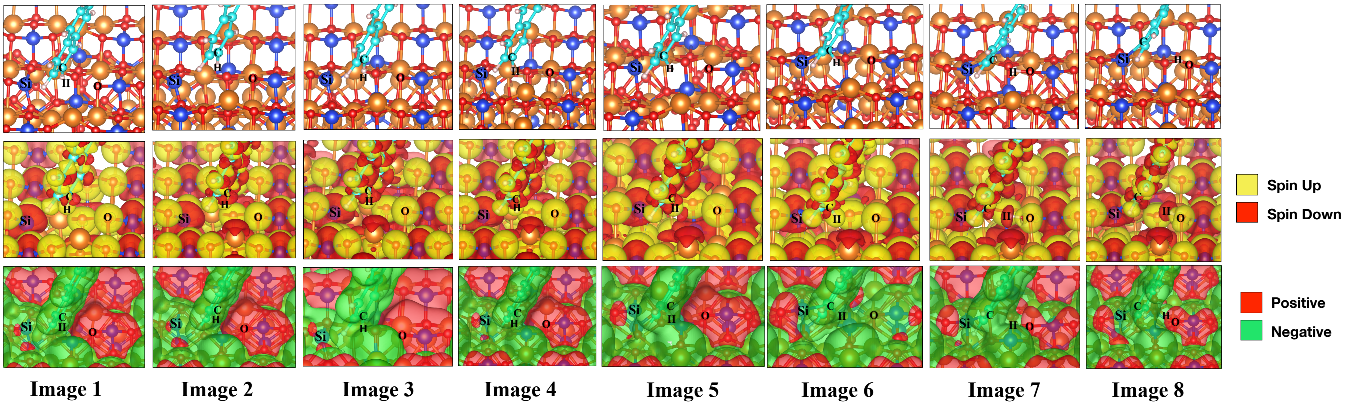


Figure S6: Optimized geometry, spin density and electrostatic potential isosurfaces from the top to bottom of each images. For spin density, yellow shows spin up population and red spin down population (isovalue 0.005 e/A$^3$). For the electrostatic potential, red shows positive potential and green negative potential (isovaleu 0.1 e/A$^3$). The reported images are referred to the optimized minimum energy path reported in Fig. S5.

Table S2: Distances (d) in Å and Voronoi charges (V) in atomic units. The reported images are referred to the optimized minimum energy path reported in Fig. S5

| | d(Si - O) | d(C - H) | d(C - Si) | d(O - H) | V(C — H) | V(Si - O) |
|---|---|---|---|---|---|---|
| Image 1 | 4.99 | 1.10 | 3.42 | 2.75 | 0.029 — 0.094 | 0.838 — -0.617 |
| Image 2 | 5.03 | 1.10 | 3.67 | 2.37 | 0.031 — 0.069 | 0.856 — -0.613 |
| Image 3 | 4.99 | 1.10 | 3.40 | 2.44 | 0.028 — 0.102 | 0.835 — -0.618 |
| Image 4 | 4.89 | 1.11 | 3.02 | 2.53 | 0.022 — 0.128 | 0.799 — -0.623 |
| Image 5 | 4.66 | 1.12 | 2.48 | 2.34 | -0.006 — 0.159 | 0.727 — -0.629 |
| Image 6 | 4.55 | 1.13 | 2.20 | 2.19 | -0.043 — 0.078 | 0.623 — -0.019 |
| Image 7 | 4.27 | 1.80 | 2.03 | 1.80 | -0.089 — 0.216 | 0.647 — -0.398 |
| Image 8 | 4.42 | 2.73 | 2.13 | 0.99 | -0.069 — 0.207 | 0.649 — -0.338 |

# Geometrical Parameters

In the previous section (Population Analysis), we see there is not orbital interaction when the molecule is 3 Å away from the surface. Therefore, we report the geometrical parameters considering only C-Mg and C-M (M is a transition metal) interactions with distances less than 3 Å.

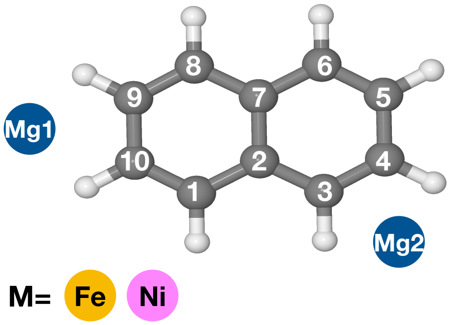


| | [010]-fo | Fe-[010]-fo | Ni-[010]-fo | $V_{MgO}$-[010]-fo |
|---|---|---|---|---|
| Mg1-C9 | 2.73 | | | |
| Mg1-C10 | 2.50 | | | |
| Mg2-C2 | 2.80 | | | |
| Mg2-C3 | 2.46 | 2.53 | | |
| Mg2-C4 | 2.59 | 2.50 | 2.44 | |
| Mg2-C5 | | | 2.85 | |
| M-C1 | | 2.26 | 2.19 | |
| M-C10 | | 2.14 | 2.22 | |
| M-C9 | | 2.89 | 2.86 | |
| Si-C8 | | | | 1.98 |
| O-C1 | | | | 1.46 |
| $\widehat{C4C3C2}$ | 120.42 | 120.55 | 119.92 | 120.85 |
| $\widehat{C10C1C2}$ | 120.00 | 119.50 | 120.17 | 110.07 |
| $\Theta(C4C3C2C1)$ | 173.06 | 179.33 | 179.81 | -176.04 |
| $\Theta(C10C1C2C3)$ | 179.30 | 164.75 | -176.48 | 148.04 |

Figure S7: Schematic figure of naphthalene structure and its atomic labels interacting with Mg and transition metals atoms (Fe and Ni) on the surface. Table of bond distances (-) in Angstrom (Å), angles ( $\widehat{\ }$ ) and dihedral angles ($\Theta$), in degree (°), of the interaction of naphthalene with the [010] forsterite surfaces. The missing values indicate bond distances larger than 3 Å. The geometrical parameters of this molecule adsorbed on [010]-fo, Fe-[010]-fo, and Ni-[010]-fo are calculated from the optimized geometry of prior work.[1]

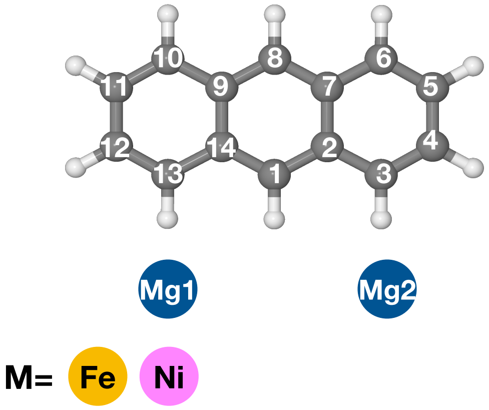


| | [010]-fo | Fe-[010]-fo | Ni-[010]-fo | $V_{MgO}$-[010]-fo |
|---|---|---|---|---|
| Mg2-C2 | 2.63 | | | |
| Mg2-C3 | 2.45 | | | |
| Mg2-C4 | 2.64 | 2.81 | 2.93 | |
| Mg2-C5 | | | | 2.70 |
| Mg1-C11 | | 2.87 | 2.80 | |
| Mg1-C12 | 2.75 | 2.43 | 2.46 | |
| Mg1-C13 | 2.50 | | | |
| Mg1-C14 | 2.94 | | | |
| M-C14 | | 2.65 | 2.58 | |
| M-C1 | | 2.14 | 2.15 | |
| M-C2 | | 2.42 | 2.51 | |
| Si-C10 | | | | 2.00 |
| O-C13 | | | | 1.46 |
| $\widehat{C12C13C14}$ | 119.94 | 120.28 | 120.02 | 110.02 |
| $\widehat{C14C1C2}$ | 121.15 | 121.17 | 121.25 | 121.17 |
| $\widehat{C2C3C4}$ | 121.38 | 119.95 | 119.71 | 121.04 |
| $\Theta(C12C13C14C1)$ | -179.46 | -178.41 | 176.72 | 150.19 |
| $\Theta(C1C2C3C4)$ | 166.67 | -174.89 | -177.39 | 173.75 |

Figure S8: Schematic figure of anthracene structure and its atomic labels interacting with Mg and transition metals atoms (Fe and Ni) on the surface. Table of bond distances (-) in Angstrom (Å), angles ( $\widehat{\ }$ ) and dihedral angles ($\Theta$), in degree (°), of the interaction of anthracene with the [010] forsterite surfaces. The missing values indicate bond distances larger than 3 Å.

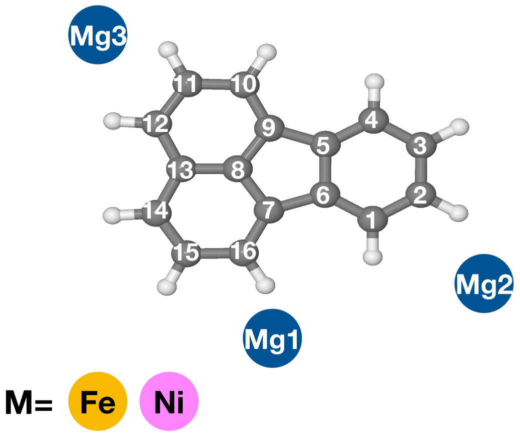


| | [010]-fo | Fe-[010]-fo | Ni-[010]-fo | $V_{MgO}$-[010]-fo |
|---|---|---|---|---|
| Mg1-C16 | 2.41 | | | |
| Mg1-C13 | | | | 2.68 |
| Mg1-C14 | | | | 2.88 |
| Mg1-C15 | 2.71 | | | |
| Mg1-C7 | 2.71 | | | |
| Mg2-C2 | 2.44 | 2.43 | 2.51 | |
| Mg2-C1 | 2.91 | | | |
| Mg2-C3 | 2.86 | 2.70 | 2.53 | 2.93 |
| Mg3-C11 | 2.76 | 2.74 | | |
| M-C15 | | 2.96 | | |
| M-C16 | | 2.17 | | |
| M-C7 | | 2.23 | 2.66 | |
| M-C6 | | 2.88 | | |
| M-C8 | | | 2.32 | |
| M-C9 | | | 2.66 | |
| M-C13 | | | 2.86 | |
| Si-C1 | | | | 2 |
| O-C4 | | | | 1.47 |
| $\widehat{C14C15C16}$ | 122.13 | 122.99 | 122.78 | 123.06 |
| $\widehat{C16C7C6}$ | 134.71 | 135.79 | 135.74 | 135.99 |
| $\widehat{C6C1C2}$ | 118.24 | 118.46 | 118.58 | 110.57 |
| $\widehat{C14C13C12}$ | 126.81 | 127.20 | 128.73 | 128.05 |
| $\widehat{C13C8C7}$ | 124.80 | 124.21 | 124.26 | 125.03 |
| $\Theta(C15C16C7C6)$ | -169.04 | 175.58 | -176.31 | -179.59 |
| $\Theta(C2C1C6C7)$ | 178.30 | 179.04 | 178.82 | -153.39 |
| $\Theta(C14C13C12C11)$ | 179.53 | 174.07 | -176.44 | 178.13 |
| $\Theta(C13C8C7C6)$ | 171.84 | 179.28 | 179.76 | 177.89 |

Figure S9: Schematic figure of fluoranthene structure and its atomic labels interacting with Mg and transition metals atoms (Fe and Ni) on the surface. Table of bond distances (-) in Angstrom (Å), angles ( $\widehat{\ }$ ) and dihedral angles ($\Theta$), in degree (°), of the interaction of fluoranthene with the [010] forsterite surfaces. The missing values indicate bond distances larger than 3 Å.

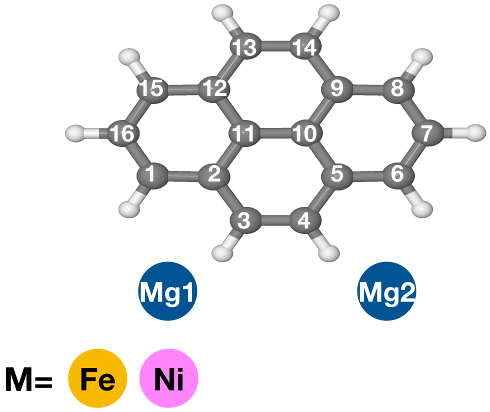


| | [010]-fo | Fe-[010]-fo | Ni-[010]-fo | $V_{MgO}$-[010]-fo |
|---|---|---|---|---|
| Mg1-C2 | 2.70 | | | |
| Mg1-C11 | 2.55 | | | |
| Mg1-C12 | 2.99 | | | |
| Mg2-C6 | 2.78 | 2.39 | 2.46 | |
| Mg2-C7 | 2.61 | 2.91 | 2.70 | |
| Mg2-C5 | | 2.62 | 2.81 | 2.83 |
| Mg2-C4 | | | | 2.67 |
| M-C16 | | 2.97 | | |
| M-C1 | | 2.22 | 2.40 | |
| M-C2 | | 2.30 | 2.17 | |
| M-C3 | | 2.96 | 2.67 | |
| M-C11 | | | 2.78 | |
| Si-C16 | | | | 2.25 |
| Si-C1 | | | | 2.83 |
| $\widehat{C1C2C3}$ | 120.34 | 121.98 | 122.01 | 120.91 |
| $\widehat{C4C5C6}$ | 122.18 | 121.99 | 120.84 | 122.48 |
| $\widehat{C2C11C10}$ | 119.84 | 119.59 | 119.73 | 120.23 |
| $\Theta(C1C2C3C4)$ | 179.28 | -172.83 | -178.45 | 175.47 |
| $\Theta(C4C5C6C7)$ | 178.38 | 175.94 | 176.18 | 176.01 |
| $\Theta(C1C2C11C10)$ | 179.80 | 177.14 | 178.30 | -169.24 |

Figure S10: Schematic figure of pyrene structure and its atomic labels interacting with Mg and transition metals atoms (Fe and Ni) on the surface. Table of bond distances (-) in Angstrom (Å), angles ( $\widehat{}$ ) and dihedral angles ($\Theta$), in degree (°), of the interaction of pyrene with the [010] forsterite surfaces. The missing values indicate bond distances larger than 3 Å.

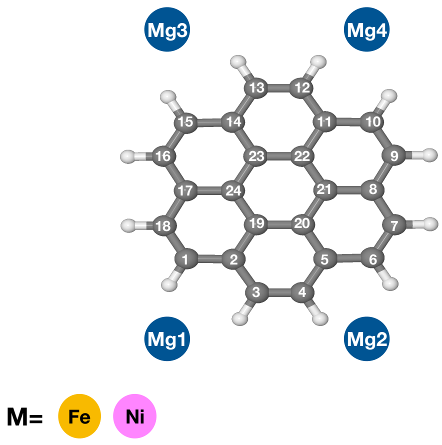


| | [010]-fo | Fe-[010]-fo | Ni-[010]-fo | $V_{MgO}$-[010]-fo |
|---|---|---|---|---|
| Mg1-C1 | 2.57 | | 2.50 | 2.59 |
| Mg1-C2 | 2.80 | | | |
| Mg1-C18 | | | 2.83 | 2.50 |
| Mg2-C6 | 2.51 | 2.41 | | |
| Mg2-C5 | 2.63 | 2.81 | | |
| Mg2-C7 | | 2.87 | | |
| Mg2-C4 | 2.99 | | | |
| Mg4-C10 | 2.43 | 2.46 | | |
| Mg4-C11 | | 2.69 | | |
| Mg4-C9 | 2.91 | | | |
| Mg4-C12 | | | 2.52 | 2.40 |
| Mg4-C13 | | | 2.69 | 2.54 |
| Mg3-C11 | 2.77 | | | 2.90 |
| Mg3-C15 | 2.93 | 2.60 | 2.88 | |
| Mg3-C14 | 2.76 | 2.60 | | |
| Mg3-C13 | 2.83 | | | |
| O-C3 | | | | 2.28 |
| M-C1 | | 2.84 | | |
| M-C2 | | 2.41 | | |
| M-C3 | | 2.28 | | |
| M-C6 | | | 2.55 | |
| M-C5 | | | 2.28 | |
| M-C20 | | | 2.54 | |

Figure S11: Schematic figure of coronene structure and its atomic labels interacting with Mg and transition metals atoms (Fe and Ni) on the surface. Table of bond distances (-), in Angstrom (Å), for the interaction of coronene with the [010] forsterite surfaces. The missing values indicate bond distances larger than 3 Å.

Table S3: Table of angles ( $\widehat{\ }$ ) and dihedral angles ($\Theta$), in degree ($^\circ$), of the interaction of coronene with the [010] forsterite surfaces. The atomic labels are referred to Fig. S11. The geometrical parameters of this molecule adsorbed on [010]-fo, Fe-[010]-fo, and Ni-[010]-fo are calculated from the optimized geometry of prior work.[1]

| | [010]-fo | Fe-[010]-fo | Ni-[010]-fo | $V_{MgO}$-[010]-fo |
|---|---|---|---|---|
| $\widehat{C18C1C2}$ | 120.61 | 120.89 | 121 | 121.47 |
| $\widehat{C5C6C7}$ | 121.19 | 121.10 | 120.35 | 120.94 |
| $\widehat{C9C10C11}$ | 121.00 | 120.99 | 121.05 | 120.50 |
| $\widehat{C16C15C14}$ | 120.88 | 120.63 | 120.86 | 120.96 |
| $\widehat{C18C17C16}$ | 121.87 | 121.90 | 121.70 | 121.88 |
| $\widehat{C17C24C23}$ | 119.99 | 120.29 | 119.82 | 119.58 |
| $\Theta(C18C1C2C3)$ | 178.05 | -173.03 | -177.82 | 179.96 |
| $\Theta(C7C6C5C4)$ | 173.25 | 170.68 | -175.06 | -175.68 |
| $\Theta(C9C10C11C12)$ | -170.93 | -174.64 | -176.72 | 176.45 |
| $\Theta(C16C15C14C13)$ | 176.15 | -178.00 | -179.91 | -176.78 |
| $\Theta(C17C24C13C22)$ | -179.76 | 177.57 | -179.98 | 174.68 |

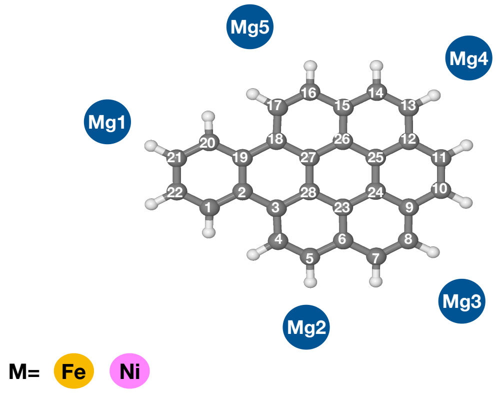


| | [010]-fo | Fe-[010]-fo | Ni-[010]-fo | $V_{MgO}$-[010]-fo |
|---|---|---|---|---|
| Mg1-C21 | 2.59 | | | |
| Mg1-C20 | 2.70 | | | |
| Mg2-C5 | 2.50 | | | |
| Mg2-C6 | 2.68 | | | |
| Mg2-C22 | | 2.70 | 2.74 | |
| Mg3-C7 | | 2.55 | 2.54 | |
| Mg3-C8 | | 2.53 | 2.55 | |
| Mg3-C6 | | 2.89 | 2.87 | |
| Mg3-C24 | | | | 2.67 |
| Mg3-C25 | | | | 2.61 |
| Mg4-C13 | 2.54 | 2.50 | 2.51 | |
| Mg4-C14 | | 2.79 | 2.79 | |
| Mg5-C15 | 2.91 | | | |
| Mg5-C16 | 2.71 | 2.56 | 2.61 | |
| Mg5-C17 | 2.62 | 2.76 | 2.73 | |
| Mg5-C18 | 2.77 | | | |
| Mg5-C15 | 2.91 | | | |
| Mg5-C27 | 2.93 | | | |
| M-C3 | | 2.28 | 2.35 | |
| M-C4 | | 2.47 | 2.41 | |
| M-C2 | | 2.65 | 2.68 | |
| Si-C20 | | | | 2.02 |
| O-C1 | | | | 1.46 |

Figure S12: Schematic figure of benzocoronene structure and its atomic labels interacting with Mg and transition metals atoms (Fe and Ni) on the surface. Table of bond distances (-), in Angstrom (Å), for the interaction of benzocoronene with the [010] forsterite surfaces. The missing values indicate bond distances larger than 3 Å. The geometrical parameters of this molecule adsorbed on [010]-fo, Fe-[010]-fo, and Ni-[010]-fo are calculated from the optimized geometry of prior work.[1]

Table S4: Table of angles ( $\frown$ ) and dihedral angles ($\Theta$), in degree ($^\circ$), of the interaction of benzocoronene with the [010] forsterite surfaces. The atomic labels are referred to Fig. S12.

| | [010]-fo | Fe-[010]-fo | Ni-[010]-fo | $V_{MgO}$-[010]-fo |
|---|---|---|---|---|
| $\widehat{C20C21C22}$ | 119.71 | 119.55 | 119.48 | 122.23 |
| $\widehat{C6C5C4}$ | 120.91 | 120.80 | 120.58 | 121.35 |
| $\widehat{C12C13C14}$ | 120.95 | 121 | 121 | 120.42 |
| $\widehat{C17C16C15}$ | 120.88 | 120.51 | 120.66 | 120.77 |
| $\Theta(C21C20C19C18)$ | 178.80 | 179.77 | -179.28 | -151.08 |
| $\Theta(C17C16C15C14)$ | 174.51 | -177.02 | -178.34 | 165.39 |
| $\Theta(C4C5C6C7)$ | -175.56 | -179.91 | -179.92 | -168.25 |
| $\Theta(C20C19C18C17)$ | -9.65 | 3.67 | 4.74 | 7.98 |
| $\Theta(C1C2C3C4)$ | 3.87 | -4.51 | 1.58 | -1.51 |

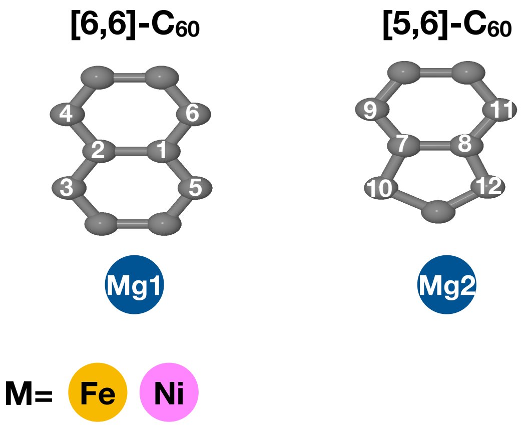


| | [010]-fo | Fe-[010]-fo | Ni-[010]-fo | $V_{MgO}$-[010]-fo |
|---|---|---|---|---|
| Mg1-C1 | 2.56 | | | |
| Mg1-C2 | 2.77 | | | |
| Mg2-C7 | 2.77 | | 2.81 | 2.90 |
| Mg2-C8 | 2.88 | | | 2.59 |
| Mg2-C11 | | | | 2.93 |
| M-C1 | | 2.15 | 2.03 | |
| M-C2 | | 2.06 | 2.15 | |
| Si-C5 | | | | 1.99 |
| O-C3 | | | | 1.44 |
| $\widehat{C9C7C10}$ | 118.81 | | 119.62 | 123.23 |
| $\widehat{C11C8C12}$ | 119.57 | | | 120.01 |
| $\widehat{C4C2C3}$ | 108.23 | 107.32 | 107.62 | 110.02 |
| $\widehat{C5C1C6}$ | 107.34 | 106.97 | 107.71 | 108.90 |
| $\Theta(C10C7C8C11)$ | 140.12 | | 143.19 | 153.27 |
| $\Theta(C3C2C1C6)$ | 137.89 | 133.63 | | 147.90 |

Figure S13: Schematic figure of fullerene [6,6] and [6,5] ring junctions and their atomic labels interacting with Mg and transition metals atoms (Fe and Ni) on the surface. Table of bond distances (-) in Angstrom (Å), angles ( $\widehat{}$ ) and dihedral angles (Θ), in degree (°), of the interaction of pyrene with the [010] forsterite surfaces. The missing values indicate bond distances larger than 3 Å.

# References


(1) Campisi, D.; Lamberts, T.; Dzade, N. Y.; Martinazzo, R.; ten Kate, I. L.; Tielens, A. G. G. M. Interaction of Aromatic Molecules with Forsterite: Accuracy of the Periodic DFT-D4 Method. *J. Phys. Chem. A* **2021**, *125*, 2770–2781.

(2) Hughbanks, T.; Hoffmann, R. Chains of Trans-Edge-Sharing Molybdenum Octahedra: Metal-Metal Bonding in Extended Systems. *J. Am. Chem. Soc.* **1983**, *105*, 3528–3537.

(3) Russell D. Johnson III, NIST Computational Chemistry Comparison and Benchmark Database, NIST Standard Reference Database Number 101, Release 21. 2020.

# Cartesian Coordinates

Cartesian coordinates of the most important adsorptions. The adsorption of naphthalene and benzocoronene on [010]-fo, Fe-[010] and Ni-[010] are reported in the supporting information of our prior study (J. Phys. Chem. A 2021, 125, 13, 2770–2781).

**Naphthalene $V_{MgO}$-[010]-fo (=)**

Number of atoms: 352

Mg 2.543480 1.494800 0.388370
Mg 0.008060 5.935140 2.261970
Mg 0.008060 3.033660 2.261970
Mg 2.347542 4.483490 4.514346
Mg 4.717108 1.483305 5.176744
Mg 2.382339 0.037717 7.433479
Mg 2.380777 2.932007 7.429180
Mg 0.174146 4.474925 9.280334
Si 2.727970 4.484400 1.221250
Si 2.018610 1.494800 3.313620
Si 4.379343 4.470890 6.384389
Si 0.354768 1.490071 8.461571
O 3.434710 5.847120 0.556660
O 3.434700 3.121670 0.556660
O 1.102310 4.484400 1.386100
O 1.223890 1.494800 1.760420
O 3.466830 4.484400 2.799080
O 3.663830 1.494800 3.191290
O 1.257330 0.166960 4.002710
O 1.257330 2.822640 4.002710
O 3.632802 5.790362 5.684497
O 3.629361 3.146443 5.693565
O 1.278559 4.476490 6.501496
O 1.090345 1.486117 6.894735
O 3.583770 4.470861 7.925591
O 3.481011 1.485353 8.304149
O 1.052717 0.135201 9.135526
O 1.056161 2.847349 9.129209
Mg 2.543480 7.474000 0.388370
Mg 0.008060 11.914340 2.261970
Mg 0.008060 9.012860 2.261970
Mg 2.363168 10.464101 4.537040
Mg 4.719178 7.480699 5.180162

Mg 2.367682 6.013483 7.422069

Mg 2.385988 8.919228 7.452422

Mg 0.183200 10.460004 9.278261

Si 2.727970 10.463600 1.221250

Si 2.018610 7.474000 3.313620

Si 4.400096 10.453762 6.436186

Si 0.345450 7.470706 8.462446

O 3.434710 11.826320 0.556660

O 3.434710 9.100870 0.556660

O 1.102310 10.463600 1.386100

O 1.223890 7.474000 1.760420

O 3.466830 10.463600 2.799080

O 3.663830 7.474000 3.191290

O 1.257330 6.146160 4.002710

O 1.257330 8.801840 4.002710

O 3.642697 11.772083 5.726300

O 3.634913 9.141413 5.724441

O 1.280035 10.461399 6.521958

O 1.079984 7.471837 6.897410

O 3.610791 10.456262 7.983758

O 3.457456 7.441548 8.284750

O 1.037895 6.109686 9.130955

O 1.046786 8.820676 9.142508

Mg 2.543480 13.453200 0.388370

Mg 0.008060 17.893540 2.261970

Mg 0.008060 14.992060 2.261970

Mg 2.350078 16.438069 4.519344

Mg 4.738897 13.426991 5.199641

Mg 2.397049 11.997030 7.457638

Mg 2.385247 14.891863 7.435393

Mg 0.182685 16.437565 9.279684

Si 2.727970 16.442800 1.221250

Si 2.018610 13.453200 3.313620

Si 4.388123 16.429494 6.393814

Si 0.356789 13.446683 8.467061

O 3.434710 17.805520 0.556660

O 3.434710 15.080070 0.556660

O 1.102310 16.442800 1.386100

O 1.223890 13.453200 1.760420

O 3.466830 16.442800 2.799080

O 3.663830 13.453200 3.191290

O 1.257330 12.125360 4.002710

O 1.257330 14.781030 4.002710

O 3.636771 17.751081 5.695583

O 3.641671 15.105926 5.701982

O 1.280040 16.438180 6.506242

O 1.092420 13.443197 6.901753

O 3.591049 16.438630 7.936199

O 3.493927 13.459563 8.297167

O 1.056261 12.094540 9.145783

O 1.056078 14.806315 9.135687

Mg 7.292480 1.494800 0.388370

Mg 4.757060 5.935140 2.261970

Mg 4.757060 3.033660 2.261970

Mg 7.088621 4.485138 4.517654

Mg 9.465055 1.487579 5.176007

Mg 7.129385 0.024027 7.450063

Mg 7.123799 2.923827 7.433348

Mg 4.906120 4.461001 9.286680

Si 7.476970 4.484400 1.221250

Si 6.767610 1.494800 3.313620

Si 9.122901 4.484096 6.404182

Si 5.101637 1.479498 8.467394

O 8.183700 5.847120 0.556660

O 8.183700 3.121670 0.556660

O 5.851310 4.484400 1.386100

O 5.972890 1.494800 1.760420

O 8.215830 4.484400 2.799080

O 8.412830 1.494800 3.191290

O 6.006330 0.166960 4.002710

O 6.006330 2.822640 4.002710

O 8.388054 5.815388 5.717051

O 8.388129 3.164640 5.693713

O 6.023497 4.460545 6.501164

O 5.841168 1.473997 6.900846

O 8.326362 4.461891 7.942678

O 8.237517 1.477629 8.303233

O 5.802646 0.125858 9.143403

O 5.802312 2.838453 9.134442

Mg 7.292480 7.474000 0.388370

Mg 4.757060 11.914340 2.261970

Mg 4.757060 9.012860 2.261970

Mg 7.124581 10.462635 4.569152

Mg 9.429576 7.501461 5.185017

Mg 7.094393 5.993524 7.438731

Mg 7.150101 8.918251 7.488600

Mg 4.963456 10.453553 9.368288

Si 7.476970 10.463600 1.221250

Si 6.767610 7.474000 3.313620

Si 9.150337 10.429643 6.489967

Si 5.076898 7.447767 8.439597

O 8.183700 11.826320 0.556660

O 8.183700 9.100870 0.556660

O 5.851310 10.463600 1.386100

O 5.972890 7.474000 1.760420

O 8.215830 10.463600 2.799080

O 8.412830 7.474000 3.191290

O 6.006330 6.146160 4.002710

O 6.006330 8.801840 4.002710

O 8.410878 11.762614 5.745005

O 8.393453 9.131512 5.703710

O 6.038984 10.449159 6.538702

O 5.838911 7.453546 6.889592

O 8.421863 10.391399 7.981449

O 8.153288 7.390883 8.385647

O 5.773081 6.092029 9.129244

O 5.759075 8.792891 9.116037

Mg 7.292480 13.453200 0.388370

| | | | |
|---|---|---|---|
| Mg | 4.757060 | 17.893540 | 2.261970 |
| Mg | 4.757060 | 14.992060 | 2.261970 |
| Mg | 7.090648 | 16.436072 | 4.521850 |
| Mg | 9.445802 | 13.409218 | 5.179331 |
| Mg | 7.186550 | 11.938952 | 7.535216 |
| Mg | 7.132379 | 14.878369 | 7.443118 |
| Mg | 4.926717 | 16.428591 | 9.285496 |
| Si | 7.476970 | 16.442800 | 1.221250 |
| Si | 6.767610 | 13.453200 | 3.313620 |
| Si | 9.133199 | 16.423611 | 6.395281 |
| Si | 5.116396 | 13.439760 | 8.460767 |
| O | 8.183700 | 17.805520 | 0.556660 |
| O | 8.183700 | 15.080070 | 0.556660 |
| O | 5.851310 | 16.442800 | 1.386100 |
| O | 5.972890 | 13.453200 | 1.760420 |
| O | 8.215830 | 16.442800 | 2.799080 |
| O | 8.412830 | 13.453200 | 3.191290 |
| O | 6.006330 | 12.125360 | 4.002710 |
| O | 6.006330 | 14.781030 | 4.002710 |
| O | 8.388530 | 17.748908 | 5.704117 |
| O | 8.389094 | 15.097878 | 5.707751 |
| O | 6.031899 | 16.429808 | 6.514862 |
| O | 5.878850 | 13.412425 | 6.909748 |
| O | 8.351505 | 16.419720 | 7.944300 |
| O | 8.238841 | 13.458246 | 8.369717 |
| O | 5.801070 | 12.101757 | 9.160322 |
| O | 5.812663 | 14.807701 | 9.124146 |

| | | | |
|---|---|---|---|
| Mg | 12.041480 | 1.494800 | 0.388370 |
| Mg | 9.506060 | 5.935140 | 2.261970 |
| Mg | 9.506060 | 3.033660 | 2.261970 |
| Mg | 11.839521 | 4.488761 | 4.523601 |
| Mg | 14.211330 | 1.511526 | 5.179338 |
| Mg | 11.884163 | 0.039803 | 7.432537 |
| Mg | 11.872359 | 2.950436 | 7.449174 |
| Mg | 9.652168 | 4.439406 | 9.297113 |
| Si | 12.225970 | 4.484400 | 1.221250 |
| Si | 11.516610 | 1.494800 | 3.313620 |
| Si | 13.858221 | 4.518381 | 6.415631 |
| Si | 9.857333 | 1.472476 | 8.465553 |
| O | 12.932710 | 5.847120 | 0.556660 |
| O | 12.932710 | 3.121670 | 0.556660 |
| O | 10.600310 | 4.484400 | 1.386100 |
| O | 10.721890 | 1.494800 | 1.760420 |
| O | 12.964830 | 4.484400 | 2.799080 |
| O | 13.161830 | 1.494800 | 3.191290 |
| O | 10.755330 | 0.166960 | 4.002710 |
| O | 10.755330 | 2.822640 | 4.002710 |
| O | 13.097260 | 5.845693 | 5.737635 |
| O | 13.119421 | 3.193461 | 5.709954 |
| O | 10.763038 | 4.517431 | 6.547872 |
| O | 10.594471 | 1.492655 | 6.898336 |
| O | 13.070680 | 4.503578 | 7.964286 |
| O | 12.975807 | 1.496217 | 8.308704 |
| O | 10.561720 | 0.111544 | 9.126864 |

O 10.554867 2.825183 9.148687

Mg 12.041480 7.474000 0.388370

Mg 9.506060 11.914340 2.261970

Mg 9.506060 9.012860 2.261970

Mg 11.823097 10.473164 4.511564

Mg 14.228231 7.465276 5.160501

Mg 11.826535 6.046142 7.524527

Mg 11.941619 8.928821 7.271382

Si 12.225475 10.459190 1.229210

Si 11.516610 7.474000 3.313620

Si 13.888210 10.463600 6.382630

Si 9.745507 7.464734 8.664803

O 12.928036 11.816420 0.557568

O 12.932710 9.100870 0.556660

O 10.600310 10.463600 1.386100

O 10.721890 7.474000 1.760420

O 12.964840 10.463600 2.799080

O 13.161830 7.474000 3.191290

O 10.755330 6.146160 4.002710

O 10.755330 8.801840 4.002710

O 13.128110 11.791550 5.692940

O 13.160112 9.142256 5.647519

O 10.816004 10.438849 6.450867

O 10.524278 7.612409 7.131691

O 13.093374 10.434693 7.896312

O 12.951548 7.514721 8.337842

O 10.539410 6.078058 9.204817

Mg 12.055880 13.451052 0.411227

Mg 9.504437 17.892787 2.256588

Mg 9.506060 14.992060 2.261970

Mg 11.837740 16.442800 4.520030

Mg 14.211300 13.453200 5.174280

Mg 11.886402 11.959383 7.381692

Mg 11.898810 14.880703 7.434924

Mg 9.699229 16.408190 9.281572

Si 12.223678 16.442075 1.231041

Si 11.510898 13.454255 3.310514

Si 13.888220 16.442800 6.382630

Si 9.847270 13.453200 8.472360

O 12.921330 17.801148 0.558679

O 12.927601 15.086210 0.560164

O 10.600310 16.442800 1.386100

O 10.721890 13.453200 1.760420

O 12.964840 16.442800 2.799080

O 13.161830 13.453200 3.191290

O 10.755330 12.125360 4.002710

O 10.755330 14.781030 4.002710

O 13.128120 17.770750 5.692940

O 13.128120 15.114850 5.692940

O 10.775646 16.418048 6.532441

O 10.586696 13.418840 6.936051

O 13.095451 16.434272 7.931011

O 12.970129 13.425639 8.303973

O 10.576666 12.009801 9.064042

| | | | |
|---|---|---|---|
| O | 10.574037 | 14.755156 | 9.207923 |
| Mg | 16.804067 | 1.494256 | 0.409997 |
| Mg | 14.254260 | 5.934329 | 2.252342 |
| Mg | 14.255060 | 3.033660 | 2.261970 |
| Mg | 16.586740 | 4.484400 | 4.520030 |
| Mg | 18.960290 | 1.494800 | 5.174280 |
| Mg | 16.631150 | 0.048728 | 7.434280 |
| Mg | 16.624526 | 2.945687 | 7.432572 |
| Mg | 14.426417 | 4.476909 | 9.294218 |
| Si | 16.973516 | 4.484537 | 1.231996 |
| Si | 16.261200 | 1.492668 | 3.310227 |
| Si | 18.637210 | 4.484400 | 6.382630 |
| Si | 14.596270 | 1.494800 | 8.472360 |
| O | 17.673856 | 5.841302 | 0.559875 |
| O | 17.674590 | 3.128122 | 0.559727 |
| O | 15.349310 | 4.484400 | 1.386100 |
| O | 15.470890 | 1.494800 | 1.760420 |
| O | 17.713830 | 4.484400 | 2.799080 |
| O | 17.910830 | 1.494800 | 3.191290 |
| O | 15.504330 | 0.166960 | 4.002710 |
| O | 15.504330 | 2.822640 | 4.002710 |
| O | 17.877110 | 5.812340 | 5.692940 |
| O | 17.877110 | 3.156450 | 5.692940 |
| O | 15.502983 | 4.499894 | 6.525493 |
| O | 15.330424 | 1.497298 | 6.902819 |
| O | 17.837673 | 4.484315 | 7.925715 |
| O | 17.729452 | 1.498197 | 8.300229 |
| O | 15.301856 | 0.140473 | 9.140066 |
| O | 15.301881 | 2.849287 | 9.142144 |
| Mg | 16.803426 | 7.475323 | 0.408531 |
| Mg | 14.254939 | 11.911775 | 2.258250 |
| Mg | 14.255060 | 9.012860 | 2.261970 |
| Mg | 16.586740 | 10.463600 | 4.520030 |
| Mg | 18.960300 | 7.474000 | 5.174280 |
| Mg | 16.629301 | 6.028862 | 7.433002 |
| Mg | 16.617500 | 8.934443 | 7.443942 |
| Mg | 14.333219 | 10.462703 | 9.335927 |
| Si | 16.973712 | 10.463969 | 1.232297 |
| Si | 16.262774 | 7.476267 | 3.307795 |
| Si | 18.637210 | 10.463600 | 6.382630 |
| Si | 14.596270 | 7.474000 | 8.472360 |
| O | 17.674856 | 11.819344 | 0.558987 |
| O | 17.674620 | 9.108072 | 0.559139 |
| O | 15.349310 | 10.463600 | 1.386100 |
| O | 15.470890 | 7.474000 | 1.760420 |
| O | 17.713840 | 10.463600 | 2.799080 |
| O | 17.910830 | 7.474000 | 3.191290 |
| O | 15.504330 | 6.146160 | 4.002710 |
| O | 15.504330 | 8.801840 | 4.002710 |
| O | 17.877120 | 11.791540 | 5.692940 |
| O | 17.877110 | 9.135650 | 5.692940 |
| O | 15.529363 | 10.464466 | 6.489501 |
| O | 15.318990 | 7.472121 | 6.906961 |
| O | 17.841403 | 10.463727 | 7.929943 |

O 17.717125 7.476961 8.303254

O 15.282386 6.119421 9.149437

O 15.267146 8.846872 9.137863

Mg 16.804886 13.453279 0.411255

Mg 14.253936 17.892029 2.255975

Mg 14.255060 14.992060 2.261970

Mg 16.586740 16.442800 4.520030

Mg 18.960300 13.453200 5.174280

Mg 16.619562 11.995490 7.445221

Mg 16.636265 14.895337 7.436457

Mg 14.432239 16.444150 9.281140

Si 16.973568 16.442728 1.232079

Si 16.261388 13.452124 3.310606

Si 18.637220 16.442800 6.382630

Si 14.596270 13.453200 8.472360

O 17.675130 17.798500 0.559409

O 17.674965 15.087470 0.559244

O 15.349310 16.442800 1.386100

O 15.470890 13.453200 1.760420

O 17.713840 16.442800 2.799080

O 17.910830 13.453200 3.191290

O 15.504330 12.125360 4.002710

O 15.504330 14.781030 4.002710

O 17.877120 17.770750 5.692940

O 17.877120 15.114850 5.692940

O 15.527324 16.441676 6.511452

O 15.333068 13.457429 6.906168

O 17.842087 16.442616 7.929332

O 17.729264 13.447166 8.309722

O 15.267762 12.075518 9.132956

O 15.294696 14.805039 9.150010

C 11.444692 9.190287 10.468030

C 11.674899 10.525392 10.630561

C 10.579333 11.555835 10.452805

C 9.236634 10.963664 10.850467

C 8.251525 11.802467 11.412714

C 7.026961 11.274134 11.869345

C 6.784867 9.889578 11.748380

C 7.751993 9.050083 11.152099

C 8.986082 9.570247 10.701687

C 10.075877 8.622405 10.245813

H 12.261545 8.464634 10.572126

H 12.653939 10.890657 10.996089

H 10.789482 12.444190 11.081167

H 8.449355 12.875829 11.517354

H 6.290585 11.932577 12.342419

H 5.871026 9.444227 12.164177

H 7.539810 7.981040 11.034951

H 10.034457 7.739219 10.922272

**Naphthalene V$_{MgO}$-[010]-fo (⊥)**

Number of atoms: 352

Mg 2.543480 1.494800 0.388370

Mg 0.008060 5.935140 2.261970

| | | | |
|---|---|---|---|
| Mg | 0.008060 | 3.033660 | 2.261970 |
| Mg | 2.344229 | 4.487631 | 4.520166 |
| Mg | 4.713290 | 1.493653 | 5.178206 |
| Mg | 2.373422 | 0.044207 | 7.439404 |
| Mg | 2.375788 | 2.946767 | 7.434616 |
| Mg | 0.177534 | 4.485983 | 9.282658 |
| Si | 2.727970 | 4.484400 | 1.221250 |
| Si | 2.018610 | 1.494800 | 3.313620 |
| Si | 4.380509 | 4.489341 | 6.393692 |
| Si | 0.350380 | 1.497272 | 8.463417 |
| O | 3.434710 | 5.847120 | 0.556660 |
| O | 3.434700 | 3.121670 | 0.556660 |
| O | 1.102310 | 4.484400 | 1.386100 |
| O | 1.223890 | 1.494800 | 1.760420 |
| O | 3.466830 | 4.484400 | 2.799080 |
| O | 3.663830 | 1.494800 | 3.191290 |
| O | 1.257330 | 0.166960 | 4.002710 |
| O | 1.257330 | 2.822640 | 4.002710 |
| O | 3.634745 | 5.814141 | 5.701908 |
| O | 3.629470 | 3.168676 | 5.695559 |
| O | 1.267689 | 4.491725 | 6.518388 |
| O | 1.088345 | 1.496975 | 6.897432 |
| O | 3.585961 | 4.485080 | 7.935880 |
| O | 3.476278 | 1.495862 | 8.303345 |
| O | 1.047476 | 0.140819 | 9.136224 |
| O | 1.050007 | 2.853108 | 9.134334 |
| Mg | 2.543480 | 7.474000 | 0.388370 |
| Mg | 0.008060 | 11.914340 | 2.261970 |
| Mg | 0.008060 | 9.012860 | 2.261970 |
| Mg | 2.352089 | 10.464386 | 4.530560 |
| Mg | 4.731411 | 7.486155 | 5.196096 |
| Mg | 2.381655 | 6.034277 | 7.443901 |
| Mg | 2.382053 | 8.929128 | 7.450564 |
| Mg | 0.179669 | 10.463454 | 9.302605 |
| Si | 2.727970 | 10.463600 | 1.221250 |
| Si | 2.018610 | 7.474000 | 3.313620 |
| Si | 4.395201 | 10.465958 | 6.413913 |
| Si | 0.347829 | 7.480506 | 8.474623 |
| O | 3.434710 | 11.826320 | 0.556660 |
| O | 3.434710 | 9.100870 | 0.556660 |
| O | 1.102310 | 10.463600 | 1.386100 |
| O | 1.223890 | 7.474000 | 1.760420 |
| O | 3.466830 | 10.463600 | 2.799080 |
| O | 3.663830 | 7.474000 | 3.191290 |
| O | 1.257330 | 6.146160 | 4.002710 |
| O | 1.257330 | 8.801840 | 4.002710 |
| O | 3.637783 | 11.782469 | 5.710998 |
| O | 3.639597 | 9.147773 | 5.712907 |
| O | 1.275911 | 10.468182 | 6.527989 |
| O | 1.086915 | 7.479995 | 6.908888 |
| O | 3.596800 | 10.467505 | 7.955911 |
| O | 3.490755 | 7.483223 | 8.307062 |
| O | 1.047398 | 6.120264 | 9.139690 |
| O | 1.053682 | 8.830678 | 9.150338 |

Mg 2.543480 13.453200 0.388370
Mg 0.008060 17.893540 2.261970
Mg 0.008060 14.992060 2.261970
Mg 2.342359 16.439949 4.522261
Mg 4.723355 13.442432 5.189697
Mg 2.383062 12.004187 7.456904
Mg 2.379823 14.895825 7.455848
Mg 0.175628 16.444157 9.286383
Si 2.727970 16.442800 1.221250
Si 2.018610 13.453200 3.313620
Si 4.375329 16.438901 6.397032
Si 0.346444 13.448272 8.485100
O 3.434710 17.805520 0.556660
O 3.434710 15.080070 0.556660
O 1.102310 16.442800 1.386100
O 1.223890 13.453200 1.760420
O 3.466830 16.442800 2.799080
O 3.663830 13.453200 3.191290
O 1.257330 12.125360 4.002710
O 1.257330 14.781030 4.002710
O 3.625347 17.759856 5.699678
O 3.630096 15.113007 5.706774
O 1.265982 16.435224 6.526036
O 1.086232 13.450287 6.918796
O 3.586244 16.443631 7.942609
O 3.489567 13.449559 8.324544
O 1.053448 12.096568 9.158491
O 1.046030 14.808717 9.151600
Mg 7.292480 1.494800 0.388370
Mg 4.757060 5.935140 2.261970
Mg 4.757060 3.033660 2.261970
Mg 7.090995 4.486520 4.520875
Mg 9.465104 1.496045 5.178282
Mg 7.126871 0.040362 7.445689
Mg 7.122207 2.939766 7.436727
Mg 4.915186 4.482116 9.292241
Si 7.476970 4.484400 1.221250
Si 6.767610 1.494800 3.313620
Si 9.124001 4.489569 6.406137
Si 5.097159 1.494530 8.467161
O 8.183700 5.847120 0.556660
O 8.183700 3.121670 0.556660
O 5.851310 4.484400 1.386100
O 5.972890 1.494800 1.760420
O 8.215830 4.484400 2.799080
O 8.412830 1.494800 3.191290
O 6.006330 0.166960 4.002710
O 6.006330 2.822640 4.002710
O 8.385362 5.821882 5.721588
O 8.381114 3.173864 5.695943
O 6.023948 4.485878 6.512448
O 5.835743 1.491508 6.900550
O 8.333918 4.473884 7.948815
O 8.231346 1.490476 8.303771

O 5.799280 0.139660 9.139841

O 5.798137 2.852482 9.135243

Mg 7.292480 7.474000 0.388370

Mg 4.757060 11.914340 2.261970

Mg 4.757060 9.012860 2.261970

Mg 7.111714 10.462246 4.540052

Mg 9.445918 7.494039 5.201310

Mg 7.115269 6.019417 7.453435

Mg 7.187397 8.940041 7.492829

Mg 4.935058 10.464369 9.306372

Si 7.476970 10.463600 1.221250

Si 6.767610 7.474000 3.313620

Si 9.157018 10.455564 6.414774

Si 5.113351 7.484270 8.480610

O 8.183700 11.826320 0.556660

O 8.183700 9.100870 0.556660

O 5.851310 10.463600 1.386100

O 5.972890 7.474000 1.760420

O 8.215830 10.463600 2.799080

O 8.412830 7.474000 3.191290

O 6.006330 6.146160 4.002710

O 6.006330 8.801840 4.002710

O 8.396881 11.772376 5.686941

O 8.381702 9.136709 5.711261

O 6.036491 10.460429 6.554455

O 5.860215 7.497076 6.920410

O 8.471109 10.478163 7.951999

O 8.194099 7.425940 8.386172

O 5.790233 6.111435 9.143943

O 5.815530 8.830015 9.177583

Mg 7.292480 13.453200 0.388370

Mg 4.757060 17.893540 2.261970

Mg 4.757060 14.992060 2.261970

Mg 7.091242 16.443830 4.517766

Mg 9.441169 13.445706 5.149978

Mg 7.166641 11.974477 7.468971

Mg 7.105634 14.904760 7.416828

Mg 4.926308 16.442338 9.289385

Si 7.476970 16.442800 1.221250

Si 6.767610 13.453200 3.313620

Si 9.127652 16.440787 6.387904

Si 5.113426 13.443228 8.485146

O 8.183700 17.805520 0.556660

O 8.183700 15.080070 0.556660

O 5.851310 16.442800 1.386100

O 5.972890 13.453200 1.760420

O 8.215830 16.442800 2.799080

O 8.412830 13.453200 3.191290

O 6.006330 12.125360 4.002710

O 6.006330 14.781030 4.002710

O 8.378589 17.767829 5.702060

O 8.371566 15.126402 5.685666

O 6.018861 16.443128 6.513587

O 5.841286 13.420472 6.915942

O 8.343028 16.429857 7.936001

O 8.211926 13.481981 8.276759

O 5.817688 12.101335 9.189514

O 5.807309 14.815771 9.132800

Mg 12.041480 1.494800 0.388370

Mg 9.506060 5.935140 2.261970

Mg 9.506060 3.033660 2.261970

Mg 11.840996 4.486129 4.521116

Mg 14.211215 1.508709 5.179706

Mg 11.876893 0.043717 7.434224

Mg 11.872655 2.949590 7.451809

Mg 9.652797 4.431991 9.315035

Si 12.225970 4.484400 1.221250

Si 11.516610 1.494800 3.313620

Si 13.862934 4.512655 6.408259

Si 9.851224 1.472475 8.468259

O 12.932710 5.847120 0.556660

O 12.932710 3.121670 0.556660

O 10.600310 4.484400 1.386100

O 10.721890 1.494800 1.760420

O 12.964830 4.484400 2.799080

O 13.161830 1.494800 3.191290

O 10.755330 0.166960 4.002710

O 10.755330 2.822640 4.002710

O 13.106512 5.837550 5.720681

O 13.122947 3.188051 5.705322

O 10.765251 4.506805 6.528202

O 10.589656 1.497895 6.901665

O 13.072667 4.501587 7.953440

O 12.975909 1.495862 8.307181

O 10.546323 0.104808 9.124273

O 10.553879 2.819037 9.155979

Mg 12.041480 7.474000 0.388370

Mg 9.506060 11.914340 2.261970

Mg 9.506060 9.012860 2.261970

Mg 11.841261 10.476012 4.496593

Mg 14.233309 7.470552 5.161296

Mg 11.831179 6.034110 7.481787

Mg 11.935414 8.932036 7.234572

Si 12.225470 10.459190 1.229210

Si 11.516610 7.474000 3.313620

Si 13.913023 10.486721 6.358876

Si 9.795963 7.440936 8.661387

O 12.928040 11.816420 0.557570

O 12.932710 9.100870 0.556660

O 10.600310 10.463600 1.386100

O 10.721890 7.474000 1.760420

O 12.964840 10.463600 2.799080

O 13.161830 7.474000 3.191290

O 10.755330 6.146160 4.002710

O 10.755330 8.801840 4.002710

O 13.148990 11.798472 5.660292

O 13.177306 9.159184 5.629134

O 10.813205 10.445516 6.403393

O 10.532461 7.581438 7.089085

O 13.073240 10.453286 7.867511

O 12.948167 7.510413 8.268881

O 10.569672 6.048313 9.184745

Mg 12.055880 13.451050 0.411230

Mg 9.504440 17.892790 2.256590

Mg 9.506060 14.992060 2.261970

Mg 11.840350 16.442270 4.518844

Mg 14.224136 13.463713 5.173393

Mg 11.854146 11.979598 7.344408

Mg 11.888713 14.896345 7.412227

Mg 9.690184 16.396039 9.278303

Si 12.223680 16.442080 1.231040

Si 11.510900 13.454260 3.310510

Si 13.881652 16.448980 6.393266

Si 9.822505 13.473608 8.387168

O 12.921330 17.801150 0.558680

O 12.927600 15.086210 0.560160

O 10.600310 16.442800 1.386100

O 10.721890 13.453200 1.760420

O 12.964840 16.442800 2.799080

O 13.161830 13.453200 3.191290

O 10.755330 12.125360 4.002710

O 10.755330 14.781030 4.002710

O 13.130306 17.770493 5.700218

O 13.134157 15.128351 5.692981

O 10.769804 16.440941 6.512158

O 10.582935 13.481173 6.846151

O 13.084790 16.438538 7.936052

O 12.930412 13.416431 8.283299

O 10.444230 11.988717 8.951088

O 10.567459 14.751218 9.135148

Mg 16.804070 1.494260 0.410000

Mg 14.254260 5.934330 2.252340

Mg 14.255060 3.033660 2.261970

Mg 16.590213 4.486922 4.522381

Mg 18.960099 1.495876 5.178914

Mg 16.624039 0.046441 7.438651

Mg 16.620561 2.951302 7.438136

Mg 14.426903 4.491789 9.285420

Si 16.973520 4.484540 1.232000

Si 16.261200 1.492670 3.310230

Si 18.622899 4.495766 6.396603

Si 14.597225 1.496910 8.468332

O 17.673860 5.841300 0.559870

O 17.674590 3.128120 0.559730

O 15.349310 4.484400 1.386100

O 15.470890 1.494800 1.760420

O 17.713830 4.484400 2.799080

O 17.910830 1.494800 3.191290

O 15.504330 0.166960 4.002710

O 15.504330 2.822640 4.002710

O 17.871603 5.817183 5.702860

O 17.871942 3.174200 5.699409

O 15.506278 4.505310 6.527823

O 15.332908 1.500269 6.901028

O 17.827120 4.495241 7.941506

O 17.725998 1.499627 8.298406

O 15.296117 0.136987 9.135600

O 15.296992 2.856066 9.137803

Mg 16.803430 7.475320 0.408530

Mg 14.254940 11.911770 2.258250

Mg 14.255060 9.012860 2.261970

Mg 16.597666 10.466810 4.530514

Mg 18.964982 7.484924 5.183950

Mg 16.621333 6.043549 7.444014

Mg 16.610838 8.950538 7.464047

Mg 14.219703 10.457159 9.390147

Si 16.973710 10.463970 1.232300

Si 16.262770 7.476270 3.307800

Si 18.629385 10.471750 6.405819

Si 14.581977 7.504666 8.456416

O 17.674860 11.819340 0.558990

O 17.674620 9.108070 0.559140

O 15.349310 10.463600 1.386100

O 15.470890 7.474000 1.760420

O 17.713840 10.463600 2.799080

O 17.910830 7.474000 3.191290

O 15.504330 6.146160 4.002710

O 15.504330 8.801840 4.002710

O 17.880837 11.793141 5.711879

O 17.881384 9.148298 5.711998

O 15.548637 10.473185 6.478656

O 15.330464 7.493007 6.903030

O 17.839951 10.469596 7.953000

O 17.719267 7.488665 8.320654

O 15.262826 6.146879 9.141478

O 15.225632 8.879431 9.131704

Mg 16.804890 13.453280 0.411260

Mg 14.253940 17.892030 2.255970

Mg 14.255060 14.992060 2.261970

Mg 16.592826 16.440625 4.524252

Mg 18.966516 13.448845 5.187774

Mg 16.603067 11.988890 7.476605

Mg 16.610481 14.884813 7.458606

Mg 14.423958 16.437190 9.284637

Si 16.973570 16.442730 1.232080

Si 16.261390 13.452120 3.310610

Si 18.620597 16.431654 6.402004

Si 14.552756 13.421632 8.474203

O 17.675130 17.798500 0.559410

O 17.674970 15.087470 0.559240

O 15.349310 16.442800 1.386100

O 15.470890 13.453200 1.760420

O 17.713840 16.442800 2.799080

O 17.910830 13.453200 3.191290

O 15.504330 12.125360 4.002710

O 15.504330 14.781030 4.002710

O 17.877034 17.757498 5.703911

O 17.870790 15.110167 5.707966

O 15.521930 16.438446 6.523080

O 15.318847 13.438940 6.918666

O 17.824639 16.434598 7.946065

O 17.717914 13.442210 8.333754

O 15.209698 12.045340 9.141609

O 15.252031 14.778445 9.153860

C 11.590628 8.910956 10.497097

C 10.266338 8.763175 9.944081

C 9.261130 9.600498 10.454827

C 9.563001 10.721041 11.296830

C 8.563964 11.700995 11.601318

C 8.905595 12.861987 12.294413

C 10.246070 13.070117 12.733633

C 11.238402 12.129355 12.454204

C 10.929889 10.951450 11.710154

C 11.918971 9.969984 11.351350

H 12.334894 8.147410 10.246121

H 9.726907 11.301203 8.798919

H 8.216182 9.428013 10.166798

H 7.544017 11.577320 11.212979

H 8.139768 13.619229 12.496596

H 10.498601 13.980046 13.290032

H 12.270867 12.303309 12.780469

H 12.901716 9.999342 11.852628

**Anthracene [010]-fo**

Number of atoms: 360

Mg 2.543480 1.494800 0.388370

Mg 0.008060 5.935140 2.261970

Mg 0.008060 3.033660 2.261970

Mg 2.345815 4.485860 4.518035

Mg 4.719654 1.497840 5.178488

Mg 2.387496 0.051084 7.435790

Mg 2.385400 2.951343 7.437139

Mg 0.180931 4.481683 9.277662

Si 2.727970 4.484400 1.221250

Si 2.018610 1.494800 3.313620

Si 4.383259 4.494984 6.388603

Si 0.362062 1.498198 8.464103

O 3.434710 5.847120 0.556660

O 3.434700 3.121670 0.556660

O 1.102310 4.484400 1.386100

O 1.223890 1.494800 1.760420

O 3.466830 4.484400 2.799080

O 3.663830 1.494800 3.191290

O 1.257330 0.166960 4.002710

O 1.257330 2.822640 4.002710

O 3.628852 5.813228 5.693595

O 3.635011 3.171312 5.695244

O 1.274012 4.492538 6.517216

O 1.097247 1.500980 6.897526

O 3.592493 4.494778 7.934167

O 3.486738 1.503398 8.303401

O 1.064302 0.144550 9.137119

O 1.061445 2.853617 9.137711

Mg 2.543480 7.474000 0.388370

Mg 0.008060 11.914340 2.261970

Mg 0.008060 9.012860 2.261970

Mg 2.358689 10.464882 4.526390

Mg 4.720647 7.483482 5.178301

Mg 2.381713 6.033398 7.439290

Mg 2.394471 8.929941 7.442425

Mg 0.167697 10.465363 9.266009

Si 2.727970 10.463600 1.221250

Si 2.018610 7.474000 3.313620

Si 4.396712 10.470064 6.412340

Si 0.347957 7.471067 8.475818

O 3.434710 11.826320 0.556660

O 3.434710 9.100870 0.556660

O 1.102310 10.463600 1.386100

O 1.223890 7.474000 1.760420

O 3.466830 10.463600 2.799080

O 3.663830 7.474000 3.191290

O 1.257330 6.146160 4.002710

O 1.257330 8.801840 4.002710

O 3.633790 11.786863 5.706342

O 3.636003 9.148849 5.711030

O 1.268036 10.466294 6.541015

O 1.089532 7.489912 6.915132

O 3.599085 10.471778 7.946699

O 3.477601 7.475812 8.313817

O 1.051615 6.117220 9.148092

O 1.038660 8.817409 9.193601

Mg 2.543480 13.453200 0.388370

Mg 0.008060 17.893540 2.261970

Mg 0.008060 14.992060 2.261970

Mg 2.346657 16.442438 4.519946

Mg 4.722325 13.449666 5.178359

Mg 2.396254 12.007740 7.442522

Mg 2.383784 14.903374 7.443390

Mg 0.189844 16.450106 9.283008

Si 2.727970 16.442800 1.221250

Si 2.018610 13.453200 3.313620

Si 4.384641 16.442833 6.390535

Si 0.362488 13.453414 8.495250

O 3.434710 17.805520 0.556660

O 3.434710 15.080070 0.556660

O 1.102310 16.442800 1.386100

O 1.223890 13.453200 1.760420

O 3.466830 16.442800 2.799080

O 3.663830 13.453200 3.191290

O 1.257330 12.125360 4.002710

O 1.257330 14.781030 4.002710

O 3.633422 17.765515 5.696285

O 3.631206 15.121779 5.698069

O 1.276088 16.441500 6.519483

| | | | |
|---|---|---|---|
| O | 1.091501 | 13.444290 | 6.925141 |
| O | 3.592861 | 16.447051 | 7.936283 |
| O | 3.485678 | 13.462489 | 8.309733 |
| O | 1.061063 | 12.100349 | 9.195383 |
| O | 1.060927 | 14.814028 | 9.158581 |
| Mg | 7.292480 | 1.494800 | 0.388370 |
| Mg | 4.757060 | 5.935140 | 2.261970 |
| Mg | 4.757060 | 3.033660 | 2.261970 |
| Mg | 7.094710 | 4.488073 | 4.519453 |
| Mg | 9.467296 | 1.497168 | 5.177027 |
| Mg | 7.135072 | 0.051188 | 7.438158 |
| Mg | 7.135937 | 2.948070 | 7.439084 |
| Mg | 4.949846 | 4.504522 | 9.258940 |
| Si | 7.476970 | 4.484400 | 1.221250 |
| Si | 6.767610 | 1.494800 | 3.313620 |
| Si | 9.131173 | 4.492080 | 6.388167 |
| Si | 5.107567 | 1.504058 | 8.468092 |
| O | 8.183700 | 5.847120 | 0.556660 |
| O | 8.183700 | 3.121670 | 0.556660 |
| O | 5.851310 | 4.484400 | 1.386100 |
| O | 5.972890 | 1.494800 | 1.760420 |
| O | 8.215830 | 4.484400 | 2.799080 |
| O | 8.412830 | 1.494800 | 3.191290 |
| O | 6.006330 | 0.166960 | 4.002710 |
| O | 6.006330 | 2.822640 | 4.002710 |
| O | 8.379293 | 5.810584 | 5.694515 |
| O | 8.380936 | 3.169453 | 5.695121 |

| | | | |
|---|---|---|---|
| O | 6.023885 | 4.492762 | 6.530795 |
| O | 5.844095 | 1.501582 | 6.901152 |
| O | 8.342648 | 4.492149 | 7.936605 |
| O | 8.239992 | 1.500785 | 8.304782 |
| O | 5.808287 | 0.149503 | 9.141890 |
| O | 5.809736 | 2.861405 | 9.138296 |
| Mg | 7.292480 | 7.474000 | 0.388370 |
| Mg | 4.757060 | 11.914340 | 2.261970 |
| Mg | 4.757060 | 9.012860 | 2.261970 |
| Mg | 7.100830 | 10.466812 | 4.538562 |
| Mg | 9.465003 | 7.485908 | 5.179963 |
| Mg | 7.138064 | 6.038760 | 7.444486 |
| Mg | 7.125599 | 8.943037 | 7.448480 |
| Mg | 4.852953 | 10.485846 | 9.404486 |
| Si | 7.476970 | 10.463600 | 1.221250 |
| Si | 6.767610 | 7.474000 | 3.313620 |
| Si | 9.143968 | 10.468233 | 6.426420 |
| Si | 5.102881 | 7.500237 | 8.460520 |
| O | 8.183700 | 11.826320 | 0.556660 |
| O | 8.183700 | 9.100870 | 0.556660 |
| O | 5.851310 | 10.463600 | 1.386100 |
| O | 5.972890 | 7.474000 | 1.760420 |
| O | 8.215830 | 10.463600 | 2.799080 |
| O | 8.412830 | 7.474000 | 3.191290 |
| O | 6.006330 | 6.146160 | 4.002710 |
| O | 6.006330 | 8.801840 | 4.002710 |
| O | 8.388371 | 11.787324 | 5.719190 |

O 8.387991 9.148140 5.719531
O 6.039019 10.471704 6.485783
O 5.839652 7.459458 6.897300
O 8.363087 10.469071 7.966443
O 8.231737 7.478703 8.315995
O 5.799974 6.159082 9.190491
O 5.795448 8.881911 9.069535
Mg 7.292480 13.453200 0.388370
Mg 4.757060 17.893540 2.261970
Mg 4.757060 14.992060 2.261970
Mg 7.094918 16.440683 4.521403
Mg 9.467832 13.445533 5.184196
Mg 7.131316 11.998111 7.455381
Mg 7.138613 14.901835 7.439772
Mg 4.942703 16.447735 9.270215
Si 7.476970 16.442800 1.221250
Si 6.767610 13.453200 3.313620
Si 9.134220 16.443479 6.390407
Si 5.110746 13.454366 8.455335
O 8.183700 17.805520 0.556660
O 8.183700 15.080070 0.556660
O 5.851310 16.442800 1.386100
O 5.972890 13.453200 1.760420
O 8.215830 16.442800 2.799080
O 8.412830 13.453200 3.191290
O 6.006330 12.125360 4.002710
O 6.006330 14.781030 4.002710
O 8.383050 17.766121 5.696398
O 8.383079 15.122267 5.697908
O 6.026162 16.447219 6.519929
O 5.846028 13.465457 6.890529
O 8.344968 16.447964 7.936935
O 8.234506 13.464108 8.319944
O 5.807479 12.094390 9.109740
O 5.807245 14.809778 9.141946
Mg 12.041480 1.494800 0.388370
Mg 9.506060 5.935140 2.261970
Mg 9.506060 3.033660 2.261970
Mg 11.841754 4.487305 4.518195
Mg 14.215544 1.495044 5.177832
Mg 11.882444 0.047177 7.435919
Mg 11.883064 2.945755 7.434394
Mg 9.700519 4.496103 9.261163
Si 12.225970 4.484400 1.221250
Si 11.516610 1.494800 3.313620
Si 13.881223 4.488478 6.389311
Si 9.860627 1.498989 8.466782
O 12.932710 5.847120 0.556660
O 12.932710 3.121670 0.556660
O 10.600310 4.484400 1.386100
O 10.721890 1.494800 1.760420
O 12.964830 4.484400 2.799080
O 13.161830 1.494800 3.191290
O 10.755330 0.166960 4.002710

O 10.755330 2.822640 4.002710

O 13.129954 5.808754 5.696583

O 13.130840 3.166284 5.696362

O 10.771485 4.487178 6.524416

O 10.594266 1.498606 6.898592

O 13.090353 4.488569 7.935222

O 12.984266 1.497696 8.304713

O 10.561785 0.143371 9.138440

O 10.563176 2.855552 9.137959

Mg 12.041480 7.474000 0.388370

Mg 9.506060 11.914340 2.261970

Mg 9.506060 9.012860 2.261970

Mg 11.832839 10.465440 4.527118

Mg 14.211233 7.479023 5.175901

Mg 11.883290 6.033261 7.440700

Mg 11.863866 8.936243 7.439427

Mg 9.618452 10.465794 9.431367

Si 12.225970 10.463600 1.221250

Si 11.516610 7.474000 3.313620

Si 13.878101 10.466940 6.389751

Si 9.853884 7.489895 8.462060

O 12.932710 11.826320 0.556660

O 12.932710 9.100870 0.556660

O 10.600310 10.463600 1.386100

O 10.721890 7.474000 1.760420

O 12.964840 10.463600 2.799080

O 13.161830 7.474000 3.191290

O 10.755330 6.146160 4.002710

O 10.755330 8.801840 4.002710

O 13.132194 11.790574 5.699659

O 13.131728 9.143088 5.696828

O 10.789362 10.467089 6.483315

O 10.590150 7.459218 6.895024

O 13.093981 10.464455 7.935323

O 12.978006 7.480696 8.315989

O 10.555854 6.142242 9.166953

O 10.553977 8.863619 9.083905

Mg 12.041480 13.453200 0.388370

Mg 9.506060 17.893540 2.261970

Mg 9.506060 14.992060 2.261970

Mg 11.842459 16.440409 4.521015

Mg 14.215112 13.451774 5.180748

Mg 11.863180 11.992177 7.450640

Mg 11.882219 14.896467 7.441866

Mg 9.695635 16.441329 9.269901

Si 12.225970 16.442800 1.221250

Si 11.516610 13.453200 3.313620

Si 13.883465 16.441500 6.393739

Si 9.856227 13.447529 8.471663

O 12.932710 17.805520 0.556660

O 12.932710 15.080070 0.556660

O 10.600310 16.442800 1.386100

O 10.721890 13.453200 1.760420

O 12.964840 16.442800 2.799080

O 13.161830 13.453200 3.191290

O 10.755330 12.125360 4.002710

O 10.755330 14.781030 4.002710

O 13.133078 17.764144 5.699667

O 13.132746 15.120843 5.699703

O 10.775185 16.444553 6.518019

O 10.591116 13.458458 6.902077

O 13.090958 16.441697 7.938490

O 12.982180 13.446578 8.310914

O 10.542181 12.083977 9.129207

O 10.563536 14.804912 9.146448

Mg 16.790480 1.494800 0.388370

Mg 14.255060 5.935140 2.261970

Mg 14.255060 3.033660 2.261970

Mg 16.591304 4.485352 4.518233

Mg 18.965897 1.495437 5.176388

Mg 16.630783 0.046307 7.440126

Mg 16.630302 2.943887 7.436074

Mg 14.434949 4.483789 9.274463

Si 16.974970 4.484400 1.221250

Si 16.265610 1.494800 3.313620

Si 18.629341 4.488373 6.389693

Si 14.604711 1.495787 8.468078

O 17.681710 5.847120 0.556660

O 17.681710 3.121670 0.556660

O 15.349310 4.484400 1.386100

O 15.470890 1.494800 1.760420

O 17.713830 4.484400 2.799080

O 17.910830 1.494800 3.191290

O 15.504330 0.166960 4.002710

O 15.504330 2.822640 4.002710

O 17.878821 5.810429 5.697310

O 17.880116 3.167125 5.694054

O 15.523087 4.485385 6.516572

O 15.340420 1.496096 6.901004

O 17.837150 4.487237 7.934558

O 17.737388 1.497178 8.302523

O 15.305870 0.140915 9.141352

O 15.308481 2.850679 9.139759

Mg 16.790480 7.474000 0.388370

Mg 14.255060 11.914340 2.261970

Mg 14.255060 9.012860 2.261970

Mg 16.587890 10.465042 4.518515

Mg 18.967188 7.477475 5.178045

Mg 16.624052 6.026450 7.435437

Mg 16.620415 8.922830 7.431253

Mg 14.435586 10.464112 9.271064

Si 16.974970 10.463600 1.221250

Si 16.265610 7.474000 3.313620

Si 18.626025 10.466348 6.388584

Si 14.598667 7.475694 8.471423

O 17.681710 11.826320 0.556660

O 17.681710 9.100870 0.556660

O 15.349310 10.463600 1.386100

O 15.470890 7.474000 1.760420
O 17.713840 10.463600 2.799080
O 17.910830 7.474000 3.191290
O 15.504330 6.146160 4.002710
O 15.504330 8.801840 4.002710
O 17.879445 11.790983 5.698635
O 17.879387 9.144116 5.693071
O 15.522681 10.466217 6.514582
O 15.332134 7.472890 6.902380
O 17.828838 10.462799 7.930974
O 17.726277 7.475294 8.301832
O 15.303458 6.119845 9.142025
O 15.306332 8.827853 9.144490
Mg 16.790480 13.453200 0.388370
Mg 14.255060 17.893540 2.261970
Mg 14.255060 14.992060 2.261970
Mg 16.591831 16.440612 4.521878
Mg 18.971204 13.452784 5.184517
Mg 16.627069 12.008175 7.438528
Mg 16.633876 14.899801 7.449404
Mg 14.432745 16.443574 9.280562
Si 16.974970 16.442800 1.221250
Si 16.265610 13.453200 3.313620
Si 18.631400 16.440998 6.394935
Si 14.601815 13.452457 8.471598
O 17.681710 17.805520 0.556660
O 17.681710 15.080070 0.556660
O 15.349310 16.442800 1.386100
O 15.470890 13.453200 1.760420
O 17.713840 16.442800 2.799080
O 17.910830 13.453200 3.191290
O 15.504330 12.125360 4.002710
O 15.504330 14.781030 4.002710
O 17.881216 17.762724 5.699820
O 17.882249 15.116332 5.705109
O 15.526076 16.441489 6.520387
O 15.341595 13.457271 6.905529
O 17.842472 16.442738 7.941923
O 17.738308 13.447452 8.322604
O 15.308872 12.098281 9.143592
O 15.304036 14.808581 9.145227
C 3.220342 10.605394 11.471450
C 3.093399 9.171125 11.530790
C 4.202514 8.388105 11.830123
C 5.510964 8.968876 11.982345
C 6.675289 8.167749 12.048308
C 7.964019 8.748351 12.034760
C 9.157670 7.946319 12.080956
C 10.418417 8.523681 12.032920
C 10.550573 9.951028 11.964102
C 9.423291 10.773434 11.907826
C 8.095385 10.198781 11.935850
C 6.934196 11.005681 11.868875
C 5.645182 10.417393 11.909004

C 4.456057 11.215319 11.706761

H 2.360801 11.241782 11.211701

H 2.136947 8.702855 11.270462

H 4.106368 7.297363 11.870185

H 6.575694 7.075884 12.027730

H 9.053349 6.857136 12.142402

H 11.313909 7.895275 12.021241

H 11.547247 10.406683 11.964851

H 9.540029 11.863920 11.873963

H 7.024752 12.087597 11.713474

H 4.554910 12.305206 11.658093

**Anthracene Fe-[010]-fo**

Number of atoms: 360

Mg 2.543480 1.494800 0.388370

Mg 0.008060 5.935140 2.261970

Mg 0.008060 3.033660 2.261970

Mg 2.341982 4.484795 4.517722

Mg 4.715929 1.493790 5.176780

Mg 2.378060 0.045901 7.436845

Mg 2.378586 2.944410 7.433647

Mg 0.181130 4.484507 9.278383

Si 2.727970 4.484400 1.221250

Si 2.018610 1.494800 3.313620

Si 4.381179 4.482600 6.385566

Si 0.355951 1.495728 8.466733

O 3.434710 5.847120 0.556660

O 3.434700 3.121670 0.556660

O 1.102310 4.484400 1.386100

O 1.223890 1.494800 1.760420

O 3.466830 4.484400 2.799080

O 3.663830 1.494800 3.191290

O 1.257330 0.166960 4.002710

O 1.257330 2.822640 4.002710

O 3.628704 5.802433 5.690627

O 3.628906 3.160605 5.693604

O 1.269272 4.485499 6.515453

O 1.090558 1.495370 6.899263

O 3.589048 4.483508 7.929171

O 3.482132 1.496118 8.304088

O 1.056531 0.140189 9.138780

O 1.056288 2.851934 9.137368

Mg 2.543480 7.474000 0.388370

Mg 0.008060 11.914340 2.261970

Mg 0.008060 9.012860 2.261970

Mg 2.349233 10.464054 4.533681

Mg 4.711509 7.476395 5.172139

Mg 2.373867 6.023753 7.431478

Mg 2.389545 8.926913 7.459258

Mg 0.187818 10.460304 9.290500

Si 2.727970 10.463600 1.221250

Si 2.018610 7.474000 3.313620

Si 4.391740 10.465384 6.420817

Si 0.354940 7.477647 8.473171

O 3.434710 11.826320 0.556660

O 3.434710 9.100870 0.556660

O 1.102310 10.463600 1.386100

O 1.223890 7.474000 1.760420

O 3.466830 10.463600 2.799080

O 3.663830 7.474000 3.191290

O 1.257330 6.146160 4.002710

O 1.257330 8.801840 4.002710

O 3.635060 11.783592 5.720443

O 3.637757 9.146353 5.719913

O 1.280227 10.464074 6.534822

O 1.091354 7.480747 6.906983

O 3.621429 10.466256 7.975490

O 3.480791 7.459142 8.300718

O 1.054096 6.117081 9.139339

O 1.063526 8.825938 9.155030

Mg 2.543480 13.453200 0.388370

Mg 0.008060 17.893540 2.261970

Mg 0.008060 14.992060 2.261970

Mg 2.341370 16.441390 4.520172

Mg 4.712785 13.448899 5.178175

Mg 2.389659 12.000168 7.463745

Mg 2.376285 14.899991 7.444196

Mg 0.183867 16.442914 9.283165

Si 2.727970 16.442800 1.221250

Si 2.018610 13.453200 3.313620

Si 4.378523 16.441873 6.391221

Si 0.355188 13.446033 8.481361

O 3.434710 17.805520 0.556660

O 3.434710 15.080070 0.556660

O 1.102310 16.442800 1.386100

O 1.223890 13.453200 1.760420

O 3.466830 16.442800 2.799080

O 3.663830 13.453200 3.191290

O 1.257330 12.125360 4.002710

O 1.257330 14.781030 4.002710

O 3.627972 17.763889 5.697566

O 3.628416 15.119505 5.698763

O 1.270038 16.439002 6.520654

O 1.092513 13.445085 6.914528

O 3.588494 16.443482 7.936855

O 3.483036 13.461846 8.319830

O 1.062526 12.095365 9.159509

O 1.053629 14.807879 9.147222

Mg 7.292480 1.494800 0.388370

Mg 4.757060 5.935140 2.261970

Mg 4.757060 3.033660 2.261970

Mg 7.092467 4.484288 4.517499

Mg 9.469133 1.495022 5.177276

Mg 7.130848 0.048953 7.437148

Mg 7.131854 2.944626 7.437171

Mg 4.928900 4.484336 9.271935

Si 7.476970 4.484400 1.221250

Si 6.767610 1.494800 3.313620

Si 9.133512 4.484502 6.385930

Si 5.103068 1.498177 8.465801

O 8.183700 5.847120 0.556660

O 8.183700 3.121670 0.556660

O 5.851310 4.484400 1.386100

O 5.972890 1.494800 1.760420

O 8.215830 4.484400 2.799080

O 8.412830 1.494800 3.191290

O 6.006330 0.166960 4.002710

O 6.006330 2.822640 4.002710

O 8.381367 5.803312 5.690049

O 8.383024 3.162689 5.694718

O 6.023497 4.478583 6.510740

O 5.840298 1.497664 6.899577

O 8.343332 4.486769 7.931361

O 8.237574 1.497403 8.302567

O 5.806233 0.144233 9.138716

O 5.806044 2.852662 9.138275

Mg 7.292480 7.474000 0.388370

Mg 4.757060 11.914340 2.261970

Mg 4.757060 9.012860 2.261970

Mg 7.099378 10.463920 4.532178

Mg 9.463116 7.478635 5.171242

Mg 7.126930 6.018411 7.425306

Mg 7.128441 8.922875 7.439441

Mg 4.972713 10.461240 9.350787

Si 7.476970 10.463600 1.221250

Si 6.767610 7.474000 3.313620

Si 9.150270 10.467378 6.418068

Si 5.102084 7.461208 8.440280

O 8.183700 11.826320 0.556660

O 8.183700 9.100870 0.556660

O 5.851310 10.463600 1.386100

O 5.972890 7.474000 1.760420

O 8.215830 10.463600 2.799080

O 8.412830 7.474000 3.191290

O 6.006330 6.146160 4.002710

O 6.006330 8.801840 4.002710

O 8.386357 11.785651 5.718555

O 8.386463 9.146343 5.724630

O 6.037119 10.468238 6.515526

O 5.840444 7.458228 6.877892

O 8.359615 10.475618 7.967629

O 8.221643 7.462981 8.297895

O 5.807720 6.114139 9.132433

O 5.809278 8.815068 9.093082

Mg 7.292480 13.453200 0.388370

Mg 4.757060 17.893540 2.261970

Mg 4.757060 14.992060 2.261970

Mg 7.092024 16.443717 4.518739

Mg 9.459880 13.451226 5.161555

Mg 7.126736 12.008002 7.436561

Mg 7.119034 14.912188 7.429017

Mg 4.928024 16.451315 9.280833
Si 7.476970 16.442800 1.221250
Si 6.767610 13.453200 3.313620
Si 9.132039 16.443527 6.387275
Si 5.103916 13.462933 8.468577
O 8.183700 17.805520 0.556660
O 8.183700 15.080070 0.556660
O 5.851310 16.442800 1.386100
O 5.972890 13.453200 1.760420
O 8.215830 16.442800 2.799080
O 8.412830 13.453200 3.191290
O 6.006330 12.125360 4.002710
O 6.006330 14.781030 4.002710
O 8.381555 17.766079 5.695345
O 8.378645 15.122348 5.694879
O 6.021742 16.447731 6.511958
O 5.833910 13.457449 6.898876
O 8.341264 16.443817 7.933623
O 8.224676 13.467561 8.293253
O 5.816927 12.118998 9.151185
O 5.807696 14.822570 9.133672
Mg 12.041480 1.494800 0.388370
Mg 9.506060 5.935140 2.261970
Mg 9.506060 3.033660 2.261970
Mg 11.844653 4.487330 4.518440
Mg 14.216489 1.497776 5.178625
Mg 11.883947 0.049662 7.438984
Mg 11.884351 2.947288 7.438393
Mg 9.692673 4.499272 9.264749
Si 12.225970 4.484400 1.221250
Si 11.516610 1.494800 3.313620
Si 13.881580 4.491552 6.390840
Si 9.857782 1.501157 8.467331
O 12.932710 5.847120 0.556660
O 12.932710 3.121670 0.556660
O 10.600310 4.484400 1.386100
O 10.721890 1.494800 1.760420
O 12.964830 4.484400 2.799080
O 13.161830 1.494800 3.191290
O 10.755330 0.166960 4.002710
O 10.755330 2.822640 4.002710
O 13.129585 5.811560 5.697425
O 13.131035 3.169256 5.696797
O 10.773359 4.489650 6.525148
O 10.594331 1.499727 6.900729
O 13.090125 4.489980 7.937250
O 12.982565 1.498843 8.307766
O 10.559426 0.145801 9.141349
O 10.556658 2.858898 9.140239
Mg 12.041480 7.474000 0.388370
Mg 9.506060 11.914340 2.261970
Mg 9.506060 9.012860 2.261970
Mg 11.849708 10.463819 4.525406
Mg 14.219769 7.485074 5.181470

Mg 11.889315 6.035106 7.439282

Mg 11.874728 8.939646 7.414829

Fe 9.592358 10.485015 9.534669

Si 12.225970 10.463600 1.221250

Si 11.516610 7.474000 3.313620

Si 13.894273 10.466679 6.411551

Si 9.838048 7.475112 8.443058

O 12.932710 11.826320 0.556660

O 12.932710 9.100870 0.556660

O 10.600310 10.463600 1.386100

O 10.721890 7.474000 1.760420

O 12.964840 10.463600 2.799080

O 13.161830 7.474000 3.191290

O 10.755330 6.146160 4.002710

O 10.755330 8.801840 4.002710

O 13.140934 11.786385 5.700071

O 13.139202 9.147803 5.700911

O 10.790639 10.467691 6.469341

O 10.588806 7.464155 6.891574

O 13.087647 10.467563 7.935800

O 12.963905 7.487207 8.315400

O 10.549060 6.150146 9.168530

O 10.521445 8.874442 9.067527

Mg 12.041480 13.453200 0.388370

Mg 9.506060 17.893540 2.261970

Mg 9.506060 14.992060 2.261970

Mg 11.844667 16.441430 4.519451

Mg 14.223985 13.446734 5.183514

Mg 11.878993 11.998157 7.403960

Mg 11.894473 14.902817 7.431692

Mg 9.690888 16.446364 9.267060

Si 12.225970 16.442800 1.221250

Si 11.516610 13.453200 3.313620

Si 13.884830 16.443819 6.393265

Si 9.842876 13.476942 8.413930

O 12.932710 17.805520 0.556660

O 12.932710 15.080070 0.556660

O 10.600310 16.442800 1.386100

O 10.721890 13.453200 1.760420

O 12.964840 16.442800 2.799080

O 13.161830 13.453200 3.191290

O 10.755330 12.125360 4.002710

O 10.755330 14.781030 4.002710

O 13.132191 17.764988 5.699291

O 13.134646 15.121810 5.698831

O 10.772739 16.445870 6.519321

O 10.594383 13.475062 6.867793

O 13.092368 16.445063 7.939298

O 12.962997 13.448079 8.319621

O 10.538728 12.089924 9.051560

O 10.552099 14.801449 9.134114

Mg 16.790480 1.494800 0.388370

Mg 14.255060 5.935140 2.261970

Mg 14.255060 3.033660 2.261970

Mg 16.591374 4.487623 4.520082

Mg 18.962404 1.493857 5.178122

Mg 16.628759 0.045264 7.440717

Mg 16.627823 2.945738 7.437540

Mg 14.440787 4.494785 9.272125

Si 16.974970 4.484400 1.221250

Si 16.265610 1.494800 3.313620

Si 18.623894 4.490109 6.392402

Si 14.603652 1.496824 8.470731

O 17.681710 5.847120 0.556660

O 17.681710 3.121670 0.556660

O 15.349310 4.484400 1.386100

O 15.470890 1.494800 1.760420

O 17.713830 4.484400 2.799080

O 17.910830 1.494800 3.191290

O 15.504330 0.166960 4.002710

O 15.504330 2.822640 4.002710

O 17.875119 5.811885 5.699353

O 17.875872 3.167002 5.696611

O 15.522112 4.490020 6.523017

O 15.338065 1.495792 6.902394

O 17.832861 4.489693 7.938088

O 17.731645 1.496370 8.304100

O 15.303403 0.139229 9.141169

O 15.304530 2.854182 9.140646

Mg 16.790480 7.474000 0.388370

Mg 14.255060 11.914340 2.261970

Mg 14.255060 9.012860 2.261970

Mg 16.590312 10.464823 4.532731

Mg 18.963530 7.482104 5.183623

Mg 16.624016 6.040147 7.447049

Mg 16.609177 8.942273 7.462368

Mg 14.288721 10.455328 9.431480

Si 16.974970 10.463600 1.221250

Si 16.265610 7.474000 3.313620

Si 18.632224 10.465841 6.403329

Si 14.588816 7.499331 8.476360

O 17.681710 11.826320 0.556660

O 17.681710 9.100870 0.556660

O 15.349310 10.463600 1.386100

O 15.470890 7.474000 1.760420

O 17.713840 10.463600 2.799080

O 17.910830 7.474000 3.191290

O 15.504330 6.146160 4.002710

O 15.504330 8.801840 4.002710

O 17.883891 11.787860 5.709564

O 17.883178 9.144748 5.707524

O 15.537891 10.465855 6.488819

O 15.329965 7.478993 6.909377

O 17.845134 10.463450 7.948605

O 17.729901 7.483931 8.316146

O 15.291432 6.140633 9.153910

O 15.274877 8.868222 9.127378

Mg 16.790480 13.453200 0.388370

Mg 14.255060 17.893540 2.261970
Mg 14.255060 14.992060 2.261970
Mg 16.592668 16.439346 4.522313
Mg 18.966493 13.445897 5.186695
Mg 16.608300 11.983611 7.470421
Mg 16.625006 14.885203 7.454540
Mg 14.441476 16.434428 9.276517
Si 16.974970 16.442800 1.221250
Si 16.265610 13.453200 3.313620
Si 18.624575 16.434564 6.398097
Si 14.588584 13.428582 8.481446
O 17.681710 17.805520 0.556660
O 17.681710 15.080070 0.556660
O 15.349310 16.442800 1.386100
O 15.470890 13.453200 1.760420
O 17.713840 16.442800 2.799080
O 17.910830 13.453200 3.191290
O 15.504330 12.125360 4.002710
O 15.504330 14.781030 4.002710
O 17.878240 17.758855 5.702137
O 17.876262 15.111566 5.706658
O 15.525391 16.438679 6.524264
O 15.331086 13.445034 6.915338
O 17.833859 16.436865 7.944047
O 17.729668 13.440140 8.325656
O 15.262335 12.056886 9.138404
O 15.292769 14.787827 9.155435

C 6.099105 10.037185 11.890000
C 6.227478 8.616147 12.065215
C 7.475980 8.012049 12.066474
C 8.681458 8.792000 11.945688
C 9.974347 8.228739 12.070318
C 11.159057 9.015515 11.966385
C 12.470382 8.438559 12.034682
C 13.615476 9.230382 11.935008
C 13.498235 10.646904 11.724471
C 12.227076 11.239026 11.606236
C 11.046683 10.445998 11.747549
C 9.734002 11.030484 11.595487
C 8.541005 10.229269 11.704765
C 7.226619 10.826255 11.673472
H 5.112951 10.515321 12.001794
H 5.327751 8.007110 12.199982
H 7.558214 6.927507 12.196677
H 10.072744 7.148714 12.229337
H 12.562389 7.355887 12.175103
H 14.608392 8.772828 12.005437
H 14.390395 11.292579 11.735467
H 12.126562 12.309180 11.387402
H 9.645318 12.122438 11.566161
H 7.123654 11.900213 11.473782

**Anthracene Ni-[010]-fo**

Number of atoms: 360

Mg 2.543480 1.494800 0.388370

Mg 0.008060 5.935140 2.261970

Mg 0.008060 3.033660 2.261970

Mg 2.342543 4.484274 4.517504

Mg 4.718138 1.491640 5.177899

Mg 2.381529 0.044835 7.437918

Mg 2.381433 2.943739 7.434890

Mg 0.184328 4.483267 9.277281

Si 2.727970 4.484400 1.221250

Si 2.018610 1.494800 3.313620

Si 4.382559 4.481955 6.385490

Si 0.359028 1.494210 8.467241

O 3.434710 5.847120 0.556660

O 3.434700 3.121670 0.556660

O 1.102310 4.484400 1.386100

O 1.223890 1.494800 1.760420

O 3.466830 4.484400 2.799080

O 3.663830 1.494800 3.191290

O 1.257330 0.166960 4.002710

O 1.257330 2.822640 4.002710

O 3.630320 5.801669 5.690197

O 3.630859 3.159480 5.694514

O 1.271388 4.484753 6.515435

O 1.093364 1.494222 6.899923

O 3.591135 4.484507 7.929851

O 3.484972 1.495917 8.304376

O 1.060251 0.139218 9.139919

O 1.059914 2.850947 9.137166

Mg 2.543480 7.474000 0.388370

Mg 0.008060 11.914340 2.261970

Mg 0.008060 9.012860 2.261970

Mg 2.349691 10.464888 4.531962

Mg 4.713171 7.475731 5.171274

Mg 2.376204 6.023499 7.431604

Mg 2.389499 8.926201 7.454177

Mg 0.189835 10.459549 9.287591

Si 2.727970 10.463600 1.221250

Si 2.018610 7.474000 3.313620

Si 4.393759 10.465941 6.417163

Si 0.356975 7.476510 8.471650

O 3.434710 11.826320 0.556660

O 3.434710 9.100870 0.556660

O 1.102310 10.463600 1.386100

O 1.223890 7.474000 1.760420

O 3.466830 10.463600 2.799080

O 3.663830 7.474000 3.191290

O 1.257330 6.146160 4.002710

O 1.257330 8.801840 4.002710

O 3.637870 11.784411 5.717786

O 3.640333 9.146159 5.717337

O 1.281945 10.464564 6.531813

O 1.093277 7.478788 6.905308

O 3.616719 10.465750 7.969009

O 3.482348 7.461021 8.302090

O 1.056313 6.116721 9.139529

O 1.065215 8.824858 9.153163

Mg 2.543480 13.453200 0.388370

Mg 0.008060 17.893540 2.261970

Mg 0.008060 14.992060 2.261970

Mg 2.343131 16.440475 4.521575

Mg 4.719407 13.448471 5.184090

Mg 2.391743 12.001584 7.462563

Mg 2.382380 14.897723 7.448977

Mg 0.187001 16.442455 9.282315

Si 2.727970 16.442800 1.221250

Si 2.018610 13.453200 3.313620

Si 4.382108 16.439805 6.394555

Si 0.357540 13.445828 8.482798

O 3.434710 17.805520 0.556660

O 3.434710 15.080070 0.556660

O 1.102310 16.442800 1.386100

O 1.223890 13.453200 1.760420

O 3.466830 16.442800 2.799080

O 3.663830 13.453200 3.191290

O 1.257330 12.125360 4.002710

O 1.257330 14.781030 4.002710

O 3.630979 17.760912 5.698857

O 3.632105 15.116163 5.703966

O 1.272350 16.437858 6.522428

O 1.096189 13.446342 6.916327

O 3.591532 16.442893 7.940204

O 3.490232 13.457148 8.326241

O 1.065003 12.094301 9.160073

O 1.056426 14.806831 9.150169

Mg 7.292480 1.494800 0.388370

Mg 4.757060 5.935140 2.261970

Mg 4.757060 3.033660 2.261970

Mg 7.093152 4.484050 4.517078

Mg 9.469947 1.493161 5.178541

Mg 7.135370 0.047554 7.441292

Mg 7.135676 2.943676 7.439311

Mg 4.934473 4.486832 9.268974

Si 7.476970 4.484400 1.221250

Si 6.767610 1.494800 3.313620

Si 9.133410 4.486247 6.384634

Si 5.105866 1.497989 8.467086

O 8.183700 5.847120 0.556660

O 8.183700 3.121670 0.556660

O 5.851310 4.484400 1.386100

O 5.972890 1.494800 1.760420

O 8.215830 4.484400 2.799080

O 8.412830 1.494800 3.191290

O 6.006330 0.166960 4.002710

O 6.006330 2.822640 4.002710

O 8.380485 5.803291 5.687379

O 8.384553 3.162652 5.694887

O 6.024444 4.479379 6.515579

O 5.843406 1.496390 6.901000

O 8.343962 4.489142 7.930659

O 8.241007 1.498094 8.305494

O 5.808554 0.144837 9.141917

O 5.808155 2.853326 9.139207

Mg 7.292480 7.474000 0.388370

Mg 4.757060 11.914340 2.261970

Mg 4.757060 9.012860 2.261970

Mg 7.103199 10.465220 4.531240

Mg 9.463530 7.478748 5.167932

Mg 7.130122 6.020278 7.426241

Mg 7.135382 8.922917 7.435133

Mg 4.966529 10.463376 9.334056

Si 7.476970 10.463600 1.221250

Si 6.767610 7.474000 3.313620

Si 9.161637 10.466788 6.407226

Si 5.103528 7.464410 8.443245

O 8.183700 11.826320 0.556660

O 8.183700 9.100870 0.556660

O 5.851310 10.463600 1.386100

O 5.972890 7.474000 1.760420

O 8.215830 10.463600 2.799080

O 8.412830 7.474000 3.191290

O 6.006330 6.146160 4.002710

O 6.006330 8.801840 4.002710

O 8.387387 11.783805 5.713156

O 8.388430 9.147133 5.716424

O 6.038826 10.468886 6.522758

O 5.840454 7.456918 6.881704

O 8.376312 10.469503 7.952759

O 8.219605 7.464789 8.298045

O 5.809243 6.121879 9.148780

O 5.810801 8.820895 9.094135

Mg 7.292480 13.453200 0.388370

Mg 4.757060 17.893540 2.261970

Mg 4.757060 14.992060 2.261970

Mg 7.094030 16.442749 4.519983

Mg 9.463859 13.449166 5.166258

Mg 7.140253 12.005907 7.442496

Mg 7.127111 14.907183 7.435279

Mg 4.933344 16.450004 9.282356

Si 7.476970 16.442800 1.221250

Si 6.767610 13.453200 3.313620

Si 9.134215 16.441038 6.389167

Si 5.111836 13.458472 8.481627

O 8.183700 17.805520 0.556660

O 8.183700 15.080070 0.556660

O 5.851310 16.442800 1.386100

O 5.972890 13.453200 1.760420

O 8.215830 16.442800 2.799080

O 8.412830 13.453200 3.191290

O 6.006330 12.125360 4.002710

O 6.006330 14.781030 4.002710

O 8.383116 17.764297 5.697621

O 8.380280 15.120583 5.696184

O 6.024939 16.444593 6.517146

O 5.839178 13.451126 6.910948

O 8.345690 16.442410 7.936420

O 8.230680 13.462354 8.298509

O 5.823207 12.114132 9.169597

O 5.812912 14.820449 9.143819

Mg 12.041480 1.494800 0.388370

Mg 9.506060 5.935140 2.261970

Mg 9.506060 3.033660 2.261970

Mg 11.845565 4.487869 4.517875

Mg 14.217504 1.496535 5.179170

Mg 11.886460 0.048392 7.439493

Mg 11.886764 2.946627 7.438002

Mg 9.708008 4.508025 9.247243

Si 12.225970 4.484400 1.221250

Si 11.516610 1.494800 3.313620

Si 13.881737 4.492875 6.391627

Si 9.861504 1.500367 8.469411

O 12.932710 5.847120 0.556660

O 12.932710 3.121670 0.556660

O 10.600310 4.484400 1.386100

O 10.721890 1.494800 1.760420

O 12.964830 4.484400 2.799080

O 13.161830 1.494800 3.191290

O 10.755330 0.166960 4.002710

O 10.755330 2.822640 4.002710

O 13.130519 5.812230 5.697156

O 13.131830 3.169926 5.698121

O 10.772644 4.490940 6.533324

O 10.596848 1.498626 6.901746

O 13.089982 4.493178 7.938661

O 12.985358 1.499036 8.307386

O 10.562027 0.143839 9.142567

O 10.561661 2.860325 9.139432

Mg 12.041480 7.474000 0.388370

Mg 9.506060 11.914340 2.261970

Mg 9.506060 9.012860 2.261970

Mg 11.848833 10.465275 4.524362

Mg 14.218950 7.485805 5.178975

Mg 11.891074 6.041634 7.440934

Mg 11.867160 8.948035 7.411229

Ni 9.548547 10.496097 9.511381

Si 12.225970 10.463600 1.221250

Si 11.516610 7.474000 3.313620

Si 13.895609 10.466306 6.402663

Si 9.837896 7.498570 8.436150

O 12.932710 11.826320 0.556660

O 12.932710 9.100870 0.556660

O 10.600310 10.463600 1.386100

O 10.721890 7.474000 1.760420

O 12.964840 10.463600 2.799080

O 13.161830 7.474000 3.191290

O 10.755330 6.146160 4.002710

O 10.755330 8.801840 4.002710

O 13.143746 11.787018 5.696713

O 13.142542 9.146300 5.694766

O 10.801148 10.469594 6.442149

O 10.589796 7.458231 6.883529

O 13.091900 10.465399 7.926406

O 12.965290 7.493358 8.319450

O 10.546481 6.172336 9.178724

O 10.523212 8.903113 9.033293

Mg 12.041480 13.453200 0.388370

Mg 9.506060 17.893540 2.261970

Mg 9.506060 14.992060 2.261970

Mg 11.845088 16.440261 4.520376

Mg 14.223583 13.445199 5.184528

Mg 11.873745 11.988974 7.412246

Mg 11.892685 14.895915 7.434955

Mg 9.701691 16.437688 9.264204

Si 12.225970 16.442800 1.221250

Si 11.516610 13.453200 3.313620

Si 13.885089 16.440998 6.394128

Si 9.850139 13.454012 8.427472

O 12.932710 17.805520 0.556660

O 12.932710 15.080070 0.556660

O 10.600310 16.442800 1.386100

O 10.721890 13.453200 1.760420

O 12.964840 16.442800 2.799080

O 13.161830 13.453200 3.191290

O 10.755330 12.125360 4.002710

O 10.755330 14.781030 4.002710

O 13.134046 17.763980 5.700858

O 13.135374 15.119886 5.699274

O 10.774883 16.444376 6.521654

O 10.595001 13.467727 6.872668

O 13.092861 16.441924 7.940012

O 12.966917 13.443285 8.318873

O 10.556527 12.080017 9.074078

O 10.558272 14.790296 9.136152

Mg 16.790480 1.494800 0.388370

Mg 14.255060 5.935140 2.261970

Mg 14.255060 3.033660 2.261970

Mg 16.591770 4.487517 4.519781

Mg 18.963886 1.492705 5.178576

Mg 16.630651 0.042975 7.441234

Mg 16.631464 2.943734 7.437463

Mg 14.447282 4.493438 9.266366

Si 16.974970 4.484400 1.221250

Si 16.265610 1.494800 3.313620

Si 18.625962 4.489688 6.392090

Si 14.606368 1.495573 8.470404

O 17.681710 5.847120 0.556660

O 17.681710 3.121670 0.556660

O 15.349310 4.484400 1.386100

O 15.470890 1.494800 1.760420

O 17.713830 4.484400 2.799080

O 17.910830 1.494800 3.191290

O 15.504330 0.166960 4.002710

O 15.504330 2.822640 4.002710

O 17.876522 5.811035 5.698835

O 17.877346 3.166201 5.697671

O 15.522333 4.489583 6.525534

O 15.340845 1.494996 6.902516

O 17.835115 4.489465 7.938076

O 17.734666 1.494402 8.305806

O 15.305154 0.137857 9.141288

O 15.308930 2.852302 9.140566

Mg 16.790480 7.474000 0.388370

Mg 14.255060 11.914340 2.261970

Mg 14.255060 9.012860 2.261970

Mg 16.590069 10.465232 4.531510

Mg 18.964199 7.481643 5.181523

Mg 16.625901 6.039791 7.445303

Mg 16.612594 8.941677 7.457839

Mg 14.293223 10.455309 9.412040

Si 16.974970 10.463600 1.221250

Si 16.265610 7.474000 3.313620

Si 18.634017 10.465990 6.400379

Si 14.591047 7.500589 8.473401

O 17.681710 11.826320 0.556660

O 17.681710 9.100870 0.556660

O 15.349310 10.463600 1.386100

O 15.470890 7.474000 1.760420

O 17.713840 10.463600 2.799080

O 17.910830 7.474000 3.191290

O 15.504330 6.146160 4.002710

O 15.504330 8.801840 4.002710

O 17.885581 11.788616 5.708457

O 17.885117 9.144747 5.704514

O 15.540037 10.464895 6.487374

O 15.331373 7.478795 6.907534

O 17.847482 10.462892 7.946077

O 17.732158 7.483184 8.313487

O 15.293011 6.142085 9.153093

O 15.282252 8.867254 9.127581

Mg 16.790480 13.453200 0.388370

Mg 14.255060 17.893540 2.261970

Mg 14.255060 14.992060 2.261970

Mg 16.592807 16.439056 4.522493

Mg 18.968322 13.446520 5.187162

Mg 16.611063 11.982875 7.468798

Mg 16.627155 14.883799 7.453966

Mg 14.443813 16.432437 9.274814

Si 16.974970 16.442800 1.221250

Si 16.265610 13.453200 3.313620

Si 18.626783 16.433677 6.398491

Si 14.590892 13.427221 8.482755

O 17.681710 17.805520 0.556660

O 17.681710 15.080070 0.556660

O 15.349310 16.442800 1.386100

O 15.470890 13.453200 1.760420

O 17.713840 16.442800 2.799080

O 17.910830 13.453200 3.191290

O 15.504330 12.125360 4.002710

O 15.504330 14.781030 4.002710

O 17.879837 17.757929 5.703047

O 17.877992 15.111060 5.706778

O 15.526204 16.436766 6.525295

O 15.334027 13.442624 6.916970

O 17.835995 16.435451 7.944674

O 17.732409 13.438825 8.325589

O 15.266799 12.057636 9.145304

O 15.296363 14.786472 9.156304

C 6.053932 9.801534 11.973538

C 6.223521 8.376676 12.020649

C 7.489135 7.815495 11.932267

C 8.659881 8.646712 11.845381

C 9.975620 8.115175 11.878251

C 11.125527 8.942610 11.810499

C 12.454702 8.401135 11.855835

C 13.569398 9.235292 11.831540

C 13.399523 10.661056 11.692402

C 12.121244 11.218834 11.568344

C 10.958287 10.380102 11.664916

C 9.633313 10.927702 11.612001

C 8.476781 10.090438 11.740408

C 7.147012 10.645194 11.793594

H 5.053691 10.232269 12.132125

H 5.345145 7.731635 12.122876

H 7.607161 6.726269 11.948274

H 10.116108 7.029923 11.945911

H 12.575888 7.314697 11.925268

H 14.577663 8.811381 11.898551

H 14.271585 11.332214 11.738266

H 11.989869 12.292539 11.390802

H 9.512042 12.017454 11.622071

H 7.008994 11.728000 11.681913

**Anthracene V$_{MgO}$-[010]-fo** (=)

Number of atoms: 358

Mg 2.543480 1.494800 0.388370

Mg 0.008060 5.935140 2.261970

Mg 0.008060 3.033660 2.261970

Mg 2.348530 4.483256 4.514365

Mg 4.718894 1.482569 5.179419

Mg 2.384940 0.034568 7.434927

Mg 2.383025 2.930037 7.429240

Mg 0.175547 4.473600 9.279381

Si 2.727970 4.484400 1.221250

Si 2.018610 1.494800 3.313620

Si 4.380344 4.471028 6.384649

Si 0.356193 1.489347 8.461256

O 3.434710 5.847120 0.556660

O 3.434700 3.121670 0.556660

O 1.102310 4.484400 1.386100

O 1.223890 1.494800 1.760420

O 3.466830 4.484400 2.799080

O 3.663830 1.494800 3.191290

O 1.257330 0.166960 4.002710

O 1.257330 2.822640 4.002710

O 3.632565 5.788174 5.682812

O 3.631359 3.145511 5.694022

O 1.278176 4.474270 6.502500

O 1.091788 1.484141 6.894891

O 3.583945 4.469310 7.925214

O 3.480797 1.482396 8.305052

O 1.053355 0.135031 9.136841

O 1.057327 2.846309 9.129552

Mg 2.543480 7.474000 0.388370

Mg 0.008060 11.914340 2.261970

Mg 0.008060 9.012860 2.261970

Mg 2.369801 10.464249 4.543540

Mg 4.717266 7.484939 5.178913

Mg 2.365976 6.011375 7.420188

Mg 2.384504 8.917288 7.448568

Mg 0.187076 10.459197 9.275228

Si 2.727970 10.463600 1.221250

Si 2.018610 7.474000 3.313620

Si 4.401888 10.452613 6.458212

Si 0.345555 7.469822 8.465424

O 3.434710 11.826320 0.556660

O 3.434710 9.100870 0.556660

O 1.102310 10.463600 1.386100

O 1.223890 7.474000 1.760420

O 3.466830 10.463600 2.799080

O 3.663830 7.474000 3.191290

O 1.257330 6.146160 4.002710

O 1.257330 8.801840 4.002710

O 3.643211 11.769761 5.738580

O 3.636102 9.142311 5.732862

O 1.278151 10.460823 6.521525

O 1.076259 7.469831 6.898933

O 3.596056 10.453896 7.994630

O 3.451821 7.436318 8.279154

O 1.037765 6.108725 9.133890

O 1.047675 8.818361 9.147185

Mg 2.543480 13.453200 0.388370

Mg 0.008060 17.893540 2.261970

Mg 0.008060 14.992060 2.261970

Mg 2.350618 16.437336 4.518900

Mg 4.737710 13.420744 5.200584

Mg 2.395526 11.996039 7.456820

Mg 2.385163 14.889115 7.434046

Mg 0.183846 16.437574 9.277197

Si 2.727970 16.442800 1.221250

Si 2.018610 13.453200 3.313620

Si 4.388476 16.425691 6.392376

Si 0.356420 13.446332 8.470975

O 3.434710 17.805520 0.556660

O 3.434710 15.080070 0.556660

O 1.102310 16.442800 1.386100

O 1.223890 13.453200 1.760420

O 3.466830 16.442800 2.799080

O 3.663830 13.453200 3.191290

O 1.257330 12.125360 4.002710

O 1.257330 14.781030 4.002710

O 3.638790 17.749472 5.696349

O 3.640607 15.104793 5.698075

O 1.279944 16.437394 6.507326

O 1.088685 13.442231 6.904562

O 3.593479 16.433351 7.935604

O 3.489775 13.460099 8.295999

O 1.056237 12.095676 9.152239

O 1.055983 14.806099 9.138764

Mg 7.292480 1.494800 0.388370

Mg 4.757060 5.935140 2.261970

Mg 4.757060 3.033660 2.261970

Mg 7.088398 4.486683 4.520458

Mg 9.464781 1.490044 5.178436

Mg 7.129131 0.023161 7.452386

Mg 7.122824 2.925516 7.442755

Mg 4.909987 4.466521 9.281936

Si 7.476970 4.484400 1.221250

Si 6.767610 1.494800 3.313620

Si 9.120630 4.488826 6.415419

Si 5.100698 1.475387 8.471439

O 8.183700 5.847120 0.556660

O 8.183700 3.121670 0.556660

O 5.851310 4.484400 1.386100

O 5.972890 1.494800 1.760420

O 8.215830 4.484400 2.799080

O 8.412830 1.494800 3.191290

O 6.006330 0.166960 4.002710

O 6.006330 2.822640 4.002710

O 8.389519 5.820678 5.727057

O 8.389964 3.169179 5.701316

O 6.024608 4.460394 6.507377

O 5.842477 1.474234 6.905810

O 8.323639 4.468161 7.954358

O 8.236597 1.476898 8.309721

O 5.799942 0.117291 9.143049

O 5.798152 2.836248 9.140379

Mg 7.292480 7.474000 0.388370

Mg 4.757060 11.914340 2.261970

Mg 4.757060 9.012860 2.261970

Mg 7.118248 10.461943 4.572242

Mg 9.425201 7.504382 5.183284

Mg 7.094363 6.000799 7.445548

Mg 7.131594 8.934094 7.490306

Mg 4.854341 10.448298 9.486422

Si 7.476970 10.463600 1.221250

Si 6.767610 7.474000 3.313620

Si 9.152252 10.429112 6.490432

Si 5.071705 7.456090 8.425275

O 8.183700 11.826320 0.556660

O 8.183700 9.100870 0.556660

O 5.851310 10.463600 1.386100

O 5.972890 7.474000 1.760420

O 8.215830 10.463600 2.799080

O 8.412830 7.474000 3.191290

O 6.006330 6.146160 4.002710

O 6.006330 8.801840 4.002710

O 8.412024 11.761011 5.746074

O 8.394671 9.131778 5.706299

O 6.042764 10.447529 6.509474

O 5.839600 7.445892 6.878626

O 8.431471 10.390961 7.985525

O 8.138484 7.408134 8.406708

O 5.766917 6.105370 9.133719

O 5.731768 8.822791 9.071606

Mg 7.292480 13.453200 0.388370

Mg 4.757060 17.893540 2.261970

Mg 4.757060 14.992060 2.261970

Mg 7.090317 16.434149 4.521736

Mg 9.442718 13.406938 5.178567

Mg 7.170312 11.921035 7.531697

Mg 7.134178 14.869469 7.448804

Mg 4.937897 16.408953 9.276322

Si 7.476970 16.442800 1.221250

Si 6.767610 13.453200 3.313620

Si 9.131198 16.422379 6.396526

Si 5.112597 13.417892 8.454643

O 8.183700 17.805520 0.556660

O 8.183700 15.080070 0.556660

O 5.851310 16.442800 1.386100

O 5.972890 13.453200 1.760420

O 8.215830 16.442800 2.799080

O 8.412830 13.453200 3.191290

O 6.006330 12.125360 4.002710

O 6.006330 14.781030 4.002710

O 8.388882 17.750026 5.707581

O 8.389661 15.097143 5.708185

O 6.032103 16.426780 6.521780

O 5.878368 13.420761 6.904636

O 8.349538 16.414865 7.946856

O 8.239614 13.440725 8.371904

O 5.779272 12.048855 9.101946

O 5.815126 14.775629 9.148487

Mg 12.041480 1.494800 0.388370

Mg 9.506060 5.935140 2.261970

Mg 9.506060 3.033660 2.261970

Mg 11.840413 4.490317 4.524866

Mg 14.210196 1.515047 5.180088

Mg 11.884450 0.044461 7.434037

Mg 11.870735 2.955330 7.456395

Mg 9.655530 4.453578 9.304865

Si 12.225970 4.484400 1.221250

Si 11.516610 1.494800 3.313620

Si 13.853728 4.524478 6.423190

Si 9.856341 1.477556 8.471086

O 12.932710 5.847120 0.556660

O 12.932710 3.121670 0.556660

O 10.600310 4.484400 1.386100

O 10.721890 1.494800 1.760420

O 12.964830 4.484400 2.799080

O 13.161830 1.494800 3.191290

O 10.755330 0.166960 4.002710

O 10.755330 2.822640 4.002710

O 13.092522 5.851092 5.745267

O 13.116494 3.197820 5.717175

O 10.760093 4.526018 6.567862

O 10.593172 1.497652 6.903490

O 13.067162 4.510475 7.973868

O 12.975363 1.499548 8.311486

O 10.563240 0.117659 9.132150

O 10.549997 2.833146 9.153985

Mg 12.041480 7.474000 0.388370

Mg 9.506060 11.914340 2.261970

Mg 9.506060 9.012860 2.261970

Mg 11.822506 10.473233 4.510708

Mg 14.227890 7.465747 5.160336

Mg 11.819031 6.055872 7.543793

Mg 11.941307 8.932781 7.271881

Si 12.225468 10.459350 1.229146

Si 11.516610 7.474000 3.313620

Si 13.888210 10.463600 6.382630

Si 9.729550 7.487203 8.684256

O 12.928249 11.816561 0.557733

O 12.932710 9.100870 0.556660

O 10.600310 10.463600 1.386100

O 10.721890 7.474000 1.760420

O 12.964840 10.463600 2.799080

O 13.161830 7.474000 3.191290

O 10.755330 6.146160 4.002710

O 10.755330 8.801840 4.002710

O 13.128110 11.791550 5.692940

O 13.159848 9.141931 5.648258

O 10.818639 10.441303 6.447416

O 10.516586 7.623507 7.155491

O 13.092799 10.437986 7.894995

O 12.950541 7.521331 8.346343

O 10.526109 6.106056 9.238194

Mg 12.055650 13.450997 0.411090

Mg 9.504423 17.892699 2.256325

Mg 9.506060 14.992060 2.261970

Mg 11.837740 16.442800 4.520030

Mg 14.211300 13.453200 5.174280

Mg 11.890631 11.965029 7.371798

Mg 11.899518 14.883514 7.434804

Mg 9.704681 16.412431 9.276615

Si 12.223660 16.442081 1.231060

| | | | |
|---|---|---|---|
| Si | 11.510738 | 13.454190 | 3.310427 |
| Si | 13.888220 | 16.442800 | 6.382630 |
| Si | 9.847270 | 13.453200 | 8.472360 |
| O | 12.921432 | 17.801281 | 0.558959 |
| O | 12.927370 | 15.086163 | 0.560337 |
| O | 10.600310 | 16.442800 | 1.386100 |
| O | 10.721890 | 13.453200 | 1.760420 |
| O | 12.964840 | 16.442800 | 2.799080 |
| O | 13.161830 | 13.453200 | 3.191290 |
| O | 10.755330 | 12.125360 | 4.002710 |
| O | 10.755330 | 14.781030 | 4.002710 |
| O | 13.128120 | 17.770750 | 5.692940 |
| O | 13.128120 | 15.114850 | 5.692940 |
| O | 10.773622 | 16.420223 | 6.540487 |
| O | 10.585041 | 13.425280 | 6.935856 |
| O | 13.094978 | 16.436546 | 7.931458 |
| O | 12.970473 | 13.430885 | 8.303859 |
| O | 10.619099 | 12.016764 | 9.057132 |
| O | 10.568111 | 14.752768 | 9.217653 |
| Mg | 16.804157 | 1.494301 | 0.409808 |
| Mg | 14.254572 | 5.934600 | 2.251624 |
| Mg | 14.255060 | 3.033660 | 2.261970 |
| Mg | 16.586740 | 4.484400 | 4.520030 |
| Mg | 18.960290 | 1.494800 | 5.174280 |
| Mg | 16.631655 | 0.048866 | 7.433874 |
| Mg | 16.624848 | 2.945661 | 7.433158 |
| Mg | 14.431095 | 4.476787 | 9.295440 |
| Si | 16.973570 | 4.484480 | 1.231956 |
| Si | 16.261262 | 1.492360 | 3.310175 |
| Si | 18.637210 | 4.484400 | 6.382630 |
| Si | 14.596270 | 1.494800 | 8.472360 |
| O | 17.673533 | 5.841263 | 0.559800 |
| O | 17.674561 | 3.128079 | 0.559738 |
| O | 15.349310 | 4.484400 | 1.386100 |
| O | 15.470890 | 1.494800 | 1.760420 |
| O | 17.713830 | 4.484400 | 2.799080 |
| O | 17.910830 | 1.494800 | 3.191290 |
| O | 15.504330 | 0.166960 | 4.002710 |
| O | 15.504330 | 2.822640 | 4.002710 |
| O | 17.877110 | 5.812340 | 5.692940 |
| O | 17.877110 | 3.156450 | 5.692940 |
| O | 15.498680 | 4.501816 | 6.531432 |
| O | 15.329699 | 1.497537 | 6.903020 |
| O | 17.837486 | 4.484057 | 7.926072 |
| O | 17.730598 | 1.497772 | 8.299272 |
| O | 15.301065 | 0.140567 | 9.140552 |
| O | 15.303822 | 2.848359 | 9.142668 |
| Mg | 16.803437 | 7.475466 | 0.408389 |
| Mg | 14.255104 | 11.911909 | 2.258067 |
| Mg | 14.255060 | 9.012860 | 2.261970 |
| Mg | 16.586740 | 10.463600 | 4.520030 |
| Mg | 18.960300 | 7.474000 | 5.174280 |
| Mg | 16.630349 | 6.029212 | 7.434885 |
| Mg | 16.618483 | 8.934084 | 7.444781 |

Mg 14.332760 10.460841 9.334124
Si 16.973763 10.464027 1.232411
Si 16.262973 7.476409 3.307739
Si 18.637210 10.463600 6.382630
Si 14.596270 7.474000 8.472360
O 17.674613 11.819414 0.558932
O 17.674484 9.108129 0.559107
O 15.349310 10.463600 1.386100
O 15.470890 7.474000 1.760420
O 17.713840 10.463600 2.799080
O 17.910830 7.474000 3.191290
O 15.504330 6.146160 4.002710
O 15.504330 8.801840 4.002710
O 17.877120 11.791540 5.692940
O 17.877110 9.135650 5.692940
O 15.529129 10.464239 6.489250
O 15.317894 7.471327 6.907496
O 17.842119 10.463378 7.930184
O 17.716650 7.476804 8.306832
O 15.282587 6.120831 9.151771
O 15.268999 8.846815 9.136564
Mg 16.804967 13.453299 0.411110
Mg 14.254377 17.891697 2.255889
Mg 14.255060 14.992060 2.261970
Mg 16.586740 16.442800 4.520030
Mg 18.960300 13.453200 5.174280
Mg 16.620834 11.995440 7.446190
Mg 16.637589 14.894077 7.436159
Mg 14.434237 16.442949 9.279097
Si 16.973666 16.442699 1.232005
Si 16.261527 13.452066 3.310558
Si 18.637220 16.442800 6.382630
Si 14.596270 13.453200 8.472360
O 17.675030 17.798579 0.559402
O 17.674921 15.087538 0.559163
O 15.349310 16.442800 1.386100
O 15.470890 13.453200 1.760420
O 17.713840 16.442800 2.799080
O 17.910830 13.453200 3.191290
O 15.504330 12.125360 4.002710
O 15.504330 14.781030 4.002710
O 17.877120 17.770750 5.692940
O 17.877120 15.114850 5.692940
O 15.527030 16.441161 6.512309
O 15.333084 13.457092 6.906349
O 17.841583 16.442447 7.928954
O 17.728458 13.447328 8.313494
O 15.265845 12.075077 9.132917
O 15.296020 14.803848 9.151382
C 4.730887 12.075477 12.280888
C 4.389101 10.691062 12.137413
C 5.379212 9.744259 11.825424
C 6.735553 10.155675 11.567308
C 7.721257 9.237060 11.086957

C 8.993716 9.673043 10.700679

C 10.048167 8.671009 10.267384

C 11.437377 9.184353 10.492667

C 11.732394 10.511337 10.624503

C 10.683022 11.589282 10.448341

C 9.323301 11.064603 10.871222

C 8.391865 11.966606 11.386186

C 7.066454 11.553760 11.711595

C 6.048474 12.486907 12.092964

H 3.948610 12.801125 12.525476

H 3.361684 10.359692 12.331545

H 5.128721 8.679654 11.754477

H 7.438353 8.186303 10.954825

H 9.959984 7.798978 10.953702

H 12.221274 8.425996 10.614088

H 12.721465 10.828289 11.008087

H 10.947349 12.479494 11.053363

H 8.664707 13.022589 11.508094

H 6.311328 13.548753 12.169383

**Anthracene $V_{MgO}$-[010]-fo ($\perp$)**

Number of atoms: 358

Mg 2.543480 1.494800 0.388370

Mg 0.008060 5.935140 2.261970

Mg 0.008060 3.033660 2.261970

Mg 2.341496 4.483172 4.516995

Mg 4.719708 1.484583 5.182376

Mg 2.375206 0.040210 7.441485

Mg 2.371655 2.937317 7.436015

Mg 0.170477 4.479169 9.285462

Si 2.727970 4.484400 1.221250

Si 2.018610 1.494800 3.313620

Si 4.372812 4.472073 6.388071

Si 0.347877 1.492609 8.467317

O 3.434710 5.847120 0.556660

O 3.434700 3.121670 0.556660

O 1.102310 4.484400 1.386100

O 1.223890 1.494800 1.760420

O 3.466830 4.484400 2.799080

O 3.663830 1.494800 3.191290

O 1.257330 0.166960 4.002710

O 1.257330 2.822640 4.002710

O 3.628116 5.796072 5.692168

O 3.621276 3.150581 5.696056

O 1.258236 4.484147 6.517459

O 1.084413 1.490027 6.900458

O 3.581422 4.476212 7.931038

O 3.475505 1.489459 8.305626

O 1.047555 0.138489 9.141238

O 1.048655 2.849926 9.135909

Mg 2.543480 7.474000 0.388370

Mg 0.008060 11.914340 2.261970

Mg 0.008060 9.012860 2.261970

Mg 2.355161 10.464860 4.530014

Mg 4.718772 7.477348 5.181225

Mg 2.368391 6.018612 7.433118

Mg 2.378016 8.921537 7.445976

Mg 0.180589 10.460685 9.295179

Si 2.727970 10.463600 1.221250

Si 2.018610 7.474000 3.313620

Si 4.396132 10.457187 6.418253

Si 0.344411 7.471145 8.469372

O 3.434710 11.826320 0.556660

O 3.434710 9.100870 0.556660

O 1.102310 10.463600 1.386100

O 1.223890 7.474000 1.760420

O 3.466830 10.463600 2.799080

O 3.663830 7.474000 3.191290

O 1.257330 6.146160 4.002710

O 1.257330 8.801840 4.002710

O 3.643497 11.777288 5.716063

O 3.637125 9.143668 5.711808

O 1.278232 10.463253 6.527615

O 1.079902 7.472855 6.902680

O 3.600039 10.457683 7.961611

O 3.467053 7.461661 8.292763

O 1.040561 6.112922 9.138752

O 1.045676 8.823623 9.144369

Mg 2.543480 13.453200 0.388370

Mg 0.008060 17.893540 2.261970

Mg 0.008060 14.992060 2.261970

Mg 2.345639 16.440451 4.523269

Mg 4.728592 13.442296 5.195341

Mg 2.392143 12.000347 7.461606

Mg 2.388390 14.892858 7.451675

Mg 0.181076 16.439067 9.286616

Si 2.727970 16.442800 1.221250

Si 2.018610 13.453200 3.313620

Si 4.381729 16.436750 6.400523

Si 0.356903 13.451134 8.478277

O 3.434710 17.805520 0.556660

O 3.434710 15.080070 0.556660

O 1.102310 16.442800 1.386100

O 1.223890 13.453200 1.760420

O 3.466830 16.442800 2.799080

O 3.663830 13.453200 3.191290

O 1.257330 12.125360 4.002710

O 1.257330 14.781030 4.002710

O 3.629799 17.755414 5.701173

O 3.635939 15.110710 5.708612

O 1.273130 16.434714 6.522079

O 1.098076 13.445057 6.913707

O 3.589657 16.439085 7.944652

O 3.493840 13.449537 8.334364

O 1.052402 12.094750 9.154132

O 1.058768 14.807926 9.147220

Mg 7.292480 1.494800 0.388370

Mg 4.757060 5.935140 2.261970

Mg 4.757060 3.033660 2.261970
Mg 7.094716 4.479607 4.515783
Mg 9.467301 1.477114 5.189526
Mg 7.144282 0.025646 7.476465
Mg 7.124029 2.918955 7.436248
Mg 4.907647 4.469843 9.291022
Si 7.476970 4.484400 1.221250
Si 6.767610 1.494800 3.313620
Si 9.123873 4.473332 6.409196
Si 5.096747 1.482546 8.471395
O 8.183700 5.847120 0.556660
O 8.183700 3.121670 0.556660
O 5.851310 4.484400 1.386100
O 5.972890 1.494800 1.760420
O 8.215830 4.484400 2.799080
O 8.412830 1.494800 3.191290
O 6.006330 0.166960 4.002710
O 6.006330 2.822640 4.002710
O 8.387306 5.797330 5.704199
O 8.385922 3.156328 5.696573
O 6.016667 4.460531 6.499307
O 5.838609 1.469460 6.908022
O 8.322830 4.458566 7.943484
O 8.227831 1.488187 8.324865
O 5.795512 0.133163 9.157693
O 5.794240 2.844429 9.134990
Mg 7.292480 7.474000 0.388370
Mg 4.757060 11.914340 2.261970
Mg 4.757060 9.012860 2.261970
Mg 7.114833 10.459351 4.541424
Mg 9.438681 7.481722 5.204676
Mg 7.085235 5.982713 7.415378
Mg 7.185072 8.913540 7.491335
Mg 4.958919 10.458521 9.298824
Si 7.476970 10.463600 1.221250
Si 6.767610 7.474000 3.313620
Si 9.162328 10.440313 6.416032
Si 5.087908 7.461931 8.451225
O 8.183700 11.826320 0.556660
O 8.183700 9.100870 0.556660
O 5.851310 10.463600 1.386100
O 5.972890 7.474000 1.760420
O 8.215830 10.463600 2.799080
O 8.412830 7.474000 3.191290
O 6.006330 6.146160 4.002710
O 6.006330 8.801840 4.002710
O 8.398821 11.758263 5.691981
O 8.382999 9.126157 5.707701
O 6.037633 10.450290 6.565837
O 5.837097 7.477310 6.897616
O 8.480633 10.445794 7.959948
O 8.179233 7.354594 8.294539
O 5.778535 6.101057 9.130260
O 5.788523 8.800833 9.155320

Mg 7.292480 13.453200 0.388370
Mg 4.757060 17.893540 2.261970
Mg 4.757060 14.992060 2.261970
Mg 7.107354 16.445475 4.533792
Mg 9.430150 13.447767 5.129959
Mg 7.168269 11.961892 7.476907
Mg 7.116347 14.907056 7.425770
Mg 4.927253 16.433769 9.292444
Si 7.476970 16.442800 1.221250
Si 6.767610 13.453200 3.313620
Si 9.144299 16.435957 6.428939
Si 5.118047 13.443149 8.496540
O 8.183700 17.805520 0.556660
O 8.183700 15.080070 0.556660
O 5.851310 16.442800 1.386100
O 5.972890 13.453200 1.760420
O 8.215830 16.442800 2.799080
O 8.412830 13.453200 3.191290
O 6.006330 12.125360 4.002710
O 6.006330 14.781030 4.002710
O 8.380505 17.759157 5.734818
O 8.377409 15.129162 5.705471
O 6.023379 16.437944 6.531341
O 5.840146 13.424148 6.927837
O 8.374982 16.416266 7.980274
O 8.215965 13.473580 8.241897
O 5.824772 12.103240 9.200598
O 5.818449 14.805888 9.171272
Mg 12.041480 1.494800 0.388370
Mg 9.506060 5.935140 2.261970
Mg 9.506060 3.033660 2.261970
Mg 11.837764 4.485592 4.530581
Mg 14.206690 1.512527 5.183551
Mg 11.861014 0.023651 7.441637
Mg 11.862071 2.941492 7.469287
Mg 9.642909 4.406368 9.308757
Si 12.225970 4.484400 1.221250
Si 11.516610 1.494800 3.313620
Si 13.849185 4.520444 6.431751
Si 9.849104 1.443200 8.483188
O 12.932710 5.847120 0.556660
O 12.932710 3.121670 0.556660
O 10.600310 4.484400 1.386100
O 10.721890 1.494800 1.760420
O 12.964830 4.484400 2.799080
O 13.161830 1.494800 3.191290
O 10.755330 0.166960 4.002710
O 10.755330 2.822640 4.002710
O 13.093556 5.855837 5.760096
O 13.116285 3.198323 5.718088
O 10.762667 4.517806 6.558782
O 10.585254 1.491917 6.911643
O 13.062964 4.502238 7.980664
O 12.972066 1.485533 8.310982

O 10.527874 0.062235 9.112454

O 10.552436 2.795292 9.172802

Mg 12.041480 7.474000 0.388370

Mg 9.506060 11.914340 2.261970

Mg 9.506060 9.012860 2.261970

Mg 11.833927 10.474724 4.491812

Mg 14.232532 7.462585 5.167510

Mg 11.835802 6.052789 7.553268

Mg 11.953225 8.937958 7.253087

Si 12.225220 10.459281 1.228059

Si 11.516610 7.474000 3.313620

Si 13.917458 10.482902 6.346256

Si 9.756433 7.422056 8.702625

O 12.927480 11.816756 0.557648

O 12.932710 9.100870 0.556660

O 10.600310 10.463600 1.386100

O 10.721890 7.474000 1.760420

O 12.964840 10.463600 2.799080

O 13.161830 7.474000 3.191290

O 10.755330 6.146160 4.002710

O 10.755330 8.801840 4.002710

O 13.157202 11.791144 5.650931

O 13.180038 9.150766 5.637587

O 10.821675 10.427809 6.395475

O 10.545309 7.610144 7.168825

O 13.095450 10.459015 7.877670

O 12.933043 7.563935 8.380810

O 10.541747 6.043493 9.233579

Mg 12.055245 13.451902 0.410883

Mg 9.503811 17.893153 2.256645

Mg 9.506060 14.992060 2.261970

Mg 11.832744 16.443479 4.524295

Mg 14.223924 13.471341 5.163934

Mg 11.860844 11.975456 7.320171

Mg 11.891978 14.898514 7.385403

Mg 9.667065 16.341988 9.447334

Si 12.223719 16.442562 1.231015

Si 11.510729 13.454596 3.310357

Si 13.888223 16.456353 6.386024

Si 9.828086 13.478865 8.290165

O 12.921637 17.801121 0.558133

O 12.927879 15.086099 0.560498

O 10.600310 16.442800 1.386100

O 10.721890 13.453200 1.760420

O 12.964840 16.442800 2.799080

O 13.161830 13.453200 3.191290

O 10.755330 12.125360 4.002710

O 10.755330 14.781030 4.002710

O 13.134683 17.776182 5.696550

O 13.147457 15.132457 5.683257

O 10.789510 16.442799 6.469008

O 10.609852 13.466174 6.766233

O 13.091949 16.435284 7.924604

O 12.951426 13.402611 8.270129

O 10.409613 11.977178 8.848375

O 10.594741 14.748560 9.005000

Mg 16.804357 1.494062 0.410830

Mg 14.254289 5.933502 2.251826

Mg 14.255060 3.033660 2.261970

Mg 16.587978 4.483446 4.522065

Mg 18.959762 1.496717 5.179211

Mg 16.624074 0.046035 7.440268

Mg 16.613172 2.948805 7.443650

Mg 14.418928 4.479112 9.311331

Si 16.973529 4.484192 1.232112

Si 16.260969 1.492449 3.311010

Si 18.612879 4.493401 6.399817

Si 14.592394 1.490467 8.470435

O 17.673800 5.841366 0.560091

O 17.674689 3.128272 0.559393

O 15.349310 4.484400 1.386100

O 15.470890 1.494800 1.760420

O 17.713830 4.484400 2.799080

O 17.910830 1.494800 3.191290

O 15.504330 0.166960 4.002710

O 15.504330 2.822640 4.002710

O 17.860502 5.814033 5.704168

O 17.863529 3.171470 5.702190

O 15.493502 4.506736 6.541766

O 15.330380 1.498643 6.903811

O 17.818947 4.492321 7.945133

O 17.723462 1.499442 8.303536

O 15.294517 0.133321 9.138957

O 15.292620 2.848952 9.143349

Mg 16.803118 7.474916 0.408043

Mg 14.254769 11.912909 2.256354

Mg 14.255060 9.012860 2.261970

Mg 16.602915 10.467432 4.523443

Mg 18.961790 7.476275 5.179929

Mg 16.601421 6.034390 7.455283

Mg 16.623279 8.929415 7.452418

Mg 14.390550 10.478773 9.271171

Si 16.973695 10.463877 1.232160

Si 16.262759 7.475828 3.307643

Si 18.633356 10.465591 6.401571

Si 14.573481 7.505609 8.504758

O 17.674379 11.819381 0.559196

O 17.674205 9.107820 0.559374

O 15.349310 10.463600 1.386100

O 15.470890 7.474000 1.760420

O 17.713840 10.463600 2.799080

O 17.910830 7.474000 3.191290

O 15.504330 6.146160 4.002710

O 15.504330 8.801840 4.002710

O 17.886773 11.789070 5.704819

O 17.879891 9.144002 5.706190

O 15.549784 10.471573 6.500911

O 15.298391 7.488875 6.933147

O 17.839285 10.467506 7.945838

O 17.716368 7.473428 8.311306

O 15.250084 6.136806 9.169567

O 15.293722 8.855909 9.162191

Mg 16.804794 13.453598 0.410679

Mg 14.254084 17.892355 2.257135

Mg 14.255060 14.992060 2.261970

Mg 16.592280 16.442812 4.525900

Mg 18.972477 13.452989 5.185388

Mg 16.633431 12.013072 7.448405

Mg 16.616998 14.903395 7.448219

Mg 14.399855 16.445521 9.294440

Si 16.973613 16.442885 1.232173

Si 16.261566 13.453174 3.310345

Si 18.627938 16.436924 6.398535

Si 14.572589 13.444233 8.453896

O 17.675321 17.798194 0.558978

O 17.674884 15.087287 0.559460

O 15.349310 16.442800 1.386100

O 15.470890 13.453200 1.760420

O 17.713840 16.442800 2.799080

O 17.910830 13.453200 3.191290

O 15.504330 12.125360 4.002710

O 15.504330 14.781030 4.002710

O 17.880956 17.760344 5.701479

O 17.878194 15.113974 5.705648

O 15.530556 16.446095 6.510516

O 15.332919 13.452784 6.898467

O 17.834316 16.440719 7.943654

O 17.730814 13.460438 8.316610

O 15.286209 12.097982 9.134410

O 15.256825 14.802540 9.139159

C 11.068003 15.678724 12.017107

C 9.649877 15.928119 11.894659

C 8.769611 14.849396 11.732916

C 9.261102 13.497083 11.703697

C 8.376785 12.398123 11.623086

C 8.869190 11.085422 11.462757

C 7.978066 9.971691 11.301425

C 8.405628 8.833414 10.647722

C 9.753954 8.726913 10.113543

C 10.700115 9.637247 10.600062

C 10.295697 10.849456 11.290938

C 11.179106 11.927311 11.566505

C 10.696400 13.247733 11.764465

C 11.568012 14.381949 11.935397

H 11.741807 16.525631 12.190080

H 9.261018 16.945071 12.047098

H 7.685267 15.003861 11.642683

H 7.293555 12.568065 11.581261

H 6.936991 10.059756 11.637630

H 7.678153 8.044705 10.430423

H 11.762469 9.512574 10.337501

H 12.266079 11.773489 11.507819

H 12.647940 14.205027 12.016106

H 9.672263 11.294012 8.721762

**Fluoranthene [010]-fo**

Number of atoms: 362

Mg 2.543480 1.494800 0.388370

Mg 0.008060 5.935140 2.261970

Mg 0.008060 3.033660 2.261970

Mg 2.355193 4.481105 4.520006

Mg 4.724312 1.496146 5.184959

Mg 2.389739 0.045223 7.448973

Mg 2.398800 2.942254 7.442386

Mg 0.159871 4.469526 9.256056

Si 2.727970 4.484400 1.221250

Si 2.018610 1.494800 3.313620

Si 4.394749 4.479183 6.394556

Si 0.365705 1.495538 8.488664

O 3.434710 5.847120 0.556660

O 3.434700 3.121670 0.556660

O 1.102310 4.484400 1.386100

O 1.223890 1.494800 1.760420

O 3.466830 4.484400 2.799080

O 3.663830 1.494800 3.191290

O 1.257330 0.166960 4.002710

O 1.257330 2.822640 4.002710

O 3.630705 5.796944 5.692927

O 3.630373 3.159061 5.700145

O 1.267334 4.485917 6.536207

O 1.097883 1.501904 6.920267

O 3.602423 4.483153 7.932896

O 3.485451 1.491236 8.320728

O 1.061664 0.137859 9.158203

O 1.066240 2.847893 9.184948

Mg 2.543480 7.474000 0.388370

Mg 0.008060 11.914340 2.261970

Mg 0.008060 9.012860 2.261970

Mg 2.354812 10.464864 4.526205

Mg 4.712060 7.469902 5.156389

Mg 2.392354 6.021664 7.426765

Mg 2.398263 8.918921 7.441100

Mg 0.184496 10.461427 9.270687

Si 2.727970 10.463600 1.221250

Si 2.018610 7.474000 3.313620

Si 4.393672 10.458879 6.409960

Si 0.346913 7.482509 8.476788

O 3.434710 11.826320 0.556660

O 3.434710 9.100870 0.556660

O 1.102310 10.463600 1.386100

O 1.223890 7.474000 1.760420

O 3.466830 10.463600 2.799080

O 3.663830 7.474000 3.191290

O 1.257330 6.146160 4.002710

O 1.257330 8.801840 4.002710

O 3.632184 11.780029 5.716158

| | | | |
|---|---|---|---|
| O | 3.631102 | 9.142726 | 5.707208 |
| O | 1.273444 | 10.457026 | 6.541401 |
| O | 1.091525 | 7.479107 | 6.920948 |
| O | 3.607553 | 10.453229 | 7.959013 |
| O | 3.493014 | 7.466258 | 8.297445 |
| O | 1.025622 | 6.119035 | 9.168928 |
| O | 1.062805 | 8.815125 | 9.199982 |
| Mg | 2.543480 | 13.453200 | 0.388370 |
| Mg | 0.008060 | 17.893540 | 2.261970 |
| Mg | 0.008060 | 14.992060 | 2.261970 |
| Mg | 2.348488 | 16.442378 | 4.522639 |
| Mg | 4.723404 | 13.444338 | 5.189327 |
| Mg | 2.394376 | 11.998717 | 7.456020 |
| Mg | 2.388770 | 14.895834 | 7.447357 |
| Mg | 0.188356 | 16.440636 | 9.283821 |
| Si | 2.727970 | 16.442800 | 1.221250 |
| Si | 2.018610 | 13.453200 | 3.313620 |
| Si | 4.385576 | 16.437348 | 6.398602 |
| Si | 0.360882 | 13.450164 | 8.474018 |
| O | 3.434710 | 17.805520 | 0.556660 |
| O | 3.434710 | 15.080070 | 0.556660 |
| O | 1.102310 | 16.442800 | 1.386100 |
| O | 1.223890 | 13.453200 | 1.760420 |
| O | 3.466830 | 16.442800 | 2.799080 |
| O | 3.663830 | 13.453200 | 3.191290 |
| O | 1.257330 | 12.125360 | 4.002710 |
| O | 1.257330 | 14.781030 | 4.002710 |
| O | 3.635178 | 17.759548 | 5.703651 |
| O | 3.634504 | 15.114642 | 5.704925 |
| O | 1.277435 | 16.442383 | 6.524669 |
| O | 1.099285 | 13.446047 | 6.908855 |
| O | 3.595561 | 16.437399 | 7.944671 |
| O | 3.482650 | 13.449745 | 8.326667 |
| O | 1.058036 | 12.094342 | 9.153139 |
| O | 1.058999 | 14.806358 | 9.147170 |
| Mg | 7.292480 | 1.494800 | 0.388370 |
| Mg | 4.757060 | 5.935140 | 2.261970 |
| Mg | 4.757060 | 3.033660 | 2.261970 |
| Mg | 7.101103 | 4.480377 | 4.530101 |
| Mg | 9.469474 | 1.494481 | 5.186904 |
| Mg | 7.134426 | 0.046152 | 7.450763 |
| Mg | 7.130628 | 2.948372 | 7.452083 |
| Mg | 4.880604 | 4.494946 | 9.361261 |
| Si | 7.476970 | 4.484400 | 1.221250 |
| Si | 6.767610 | 1.494800 | 3.313620 |
| Si | 9.148396 | 4.468984 | 6.409211 |
| Si | 5.109042 | 1.497513 | 8.482565 |
| O | 8.183700 | 5.847120 | 0.556660 |
| O | 8.183700 | 3.121670 | 0.556660 |
| O | 5.851310 | 4.484400 | 1.386100 |
| O | 5.972890 | 1.494800 | 1.760420 |
| O | 8.215830 | 4.484400 | 2.799080 |
| O | 8.412830 | 1.494800 | 3.191290 |
| O | 6.006330 | 0.166960 | 4.002710 |

O 6.006330 2.822640 4.002710

O 8.396646 5.786096 5.694588

O 8.388135 3.150269 5.710654

O 6.036489 4.467343 6.478925

O 5.840183 1.490289 6.913098

O 8.356394 4.478326 7.944107

O 8.230556 1.489851 8.326989

O 5.805832 0.136918 9.157540

O 5.800805 2.857200 9.152809

Mg 7.292480 7.474000 0.388370

Mg 4.757060 11.914340 2.261970

Mg 4.757060 9.012860 2.261970

Mg 7.090924 10.463127 4.521109

Mg 9.469389 7.462087 5.176966

Mg 7.139134 6.005725 7.408057

Mg 7.139629 8.912621 7.408290

Mg 4.916716 10.404451 9.360938

Si 7.476970 10.463600 1.221250

Si 6.767610 7.474000 3.313620

Si 9.136550 10.463275 6.382387

Si 5.125421 7.456435 8.393629

O 8.183700 11.826320 0.556660

O 8.183700 9.100870 0.556660

O 5.851310 10.463600 1.386100

O 5.972890 7.474000 1.760420

O 8.215830 10.463600 2.799080

O 8.412830 7.474000 3.191290

O 6.006330 6.146160 4.002710

O 6.006330 8.801840 4.002710

O 8.384986 11.788201 5.698022

O 8.390653 9.142956 5.681996

O 6.036300 10.463714 6.490543

O 5.853354 7.463387 6.832768

O 8.348596 10.452937 7.927274

O 8.240826 7.462994 8.282333

O 5.822986 6.091127 9.036195

O 5.840542 8.810287 9.049486

Mg 7.292480 13.453200 0.388370

Mg 4.757060 17.893540 2.261970

Mg 4.757060 14.992060 2.261970

Mg 7.094944 16.442269 4.523116

Mg 9.467704 13.451749 5.180554

Mg 7.126042 11.983809 7.451397

Mg 7.131600 14.885210 7.453068

Mg 4.949187 16.428683 9.276709

Si 7.476970 16.442800 1.221250

Si 6.767610 13.453200 3.313620

Si 9.128070 16.436533 6.399268

Si 5.104268 13.426728 8.491542

O 8.183700 17.805520 0.556660

O 8.183700 15.080070 0.556660

O 5.851310 16.442800 1.386100

O 5.972890 13.453200 1.760420

O 8.215830 16.442800 2.799080

O 8.412830 13.453200 3.191290

O 6.006330 12.125360 4.002710

O 6.006330 14.781030 4.002710

O 8.380195 17.758516 5.704286

O 8.379530 15.112715 5.706433

O 6.026805 16.433793 6.530758

O 5.838824 13.436238 6.921444

O 8.339374 16.434335 7.945660

O 8.235271 13.435147 8.319417

O 5.785403 12.055920 9.154769

O 5.810075 14.784073 9.161789

Mg 12.041480 1.494800 0.388370

Mg 9.506060 5.935140 2.261970

Mg 9.506060 3.033660 2.261970

Mg 11.835067 4.482406 4.525743

Mg 14.212265 1.494828 5.178486

Mg 11.877943 0.054876 7.448299

Mg 11.864088 2.960034 7.457111

Mg 9.590388 4.501024 9.413686

Si 12.225970 4.484400 1.221250

Si 11.516610 1.494800 3.313620

Si 13.880609 4.480047 6.385974

Si 9.852905 1.511201 8.484335

O 12.932710 5.847120 0.556660

O 12.932710 3.121670 0.556660

O 10.600310 4.484400 1.386100

O 10.721890 1.494800 1.760420

O 12.964830 4.484400 2.799080

O 13.161830 1.494800 3.191290

O 10.755330 0.166960 4.002710

O 10.755330 2.822640 4.002710

O 13.133849 5.803528 5.693688

O 13.132153 3.157082 5.698140

O 10.792427 4.477868 6.482737

O 10.588077 1.497702 6.914047

O 13.099404 4.484740 7.932489

O 12.980778 1.504456 8.318317

O 10.558845 0.152924 9.157827

O 10.532300 2.880760 9.143078

Mg 12.041480 7.474000 0.388370

Mg 9.506060 11.914340 2.261970

Mg 9.506060 9.012860 2.261970

Mg 11.841202 10.461239 4.520081

Mg 14.210084 7.470678 5.174185

Mg 11.869635 6.011507 7.437966

Mg 11.886462 8.916506 7.439485

Mg 9.699799 10.451719 9.253773

Si 12.225970 10.463600 1.221250

Si 11.516610 7.474000 3.313620

Si 13.881478 10.465342 6.390608

Si 9.858801 7.456638 8.453783

O 12.932710 11.826320 0.556660

O 12.932710 9.100870 0.556660

O 10.600310 10.463600 1.386100

O 10.721890 7.474000 1.760420

O 12.964840 10.463600 2.799080

O 13.161830 7.474000 3.191290

O 10.755330 6.146160 4.002710

O 10.755330 8.801840 4.002710

O 13.130908 11.788308 5.699494

O 13.133137 9.143449 5.697246

O 10.777721 10.464599 6.521997

O 10.601060 7.484085 6.888871

O 13.091835 10.463403 7.937487

O 12.982293 7.467091 8.311744

O 10.560802 6.089112 9.092211

O 10.554231 8.804850 9.161476

Mg 12.041480 13.453200 0.388370

Mg 9.506060 17.893540 2.261970

Mg 9.506060 14.992060 2.261970

Mg 11.841232 16.444485 4.522446

Mg 14.215074 13.456481 5.178255

Mg 11.882153 12.006462 7.439004

Mg 11.877930 14.903835 7.444101

Mg 9.691924 16.451448 9.278265

Si 12.225970 16.442800 1.221250

Si 11.516610 13.453200 3.313620

Si 13.879970 16.445032 6.397060

Si 9.856432 13.448572 8.475967

O 12.932710 17.805520 0.556660

O 12.932710 15.080070 0.556660

O 10.600310 16.442800 1.386100

O 10.721890 13.453200 1.760420

O 12.964840 16.442800 2.799080

O 13.161830 13.453200 3.191290

O 10.755330 12.125360 4.002710

O 10.755330 14.781030 4.002710

O 13.128040 17.765122 5.703014

O 13.129662 15.122186 5.703305

O 10.769539 16.442685 6.526525

O 10.590134 13.453659 6.907271

O 13.088555 16.446149 7.942697

O 12.982634 13.454779 8.311274

O 10.564511 12.092667 9.144497

O 10.552494 14.808337 9.146533

Mg 16.790480 1.494800 0.388370

Mg 14.255060 5.935140 2.261970

Mg 14.255060 3.033660 2.261970

Mg 16.585985 4.483354 4.515789

Mg 18.972126 1.493682 5.184206

Mg 16.632091 0.048676 7.445693

Mg 16.624518 2.941589 7.429961

Mg 14.436904 4.485636 9.273445

Si 16.974970 4.484400 1.221250

Si 16.265610 1.494800 3.313620

Si 18.625681 4.484895 6.381955

Si 14.601846 1.498872 8.472505

O 17.681710 5.847120 0.556660

O 17.681710 3.121670 0.556660
O 15.349310 4.484400 1.386100
O 15.470890 1.494800 1.760420
O 17.713830 4.484400 2.799080
O 17.910830 1.494800 3.191290
O 15.504330 0.166960 4.002710
O 15.504330 2.822640 4.002710
O 17.880799 5.809284 5.690560
O 17.880759 3.162028 5.689802
O 15.524895 4.483079 6.506370
O 15.337654 1.492231 6.904150
O 17.824211 4.486144 7.922052
O 17.741550 1.503728 8.310893
O 15.306054 0.143788 9.145058
O 15.312322 2.852703 9.140387
Mg 16.790480 7.474000 0.388370
Mg 14.255060 11.914340 2.261970
Mg 14.255060 9.012860 2.261970
Mg 16.591558 10.464449 4.518473
Mg 18.966539 7.476959 5.176101
Mg 16.621124 6.027153 7.425437
Mg 16.620110 8.925908 7.430964
Mg 14.435107 10.463685 9.277515
Si 16.974970 10.463600 1.221250
Si 16.265610 7.474000 3.313620
Si 18.630522 10.464568 6.393293
Si 14.601928 7.471469 8.468037
O 17.681710 11.826320 0.556660
O 17.681710 9.100870 0.556660
O 15.349310 10.463600 1.386100
O 15.470890 7.474000 1.760420
O 17.713840 10.463600 2.799080
O 17.910830 7.474000 3.191290
O 15.504330 6.146160 4.002710
O 15.504330 8.801840 4.002710
O 17.883172 11.787917 5.701389
O 17.881696 9.143499 5.695956
O 15.524541 10.467750 6.514948
O 15.334440 7.475316 6.898098
O 17.835734 10.458963 7.937398
O 17.727295 7.482352 8.299664
O 15.312507 6.118985 9.138160
O 15.304857 8.827853 9.138932
Mg 16.790480 13.453200 0.388370
Mg 14.255060 17.893540 2.261970
Mg 14.255060 14.992060 2.261970
Mg 16.592230 16.443610 4.522324
Mg 18.971572 13.454523 5.182464
Mg 16.629880 12.006800 7.439689
Mg 16.630649 14.902233 7.442218
Mg 14.434708 16.445681 9.280948
Si 16.974970 16.442800 1.221250
Si 16.265610 13.453200 3.313620
Si 18.632714 16.442076 6.397030

Si 14.603488 13.454566 8.469174

O 17.681710 17.805520 0.556660

O 17.681710 15.080070 0.556660

O 15.349310 16.442800 1.386100

O 15.470890 13.453200 1.760420

O 17.713840 16.442800 2.799080

O 17.910830 13.453200 3.191290

O 15.504330 12.125360 4.002710

O 15.504330 14.781030 4.002710

O 17.882646 17.764539 5.703368

O 17.881896 15.119341 5.703495

O 15.523182 16.443565 6.521043

O 15.339363 13.454424 6.902001

O 17.841155 16.443348 7.942754

O 17.736449 13.451344 8.307811

O 15.306203 12.098315 9.140739

O 15.306329 14.810483 9.140486

C 9.340401 4.437982 11.842196

C 9.801550 5.776305 11.962664

C 8.892628 6.859455 12.046656

C 7.511516 6.594889 12.025867

C 7.045943 5.232798 11.916922

C 7.945860 4.158527 11.788403

C 5.571971 5.251597 11.867165

C 4.574549 4.270336 11.737764

C 3.221853 4.703255 11.489195

C 2.868515 6.065150 11.440746

C 3.860770 7.078065 11.664634

C 5.187867 6.630497 11.871273

C 6.324802 7.486605 12.018819

C 6.094477 8.863941 12.080081

C 4.746999 9.353354 11.912683

C 3.654896 8.500977 11.663130

H 10.060223 3.608576 11.794986

H 10.876536 5.981463 11.959250

H 9.277562 7.883248 12.107233

H 7.579826 3.135737 11.629014

H 4.813648 3.200083 11.761474

H 2.462259 3.929464 11.302412

H 1.848422 6.356624 11.169179

H 6.905949 9.580904 12.251290

H 4.564119 10.431080 12.047693

H 2.649070 8.893610 11.461659

**Fluoranthene Fe-[010]-fo**

Number of atoms: 362

Mg 2.543480 1.494800 0.388370

Mg 0.008060 5.935140 2.261970

Mg 0.008060 3.033660 2.261970

Mg 2.343077 4.485690 4.520516

Mg 4.721748 1.496778 5.180648

Mg 2.379739 0.050279 7.433996

Mg 2.380891 2.948479 7.438183

Mg 0.180918 4.487586 9.281673

| | | | |
|---|---|---|---|
| Si | 2.727970 | 4.484400 | 1.221250 |
| Si | 2.018610 | 1.494800 | 3.313620 |
| Si | 4.383893 | 4.488237 | 6.393934 |
| Si | 0.357647 | 1.499571 | 8.466859 |
| O | 3.434710 | 5.847120 | 0.556660 |
| O | 3.434700 | 3.121670 | 0.556660 |
| O | 1.102310 | 4.484400 | 1.386100 |
| O | 1.223890 | 1.494800 | 1.760420 |
| O | 3.466830 | 4.484400 | 2.799080 |
| O | 3.663830 | 1.494800 | 3.191290 |
| O | 1.257330 | 0.166960 | 4.002710 |
| O | 1.257330 | 2.822640 | 4.002710 |
| O | 3.633191 | 5.810450 | 5.701575 |
| O | 3.633466 | 3.167558 | 5.698092 |
| O | 1.270379 | 4.489119 | 6.518931 |
| O | 1.092805 | 1.501121 | 6.899281 |
| O | 3.591465 | 4.488290 | 7.938175 |
| O | 3.484219 | 1.496829 | 8.303635 |
| O | 1.059480 | 0.143547 | 9.137585 |
| O | 1.058196 | 2.855385 | 9.139221 |
| Mg | 2.543480 | 7.474000 | 0.388370 |
| Mg | 0.008060 | 11.914340 | 2.261970 |
| Mg | 0.008060 | 9.012860 | 2.261970 |
| Mg | 2.341825 | 10.463440 | 4.521005 |
| Mg | 4.721002 | 7.475209 | 5.183122 |
| Mg | 2.378537 | 6.028536 | 7.443824 |
| Mg | 2.377692 | 8.925446 | 7.439755 |
| Mg | 0.187157 | 10.467825 | 9.283105 |
| Si | 2.727970 | 10.463600 | 1.221250 |
| Si | 2.018610 | 7.474000 | 3.313620 |
| Si | 4.384968 | 10.466617 | 6.395809 |
| Si | 0.352629 | 7.479664 | 8.472360 |
| O | 3.434710 | 11.826320 | 0.556660 |
| O | 3.434710 | 9.100870 | 0.556660 |
| O | 1.102310 | 10.463600 | 1.386100 |
| O | 1.223890 | 7.474000 | 1.760420 |
| O | 3.466830 | 10.463600 | 2.799080 |
| O | 3.663830 | 7.474000 | 3.191290 |
| O | 1.257330 | 6.146160 | 4.002710 |
| O | 1.257330 | 8.801840 | 4.002710 |
| O | 3.640938 | 11.789483 | 5.699512 |
| O | 3.639263 | 9.144477 | 5.700433 |
| O | 1.278691 | 10.465259 | 6.510929 |
| O | 1.091065 | 7.475547 | 6.904869 |
| O | 3.585963 | 10.468030 | 7.936416 |
| O | 3.483426 | 7.478113 | 8.314952 |
| O | 1.053459 | 6.121787 | 9.143194 |
| O | 1.060781 | 8.832833 | 9.143484 |
| Mg | 2.543480 | 13.453200 | 0.388370 |
| Mg | 0.008060 | 17.893540 | 2.261970 |
| Mg | 0.008060 | 14.992060 | 2.261970 |
| Mg | 2.342168 | 16.443596 | 4.517958 |
| Mg | 4.716826 | 13.460275 | 5.174414 |
| Mg | 2.376578 | 12.008421 | 7.433448 |

Mg 2.371080 14.908978 7.431411

Mg 0.185975 16.447175 9.280277

Si 2.727970 16.442800 1.221250

Si 2.018610 13.453200 3.313620

Si 4.382158 16.445595 6.389700

Si 0.357156 13.453887 8.471196

O 3.434710 17.805520 0.556660

O 3.434710 15.080070 0.556660

O 1.102310 16.442800 1.386100

O 1.223890 13.453200 1.760420

O 3.466830 16.442800 2.799080

O 3.663830 13.453200 3.191290

O 1.257330 12.125360 4.002710

O 1.257330 14.781030 4.002710

O 3.633036 17.767037 5.695672

O 3.631453 15.124299 5.695168

O 1.273290 16.446990 6.513282

O 1.089468 13.456821 6.900566

O 3.586457 16.442925 7.932923

O 3.475288 13.466966 8.300631

O 1.066180 12.099995 9.141907

O 1.057866 14.812796 9.140362

Mg 7.292480 1.494800 0.388370

Mg 4.757060 5.935140 2.261970

Mg 4.757060 3.033660 2.261970

Mg 7.095047 4.482917 4.521298

Mg 9.472424 1.486652 5.191938

Mg 7.142815 0.045440 7.454368

Mg 7.134585 2.942939 7.446064

Mg 4.927643 4.482668 9.286570

Si 7.476970 4.484400 1.221250

Si 6.767610 1.494800 3.313620

Si 9.131910 4.484688 6.397206

Si 5.105283 1.496758 8.469369

O 8.183700 5.847120 0.556660

O 8.183700 3.121670 0.556660

O 5.851310 4.484400 1.386100

O 5.972890 1.494800 1.760420

O 8.215830 4.484400 2.799080

O 8.412830 1.494800 3.191290

O 6.006330 0.166960 4.002710

O 6.006330 2.822640 4.002710

O 8.383463 5.809133 5.704054

O 8.383321 3.164203 5.700803

O 6.026299 4.487109 6.519375

O 5.844792 1.493237 6.905139

O 8.343262 4.487528 7.943679

O 8.230340 1.500310 8.321769

O 5.804733 0.143945 9.149660

O 5.804607 2.852797 9.142279

Mg 7.292480 7.474000 0.388370

Mg 4.757060 11.914340 2.261970

Mg 4.757060 9.012860 2.261970

Mg 7.104723 10.462178 4.530952

| | | | |
|---|---|---|---|
| Mg | 9.467587 | 7.480230 | 5.178819 |
| Mg | 7.127479 | 6.023531 | 7.445141 |
| Mg | 7.136475 | 8.924682 | 7.461242 |
| Mg | 4.928640 | 10.461953 | 9.270182 |
| Si | 7.476970 | 10.463600 | 1.221250 |
| Si | 6.767610 | 7.474000 | 3.313620 |
| Si | 9.157501 | 10.462618 | 6.420650 |
| Si | 5.103257 | 7.472126 | 8.484274 |
| O | 8.183700 | 11.826320 | 0.556660 |
| O | 8.183700 | 9.100870 | 0.556660 |
| O | 5.851310 | 10.463600 | 1.386100 |
| O | 5.972890 | 7.474000 | 1.760420 |
| O | 8.215830 | 10.463600 | 2.799080 |
| O | 8.412830 | 7.474000 | 3.191290 |
| O | 6.006330 | 6.146160 | 4.002710 |
| O | 6.006330 | 8.801840 | 4.002710 |
| O | 8.383944 | 11.776300 | 5.724822 |
| O | 8.382225 | 9.143283 | 5.736505 |
| O | 6.028476 | 10.466628 | 6.543655 |
| O | 5.841411 | 7.481976 | 6.917747 |
| O | 8.372574 | 10.465280 | 7.982589 |
| O | 8.229535 | 7.472435 | 8.323464 |
| O | 5.805148 | 6.114931 | 9.150579 |
| O | 5.805426 | 8.823147 | 9.176046 |
| Mg | 7.292480 | 13.453200 | 0.388370 |
| Mg | 4.757060 | 17.893540 | 2.261970 |
| Mg | 4.757060 | 14.992060 | 2.261970 |
| Mg | 7.105045 | 16.445667 | 4.529393 |
| Mg | 9.456429 | 13.455432 | 5.151471 |
| Mg | 7.132388 | 12.011522 | 7.440782 |
| Mg | 7.139603 | 14.910460 | 7.435981 |
| Mg | 4.925750 | 16.451526 | 9.271883 |
| Si | 7.476970 | 16.442800 | 1.221250 |
| Si | 6.767610 | 13.453200 | 3.313620 |
| Si | 9.145290 | 16.442275 | 6.415923 |
| Si | 5.092961 | 13.470721 | 8.461728 |
| O | 8.183700 | 17.805520 | 0.556660 |
| O | 8.183700 | 15.080070 | 0.556660 |
| O | 5.851310 | 16.442800 | 1.386100 |
| O | 5.972890 | 13.453200 | 1.760420 |
| O | 8.215830 | 16.442800 | 2.799080 |
| O | 8.412830 | 13.453200 | 3.191290 |
| O | 6.006330 | 12.125360 | 4.002710 |
| O | 6.006330 | 14.781030 | 4.002710 |
| O | 8.382597 | 17.762156 | 5.719092 |
| O | 8.382370 | 15.126665 | 5.708019 |
| O | 6.022929 | 16.444174 | 6.530918 |
| O | 5.837698 | 13.473111 | 6.905300 |
| O | 8.355523 | 16.436113 | 7.961223 |
| O | 8.239556 | 13.455384 | 8.282745 |
| O | 5.787939 | 12.109276 | 9.133469 |
| O | 5.802944 | 14.807735 | 9.181246 |
| Mg | 12.041480 | 1.494800 | 0.388370 |
| Mg | 9.506060 | 5.935140 | 2.261970 |

| | | | |
|---|---|---|---|
| Mg | 9.506060 | 3.033660 | 2.261970 |
| Mg | 11.845036 | 4.482797 | 4.520340 |
| Mg | 14.214351 | 1.492108 | 5.180284 |
| Mg | 11.868300 | 0.019436 | 7.457094 |
| Mg | 11.878413 | 2.924916 | 7.454052 |
| Mg | 9.696672 | 4.466213 | 9.276277 |
| Si | 12.225970 | 4.484400 | 1.221250 |
| Si | 11.516610 | 1.494800 | 3.313620 |
| Si | 13.876460 | 4.478489 | 6.398929 |
| Si | 9.850360 | 1.463337 | 8.495266 |
| O | 12.932710 | 5.847120 | 0.556660 |
| O | 12.932710 | 3.121670 | 0.556660 |
| O | 10.600310 | 4.484400 | 1.386100 |
| O | 10.721890 | 1.494800 | 1.760420 |
| O | 12.964830 | 4.484400 | 2.799080 |
| O | 13.161830 | 1.494800 | 3.191290 |
| O | 10.755330 | 0.166960 | 4.002710 |
| O | 10.755330 | 2.822640 | 4.002710 |
| O | 13.126929 | 5.801350 | 5.703569 |
| O | 13.125591 | 3.157913 | 5.703369 |
| O | 10.771583 | 4.481895 | 6.535719 |
| O | 10.587737 | 1.477347 | 6.925247 |
| O | 13.085583 | 4.475968 | 7.945319 |
| O | 12.978115 | 1.475572 | 8.322110 |
| O | 10.514262 | 0.083050 | 9.154862 |
| O | 10.557593 | 2.822761 | 9.166082 |
| Mg | 12.041480 | 7.474000 | 0.388370 |
| Mg | 9.506060 | 11.914340 | 2.261970 |
| Mg | 9.506060 | 9.012860 | 2.261970 |
| Mg | 11.852037 | 10.461921 | 4.523881 |
| Mg | 14.220462 | 7.475150 | 5.183190 |
| Mg | 11.887218 | 6.025194 | 7.444511 |
| Mg | 11.890612 | 8.934713 | 7.423773 |
| Fe | 9.601609 | 10.461558 | 9.548987 |
| Si | 12.225970 | 10.463600 | 1.221250 |
| Si | 11.516610 | 7.474000 | 3.313620 |
| Si | 13.901487 | 10.455762 | 6.405617 |
| Si | 9.846896 | 7.461904 | 8.468279 |
| O | 12.932710 | 11.826320 | 0.556660 |
| O | 12.932710 | 9.100870 | 0.556660 |
| O | 10.600310 | 10.463600 | 1.386100 |
| O | 10.721890 | 7.474000 | 1.760420 |
| O | 12.964840 | 10.463600 | 2.799080 |
| O | 13.161830 | 7.474000 | 3.191290 |
| O | 10.755330 | 6.146160 | 4.002710 |
| O | 10.755330 | 8.801840 | 4.002710 |
| O | 13.148160 | 11.771562 | 5.688942 |
| O | 13.141309 | 9.136071 | 5.703239 |
| O | 10.795911 | 10.443610 | 6.467881 |
| O | 10.588315 | 7.478674 | 6.915241 |
| O | 13.101146 | 10.462349 | 7.934802 |
| O | 12.963262 | 7.477666 | 8.324525 |
| O | 10.549686 | 6.121165 | 9.159881 |
| O | 10.540491 | 8.844179 | 9.152261 |

Mg 12.041480 13.453200 0.388370

Mg 9.506060 17.893540 2.261970

Mg 9.506060 14.992060 2.261970

Mg 11.839101 16.443423 4.522502

Mg 14.220626 13.446460 5.175569

Mg 11.890534 11.987589 7.383615

Mg 11.890546 14.898808 7.401297

Mg 9.630724 16.370682 9.402503

Si 12.225970 16.442800 1.221250

Si 11.516610 13.453200 3.313620

Si 13.887087 16.444706 6.384228

Si 9.865922 13.452862 8.365339

O 12.932710 17.805520 0.556660

O 12.932710 15.080070 0.556660

O 10.600310 16.442800 1.386100

O 10.721890 13.453200 1.760420

O 12.964840 16.442800 2.799080

O 13.161830 13.453200 3.191290

O 10.755330 12.125360 4.002710

O 10.755330 14.781030 4.002710

O 13.133862 17.766944 5.697913

O 13.141773 15.123989 5.683494

O 10.787612 16.444977 6.480398

O 10.607285 13.446879 6.814398

O 13.098926 16.433803 7.929006

O 12.978282 13.439858 8.279377

O 10.552632 12.057065 8.986375

O 10.582692 14.800377 9.020170

Mg 16.790480 1.494800 0.388370

Mg 14.255060 5.935140 2.261970

Mg 14.255060 3.033660 2.261970

Mg 16.590894 4.486464 4.521663

Mg 18.962420 1.499199 5.177983

Mg 16.629665 0.047443 7.439234

Mg 16.623867 2.946116 7.441704

Mg 14.437657 4.491809 9.280052

Si 16.974970 4.484400 1.221250

Si 16.265610 1.494800 3.313620

Si 18.624159 4.491844 6.395913

Si 14.600656 1.488991 8.475634

O 17.681710 5.847120 0.556660

O 17.681710 3.121670 0.556660

O 15.349310 4.484400 1.386100

O 15.470890 1.494800 1.760420

O 17.713830 4.484400 2.799080

O 17.910830 1.494800 3.191290

O 15.504330 0.166960 4.002710

O 15.504330 2.822640 4.002710

O 17.873386 5.812263 5.701234

O 17.875849 3.169749 5.699071

O 15.518216 4.487095 6.525589

O 15.334557 1.495303 6.906650

O 17.832349 4.491098 7.941338

O 17.733573 1.498936 8.304461

O 15.310400 0.135000 9.144608
O 15.299197 2.849891 9.143213
Mg 16.790480 7.474000 0.388370
Mg 14.255060 11.914340 2.261970
Mg 14.255060 9.012860 2.261970
Mg 16.587151 10.463679 4.528498
Mg 18.961502 7.478831 5.180681
Mg 16.620318 6.038976 7.449149
Mg 16.608013 8.943912 7.461444
Mg 14.312624 10.484537 9.427321
Si 16.974970 10.463600 1.221250
Si 16.265610 7.474000 3.313620
Si 18.630177 10.464128 6.392576
Si 14.589719 7.498548 8.482347
O 17.681710 11.826320 0.556660
O 17.681710 9.100870 0.556660
O 15.349310 10.463600 1.386100
O 15.470890 7.474000 1.760420
O 17.713840 10.463600 2.799080
O 17.910830 7.474000 3.191290
O 15.504330 6.146160 4.002710
O 15.504330 8.801840 4.002710
O 17.883917 11.788622 5.700896
O 17.882855 9.141732 5.700471
O 15.544375 10.460289 6.477959
O 15.327804 7.480573 6.913614
O 17.847917 10.465445 7.939008
O 17.727944 7.485436 8.315000
O 15.292225 6.136632 9.153386
O 15.266655 8.869688 9.136237
Mg 16.790480 13.453200 0.388370
Mg 14.255060 17.893540 2.261970
Mg 14.255060 14.992060 2.261970
Mg 16.590563 16.440365 4.521421
Mg 18.960987 13.450841 5.177981
Mg 16.616691 11.991642 7.445862
Mg 16.631528 14.893808 7.441714
Mg 14.446030 16.430302 9.261351
Si 16.974970 16.442800 1.221250
Si 16.265610 13.453200 3.313620
Si 18.627536 16.442595 6.392195
Si 14.599265 13.432149 8.450666
O 17.681710 17.805520 0.556660
O 17.681710 15.080070 0.556660
O 15.349310 16.442800 1.386100
O 15.470890 13.453200 1.760420
O 17.713840 16.442800 2.799080
O 17.910830 13.453200 3.191290
O 15.504330 12.125360 4.002710
O 15.504330 14.781030 4.002710
O 17.879456 17.765995 5.698946
O 17.880474 15.119905 5.699243
O 15.529156 16.443889 6.519877
O 15.345567 13.460360 6.887024

O 17.839207 16.441746 7.939287

O 17.734010 13.448180 8.312927

O 15.298539 12.063292 9.088453

O 15.296619 14.783191 9.150647

C 13.658217 10.270085 11.754571

C 14.133409 11.606600 11.878233

C 13.231362 12.695562 11.910910

C 11.848294 12.442482 11.856338

C 11.364798 11.081885 11.741138

C 12.265186 10.001270 11.654619

C 9.889248 11.129209 11.656572

C 8.833163 10.162949 11.557737

C 7.467557 10.658044 11.596425

C 7.153375 12.014460 11.631513

C 8.201075 13.012533 11.677209

C 9.530633 12.526816 11.736142

C 10.687149 13.350797 11.869779

C 10.502595 14.741580 11.978769

C 9.174134 15.267196 11.876445

C 8.041275 14.431831 11.705500

H 14.369520 9.432231 11.774088

H 15.209480 11.801829 11.934227

H 13.617846 13.717807 11.985518

H 11.905184 8.977898 11.493049

H 9.017112 9.090309 11.692879

H 6.676289 9.896639 11.561266

H 6.110182 12.341662 11.631948

H 11.344875 15.422411 12.147100

H 9.013515 16.342159 12.056507

H 7.037722 14.860453 11.600912

**Fluoranthene Ni-[010]-fo**

Number of atoms: 362

Mg 2.543480 1.494800 0.388370

Mg 0.008060 5.935140 2.261970

Mg 0.008060 3.033660 2.261970

Mg 2.342760 4.484586 4.519390

Mg 4.718520 1.493645 5.179988

Mg 2.379043 0.045124 7.442197

Mg 2.378638 2.943446 7.441660

Mg 0.178007 4.484335 9.281929

Si 2.727970 4.484400 1.221250

Si 2.018610 1.494800 3.313620

Si 4.381631 4.483105 6.393701

Si 0.354303 1.493613 8.470454

O 3.434710 5.847120 0.556660

O 3.434700 3.121670 0.556660

O 1.102310 4.484400 1.386100

O 1.223890 1.494800 1.760420

O 3.466830 4.484400 2.799080

O 3.663830 1.494800 3.191290

O 1.257330 0.166960 4.002710

O 1.257330 2.822640 4.002710

O 3.631388 5.803749 5.697952

O 3.631605 3.161481 5.700071

O 1.270140 4.481290 6.519381

O 1.091654 1.494121 6.903662

O 3.590596 4.485243 7.938409

O 3.482774 1.495008 8.310319

O 1.055105 0.137672 9.142276

O 1.052837 2.850506 9.142887

Mg 2.543480 7.474000 0.388370

Mg 0.008060 11.914340 2.261970

Mg 0.008060 9.012860 2.261970

Mg 2.340322 10.463795 4.519450

Mg 4.718270 7.472670 5.179364

Mg 2.372956 6.021112 7.437708

Mg 2.372671 8.921189 7.432417

Mg 0.183530 10.461533 9.280959

Si 2.727970 10.463600 1.221250

Si 2.018610 7.474000 3.313620

Si 4.380607 10.464005 6.392435

Si 0.351488 7.475136 8.468714

O 3.434710 11.826320 0.556660

O 3.434710 9.100870 0.556660

O 1.102310 10.463600 1.386100

O 1.223890 7.474000 1.760420

O 3.466830 10.463600 2.799080

O 3.663830 7.474000 3.191290

O 1.257330 6.146160 4.002710

O 1.257330 8.801840 4.002710

O 3.636026 11.787763 5.698532

O 3.635582 9.141675 5.696047

O 1.274360 10.464211 6.509427

O 1.087777 7.469716 6.900522

O 3.577901 10.463504 7.931524

O 3.474808 7.468095 8.307195

O 1.051532 6.117401 9.140141

O 1.059823 8.828063 9.139615

Mg 2.543480 13.453200 0.388370

Mg 0.008060 17.893540 2.261970

Mg 0.008060 14.992060 2.261970

Mg 2.342897 16.441877 4.520758

Mg 4.719995 13.454111 5.181505

Mg 2.372956 12.005627 7.435277

Mg 2.372911 14.903451 7.441743

Mg 0.179453 16.441841 9.283251

Si 2.727970 16.442800 1.221250

Si 2.018610 13.453200 3.313620

Si 4.381471 16.441366 6.396564

Si 0.351856 13.449099 8.473574

O 3.434710 17.805520 0.556660

O 3.434710 15.080070 0.556660

O 1.102310 16.442800 1.386100

O 1.223890 13.453200 1.760420

O 3.466830 16.442800 2.799080

O 3.663830 13.453200 3.191290

O 1.257330 12.125360 4.002710

O 1.257330 14.781030 4.002710

O 3.631947 17.763481 5.702644

O 3.631783 15.119814 5.701658

O 1.270431 16.441839 6.519875

O 1.087195 13.455208 6.904342

O 3.589953 16.440064 7.941672

O 3.475238 13.457786 8.311941

O 1.060549 12.094738 9.142522

O 1.051437 14.807637 9.144927

Mg 7.292480 1.494800 0.388370

Mg 4.757060 5.935140 2.261970

Mg 4.757060 3.033660 2.261970

Mg 7.094662 4.484508 4.519897

Mg 9.469623 1.493203 5.182215

Mg 7.134221 0.045354 7.446967

Mg 7.134378 2.943459 7.445493

Mg 4.930565 4.481783 9.282814

Si 7.476970 4.484400 1.221250

Si 6.767610 1.494800 3.313620

Si 9.129803 4.488121 6.391934

Si 5.104187 1.495505 8.470485

O 8.183700 5.847120 0.556660

O 8.183700 3.121670 0.556660

O 5.851310 4.484400 1.386100

O 5.972890 1.494800 1.760420

O 8.215830 4.484400 2.799080

O 8.412830 1.494800 3.191290

O 6.006330 0.166960 4.002710

O 6.006330 2.822640 4.002710

O 8.376776 5.806228 5.695400

O 8.382029 3.164073 5.699407

O 6.023468 4.485549 6.524797

O 5.842268 1.494798 6.904251

O 8.341094 4.489097 7.938872

O 8.237900 1.494626 8.310331

O 5.805851 0.140672 9.143987

O 5.806729 2.850553 9.142786

Mg 7.292480 7.474000 0.388370

Mg 4.757060 11.914340 2.261970

Mg 4.757060 9.012860 2.261970

Mg 7.111968 10.463848 4.535982

Mg 9.465452 7.482258 5.176301

Mg 7.129203 6.025020 7.441537

Mg 7.135979 8.922160 7.456832

Mg 4.920511 10.461258 9.261205

Si 7.476970 10.463600 1.221250

Si 6.767610 7.474000 3.313620

Si 9.156828 10.463858 6.435065

Si 5.093546 7.464681 8.478267

O 8.183700 11.826320 0.556660

O 8.183700 9.100870 0.556660

O 5.851310 10.463600 1.386100

O 5.972890 7.474000 1.760420

O 8.215830 10.463600 2.799080

O 8.412830 7.474000 3.191290

O 6.006330 6.146160 4.002710

O 6.006330 8.801840 4.002710

O 8.382183 11.780726 5.733705

O 8.382162 9.147606 5.732807

O 6.021817 10.464271 6.548623

O 5.834234 7.471830 6.916422

O 8.361057 10.464242 7.978285

O 8.222036 7.467849 8.316315

O 5.803690 6.120786 9.174727

O 5.788511 8.818520 9.168620

Mg 7.292480 13.453200 0.388370

Mg 4.757060 17.893540 2.261970

Mg 4.757060 14.992060 2.261970

Mg 7.094837 16.441815 4.520433

Mg 9.465594 13.443094 5.175781

Mg 7.136453 12.005056 7.458655

Mg 7.130570 14.900095 7.446031

Mg 4.931939 16.445120 9.283987

Si 7.476970 16.442800 1.221250

Si 6.767610 13.453200 3.313620

Si 9.129327 16.435086 6.393556

Si 5.093776 13.460102 8.481218

O 8.183700 17.805520 0.556660

O 8.183700 15.080070 0.556660

O 5.851310 16.442800 1.386100

O 5.972890 13.453200 1.760420

O 8.215830 16.442800 2.799080

O 8.412830 13.453200 3.191290

O 6.006330 12.125360 4.002710

O 6.006330 14.781030 4.002710

O 8.382305 17.759815 5.701320

O 8.376503 15.115070 5.699703

O 6.023656 16.439499 6.527253

O 5.835498 13.454827 6.920270

O 8.341731 16.436278 7.941895

O 8.223732 13.457249 8.322174

O 5.788077 12.104134 9.168944

O 5.804634 14.804921 9.177963

Mg 12.041480 1.494800 0.388370

Mg 9.506060 5.935140 2.261970

Mg 9.506060 3.033660 2.261970

Mg 11.844910 4.487613 4.519183

Mg 14.214982 1.492576 5.179917

Mg 11.882734 0.043807 7.443445

Mg 11.882630 2.941909 7.441937

Mg 9.706703 4.504137 9.256238

Si 12.225970 4.484400 1.221250

Si 11.516610 1.494800 3.313620

Si 13.878772 4.486599 6.393849

Si 9.858248 1.493644 8.476244

O 12.932710 5.847120 0.556660

O 12.932710 3.121670 0.556660

O 10.600310 4.484400 1.386100

O 10.721890 1.494800 1.760420

O 12.964830 4.484400 2.799080

O 13.161830 1.494800 3.191290

O 10.755330 0.166960 4.002710

O 10.755330 2.822640 4.002710

O 13.127807 5.805923 5.698339

O 13.127756 3.163313 5.700588

O 10.768657 4.487938 6.538810

O 10.592270 1.492978 6.907072

O 13.087695 4.486834 7.940958

O 12.980400 1.492985 8.312792

O 10.556937 0.134006 9.146383

O 10.557414 2.853767 9.145195

Mg 12.041480 7.474000 0.388370

Mg 9.506060 11.914340 2.261970

Mg 9.506060 9.012860 2.261970

Mg 11.846843 10.463278 4.530134

Mg 14.215128 7.483065 5.179869

Mg 11.888623 6.037759 7.445837

Mg 11.867022 8.947118 7.425231

Ni 9.525006 10.467180 9.554653

Si 12.225970 10.463600 1.221250

Si 11.516610 7.474000 3.313620

Si 13.893405 10.462264 6.410670

Si 9.844666 7.501675 8.451176

O 12.932710 11.826320 0.556660

O 12.932710 9.100870 0.556660

O 10.600310 10.463600 1.386100

O 10.721890 7.474000 1.760420

O 12.964840 10.463600 2.799080

O 13.161830 7.474000 3.191290

O 10.755330 6.146160 4.002710

O 10.755330 8.801840 4.002710

O 13.138564 11.782258 5.702394

O 13.138071 9.142589 5.703347

O 10.796170 10.463336 6.446231

O 10.586868 7.453883 6.892381

O 13.100866 10.462583 7.939461

O 12.967011 7.485710 8.323086

O 10.546374 6.167880 9.188007

O 10.539165 8.903002 9.038114

Mg 12.041480 13.453200 0.388370

Mg 9.506060 17.893540 2.261970

Mg 9.506060 14.992060 2.261970

Mg 11.844512 16.438600 4.520084

Mg 14.216040 13.440659 5.179974

Mg 11.868160 11.977659 7.423109

Mg 11.889154 14.885450 7.445195

Mg 9.708456 16.420223 9.257416

Si 12.225970 16.442800 1.221250

Si 11.516610 13.453200 3.313620

Si 13.878786 16.435029 6.395231

Si 9.846910 13.423927 8.451285

O 12.932710 17.805520 0.556660

O 12.932710 15.080070 0.556660
O 10.600310 16.442800 1.386100
O 10.721890 13.453200 1.760420
O 12.964840 16.442800 2.799080
O 13.161830 13.453200 3.191290
O 10.755330 12.125360 4.002710
O 10.755330 14.781030 4.002710
O 13.128010 17.759149 5.702706
O 13.127762 15.115278 5.700431
O 10.768501 16.435875 6.538653
O 10.586058 13.471151 6.891065
O 13.087372 16.435266 7.942548
O 12.968398 13.436965 8.321619
O 10.545934 12.025245 9.038664
O 10.549908 14.757134 9.187675
Mg 16.790480 1.494800 0.388370
Mg 14.255060 5.935140 2.261970
Mg 14.255060 3.033660 2.261970
Mg 16.590782 4.486445 4.520938
Mg 18.963642 1.492753 5.180043
Mg 16.626255 0.042504 7.440436
Mg 16.625890 2.941744 7.440097
Mg 14.441886 4.491975 9.272649
Si 16.974970 4.484400 1.221250
Si 16.265610 1.494800 3.313620
Si 18.624337 4.484557 6.394574
Si 14.601747 1.492652 8.473579
O 17.681710 5.847120 0.556660
O 17.681710 3.121670 0.556660
O 15.349310 4.484400 1.386100
O 15.470890 1.494800 1.760420
O 17.713830 4.484400 2.799080
O 17.910830 1.494800 3.191290
O 15.504330 0.166960 4.002710
O 15.504330 2.822640 4.002710
O 17.875480 5.804346 5.697540
O 17.875726 3.161225 5.700429
O 15.519963 4.483878 6.524208
O 15.335114 1.492522 6.904851
O 17.831987 4.486485 7.939662
O 17.730384 1.492053 8.306924
O 15.302617 0.134797 9.142689
O 15.303347 2.849983 9.143091
Mg 16.790480 7.474000 0.388370
Mg 14.255060 11.914340 2.261970
Mg 14.255060 9.012860 2.261970
Mg 16.583916 10.463864 4.528842
Mg 18.958740 7.476891 5.177961
Mg 16.622127 6.034656 7.443761
Mg 16.607255 8.939230 7.452381
Mg 14.309550 10.461846 9.428339
Si 16.974970 10.463600 1.221250
Si 16.265610 7.474000 3.313620
Si 18.626258 10.464193 6.391015

Si 14.592998 7.496318 8.470226
O 17.681710 11.826320 0.556660
O 17.681710 9.100870 0.556660
O 15.349310 10.463600 1.386100
O 15.470890 7.474000 1.760420
O 17.713840 10.463600 2.799080
O 17.910830 7.474000 3.191290
O 15.504330 6.146160 4.002710
O 15.504330 8.801840 4.002710
O 17.879821 11.788989 5.701523
O 17.880228 9.141440 5.697394
O 15.538727 10.462605 6.478345
O 15.329645 7.472192 6.902026
O 17.844515 10.461665 7.937239
O 17.727869 7.480284 8.309184
O 15.294544 6.136318 9.148615
O 15.285380 8.864180 9.116918
Mg 16.790480 13.453200 0.388370
Mg 14.255060 17.893540 2.261970
Mg 14.255060 14.992060 2.261970
Mg 16.591075 16.439216 4.521027
Mg 18.960724 13.448625 5.179082
Mg 16.608623 11.984069 7.453390
Mg 16.623728 14.886160 7.446106
Mg 14.443147 16.429168 9.272831
Si 16.974970 16.442800 1.221250
Si 16.265610 13.453200 3.313620
Si 18.624470 16.435750 6.396239
Si 14.594146 13.425917 8.468885
O 17.681710 17.805520 0.556660
O 17.681710 15.080070 0.556660
O 15.349310 16.442800 1.386100
O 15.470890 13.453200 1.760420
O 17.713840 16.442800 2.799080
O 17.910830 13.453200 3.191290
O 15.504330 12.125360 4.002710
O 15.504330 14.781030 4.002710
O 17.877171 17.759901 5.701745
O 17.875674 15.114782 5.701757
O 15.520172 16.437726 6.525039
O 15.331313 13.450319 6.901048
O 17.832740 16.435337 7.941768
O 17.728135 13.441501 8.314947
O 15.287219 12.058018 9.114289
O 15.295299 14.783990 9.150489
C 13.863123 9.789634 11.801793
C 13.853659 11.213751 11.796609
C 12.628753 11.931051 11.797095
C 11.426234 11.208639 11.879630
C 11.435807 9.762359 11.882447
C 12.647992 9.055571 11.805365
C 10.036805 9.288458 11.882553
C 9.399319 8.040562 11.833981
C 7.970787 8.004160 11.774308

C 7.178376 9.163780 11.804537
C 7.789690 10.460802 11.835145
C 9.219661 10.470704 11.850432
C 10.020870 11.664261 11.878766
C 9.366835 12.903430 11.825470
C 7.937898 12.919857 11.765660
C 7.161813 11.749575 11.801602
H 14.817388 9.248112 11.793850
H 14.800688 11.767218 11.779186
H 12.623591 13.024013 11.731211
H 12.658086 7.962116 11.747951
H 9.977237 7.110195 11.796880
H 7.463358 7.037827 11.669480
H 6.088841 9.060235 11.772175
H 9.932721 13.841053 11.785456
H 7.416753 13.878226 11.654573
H 6.070659 11.837994 11.771698

**Fluoranthene $V_{MgO}$-[010]-fo (=)**

Number of atoms: 360

Mg 2.543480 1.494800 0.388370
Mg 0.008060 5.935140 2.261970
Mg 0.008060 3.033660 2.261970
Mg 2.347348 4.483226 4.513374
Mg 4.717318 1.481609 5.177876
Mg 2.385189 0.036372 7.435543
Mg 2.383931 2.932471 7.429181
Mg 0.176683 4.474889 9.278484
Si 2.727970 4.484400 1.221250
Si 2.018610 1.494800 3.313620
Si 4.380690 4.471476 6.380917
Si 0.356810 1.490715 8.460272
O 3.434710 5.847120 0.556660
O 3.434700 3.121670 0.556660
O 1.102310 4.484400 1.386100
O 1.223890 1.494800 1.760420
O 3.466830 4.484400 2.799080
O 3.663830 1.494800 3.191290
O 1.257330 0.166960 4.002710
O 1.257330 2.822640 4.002710
O 3.632746 5.788182 5.679211
O 3.631017 3.145560 5.693177
O 1.278556 4.476264 6.501530
O 1.093042 1.486305 6.894075
O 3.586043 4.473856 7.922238
O 3.482056 1.485239 8.306031
O 1.053549 0.135772 9.134969
O 1.058185 2.847375 9.129069
Mg 2.543480 7.474000 0.388370
Mg 0.008060 11.914340 2.261970
Mg 0.008060 9.012860 2.261970
Mg 2.371610 10.464545 4.547151
Mg 4.716347 7.484905 5.175985
Mg 2.368036 6.014682 7.418305

Mg 2.388201 8.921023 7.457070
Mg 0.183364 10.458825 9.276275
Si 2.727970 10.463600 1.221250
Si 2.018610 7.474000 3.313620
Si 4.406012 10.453744 6.464575
Si 0.346835 7.470576 8.464652
O 3.434710 11.826320 0.556660
O 3.434710 9.100870 0.556660
O 1.102310 10.463600 1.386100
O 1.223890 7.474000 1.760420
O 3.466830 10.463600 2.799080
O 3.663830 7.474000 3.191290
O 1.257330 6.146160 4.002710
O 1.257330 8.801840 4.002710
O 3.642085 11.768987 5.745162
O 3.637371 9.142426 5.742327
O 1.278431 10.460907 6.526343
O 1.079051 7.473428 6.899520
O 3.606920 10.456639 8.008094
O 3.460215 7.437499 8.279179
O 1.040353 6.109374 9.131714
O 1.047121 8.818910 9.149133
Mg 2.543480 13.453200 0.388370
Mg 0.008060 17.893540 2.261970
Mg 0.008060 14.992060 2.261970
Mg 2.349958 16.437838 4.518725
Mg 4.734753 13.420497 5.198088
Mg 2.397298 11.994919 7.465497
Mg 2.382327 14.890024 7.433429
Mg 0.182316 16.438994 9.278618
Si 2.727970 16.442800 1.221250
Si 2.018610 13.453200 3.313620
Si 4.386960 16.425616 6.391246
Si 0.354863 13.446026 8.470860
O 3.434710 17.805520 0.556660
O 3.434710 15.080070 0.556660
O 1.102310 16.442800 1.386100
O 1.223890 13.453200 1.760420
O 3.466830 16.442800 2.799080
O 3.663830 13.453200 3.191290
O 1.257330 12.125360 4.002710
O 1.257330 14.781030 4.002710
O 3.637583 17.750076 5.696198
O 3.638652 15.105291 5.696991
O 1.279837 16.437984 6.506829
O 1.088794 13.440032 6.905614
O 3.592877 16.433972 7.934598
O 3.488443 13.464319 8.298282
O 1.052925 12.095564 9.155060
O 1.052945 14.806868 9.137900
Mg 7.292480 1.494800 0.388370
Mg 4.757060 5.935140 2.261970
Mg 4.757060 3.033660 2.261970
Mg 7.088377 4.486166 4.517316

| | | | |
|---|---|---|---|
| Mg | 9.464500 | 1.485637 | 5.174564 |
| Mg | 7.128005 | 0.021082 | 7.449238 |
| Mg | 7.123628 | 2.919999 | 7.432304 |
| Mg | 4.915851 | 4.470364 | 9.273368 |
| Si | 7.476970 | 4.484400 | 1.221250 |
| Si | 6.767610 | 1.494800 | 3.313620 |
| Si | 9.125343 | 4.480516 | 6.405524 |
| Si | 5.102251 | 1.476694 | 8.470976 |
| O | 8.183700 | 5.847120 | 0.556660 |
| O | 8.183700 | 3.121670 | 0.556660 |
| O | 5.851310 | 4.484400 | 1.386100 |
| O | 5.972890 | 1.494800 | 1.760420 |
| O | 8.215830 | 4.484400 | 2.799080 |
| O | 8.412830 | 1.494800 | 3.191290 |
| O | 6.006330 | 0.166960 | 4.002710 |
| O | 6.006330 | 2.822640 | 4.002710 |
| O | 8.393153 | 5.814400 | 5.721974 |
| O | 8.388530 | 3.163159 | 5.694198 |
| O | 6.024657 | 4.457420 | 6.507800 |
| O | 5.841125 | 1.471231 | 6.903486 |
| O | 8.326837 | 4.457713 | 7.944700 |
| O | 8.239133 | 1.474636 | 8.304001 |
| O | 5.801259 | 0.119439 | 9.144206 |
| O | 5.802576 | 2.838731 | 9.135960 |
| Mg | 7.292480 | 7.474000 | 0.388370 |
| Mg | 4.757060 | 11.914340 | 2.261970 |
| Mg | 4.757060 | 9.012860 | 2.261970 |

| | | | |
|---|---|---|---|
| Mg | 7.118151 | 10.463106 | 4.570285 |
| Mg | 9.427862 | 7.500205 | 5.179602 |
| Mg | 7.103639 | 5.991870 | 7.439726 |
| Mg | 7.145503 | 8.923964 | 7.468302 |
| Mg | 4.904653 | 10.443710 | 9.479141 |
| Si | 7.476970 | 10.463600 | 1.221250 |
| Si | 6.767610 | 7.474000 | 3.313620 |
| Si | 9.153751 | 10.428369 | 6.488826 |
| Si | 5.081062 | 7.460759 | 8.413222 |
| O | 8.183700 | 11.826320 | 0.556660 |
| O | 8.183700 | 9.100870 | 0.556660 |
| O | 5.851310 | 10.463600 | 1.386100 |
| O | 5.972890 | 7.474000 | 1.760420 |
| O | 8.215830 | 10.463600 | 2.799080 |
| O | 8.412830 | 7.474000 | 3.191290 |
| O | 6.006330 | 6.146160 | 4.002710 |
| O | 6.006330 | 8.801840 | 4.002710 |
| O | 8.416365 | 11.760186 | 5.740889 |
| O | 8.399628 | 9.128953 | 5.700511 |
| O | 6.046863 | 10.448931 | 6.496855 |
| O | 5.847982 | 7.425896 | 6.867664 |
| O | 8.424644 | 10.387954 | 7.976916 |
| O | 8.150489 | 7.392025 | 8.408491 |
| O | 5.773906 | 6.115320 | 9.149542 |
| O | 5.755201 | 8.840334 | 9.012704 |
| Mg | 7.292480 | 13.453200 | 0.388370 |
| Mg | 4.757060 | 17.893540 | 2.261970 |

Mg 4.757060 14.992060 2.261970
Mg 7.090157 16.433618 4.521332
Mg 9.445589 13.406898 5.178156
Mg 7.171909 11.922943 7.521746
Mg 7.134254 14.871291 7.445796
Mg 4.938054 16.410931 9.273762
Si 7.476970 16.442800 1.221250
Si 6.767610 13.453200 3.313620
Si 9.131898 16.421770 6.395598
Si 5.111673 13.422621 8.447531
O 8.183700 17.805520 0.556660
O 8.183700 15.080070 0.556660
O 5.851310 16.442800 1.386100
O 5.972890 13.453200 1.760420
O 8.215830 16.442800 2.799080
O 8.412830 13.453200 3.191290
O 6.006330 12.125360 4.002710
O 6.006330 14.781030 4.002710
O 8.387531 17.748086 5.705685
O 8.390121 15.096292 5.708366
O 6.030624 16.427077 6.520317
O 5.876316 13.425819 6.897683
O 8.348669 16.416586 7.945184
O 8.237791 13.446099 8.369898
O 5.784495 12.056571 9.090863
O 5.817013 14.778174 9.146047
Mg 12.041480 1.494800 0.388370
Mg 9.506060 5.935140 2.261970
Mg 9.506060 3.033660 2.261970
Mg 11.840584 4.488313 4.523008
Mg 14.212185 1.511982 5.179056
Mg 11.885936 0.039266 7.430829
Mg 11.874653 2.948678 7.448553
Mg 9.651925 4.423289 9.298745
Si 12.225970 4.484400 1.221250
Si 11.516610 1.494800 3.313620
Si 13.858486 4.518966 6.416423
Si 9.859320 1.468107 8.462989
O 12.932710 5.847120 0.556660
O 12.932710 3.121670 0.556660
O 10.600310 4.484400 1.386100
O 10.721890 1.494800 1.760420
O 12.964830 4.484400 2.799080
O 13.161830 1.494800 3.191290
O 10.755330 0.166960 4.002710
O 10.755330 2.822640 4.002710
O 13.096205 5.846901 5.740782
O 13.120007 3.194251 5.709591
O 10.764771 4.515595 6.550872
O 10.595125 1.491868 6.896157
O 13.071981 4.501929 7.965328
O 12.976313 1.495252 8.307755
O 10.565786 0.110004 9.125345
O 10.560341 2.816314 9.149720

Mg 12.041480 7.474000 0.388370

Mg 9.506060 11.914340 2.261970

Mg 9.506060 9.012860 2.261970

Mg 11.822789 10.473329 4.512741

Mg 14.227815 7.465769 5.160372

Mg 11.827343 6.044416 7.530696

Mg 11.941360 8.930219 7.278883

Si 12.225409 10.459321 1.229244

Si 11.516610 7.474000 3.313620

Si 13.888210 10.463600 6.382630

Si 9.743703 7.448573 8.686352

O 12.928055 11.816447 0.557552

O 12.932710 9.100870 0.556660

O 10.600310 10.463600 1.386100

O 10.721890 7.474000 1.760420

O 12.964840 10.463600 2.799080

O 13.161830 7.474000 3.191290

O 10.755330 6.146160 4.002710

O 10.755330 8.801840 4.002710

O 13.128110 11.791550 5.692940

O 13.159153 9.140825 5.651588

O 10.820562 10.438155 6.452390

O 10.522247 7.613659 7.157571

O 13.095345 10.436632 7.898663

O 12.951535 7.513980 8.343331

O 10.540924 6.059591 9.214499

Mg 12.055796 13.450986 0.411197

Mg 9.504508 17.892502 2.255634

Mg 9.506060 14.992060 2.261970

Mg 11.837740 16.442800 4.520030

Mg 14.211300 13.453200 5.174280

Mg 11.886778 11.959384 7.384612

Mg 11.899567 14.881026 7.434723

Mg 9.699788 16.411251 9.277185

Si 12.223689 16.442025 1.231081

Si 11.510820 13.454116 3.310451

Si 13.888220 16.442800 6.382630

Si 9.847270 13.453200 8.472360

O 12.921274 17.801186 0.558789

O 12.927495 15.086221 0.560156

O 10.600310 16.442800 1.386100

O 10.721890 13.453200 1.760420

O 12.964840 16.442800 2.799080

O 13.161830 13.453200 3.191290

O 10.755330 12.125360 4.002710

O 10.755330 14.781030 4.002710

O 13.128120 17.770750 5.692940

O 13.128120 15.114850 5.692940

O 10.774227 16.418063 6.535795

O 10.587944 13.420394 6.937403

O 13.095705 16.433832 7.930986

O 12.970248 13.426788 8.305552

O 10.585338 12.022706 9.075276

O 10.567496 14.757038 9.212423

Mg 16.804085 1.494237 0.409871
Mg 14.254469 5.934448 2.251782
Mg 14.255060 3.033660 2.261970
Mg 16.586740 4.484400 4.520030
Mg 18.960290 1.494800 5.174280
Mg 16.631791 0.049520 7.433432
Mg 16.625266 2.945861 7.432903
Mg 14.429152 4.476827 9.293904
Si 16.973535 4.484479 1.231951
Si 16.261340 1.492586 3.310057
Si 18.637210 4.484400 6.382630
Si 14.596270 1.494800 8.472360
O 17.673588 5.841297 0.559828
O 17.674523 3.128096 0.559685
O 15.349310 4.484400 1.386100
O 15.470890 1.494800 1.760420
O 17.713830 4.484400 2.799080
O 17.910830 1.494800 3.191290
O 15.504330 0.166960 4.002710
O 15.504330 2.822640 4.002710
O 17.877110 5.812340 5.692940
O 17.877110 3.156450 5.692940
O 15.503136 4.500450 6.525672
O 15.330549 1.498028 6.902727
O 17.838042 4.484354 7.926009
O 17.731295 1.498705 8.298979
O 15.301945 0.140702 9.140083
O 15.302673 2.848435 9.143158
Mg 16.803434 7.475430 0.408331
Mg 14.254873 11.911945 2.258169
Mg 14.255060 9.012860 2.261970
Mg 16.586740 10.463600 4.520030
Mg 18.960300 7.474000 5.174280
Mg 16.630261 6.029287 7.433564
Mg 16.619505 8.932956 7.443084
Mg 14.344280 10.462259 9.330184
Si 16.973748 10.463987 1.232364
Si 16.262901 7.476286 3.307682
Si 18.637210 10.463600 6.382630
Si 14.596270 7.474000 8.472360
O 17.674645 11.819401 0.558922
O 17.674495 9.108053 0.559137
O 15.349310 10.463600 1.386100
O 15.470890 7.474000 1.760420
O 17.713840 10.463600 2.799080
O 17.910830 7.474000 3.191290
O 15.504330 6.146160 4.002710
O 15.504330 8.801840 4.002710
O 17.877120 11.791540 5.692940
O 17.877110 9.135650 5.692940
O 15.529091 10.464105 6.490627
O 15.318202 7.472374 6.907184
O 17.840188 10.463261 7.929813
O 17.718099 7.476264 8.305231

O 15.283274 6.119610 9.149070

O 15.270630 8.844211 9.139198

Mg 16.804942 13.453287 0.411188

Mg 14.254065 17.891965 2.255669

Mg 14.255060 14.992060 2.261970

Mg 16.586740 16.442800 4.520030

Mg 18.960300 13.453200 5.174280

Mg 16.621275 11.996673 7.443826

Mg 16.636464 14.895338 7.436407

Mg 14.433211 16.444063 9.280203

Si 16.973626 16.442659 1.232074

Si 16.261465 13.452112 3.310528

Si 18.637220 16.442800 6.382630

Si 14.596270 13.453200 8.472360

O 17.675066 17.798545 0.559517

O 17.674840 15.087527 0.559136

O 15.349310 16.442800 1.386100

O 15.470890 13.453200 1.760420

O 17.713840 16.442800 2.799080

O 17.910830 13.453200 3.191290

O 15.504330 12.125360 4.002710

O 15.504330 14.781030 4.002710

O 17.877120 17.770750 5.692940

O 17.877120 15.114850 5.692940

O 15.527219 16.442091 6.511234

O 15.332321 13.457379 6.906026

O 17.841386 16.443325 7.928942

O 17.727155 13.447482 8.311569

O 15.270507 12.077673 9.133909

O 15.294860 14.805154 9.149710

C 11.454176 9.172320 10.486486

C 11.664000 10.513860 10.631167

C 10.562885 11.546955 10.462521

C 9.247701 10.911659 10.813772

C 9.031511 9.539373 10.722083

C 10.087725 8.572865 10.304436

C 7.669871 9.243818 11.218965

C 6.906155 8.091800 11.436688

C 5.572396 8.237729 11.940632

C 4.982874 9.486969 12.198669

C 5.755345 10.692278 12.006247

C 7.079533 10.507759 11.534810

C 8.020338 11.563303 11.305392

C 7.606288 12.870784 11.571983

C 6.271741 13.092045 12.052644

C 5.358212 12.055675 12.269880

H 12.288886 8.465158 10.570367

H 12.651023 10.894806 10.957069

H 10.761428 12.427834 11.106679

H 10.054779 7.686908 10.976603

H 7.263320 7.090303 11.173126

H 4.977149 7.332286 12.103963

H 3.961702 9.540439 12.595462

H 8.268316 13.730152 11.415649

H 5.958137 14.122361 12.253687

H 4.352268 12.274252 12.646236

**Fluoranthene V$_{MgO}$-[010]-fo (⊥)**

Number of atoms: 360

Mg 2.543480 1.494800 0.388370

Mg 0.008060 5.935140 2.261970

Mg 0.008060 3.033660 2.261970

Mg 2.344315 4.486556 4.519786

Mg 4.714293 1.489945 5.177242

Mg 2.376436 0.043734 7.436312

Mg 2.377149 2.944615 7.434383

Mg 0.178939 4.484137 9.282955

Si 2.727970 4.484400 1.221250

Si 2.018610 1.494800 3.313620

Si 4.380474 4.483469 6.391477

Si 0.353205 1.495792 8.463869

O 3.434710 5.847120 0.556660

O 3.434700 3.121670 0.556660

O 1.102310 4.484400 1.386100

O 1.223890 1.494800 1.760420

O 3.466830 4.484400 2.799080

O 3.663830 1.494800 3.191290

O 1.257330 0.166960 4.002710

O 1.257330 2.822640 4.002710

O 3.635482 5.809897 5.701633

O 3.628284 3.163468 5.694957

O 1.269958 4.489633 6.518111

O 1.088852 1.495266 6.896897

O 3.589571 4.480499 7.935499

O 3.477787 1.494641 8.301818

O 1.052125 0.140360 9.136135

O 1.052980 2.852315 9.133697

Mg 2.543480 7.474000 0.388370

Mg 0.008060 11.914340 2.261970

Mg 0.008060 9.012860 2.261970

Mg 2.356144 10.463211 4.532551

Mg 4.728567 7.485581 5.193565

Mg 2.385981 6.030244 7.445946

Mg 2.389003 8.924789 7.454917

Mg 0.183252 10.462318 9.296159

Si 2.727970 10.463600 1.221250

Si 2.018610 7.474000 3.313620

Si 4.399377 10.460905 6.417951

Si 0.352032 7.476394 8.471474

O 3.434710 11.826320 0.556660

O 3.434710 9.100870 0.556660

O 1.102310 10.463600 1.386100

O 1.223890 7.474000 1.760420

O 3.466830 10.463600 2.799080

O 3.663830 7.474000 3.191290

O 1.257330 6.146160 4.002710

O 1.257330 8.801840 4.002710

O 3.640016 11.776141 5.713010

O 3.639328 9.144140 5.716907

O 1.279373 10.463399 6.529295

O 1.093182 7.477831 6.907298

O 3.605379 10.460435 7.962666

O 3.490332 7.476549 8.316829

O 1.050019 6.117559 9.139970

O 1.052783 8.827169 9.150411

Mg 2.543480 13.453200 0.388370

Mg 0.008060 17.893540 2.261970

Mg 0.008060 14.992060 2.261970

Mg 2.343797 16.440085 4.521164

Mg 4.722906 13.440579 5.184831

Mg 2.389223 12.000478 7.454936

Mg 2.381771 14.896011 7.446100

Mg 0.182424 16.442967 9.285022

Si 2.727970 16.442800 1.221250

Si 2.018610 13.453200 3.313620

Si 4.378255 16.437749 6.393506

Si 0.354341 13.448773 8.479340

O 3.434710 17.805520 0.556660

O 3.434710 15.080070 0.556660

O 1.102310 16.442800 1.386100

O 1.223890 13.453200 1.760420

O 3.466830 16.442800 2.799080

O 3.663830 13.453200 3.191290

O 1.257330 12.125360 4.002710

O 1.257330 14.781030 4.002710

O 3.626065 17.758806 5.696659

O 3.630186 15.113676 5.701848

O 1.268368 16.436071 6.521923

O 1.089417 13.446919 6.912198

O 3.587571 16.443333 7.938385

O 3.487045 13.453382 8.308738

O 1.056707 12.095395 9.153882

O 1.052659 14.808107 9.147226

Mg 7.292480 1.494800 0.388370

Mg 4.757060 5.935140 2.261970

Mg 4.757060 3.033660 2.261970

Mg 7.091728 4.483961 4.517340

Mg 9.465719 1.487585 5.177031

Mg 7.127973 0.033390 7.445617

Mg 7.125847 2.929264 7.433000

Mg 4.919525 4.475184 9.291689

Si 7.476970 4.484400 1.221250

Si 6.767610 1.494800 3.313620

Si 9.126738 4.476093 6.397683

Si 5.098684 1.489830 8.464482

O 8.183700 5.847120 0.556660

O 8.183700 3.121670 0.556660

O 5.851310 4.484400 1.386100

O 5.972890 1.494800 1.760420

O 8.215830 4.484400 2.799080

O 8.412830 1.494800 3.191290

O 6.006330 0.166960 4.002710

O 6.006330 2.822640 4.002710

O 8.387313 5.803911 5.705379

O 8.381215 3.157974 5.694113

O 6.023899 4.476552 6.506117

O 5.837368 1.484951 6.898068

O 8.333912 4.463240 7.938611

O 8.234611 1.481017 8.305560

O 5.798716 0.134975 9.137480

O 5.802740 2.846663 9.132114

Mg 7.292480 7.474000 0.388370

Mg 4.757060 11.914340 2.261970

Mg 4.757060 9.012860 2.261970

Mg 7.115727 10.462531 4.543581

Mg 9.447134 7.486293 5.197919

Mg 7.106186 6.004164 7.434459

Mg 7.188468 8.931955 7.482438

Mg 4.948807 10.470624 9.318441

Si 7.476970 10.463600 1.221250

Si 6.767610 7.474000 3.313620

Si 9.159043 10.452712 6.428057

Si 5.113144 7.477521 8.483690

O 8.183700 11.826320 0.556660

O 8.183700 9.100870 0.556660

O 5.851310 10.463600 1.386100

O 5.972890 7.474000 1.760420

O 8.215830 10.463600 2.799080

O 8.412830 7.474000 3.191290

O 6.006330 6.146160 4.002710

O 6.006330 8.801840 4.002710

O 8.401461 11.767788 5.694001

O 8.390365 9.135190 5.710966

O 6.040770 10.452973 6.549415

O 5.851500 7.495053 6.920524

O 8.467360 10.467071 7.961527

O 8.201172 7.397879 8.326586

O 5.795322 6.103894 9.139521

O 5.816756 8.819889 9.193756

Mg 7.292480 13.453200 0.388370

Mg 4.757060 17.893540 2.261970

Mg 4.757060 14.992060 2.261970

Mg 7.092168 16.441962 4.518608

Mg 9.446667 13.437113 5.161190

Mg 7.163438 11.969757 7.481658

Mg 7.108454 14.900794 7.413024

Mg 4.924180 16.438656 9.288934

Si 7.476970 16.442800 1.221250

Si 6.767610 13.453200 3.313620

Si 9.130660 16.432522 6.389727

Si 5.111417 13.446421 8.461999

O 8.183700 17.805520 0.556660

O 8.183700 15.080070 0.556660

O 5.851310 16.442800 1.386100

O 5.972890 13.453200 1.760420

O 8.215830 16.442800 2.799080

O 8.412830 13.453200 3.191290

O 6.006330 12.125360 4.002710

O 6.006330 14.781030 4.002710

O 8.382976 17.759136 5.701957

O 8.377262 15.117213 5.687340

O 6.021820 16.442656 6.506856

O 5.846126 13.425369 6.895953

O 8.345198 16.424698 7.936055

O 8.207769 13.480010 8.284484

O 5.812791 12.104283 9.148101

O 5.805100 14.816326 9.114924

Mg 12.041480 1.494800 0.388370

Mg 9.506060 5.935140 2.261970

Mg 9.506060 3.033660 2.261970

Mg 11.839129 4.484065 4.521073

Mg 14.213421 1.499794 5.180188

Mg 11.879061 0.031080 7.436600

Mg 11.873144 2.938809 7.444834

Mg 9.651109 4.417390 9.304771

Si 12.225970 4.484400 1.221250

Si 11.516610 1.494800 3.313620

Si 13.867353 4.502248 6.403248

Si 9.854799 1.459786 8.467717

O 12.932710 5.847120 0.556660

O 12.932710 3.121670 0.556660

O 10.600310 4.484400 1.386100

O 10.721890 1.494800 1.760420

O 12.964830 4.484400 2.799080

O 13.161830 1.494800 3.191290

O 10.755330 0.166960 4.002710

O 10.755330 2.822640 4.002710

O 13.111537 5.831570 5.721542

O 13.125999 3.179947 5.699328

O 10.768689 4.494295 6.518915

O 10.591735 1.483268 6.900059

O 13.077991 4.489661 7.948265

O 12.978499 1.487375 8.303533

O 10.550975 0.092847 9.124640

O 10.558672 2.807502 9.151759

Mg 12.041480 7.474000 0.388370

Mg 9.506060 11.914340 2.261970

Mg 9.506060 9.012860 2.261970

Mg 11.836596 10.475516 4.500507

Mg 14.232687 7.465918 5.159195

Mg 11.845854 6.024133 7.478747

Mg 11.941781 8.925582 7.242719

Si 12.225507 10.459193 1.228864

Si 11.516610 7.474000 3.313620

Si 13.913264 10.485022 6.357457

Si 9.801873 7.417938 8.615984

O 12.927795 11.816458 0.557652

O 12.932710 9.100870 0.556660

O 10.600310 10.463600 1.386100

O 10.721890 7.474000 1.760420

O 12.964840 10.463600 2.799080
O 13.161830 7.474000 3.191290
O 10.755330 6.146160 4.002710
O 10.755330 8.801840 4.002710
O 13.145242 11.796874 5.672845
O 13.178651 9.156794 5.633670
O 10.817828 10.440415 6.423223
O 10.556017 7.563689 7.058927
O 13.088884 10.443233 7.881553
O 12.947498 7.514990 8.301750
O 10.569389 6.030361 9.154729
Mg 12.055965 13.451508 0.411601
Mg 9.505263 17.892942 2.257441
Mg 9.506060 14.992060 2.261970
Mg 11.840643 16.439571 4.520728
Mg 14.222174 13.459453 5.172912
Mg 11.851388 11.959602 7.385547
Mg 11.887288 14.876300 7.424635
Mg 9.690660 16.385467 9.279468
Si 12.223790 16.442409 1.231024
Si 11.511005 13.453858 3.310909
Si 13.881835 16.437327 6.395371
Si 9.815266 13.450707 8.416628
O 12.921640 17.800962 0.557744
O 12.927869 15.086268 0.560258
O 10.600310 16.442800 1.386100
O 10.721890 13.453200 1.760420
O 12.964840 16.442800 2.799080
O 13.161830 13.453200 3.191290
O 10.755330 12.125360 4.002710
O 10.755330 14.781030 4.002710
O 13.131523 17.760460 5.703090
O 13.132553 15.116244 5.698021
O 10.772705 16.425073 6.516846
O 10.584333 13.446817 6.876507
O 13.085760 16.424972 7.938262
O 12.943913 13.400206 8.303277
O 10.410351 11.970842 9.025668
O 10.566879 14.736747 9.147847
Mg 16.804110 1.494072 0.410611
Mg 14.253552 5.934082 2.253318
Mg 14.255060 3.033660 2.261970
Mg 16.591442 4.484878 4.521796
Mg 18.961926 1.493688 5.178429
Mg 16.626798 0.044621 7.440853
Mg 16.623163 2.947337 7.438445
Mg 14.422289 4.482147 9.289768
Si 16.973419 4.484437 1.232115
Si 16.261036 1.493212 3.310338
Si 18.625141 4.491548 6.395511
Si 14.599126 1.492519 8.467906
O 17.673942 5.841378 0.559934
O 17.674800 3.128144 0.559678
O 15.349310 4.484400 1.386100

O 15.470890 1.494800 1.760420

O 17.713830 4.484400 2.799080

O 17.910830 1.494800 3.191290

O 15.504330 0.166960 4.002710

O 15.504330 2.822640 4.002710

O 17.872866 5.813108 5.701680

O 17.873875 3.170348 5.698465

O 15.510568 4.498077 6.522281

O 15.335432 1.496696 6.901014

O 17.829497 4.490283 7.940339

O 17.728567 1.497550 8.300678

O 15.299229 0.134418 9.137000

O 15.298217 2.850548 9.139512

Mg 16.803354 7.475185 0.408600

Mg 14.254730 11.912552 2.258083

Mg 14.255060 9.012860 2.261970

Mg 16.600621 10.465958 4.526883

Mg 18.969132 7.479631 5.182936

Mg 16.620215 6.034534 7.442368

Mg 16.621224 8.936991 7.452668

Mg 14.314758 10.461694 9.334048

Si 16.973604 10.463915 1.232138

Si 16.262528 7.475788 3.307732

Si 18.634243 10.466789 6.402178

Si 14.584532 7.493791 8.464697

O 17.675017 11.819395 0.559111

O 17.674822 9.108097 0.559283

O 15.349310 10.463600 1.386100

O 15.470890 7.474000 1.760420

O 17.713840 10.463600 2.799080

O 17.910830 7.474000 3.191290

O 15.504330 6.146160 4.002710

O 15.504330 8.801840 4.002710

O 17.884161 11.788687 5.707951

O 17.883169 9.144769 5.706899

O 15.548235 10.471366 6.489404

O 15.323686 7.486640 6.906139

O 17.841190 10.464818 7.947577

O 17.724290 7.479460 8.310859

O 15.266210 6.132423 9.139004

O 15.257914 8.855674 9.142820

Mg 16.804823 13.453409 0.411248

Mg 14.253328 17.892647 2.256105

Mg 14.255060 14.992060 2.261970

Mg 16.593050 16.440766 4.524581

Mg 18.968921 13.449662 5.184532

Mg 16.620651 11.996510 7.461688

Mg 16.616530 14.888843 7.456175

Mg 14.424059 16.437661 9.287899

Si 16.973581 16.442630 1.232034

Si 16.261214 13.452593 3.310666

Si 18.623403 16.432565 6.399935

Si 14.569086 13.426185 8.473852

O 17.675469 17.798197 0.559202

O 17.675376 15.087243 0.559380

O 15.349310 16.442800 1.386100

O 15.470890 13.453200 1.760420

O 17.713840 16.442800 2.799080

O 17.910830 13.453200 3.191290

O 15.504330 12.125360 4.002710

O 15.504330 14.781030 4.002710

O 17.876951 17.757191 5.703084

O 17.872510 15.110757 5.707030

O 15.522761 16.435497 6.523248

O 15.323201 13.441876 6.914930

O 17.830131 16.435558 7.945351

O 17.726392 13.446844 8.324321

O 15.258541 12.063349 9.141455

O 15.262040 14.784678 9.151261

C 11.416834 9.092751 10.482712

C 10.154857 8.738080 9.923654

C 8.980944 9.308659 10.492010

C 9.093834 10.274641 11.499127

C 10.378513 10.719736 11.969910

C 11.541104 10.087843 11.485634

C 10.146600 11.772685 12.983508

C 10.951697 12.565162 13.806790

C 10.320530 13.490709 14.700355

C 8.928965 13.630569 14.790580

C 8.073104 12.824699 13.968024

C 8.725324 11.927674 13.086226

C 8.044991 11.020118 12.218616

C 6.648239 10.985323 12.250147

C 5.960703 11.896339 13.115653

C 6.637869 12.799124 13.946915

H 12.300358 8.534486 10.143992

H 7.992067 8.977667 10.150191

H 12.523001 10.320834 11.925038

H 12.045224 12.492966 13.787449

H 10.955690 14.109412 15.344549

H 8.496573 14.348790 15.496933

H 6.096875 10.217324 11.685926

H 4.865087 11.878711 13.139160

H 6.069027 13.473278 14.597433

H 9.695679 11.279607 8.871129

**Pyrene [010]-fo**

Number of atoms: 362

Mg 2.543480 1.494800 0.388370

Mg 0.008060 5.935140 2.261970

Mg 0.008060 3.033660 2.261970

Mg 2.345122 4.484885 4.518732

Mg 4.720340 1.492333 5.179449

Mg 2.388802 0.044265 7.442628

Mg 2.388554 2.943691 7.438797

Mg 0.189290 4.482514 9.280469

Si 2.727970 4.484400 1.221250

Si 2.018610 1.494800 3.313620

Si 4.386991 4.484451 6.386459

Si 0.363146 1.495121 8.467587

O 3.434710 5.847120 0.556660

O 3.434700 3.121670 0.556660

O 1.102310 4.484400 1.386100

O 1.223890 1.494800 1.760420

O 3.466830 4.484400 2.799080

O 3.663830 1.494800 3.191290

O 1.257330 0.166960 4.002710

O 1.257330 2.822640 4.002710

O 3.633473 5.803229 5.691946

O 3.635195 3.161304 5.696632

O 1.277242 4.484893 6.517823

O 1.099538 1.494532 6.901258

O 3.598936 4.486121 7.932746

O 3.489424 1.495007 8.308010

O 1.063139 0.139769 9.140905

O 1.064516 2.850485 9.139496

Mg 2.543480 7.474000 0.388370

Mg 0.008060 11.914340 2.261970

Mg 0.008060 9.012860 2.261970

Mg 2.358243 10.463124 4.538719

Mg 4.717670 7.479516 5.177714

Mg 2.384972 6.024309 7.437095

Mg 2.397955 8.923628 7.463180

Mg 0.192358 10.459415 9.284269

Si 2.727970 10.463600 1.221250

Si 2.018610 7.474000 3.313620

Si 4.397837 10.462778 6.437311

Si 0.362217 7.473826 8.475883

O 3.434710 11.826320 0.556660

O 3.434710 9.100870 0.556660

O 1.102310 10.463600 1.386100

O 1.223890 7.474000 1.760420

O 3.466830 10.463600 2.799080

O 3.663830 7.474000 3.191290

O 1.257330 6.146160 4.002710

O 1.257330 8.801840 4.002710

O 3.637564 11.780945 5.732781

O 3.638751 9.144973 5.730919

O 1.281755 10.462033 6.538634

O 1.098742 7.478697 6.909844

O 3.619310 10.461356 7.989926

O 3.491610 7.457830 8.314193

O 1.062109 6.115591 9.144914

O 1.066074 8.823655 9.158950

Mg 2.543480 13.453200 0.388370

Mg 0.008060 17.893540 2.261970

Mg 0.008060 14.992060 2.261970

Mg 2.345542 16.441753 4.520758

Mg 4.716549 13.445014 5.177312

Mg 2.396337 11.998706 7.465904

Mg 2.381183 14.899439 7.441052

Mg 0.190678 16.443386 9.283067

| | | | |
|---|---|---|---|
| Si | 2.727970 | 16.442800 | 1.221250 |
| Si | 2.018610 | 13.453200 | 3.313620 |
| Si | 4.385575 | 16.437835 | 6.391899 |
| Si | 0.361110 | 13.447180 | 8.482230 |
| O | 3.434710 | 17.805520 | 0.556660 |
| O | 3.434710 | 15.080070 | 0.556660 |
| O | 1.102310 | 16.442800 | 1.386100 |
| O | 1.223890 | 13.453200 | 1.760420 |
| O | 3.466830 | 16.442800 | 2.799080 |
| O | 3.663830 | 13.453200 | 3.191290 |
| O | 1.257330 | 12.125360 | 4.002710 |
| O | 1.257330 | 14.781030 | 4.002710 |
| O | 3.634759 | 17.761435 | 5.700614 |
| O | 3.631456 | 15.118835 | 5.697665 |
| O | 1.276211 | 16.440293 | 6.521794 |
| O | 1.096191 | 13.442672 | 6.915387 |
| O | 3.598092 | 16.437213 | 7.939355 |
| O | 3.487708 | 13.466159 | 8.315596 |
| O | 1.064901 | 12.095775 | 9.163390 |
| O | 1.059305 | 14.807805 | 9.148564 |
| Mg | 7.292480 | 1.494800 | 0.388370 |
| Mg | 4.757060 | 5.935140 | 2.261970 |
| Mg | 4.757060 | 3.033660 | 2.261970 |
| Mg | 7.094854 | 4.484976 | 4.517274 |
| Mg | 9.471374 | 1.491369 | 5.177824 |
| Mg | 7.137861 | 0.045173 | 7.440514 |
| Mg | 7.137891 | 2.939030 | 7.437529 |
| Mg | 4.949840 | 4.490549 | 9.263892 |
| Si | 7.476970 | 4.484400 | 1.221250 |
| Si | 6.767610 | 1.494800 | 3.313620 |
| Si | 9.136778 | 4.478662 | 6.382850 |
| Si | 5.110370 | 1.493868 | 8.470112 |
| O | 8.183700 | 5.847120 | 0.556660 |
| O | 8.183700 | 3.121670 | 0.556660 |
| O | 5.851310 | 4.484400 | 1.386100 |
| O | 5.972890 | 1.494800 | 1.760420 |
| O | 8.215830 | 4.484400 | 2.799080 |
| O | 8.412830 | 1.494800 | 3.191290 |
| O | 6.006330 | 0.166960 | 4.002710 |
| O | 6.006330 | 2.822640 | 4.002710 |
| O | 8.384585 | 5.795944 | 5.686344 |
| O | 8.383884 | 3.155919 | 5.694785 |
| O | 6.027979 | 4.481073 | 6.523414 |
| O | 5.846017 | 1.493217 | 6.902684 |
| O | 8.347138 | 4.480737 | 7.929668 |
| O | 8.244602 | 1.493011 | 8.306747 |
| O | 5.811776 | 0.137241 | 9.141110 |
| O | 5.813911 | 2.849781 | 9.141084 |
| Mg | 7.292480 | 7.474000 | 0.388370 |
| Mg | 4.757060 | 11.914340 | 2.261970 |
| Mg | 4.757060 | 9.012860 | 2.261970 |
| Mg | 7.098224 | 10.463733 | 4.532700 |
| Mg | 9.465665 | 7.477254 | 5.173572 |
| Mg | 7.145176 | 6.022809 | 7.431188 |

Mg 7.133440 8.927296 7.434101

Mg 4.925292 10.457735 9.423387

Si 7.476970 10.463600 1.221250

Si 6.767610 7.474000 3.313620

Si 9.144657 10.462136 6.412273

Si 5.114354 7.471738 8.453888

O 8.183700 11.826320 0.556660

O 8.183700 9.100870 0.556660

O 5.851310 10.463600 1.386100

O 5.972890 7.474000 1.760420

O 8.215830 10.463600 2.799080

O 8.412830 7.474000 3.191290

O 6.006330 6.146160 4.002710

O 6.006330 8.801840 4.002710

O 8.388371 11.783759 5.709830

O 8.387208 9.141880 5.709255

O 6.041295 10.462771 6.493324

O 5.845668 7.447103 6.889885

O 8.358957 10.462323 7.953171

O 8.237898 7.465708 8.297661

O 5.821830 6.133583 9.178610

O 5.817555 8.839493 9.082243

Mg 7.292480 13.453200 0.388370

Mg 4.757060 17.893540 2.261970

Mg 4.757060 14.992060 2.261970

Mg 7.094810 16.440744 4.519556

Mg 9.465428 13.446672 5.171800

Mg 7.131538 11.993935 7.434880

Mg 7.143628 14.897454 7.434758

Mg 4.951906 16.431629 9.269854

Si 7.476970 16.442800 1.221250

Si 6.767610 13.453200 3.313620

Si 9.136592 16.440243 6.387733

Si 5.109974 13.451824 8.452062

O 8.183700 17.805520 0.556660

O 8.183700 15.080070 0.556660

O 5.851310 16.442800 1.386100

O 5.972890 13.453200 1.760420

O 8.215830 16.442800 2.799080

O 8.412830 13.453200 3.191290

O 6.006330 12.125360 4.002710

O 6.006330 14.781030 4.002710

O 8.384773 17.763498 5.697449

O 8.383570 15.121581 5.693393

O 6.027421 16.440644 6.524479

O 5.844581 13.471818 6.889323

O 8.347554 16.439835 7.935150

O 8.238206 13.451909 8.299949

O 5.808522 12.085530 9.090991

O 5.822568 14.788918 9.171321

Mg 12.041480 1.494800 0.388370

Mg 9.506060 5.935140 2.261970

Mg 9.506060 3.033660 2.261970

Mg 11.845515 4.486287 4.518748

Mg 14.219817 1.492335 5.178625

Mg 11.889536 0.046067 7.439137

Mg 11.889147 2.940368 7.436693

Mg 9.703139 4.488041 9.251962

Si 12.225970 4.484400 1.221250

Si 11.516610 1.494800 3.313620

Si 13.887357 4.482757 6.390260

Si 9.865243 1.492606 8.469446

O 12.932710 5.847120 0.556660

O 12.932710 3.121670 0.556660

O 10.600310 4.484400 1.386100

O 10.721890 1.494800 1.760420

O 12.964830 4.484400 2.799080

O 13.161830 1.494800 3.191290

O 10.755330 0.166960 4.002710

O 10.755330 2.822640 4.002710

O 13.136391 5.802501 5.696137

O 13.135577 3.160787 5.698003

O 10.776758 4.478746 6.524216

O 10.599036 1.493489 6.901460

O 13.096156 4.482754 7.936343

O 12.988855 1.494231 8.309066

O 10.567421 0.136419 9.140995

O 10.568354 2.848516 9.141723

Mg 12.041480 7.474000 0.388370

Mg 9.506060 11.914340 2.261970

Mg 9.506060 9.012860 2.261970

Mg 11.838420 10.462842 4.523130

Mg 14.219777 7.476977 5.180227

Mg 11.891461 6.026572 7.438719

Mg 11.869446 8.930982 7.419954

Mg 9.629942 10.465161 9.397390

Si 12.225970 10.463600 1.221250

Si 11.516610 7.474000 3.313620

Si 13.889531 10.461516 6.391807

Si 9.859674 7.482804 8.447455

O 12.932710 11.826320 0.556660

O 12.932710 9.100870 0.556660

O 10.600310 10.463600 1.386100

O 10.721890 7.474000 1.760420

O 12.964840 10.463600 2.799080

O 13.161830 7.474000 3.191290

O 10.755330 6.146160 4.002710

O 10.755330 8.801840 4.002710

O 13.140544 11.784054 5.693757

O 13.140240 9.140204 5.692763

O 10.789115 10.462028 6.463693

O 10.596680 7.446985 6.883153

O 13.091669 10.461453 7.925544

O 12.978669 7.477040 8.311092

O 10.559820 6.137303 9.165267

O 10.558066 8.859479 9.061973

Mg 12.041480 13.453200 0.388370

Mg 9.506060 17.893540 2.261970

Mg 9.506060 14.992060 2.261970

Mg 11.845351 16.439150 4.520195

Mg 14.221720 13.447243 5.182727

Mg 11.871988 11.993301 7.415958

Mg 11.892093 14.896517 7.440551

Mg 9.705521 16.432221 9.258142

Si 12.225970 16.442800 1.221250

Si 11.516610 13.453200 3.313620

Si 13.887135 16.439028 6.394243

Si 9.860868 13.437244 8.439033

O 12.932710 17.805520 0.556660

O 12.932710 15.080070 0.556660

O 10.600310 16.442800 1.386100

O 10.721890 13.453200 1.760420

O 12.964840 16.442800 2.799080

O 13.161830 13.453200 3.191290

O 10.755330 12.125360 4.002710

O 10.755330 14.781030 4.002710

O 13.135669 17.761459 5.701535

O 13.136686 15.118568 5.700319

O 10.776918 16.443407 6.526717

O 10.597948 13.478864 6.877061

O 13.095455 16.439649 7.940947

O 12.978040 13.446244 8.311354

O 10.566121 12.064108 9.051513

O 10.553906 14.779065 9.166685

Mg 16.790480 1.494800 0.388370

Mg 14.255060 5.935140 2.261970

Mg 14.255060 3.033660 2.261970

Mg 16.593854 4.485819 4.520706

Mg 18.968090 1.492389 5.180051

Mg 16.634217 0.043802 7.441601

Mg 16.635192 2.942734 7.438616

Mg 14.441710 4.485121 9.275076

Si 16.974970 4.484400 1.221250

Si 16.265610 1.494800 3.313620

Si 18.632131 4.486620 6.392992

Si 14.609599 1.494466 8.470329

O 17.681710 5.847120 0.556660

O 17.681710 3.121670 0.556660

O 15.349310 4.484400 1.386100

O 15.470890 1.494800 1.760420

O 17.713830 4.484400 2.799080

O 17.910830 1.494800 3.191290

O 15.504330 0.166960 4.002710

O 15.504330 2.822640 4.002710

O 17.881387 5.808465 5.700289

O 17.882048 3.164507 5.697927

O 15.528379 4.485013 6.521450

O 15.344127 1.494083 6.902624

O 17.842164 4.485859 7.939181

O 17.738559 1.494453 8.305607

O 15.310717 0.138639 9.142269

O 15.311912 2.849356 9.142495

Mg 16.790480 7.474000 0.388370

Mg 14.255060 11.914340 2.261970

Mg 14.255060 9.012860 2.261970

Mg 16.593478 10.464035 4.526903

Mg 18.970525 7.478472 5.183236

Mg 16.632353 6.031173 7.445233

Mg 16.626290 8.929518 7.450750

Mg 14.374356 10.458324 9.319813

Si 16.974970 10.463600 1.221250

Si 16.265610 7.474000 3.313620

Si 18.636586 10.463677 6.400348

Si 14.599053 7.480733 8.477039

O 17.681710 11.826320 0.556660

O 17.681710 9.100870 0.556660

O 15.349310 10.463600 1.386100

O 15.470890 7.474000 1.760420

O 17.713840 10.463600 2.799080

O 17.910830 7.474000 3.191290

O 15.504330 6.146160 4.002710

O 15.504330 8.801840 4.002710

O 17.886070 11.785546 5.706763

O 17.885257 9.143145 5.704776

O 15.533391 10.463221 6.508117

O 15.340009 7.477587 6.911449

O 17.844299 10.462145 7.946237

O 17.736877 7.477956 8.314589

O 15.304256 6.125061 9.150710

O 15.289485 8.840234 9.153027

Mg 16.790480 13.453200 0.388370

Mg 14.255060 17.893540 2.261970

Mg 14.255060 14.992060 2.261970

Mg 16.593844 16.439749 4.521972

Mg 18.972040 13.446969 5.185543

Mg 16.625794 11.993776 7.455613

Mg 16.633145 14.890689 7.450657

Mg 14.443113 16.438618 9.278336

Si 16.974970 16.442800 1.221250

Si 16.265610 13.453200 3.313620

Si 18.630867 16.435892 6.397334

Si 14.597862 13.441345 8.478869

O 17.681710 17.805520 0.556660

O 17.681710 15.080070 0.556660

O 15.349310 16.442800 1.386100

O 15.470890 13.453200 1.760420

O 17.713840 16.442800 2.799080

O 17.910830 13.453200 3.191290

O 15.504330 12.125360 4.002710

O 15.504330 14.781030 4.002710

O 17.881812 17.758792 5.702016

O 17.881522 15.112119 5.706521

O 15.528526 16.437003 6.523501

O 15.341081 13.445059 6.914721

O 17.840990 16.437522 7.943874

O 17.735587 13.442924 8.323143

O 15.284571 12.079438 9.154587

O 15.302678 14.796815 9.153456

C 12.094516 10.200836 11.993029

C 11.403482 11.427358 11.920834

C 9.980273 11.450260 11.889057

C 9.261677 10.195529 11.911472

C 9.985286 8.943103 11.948938

C 11.403160 8.970702 11.986501

C 9.236766 7.708707 11.865369

C 7.855907 7.708174 11.816749

C 7.104413 8.941112 11.893435

C 5.688571 8.959195 11.972396

C 4.985348 10.188239 12.016395

C 5.679843 11.418661 11.920143

C 7.101119 11.441867 11.838771

C 7.817526 10.193850 11.884352

C 7.845332 12.675423 11.704782

C 9.227149 12.680790 11.747708

H 13.191102 10.199738 12.076519

H 11.950801 12.374480 11.862903

H 11.956530 8.025589 11.977516

H 9.804609 6.773487 11.798555

H 7.294801 6.774787 11.685316

H 5.141889 8.010930 12.004441

H 3.898128 10.185731 12.169904

H 5.133031 12.367179 11.935626

H 7.285854 13.602139 11.523799

H 9.788983 13.614286 11.621483

**Pyrene Fe-[010]-fo**

Number of atoms: 362

Mg 2.543480 1.494800 0.388370

Mg 0.008060 5.935140 2.261970

Mg 0.008060 3.033660 2.261970

Mg 2.344663 4.484485 4.517172

Mg 4.719022 1.494335 5.177971

Mg 2.385181 0.048073 7.435519

Mg 2.384943 2.945296 7.433432

Mg 0.189260 4.483516 9.281788

Si 2.727970 4.484400 1.221250

Si 2.018610 1.494800 3.313620

Si 4.383752 4.485348 6.381814

Si 0.360930 1.496889 8.462677

O 3.434710 5.847120 0.556660

O 3.434700 3.121670 0.556660

O 1.102310 4.484400 1.386100

O 1.223890 1.494800 1.760420

O 3.466830 4.484400 2.799080

O 3.663830 1.494800 3.191290

O 1.257330 0.166960 4.002710

O 1.257330 2.822640 4.002710

O 3.629168 5.803936 5.687413

O 3.631216 3.162272 5.691465

O 1.274873 4.490532 6.516514

O 1.096484 1.497831 6.895675

O 3.592124 4.485144 7.926351

O 3.485688 1.497798 8.305243

O 1.060780 0.142043 9.135769

O 1.062410 2.851519 9.134921

Mg 2.543480 7.474000 0.388370

Mg 0.008060 11.914340 2.261970

Mg 0.008060 9.012860 2.261970

Mg 2.359724 10.464611 4.532452

Mg 4.714020 7.480490 5.169367

Mg 2.382315 6.027414 7.434273

Mg 2.397590 8.926719 7.448970

Mg 0.194932 10.465345 9.280239

Si 2.727970 10.463600 1.221250

Si 2.018610 7.474000 3.313620

Si 4.399126 10.466162 6.421845

Si 0.367622 7.476644 8.500568

O 3.434710 11.826320 0.556660

O 3.434710 9.100870 0.556660

O 1.102310 10.463600 1.386100

O 1.223890 7.474000 1.760420

O 3.466830 10.463600 2.799080

O 3.663830 7.474000 3.191290

O 1.257330 6.146160 4.002710

O 1.257330 8.801840 4.002710

O 3.637893 11.782815 5.713124

O 3.638987 9.147633 5.714542

O 1.275059 10.462264 6.533784

O 1.090174 7.487805 6.927154

O 3.603203 10.466026 7.957773

O 3.486429 7.464366 8.295126

O 1.068879 6.115150 9.161333

O 1.073259 8.822616 9.202754

Mg 2.543480 13.453200 0.388370

Mg 0.008060 17.893540 2.261970

Mg 0.008060 14.992060 2.261970

Mg 2.345486 16.442513 4.519194

Mg 4.719019 13.446392 5.179384

Mg 2.392209 12.005059 7.446443

Mg 2.377262 14.905535 7.431323

Mg 0.186307 16.446796 9.279579

Si 2.727970 16.442800 1.221250

Si 2.018610 13.453200 3.313620

Si 4.383852 16.441975 6.389405

Si 0.360944 13.453790 8.473223

O 3.434710 17.805520 0.556660

O 3.434710 15.080070 0.556660

O 1.102310 16.442800 1.386100

O 1.223890 13.453200 1.760420

O 3.466830 16.442800 2.799080

O 3.663830 13.453200 3.191290

O 1.257330 12.125360 4.002710

O 1.257330 14.781030 4.002710

O 3.633267 17.764974 5.695813

O 3.631803 15.122831 5.693159

O 1.273122 16.443863 6.513847

O 1.090317 13.449412 6.903882

O 3.591420 16.444431 7.933522

O 3.473677 13.470395 8.297882

O 1.063558 12.101747 9.151652

O 1.058069 14.813819 9.140274

Mg 7.292480 1.494800 0.388370

Mg 4.757060 5.935140 2.261970

Mg 4.757060 3.033660 2.261970

Mg 7.094276 4.485071 4.518541

Mg 9.468042 1.492243 5.178002

Mg 7.132114 0.044522 7.441308

Mg 7.135467 2.940785 7.436968

Mg 4.943536 4.486074 9.254911

Si 7.476970 4.484400 1.221250

Si 6.767610 1.494800 3.313620

Si 9.136929 4.481531 6.386817

Si 5.106463 1.493547 8.469455

O 8.183700 5.847120 0.556660

O 8.183700 3.121670 0.556660

O 5.851310 4.484400 1.386100

O 5.972890 1.494800 1.760420

O 8.215830 4.484400 2.799080

O 8.412830 1.494800 3.191290

O 6.006330 0.166960 4.002710

O 6.006330 2.822640 4.002710

O 8.385423 5.799045 5.687893

O 8.384269 3.159008 5.696835

O 6.025285 4.475271 6.513507

O 5.842473 1.493918 6.902447

O 8.344959 4.485728 7.931296

O 8.237863 1.493930 8.306687

O 5.805170 0.135903 9.140774

O 5.811321 2.848059 9.142611

Mg 7.292480 7.474000 0.388370

Mg 4.757060 11.914340 2.261970

Mg 4.757060 9.012860 2.261970

Mg 7.100144 10.466358 4.543687

Mg 9.463027 7.479568 5.173772

Mg 7.132344 6.020856 7.421537

Mg 7.129118 8.931386 7.436685

Mg 4.870716 10.439614 9.413453

Si 7.476970 10.463600 1.221250

Si 6.767610 7.474000 3.313620

Si 9.156731 10.467678 6.440220

Si 5.111063 7.479626 8.425039

O 8.183700 11.826320 0.556660

O 8.183700 9.100870 0.556660

O 5.851310 10.463600 1.386100

O 5.972890 7.474000 1.760420

O 8.215830 10.463600 2.799080

O 8.412830 7.474000 3.191290

O 6.006330 6.146160 4.002710

O 6.006330 8.801840 4.002710

O 8.392051 11.784616 5.740083

O 8.398206 9.149182 5.733196

O 6.044888 10.468070 6.486208

O 5.847657 7.450380 6.861830

O 8.379424 10.465673 7.991341

O 8.230063 7.462218 8.293776

O 5.809620 6.126101 9.125168

O 5.814332 8.852400 9.032665

Mg 7.292480 13.453200 0.388370

Mg 4.757060 17.893540 2.261970

Mg 4.757060 14.992060 2.261970

Mg 7.092657 16.440525 4.521143

Mg 9.462072 13.443022 5.178936

Mg 7.120165 11.989431 7.467228

Mg 7.126261 14.898925 7.441816

Mg 4.934401 16.435317 9.274815

Si 7.476970 16.442800 1.221250

Si 6.767610 13.453200 3.313620

Si 9.130844 16.437977 6.393405

Si 5.094353 13.447682 8.449337

O 8.183700 17.805520 0.556660

O 8.183700 15.080070 0.556660

O 5.851310 16.442800 1.386100

O 5.972890 13.453200 1.760420

O 8.215830 16.442800 2.799080

O 8.412830 13.453200 3.191290

O 6.006330 12.125360 4.002710

O 6.006330 14.781030 4.002710

O 8.383073 17.761155 5.699351

O 8.382313 15.114968 5.702097

O 6.026322 16.443521 6.517686

O 5.843689 13.460327 6.889500

O 8.341968 16.439624 7.940614

O 8.222629 13.458539 8.327321

O 5.769741 12.075088 9.093056

O 5.799866 14.796575 9.143732

Mg 12.041480 1.494800 0.388370

Mg 9.506060 5.935140 2.261970

Mg 9.506060 3.033660 2.261970

Mg 11.843424 4.486398 4.520088

Mg 14.215504 1.495262 5.176760

Mg 11.881198 0.048995 7.439463

Mg 11.881937 2.948721 7.438657

Mg 9.677444 4.502771 9.281993

Si 12.225970 4.484400 1.221250

Si 11.516610 1.494800 3.313620

Si 13.886293 4.487225 6.390925

Si 9.858029 1.502819 8.469813

O 12.932710 5.847120 0.556660

O 12.932710 3.121670 0.556660

O 10.600310 4.484400 1.386100

O 10.721890 1.494800 1.760420

O 12.964830 4.484400 2.799080

O 13.161830 1.494800 3.191290

O 10.755330 0.166960 4.002710

O 10.755330 2.822640 4.002710

O 13.135569 5.809034 5.698737

O 13.134089 3.166582 5.696800

O 10.777824 4.485239 6.517156

O 10.593872 1.499481 6.902251

O 13.094449 4.487233 7.937054

O 12.983593 1.499382 8.307268

O 10.562151 0.148142 9.143543

O 10.551556 2.864235 9.140377

Mg 12.041480 7.474000 0.388370

Mg 9.506060 11.914340 2.261970

Mg 9.506060 9.012860 2.261970

Mg 11.837173 10.464872 4.523459

Mg 14.220793 7.477332 5.179251

Mg 11.890078 6.028174 7.439089

Mg 11.872841 8.935300 7.416690

Fe 9.619108 10.462423 9.591764

Si 12.225970 10.463600 1.221250

Si 11.516610 7.474000 3.313620

Si 13.886110 10.466940 6.392027

Si 9.845364 7.468839 8.438945

O 12.932710 11.826320 0.556660

O 12.932710 9.100870 0.556660

O 10.600310 10.463600 1.386100

O 10.721890 7.474000 1.760420

O 12.964840 10.463600 2.799080

O 13.161830 7.474000 3.191290

O 10.755330 6.146160 4.002710

O 10.755330 8.801840 4.002710

O 13.137116 11.789327 5.699032

O 13.139811 9.143594 5.695572

O 10.801444 10.469287 6.477871

O 10.597633 7.457278 6.885948

O 13.092879 10.464317 7.935205

O 12.977553 7.486471 8.309445

O 10.560779 6.139277 9.155799

O 10.536927 8.863281 9.057297

Mg 12.041480 13.453200 0.388370

Mg 9.506060 17.893540 2.261970

Mg 9.506060 14.992060 2.261970

Mg 11.841945 16.441261 4.520569

Mg 14.217567 13.452343 5.178437

Mg 11.869922 11.990570 7.435240

Mg 11.882821 14.896798 7.441685

Mg 9.692189 16.448983 9.274168

Si 12.225970 16.442800 1.221250

Si 11.516610 13.453200 3.313620

Si 13.880619 16.441760 6.394795

Si 9.840518 13.462597 8.462908

O 12.932710 17.805520 0.556660

O 12.932710 15.080070 0.556660

O 10.600310 16.442800 1.386100

O 10.721890 13.453200 1.760420

O 12.964840 16.442800 2.799080

O 13.161830 13.453200 3.191290

O 10.755330 12.125360 4.002710

O 10.755330 14.781030 4.002710

O 13.129580 17.763526 5.700280

O 13.129610 15.118940 5.702430

O 10.771965 16.440417 6.524020

O 10.585883 13.452811 6.906884

O 13.088237 16.441913 7.940646

O 12.973547 13.442064 8.313299

O 10.533676 12.089065 9.132567

O 10.554102 14.804549 9.148882

Mg 16.790480 1.494800 0.388370

Mg 14.255060 5.935140 2.261970

Mg 14.255060 3.033660 2.261970

Mg 16.592640 4.486710 4.521261

Mg 18.965439 1.496745 5.176634

Mg 16.630075 0.047874 7.436798

Mg 16.631914 2.946553 7.437004

Mg 14.436274 4.483971 9.279441

Si 16.974970 4.484400 1.221250

Si 16.265610 1.494800 3.313620

Si 18.630333 4.491738 6.392978

Si 14.604881 1.497837 8.466876

O 17.681710 5.847120 0.556660

O 17.681710 3.121670 0.556660

O 15.349310 4.484400 1.386100

O 15.470890 1.494800 1.760420

O 17.713830 4.484400 2.799080

O 17.910830 1.494800 3.191290

O 15.504330 0.166960 4.002710

O 15.504330 2.822640 4.002710

O 17.879972 5.816022 5.704036

O 17.880215 3.171600 5.695094

O 15.527556 4.489106 6.519764

O 15.339520 1.498482 6.899570

O 17.842091 4.488117 7.939959

O 17.736496 1.497605 8.298860

O 15.306156 0.142169 9.137874

O 15.309569 2.851407 9.139575

Mg 16.790480 7.474000 0.388370

Mg 14.255060 11.914340 2.261970

Mg 14.255060 9.012860 2.261970

Mg 16.592986 10.463758 4.523247

Mg 18.971180 7.480765 5.186816

Mg 16.634936 6.032788 7.449697

Mg 16.634469 8.923716 7.450221

Mg 14.429915 10.463860 9.278982

Si 16.974970 10.463600 1.221250

Si 16.265610 7.474000 3.313620

Si 18.631934 10.466006 6.396816

Si 14.599247 7.478781 8.473347

O 17.681710 11.826320 0.556660

| | | | |
|---|---|---|---|
| O | 17.681710 | 9.100870 | 0.556660 |
| O | 15.349310 | 10.463600 | 1.386100 |
| O | 15.470890 | 7.474000 | 1.760420 |
| O | 17.713840 | 10.463600 | 2.799080 |
| O | 17.910830 | 7.474000 | 3.191290 |
| O | 15.504330 | 6.146160 | 4.002710 |
| O | 15.504330 | 8.801840 | 4.002710 |
| O | 17.880189 | 11.787506 | 5.703658 |
| O | 17.880743 | 9.143776 | 5.703244 |
| O | 15.528416 | 10.465147 | 6.521914 |
| O | 15.341974 | 7.477764 | 6.909332 |
| O | 17.843564 | 10.463798 | 7.944404 |
| O | 17.741918 | 7.478777 | 8.330561 |
| O | 15.300248 | 6.122221 | 9.145896 |
| O | 15.303838 | 8.829574 | 9.151952 |
| Mg | 16.790480 | 13.453200 | 0.388370 |
| Mg | 14.255060 | 17.893540 | 2.261970 |
| Mg | 14.255060 | 14.992060 | 2.261970 |
| Mg | 16.592105 | 16.441496 | 4.520770 |
| Mg | 18.967807 | 13.451600 | 5.180087 |
| Mg | 16.631066 | 12.005994 | 7.446560 |
| Mg | 16.628928 | 14.899523 | 7.444886 |
| Mg | 14.433174 | 16.446167 | 9.280955 |
| Si | 16.974970 | 16.442800 | 1.221250 |
| Si | 16.265610 | 13.453200 | 3.313620 |
| Si | 18.628340 | 16.440723 | 6.392398 |
| Si | 14.596537 | 13.450373 | 8.470681 |
| O | 17.681710 | 17.805520 | 0.556660 |
| O | 17.681710 | 15.080070 | 0.556660 |
| O | 15.349310 | 16.442800 | 1.386100 |
| O | 15.470890 | 13.453200 | 1.760420 |
| O | 17.713840 | 16.442800 | 2.799080 |
| O | 17.910830 | 13.453200 | 3.191290 |
| O | 15.504330 | 12.125360 | 4.002710 |
| O | 15.504330 | 14.781030 | 4.002710 |
| O | 17.878837 | 17.763495 | 5.697203 |
| O | 17.877219 | 15.117425 | 5.701576 |
| O | 15.522554 | 16.442016 | 6.519075 |
| O | 15.336068 | 13.452811 | 6.906027 |
| O | 17.838167 | 16.443787 | 7.938636 |
| O | 17.735000 | 13.453281 | 8.313128 |
| O | 15.302429 | 12.098068 | 9.145633 |
| O | 15.296406 | 14.807921 | 9.141927 |
| C | 10.866456 | 9.202988 | 11.974067 |
| C | 10.160814 | 10.424003 | 11.740888 |
| C | 8.712386 | 10.417898 | 11.701860 |
| C | 8.017510 | 9.148730 | 11.845386 |
| C | 8.763386 | 7.930110 | 11.980744 |
| C | 10.192927 | 7.984544 | 12.053197 |
| C | 8.040106 | 6.691193 | 12.084440 |
| C | 6.655227 | 6.655715 | 12.039430 |
| C | 5.884488 | 7.864897 | 11.925473 |
| C | 4.463708 | 7.855399 | 11.815575 |
| C | 3.739083 | 9.052557 | 11.704005 |

C 4.410876 10.306952 11.759120

C 5.838917 10.363043 11.843556

C 6.583334 9.125207 11.871750

C 6.560746 11.618798 11.814377

C 7.937886 11.644575 11.725230

H 11.958409 9.224971 12.061662

H 10.686196 11.386532 11.787582

H 10.758312 7.052477 12.161230

H 8.608941 5.763917 12.236474

H 6.126039 5.699596 12.126790

H 3.926429 6.900256 11.801028

H 2.652753 9.009403 11.552906

H 3.834340 11.242502 11.788642

H 5.995564 12.555758 11.812985

H 8.467669 12.602758 11.675253

**Pyrene Ni-[010]-fo**

Number of atoms: 362

Mg 2.543480 1.494800 0.388370

Mg 0.008060 5.935140 2.261970

Mg 0.008060 3.033660 2.261970

Mg 2.344504 4.485612 4.517386

Mg 4.721255 1.494736 5.179822

Mg 2.387098 0.048061 7.437498

Mg 2.385661 2.946080 7.434593

Mg 0.188449 4.485005 9.278486

Si 2.727970 4.484400 1.221250

Si 2.018610 1.494800 3.313620

Si 4.383769 4.488207 6.384600

Si 0.361935 1.497783 8.463872

O 3.434710 5.847120 0.556660

O 3.434700 3.121670 0.556660

O 1.102310 4.484400 1.386100

O 1.223890 1.494800 1.760420

O 3.466830 4.484400 2.799080

O 3.663830 1.494800 3.191290

O 1.257330 0.166960 4.002710

O 1.257330 2.822640 4.002710

O 3.630834 5.807683 5.690939

O 3.634070 3.164992 5.693043

O 1.275315 4.489356 6.516874

O 1.097184 1.497624 6.897045

O 3.592341 4.488364 7.929162

O 3.488232 1.498487 8.303747

O 1.062719 0.143433 9.137113

O 1.062984 2.852862 9.136280

Mg 2.543480 7.474000 0.388370

Mg 0.008060 11.914340 2.261970

Mg 0.008060 9.012860 2.261970

Mg 2.358533 10.463461 4.534025

Mg 4.718124 7.482037 5.178005

Mg 2.384408 6.030438 7.437427

Mg 2.393858 8.927882 7.456006

Mg 0.192025 10.466502 9.278930

Si 2.727970 10.463600 1.221250

Si 2.018610 7.474000 3.313620

Si 4.398180 10.465334 6.428105

Si 0.362726 7.477848 8.484617

O 3.434710 11.826320 0.556660

O 3.434710 9.100870 0.556660

O 1.102310 10.463600 1.386100

O 1.223890 7.474000 1.760420

O 3.466830 10.463600 2.799080

O 3.663830 7.474000 3.191290

O 1.257330 6.146160 4.002710

O 1.257330 8.801840 4.002710

O 3.639439 11.782520 5.718912

O 3.638959 9.146521 5.722408

O 1.277066 10.464408 6.530977

O 1.094422 7.483105 6.915503

O 3.604207 10.466871 7.968746

O 3.485328 7.468594 8.315240

O 1.061917 6.118764 9.152266

O 1.065006 8.829046 9.169038

Mg 2.543480 13.453200 0.388370

Mg 0.008060 17.893540 2.261970

Mg 0.008060 14.992060 2.261970

Mg 2.345447 16.442988 4.519249

Mg 4.716999 13.448110 5.173499

Mg 2.390010 12.004372 7.446867

Mg 2.377118 14.905999 7.432236

Mg 0.188945 16.447976 9.279903

Si 2.727970 16.442800 1.221250

Si 2.018610 13.453200 3.313620

Si 4.385179 16.441506 6.389703

Si 0.360546 13.454551 8.475173

O 3.434710 17.805520 0.556660

O 3.434710 15.080070 0.556660

O 1.102310 16.442800 1.386100

O 1.223890 13.453200 1.760420

O 3.466830 16.442800 2.799080

O 3.663830 13.453200 3.191290

O 1.257330 12.125360 4.002710

O 1.257330 14.781030 4.002710

O 3.634531 17.765054 5.697596

O 3.631283 15.123296 5.693134

O 1.274024 16.444472 6.516054

O 1.090553 13.451074 6.906137

O 3.594002 16.441696 7.935350

O 3.477780 13.469063 8.297446

O 1.064272 12.101525 9.151865

O 1.059579 14.813599 9.143383

Mg 7.292480 1.494800 0.388370

Mg 4.757060 5.935140 2.261970

Mg 4.757060 3.033660 2.261970

Mg 7.095000 4.485599 4.517424

Mg 9.469516 1.493089 5.179737

Mg 7.136771 0.045305 7.443320

Mg 7.137930 2.942284 7.440142

Mg 4.944606 4.490049 9.256669

Si 7.476970 4.484400 1.221250

Si 6.767610 1.494800 3.313620

Si 9.134177 4.485782 6.385948

Si 5.108968 1.494919 8.469868

O 8.183700 5.847120 0.556660

O 8.183700 3.121670 0.556660

O 5.851310 4.484400 1.386100

O 5.972890 1.494800 1.760420

O 8.215830 4.484400 2.799080

O 8.412830 1.494800 3.191290

O 6.006330 0.166960 4.002710

O 6.006330 2.822640 4.002710

O 8.381389 5.801739 5.687378

O 8.383612 3.161428 5.696951

O 6.024829 4.481915 6.524360

O 5.846097 1.494360 6.903156

O 8.343875 4.489622 7.932085

O 8.240289 1.495358 8.308838

O 5.807979 0.137744 9.142225

O 5.811287 2.850841 9.142806

Mg 7.292480 7.474000 0.388370

Mg 4.757060 11.914340 2.261970

Mg 4.757060 9.012860 2.261970

Mg 7.102854 10.464770 4.538624

Mg 9.464258 7.480667 5.173939

Mg 7.132371 6.025744 7.430867

Mg 7.129378 8.934267 7.442050

Mg 4.877959 10.455964 9.418450

Si 7.476970 10.463600 1.221250

Si 6.767610 7.474000 3.313620

Si 9.165514 10.464154 6.421099

Si 5.109254 7.484867 8.456265

O 8.183700 11.826320 0.556660

O 8.183700 9.100870 0.556660

O 5.851310 10.463600 1.386100

O 5.972890 7.474000 1.760420

O 8.215830 10.463600 2.799080

O 8.412830 7.474000 3.191290

O 6.006330 6.146160 4.002710

O 6.006330 8.801840 4.002710

O 8.392718 11.779905 5.719586

O 8.394162 9.147088 5.721756

O 6.043853 10.464988 6.492424

O 5.839710 7.455043 6.890906

O 8.385041 10.468378 7.964090

O 8.221861 7.466883 8.295781

O 5.806566 6.136976 9.170995

O 5.812369 8.858057 9.073463

Mg 7.292480 13.453200 0.388370

Mg 4.757060 17.893540 2.261970

Mg 4.757060 14.992060 2.261970

Mg 7.094292 16.440274 4.521183

Mg 9.466041 13.442269 5.176184

Mg 7.128388 11.992704 7.442113

Mg 7.139674 14.895722 7.441159

Mg 4.947101 16.431875 9.266923

Si 7.476970 16.442800 1.221250

Si 6.767610 13.453200 3.313620

Si 9.133573 16.437916 6.392482

Si 5.099091 13.449954 8.435727

O 8.183700 17.805520 0.556660

O 8.183700 15.080070 0.556660

O 5.851310 16.442800 1.386100

O 5.972890 13.453200 1.760420

O 8.215830 16.442800 2.799080

O 8.412830 13.453200 3.191290

O 6.006330 12.125360 4.002710

O 6.006330 14.781030 4.002710

O 8.384431 17.761769 5.700332

O 8.383400 15.116864 5.699540

O 6.027114 16.442686 6.524264

O 5.846237 13.469877 6.879450

O 8.345942 16.438747 7.940958

O 8.228552 13.447379 8.316415

O 5.784753 12.079476 9.075367

O 5.809146 14.787261 9.153846

Mg 12.041480 1.494800 0.388370

Mg 9.506060 5.935140 2.261970

Mg 9.506060 3.033660 2.261970

Mg 11.843753 4.488158 4.519638

Mg 14.216256 1.496250 5.178394

Mg 11.883473 0.048396 7.439937

Mg 11.883163 2.947418 7.438492

Mg 9.698171 4.512017 9.259691

Si 12.225970 4.484400 1.221250

Si 11.516610 1.494800 3.313620

Si 13.882726 4.490879 6.393214

Si 9.860395 1.500097 8.473013

O 12.932710 5.847120 0.556660

O 12.932710 3.121670 0.556660

O 10.600310 4.484400 1.386100

O 10.721890 1.494800 1.760420

O 12.964830 4.484400 2.799080

O 13.161830 1.494800 3.191290

O 10.755330 0.166960 4.002710

O 10.755330 2.822640 4.002710

O 13.132369 5.811152 5.699726

O 13.132350 3.168581 5.699404

O 10.774449 4.488224 6.526685

O 10.594600 1.497681 6.904168

O 13.089736 4.491872 7.939613

O 12.984445 1.499393 8.308176

O 10.562114 0.142450 9.144081

O 10.555713 2.863340 9.140614

Mg 12.041480 7.474000 0.388370

Mg 9.506060 11.914340 2.261970

Mg 9.506060 9.012860 2.261970
Mg 11.838105 10.464826 4.523459
Mg 14.218471 7.479038 5.179415
Mg 11.887088 6.039034 7.444426
Mg 11.862694 8.947266 7.424590
Ni 9.527853 10.474795 9.557047
Si 12.225970 10.463600 1.221250
Si 11.516610 7.474000 3.313620
Si 13.888604 10.464524 6.387284
Si 9.839236 7.500597 8.440490
O 12.932710 11.826320 0.556660
O 12.932710 9.100870 0.556660
O 10.600310 10.463600 1.386100
O 10.721890 7.474000 1.760420
O 12.964840 10.463600 2.799080
O 13.161830 7.474000 3.191290
O 10.755330 6.146160 4.002710
O 10.755330 8.801840 4.002710
O 13.142057 11.787726 5.694961
O 13.141453 9.141651 5.692452
O 10.808361 10.468208 6.447313
O 10.593642 7.457976 6.885477
O 13.100324 10.462474 7.928556
O 12.974544 7.492886 8.318578
O 10.551875 6.167034 9.170461
O 10.526774 8.899307 9.039269
Mg 12.041480 13.453200 0.388370
Mg 9.506060 17.893540 2.261970
Mg 9.506060 14.992060 2.261970
Mg 11.843345 16.440209 4.520272
Mg 14.217426 13.450569 5.179280
Mg 11.866108 11.979622 7.436463
Mg 11.883549 14.889854 7.440847
Mg 9.707907 16.431764 9.263801
Si 12.225970 16.442800 1.221250
Si 11.516610 13.453200 3.313620
Si 13.880942 16.440886 6.395683
Si 9.849405 13.430213 8.455789
O 12.932710 17.805520 0.556660
O 12.932710 15.080070 0.556660
O 10.600310 16.442800 1.386100
O 10.721890 13.453200 1.760420
O 12.964840 16.442800 2.799080
O 13.161830 13.453200 3.191290
O 10.755330 12.125360 4.002710
O 10.755330 14.781030 4.002710
O 13.130803 17.764120 5.702580
O 13.131124 15.119750 5.700934
O 10.774113 16.440002 6.528833
O 10.590250 13.454852 6.894192
O 13.087276 16.439315 7.941259
O 12.975942 13.439524 8.310671
O 10.558611 12.058087 9.103132
O 10.552385 14.776952 9.157169

| | | | |
|---|---|---|---|
| Mg | 16.790480 | 1.494800 | 0.388370 |
| Mg | 14.255060 | 5.935140 | 2.261970 |
| Mg | 14.255060 | 3.033660 | 2.261970 |
| Mg | 16.592328 | 4.486822 | 4.520521 |
| Mg | 18.965725 | 1.495924 | 5.177161 |
| Mg | 16.630766 | 0.047353 | 7.438587 |
| Mg | 16.631747 | 2.945732 | 7.437801 |
| Mg | 14.436698 | 4.485056 | 9.277055 |
| Si | 16.974970 | 4.484400 | 1.221250 |
| Si | 16.265610 | 1.494800 | 3.313620 |
| Si | 18.630242 | 4.490862 | 6.392112 |
| Si | 14.605610 | 1.497552 | 8.469452 |
| O | 17.681710 | 5.847120 | 0.556660 |
| O | 17.681710 | 3.121670 | 0.556660 |
| O | 15.349310 | 4.484400 | 1.386100 |
| O | 15.470890 | 1.494800 | 1.760420 |
| O | 17.713830 | 4.484400 | 2.799080 |
| O | 17.910830 | 1.494800 | 3.191290 |
| O | 15.504330 | 0.166960 | 4.002710 |
| O | 15.504330 | 2.822640 | 4.002710 |
| O | 17.878278 | 5.813224 | 5.701425 |
| O | 17.879786 | 3.169831 | 5.695530 |
| O | 15.524261 | 4.489035 | 6.522921 |
| O | 15.339847 | 1.497459 | 6.901741 |
| O | 17.839933 | 4.487792 | 7.938560 |
| O | 17.737326 | 1.497586 | 8.301242 |
| O | 15.306134 | 0.142213 | 9.141860 |
| O | 15.309386 | 2.851777 | 9.141727 |
| Mg | 16.790480 | 7.474000 | 0.388370 |
| Mg | 14.255060 | 11.914340 | 2.261970 |
| Mg | 14.255060 | 9.012860 | 2.261970 |
| Mg | 16.592314 | 10.464074 | 4.524540 |
| Mg | 18.970488 | 7.479467 | 5.183949 |
| Mg | 16.630505 | 6.032003 | 7.446938 |
| Mg | 16.631383 | 8.925398 | 7.447274 |
| Mg | 14.428303 | 10.464635 | 9.276641 |
| Si | 16.974970 | 10.463600 | 1.221250 |
| Si | 16.265610 | 7.474000 | 3.313620 |
| Si | 18.632779 | 10.465937 | 6.396677 |
| Si | 14.596943 | 7.482202 | 8.477410 |
| O | 17.681710 | 11.826320 | 0.556660 |
| O | 17.681710 | 9.100870 | 0.556660 |
| O | 15.349310 | 10.463600 | 1.386100 |
| O | 15.470890 | 7.474000 | 1.760420 |
| O | 17.713840 | 10.463600 | 2.799080 |
| O | 17.910830 | 7.474000 | 3.191290 |
| O | 15.504330 | 6.146160 | 4.002710 |
| O | 15.504330 | 8.801840 | 4.002710 |
| O | 17.881824 | 11.788119 | 5.703400 |
| O | 17.882322 | 9.143701 | 5.702890 |
| O | 15.532140 | 10.464322 | 6.517788 |
| O | 15.337023 | 7.478239 | 6.911614 |
| O | 17.842005 | 10.465639 | 7.943134 |
| O | 17.737549 | 7.478136 | 8.318967 |

O 15.298248 6.124265 9.148950

O 15.305488 8.830738 9.157113

Mg 16.790480 13.453200 0.388370

Mg 14.255060 17.893540 2.261970

Mg 14.255060 14.992060 2.261970

Mg 16.591769 16.441004 4.520973

Mg 18.967563 13.451226 5.180089

Mg 16.630612 12.005455 7.445144

Mg 16.629583 14.898948 7.447222

Mg 14.435131 16.444355 9.278400

Si 16.974970 16.442800 1.221250

Si 16.265610 13.453200 3.313620

Si 18.628855 16.440890 6.393597

Si 14.597228 13.448678 8.470192

O 17.681710 17.805520 0.556660

O 17.681710 15.080070 0.556660

O 15.349310 16.442800 1.386100

O 15.470890 13.453200 1.760420

O 17.713840 16.442800 2.799080

O 17.910830 13.453200 3.191290

O 15.504330 12.125360 4.002710

O 15.504330 14.781030 4.002710

O 17.879223 17.763329 5.698515

O 17.877890 15.117371 5.703556

O 15.523037 16.442393 6.522970

O 15.338317 13.453004 6.905762

O 17.839426 16.443599 7.940419

O 17.735133 13.452496 8.316994

O 15.304501 12.097188 9.145690

O 15.298038 14.805985 9.143279

C 11.194001 9.190290 11.855518

C 10.610400 10.468412 11.697904

C 9.182617 10.610937 11.698977

C 8.357872 9.428996 11.855451

C 8.977008 8.135114 11.963476

C 10.396361 8.039638 11.961890

C 8.125653 6.974544 12.064812

C 6.748929 7.088825 12.073843

C 6.105718 8.378651 12.004228

C 4.692824 8.516366 12.011387

C 4.094660 9.791267 11.920036

C 4.894977 10.958853 11.823397

C 6.320380 10.860712 11.797966

C 6.928381 9.558237 11.895347

C 7.160464 12.026906 11.618288

C 8.537017 11.909852 11.583131

H 12.284750 9.085787 11.857488

H 11.224862 11.368916 11.578845

H 10.866893 7.051211 12.002162

H 8.596730 5.988115 12.159757

H 6.119129 6.195976 12.145224

H 4.060285 7.624609 12.073080

H 3.001587 9.876180 11.935957

H 4.428267 11.950688 11.785745

H 6.690355 13.005711 11.470938

H 9.171413 12.793240 11.448807

**Pyrene V$_{MgO}$-[010]-fo (=)**

Number of atoms: 360

Mg 2.543480 1.494800 0.388370

Mg 0.008060 5.935140 2.261970

Mg 0.008060 3.033660 2.261970

Mg 2.344478 4.484288 4.511983

Mg 4.711944 1.488747 5.170423

Mg 2.383058 0.049113 7.430810

Mg 2.380687 2.942063 7.431207

Mg 0.175949 4.479268 9.276568

Si 2.727970 4.484400 1.221250

Si 2.018610 1.494800 3.313620

Si 4.374675 4.484020 6.379889

Si 0.356048 1.493849 8.463594

O 3.434710 5.847120 0.556660

O 3.434700 3.121670 0.556660

O 1.102310 4.484400 1.386100

O 1.223890 1.494800 1.760420

O 3.466830 4.484400 2.799080

O 3.663830 1.494800 3.191290

O 1.257330 0.166960 4.002710

O 1.257330 2.822640 4.002710

O 3.629128 5.807652 5.688595

O 3.626001 3.161097 5.689481

O 1.278490 4.484932 6.506744

O 1.090880 1.497757 6.896525

O 3.588679 4.483359 7.925533

O 3.479820 1.501338 8.309289

O 1.057784 0.141327 9.135923

O 1.056486 2.851656 9.134620

Mg 2.543480 7.474000 0.388370

Mg 0.008060 11.914340 2.261970

Mg 0.008060 9.012860 2.261970

Mg 2.363396 10.467008 4.535976

Mg 4.714903 7.493445 5.176298

Mg 2.372244 6.024678 7.427396

Mg 2.386880 8.925747 7.452468

Mg 0.190601 10.468703 9.272535

Si 2.727970 10.463600 1.221250

Si 2.018610 7.474000 3.313620

Si 4.396931 10.466729 6.434445

Si 0.348597 7.473302 8.472453

O 3.434710 11.826320 0.556660

O 3.434710 9.100870 0.556660

O 1.102310 10.463600 1.386100

O 1.223890 7.474000 1.760420

O 3.466830 10.463600 2.799080

O 3.663830 7.474000 3.191290

O 1.257330 6.146160 4.002710

O 1.257330 8.801840 4.002710

O 3.633769 11.779794 5.722734

O 3.634225 9.146943 5.726650

O 1.278410 10.461476 6.523423

O 1.081616 7.478382 6.906149

O 3.603611 10.460239 7.981614

O 3.466693 7.454716 8.298838

O 1.044702 6.113610 9.142037

O 1.044919 8.822137 9.158501

Mg 2.543480 13.453200 0.388370

Mg 0.008060 17.893540 2.261970

Mg 0.008060 14.992060 2.261970

Mg 2.347280 16.439845 4.516148

Mg 4.733124 13.439426 5.188809

Mg 2.396071 12.006755 7.450747

Mg 2.380942 14.904450 7.424840

Mg 0.185843 16.443279 9.276023

Si 2.727970 16.442800 1.221250

Si 2.018610 13.453200 3.313620

Si 4.381680 16.443980 6.383681

Si 0.363432 13.455272 8.468233

O 3.434710 17.805520 0.556660

O 3.434710 15.080070 0.556660

O 1.102310 16.442800 1.386100

O 1.223890 13.453200 1.760420

O 3.466830 16.442800 2.799080

O 3.663830 13.453200 3.191290

O 1.257330 12.125360 4.002710

O 1.257330 14.781030 4.002710

O 3.627217 17.766787 5.689626

O 3.634263 15.121762 5.690412

O 1.279698 16.445829 6.504539

O 1.089450 13.448591 6.898938

O 3.587757 16.452659 7.927062

O 3.480869 13.475058 8.281253

O 1.064824 12.101375 9.146813

O 1.063435 14.814055 9.135620

Mg 7.292480 1.494800 0.388370

Mg 4.757060 5.935140 2.261970

Mg 4.757060 3.033660 2.261970

Mg 7.081893 4.482901 4.507452

Mg 9.464793 1.492436 5.170697

Mg 7.126734 0.050420 7.426337

Mg 7.115291 2.944303 7.412546

Mg 4.905360 4.481591 9.292916

Si 7.476970 4.484400 1.221250

Si 6.767610 1.494800 3.313620

Si 9.130161 4.502345 6.388736

Si 5.101247 1.499630 8.467978

O 8.183700 5.847120 0.556660

O 8.183700 3.121670 0.556660

O 5.851310 4.484400 1.386100

O 5.972890 1.494800 1.760420

O 8.215830 4.484400 2.799080

O 8.412830 1.494800 3.191290

O 6.006330 0.166960 4.002710

O 6.006330 2.822640 4.002710

O 8.395108 5.850087 5.729515

O 8.386691 3.189797 5.680620

O 6.020235 4.475641 6.486434

O 5.830477 1.499380 6.896587

O 8.316014 4.490554 7.926788

O 8.231528 1.516015 8.294586

O 5.805577 0.149821 9.141181

O 5.803931 2.861111 9.133352

Mg 7.292480 7.474000 0.388370

Mg 4.757060 11.914340 2.261970

Mg 4.757060 9.012860 2.261970

Mg 7.126919 10.475425 4.571665

Mg 9.407903 7.524503 5.130861

Mg 7.072461 5.999280 7.418628

Mg 7.124602 8.947978 7.459478

Mg 4.924784 10.455271 9.394819

Si 7.476970 10.463600 1.221250

Si 6.767610 7.474000 3.313620

Si 9.150771 10.445519 6.506709

Si 5.086604 7.468419 8.448442

O 8.183700 11.826320 0.556660

O 8.183700 9.100870 0.556660

O 5.851310 10.463600 1.386100

O 5.972890 7.474000 1.760420

O 8.215830 10.463600 2.799080

O 8.412830 7.474000 3.191290

O 6.006330 6.146160 4.002710

O 6.006330 8.801840 4.002710

O 8.413835 11.764489 5.745863

O 8.391172 9.134489 5.721513

O 6.034853 10.468376 6.518369

O 5.839421 7.465605 6.888305

O 8.389546 10.388298 7.988978

O 8.148629 7.414981 8.422068

O 5.787253 6.108380 9.131569

O 5.786732 8.818524 9.099143

Mg 7.292480 13.453200 0.388370

Mg 4.757060 17.893540 2.261970

Mg 4.757060 14.992060 2.261970

Mg 7.095768 16.437140 4.520234

Mg 9.459602 13.436795 5.202672

Mg 7.194526 11.982775 7.523829

Mg 7.136199 14.906989 7.427049

Mg 4.930434 16.450153 9.268297

Si 7.476970 16.442800 1.221250

Si 6.767610 13.453200 3.313620

Si 9.134547 16.449963 6.382863

Si 5.104265 13.464623 8.432512

O 8.183700 17.805520 0.556660

O 8.183700 15.080070 0.556660

O 5.851310 16.442800 1.386100

O 5.972890 13.453200 1.760420

O 8.215830 16.442800 2.799080

O 8.412830 13.453200 3.191290

O 6.006330 12.125360 4.002710

O 6.006330 14.781030 4.002710

O 8.378141 17.777499 5.691779

O 8.377138 15.138554 5.681916

O 6.021755 16.453157 6.508423

O 5.864278 13.445906 6.886676

O 8.337891 16.465683 7.923893

O 8.217957 13.517672 8.330710

O 5.767704 12.109506 9.118215

O 5.794904 14.820806 9.120186

Mg 12.041480 1.494800 0.388370

Mg 9.506060 5.935140 2.261970

Mg 9.506060 3.033660 2.261970

Mg 11.838147 4.480609 4.514153

Mg 14.211492 1.497071 5.176816

Mg 11.878653 0.041682 7.427591

Mg 11.872195 2.953849 7.446689

Mg 9.571963 4.373754 9.328221

Si 12.225970 4.484400 1.221250

Si 11.516610 1.494800 3.313620

Si 13.873928 4.502263 6.398376

Si 9.851967 1.477078 8.459184

O 12.932710 5.847120 0.556660

O 12.932710 3.121670 0.556660

O 10.600310 4.484400 1.386100

O 10.721890 1.494800 1.760420

O 12.964830 4.484400 2.799080

O 13.161830 1.494800 3.191290

O 10.755330 0.166960 4.002710

O 10.755330 2.822640 4.002710

O 13.112562 5.840945 5.731578

O 13.124149 3.189154 5.694295

O 10.771091 4.520609 6.512626

O 10.591192 1.510841 6.896517

O 13.087839 4.494353 7.948050

O 12.975196 1.506779 8.306752

O 10.546341 0.126898 9.127654

O 10.556367 2.823088 9.159111

Mg 12.041480 7.474000 0.388370

Mg 9.506060 11.914340 2.261970

Mg 9.506060 9.012860 2.261970

Mg 11.822589 10.493870 4.522044

Mg 14.235594 7.467530 5.151044

Mg 11.882101 6.027554 7.465082

Mg 11.948753 8.939121 7.259108

Si 12.225586 10.460662 1.229815

Si 11.516610 7.474000 3.313620

Si 13.888210 10.463600 6.382630

Si 9.733830 7.310619 8.556366

O 12.929633 11.815376 0.556123

O 12.932710 9.100870 0.556660

O 10.600310 10.463600 1.386100

O 10.721890 7.474000 1.760420

| | | | |
|---|---|---|---|
| O | 12.964840 | 10.463600 | 2.799080 |
| O | 13.161830 | 7.474000 | 3.191290 |
| O | 10.755330 | 6.146160 | 4.002710 |
| O | 10.755330 | 8.801840 | 4.002710 |
| O | 13.128110 | 11.791550 | 5.692940 |
| O | 13.164972 | 9.133989 | 5.655698 |
| O | 10.812967 | 10.417925 | 6.459250 |
| O | 10.537720 | 7.602881 | 7.110782 |
| O | 13.089132 | 10.429746 | 7.922494 |
| O | 12.941021 | 7.525904 | 8.354693 |
| O | 10.551193 | 5.968852 | 9.087259 |
| Mg | 12.058408 | 13.451880 | 0.417597 |
| Mg | 9.506458 | 17.889855 | 2.259729 |
| Mg | 9.506060 | 14.992060 | 2.261970 |
| Mg | 11.837740 | 16.442800 | 4.520030 |
| Mg | 14.211300 | 13.453200 | 5.174280 |
| Mg | 11.800177 | 11.976814 | 7.495409 |
| Mg | 11.871860 | 14.892987 | 7.441511 |
| Mg | 9.665902 | 16.429528 | 9.277541 |
| Si | 12.223702 | 16.441515 | 1.231780 |
| Si | 11.509076 | 13.454960 | 3.316766 |
| Si | 13.888220 | 16.442800 | 6.382630 |
| Si | 9.847270 | 13.453200 | 8.472360 |
| O | 12.921499 | 17.801814 | 0.558950 |
| O | 12.929841 | 15.087905 | 0.558799 |
| O | 10.600310 | 16.442800 | 1.386100 |
| O | 10.721890 | 13.453200 | 1.760420 |
| O | 12.964840 | 16.442800 | 2.799080 |
| O | 13.161830 | 13.453200 | 3.191290 |
| O | 10.755330 | 12.125360 | 4.002710 |
| O | 10.755330 | 14.781030 | 4.002710 |
| O | 13.128120 | 17.770750 | 5.692940 |
| O | 13.128120 | 15.114850 | 5.692940 |
| O | 10.775595 | 16.464621 | 6.496813 |
| O | 10.580905 | 13.493141 | 6.874137 |
| O | 13.095556 | 16.445607 | 7.925645 |
| O | 12.984187 | 13.438696 | 8.306489 |
| O | 10.519190 | 12.083760 | 9.054891 |
| O | 10.569658 | 14.830764 | 9.122229 |
| Mg | 16.804019 | 1.493255 | 0.410441 |
| Mg | 14.254376 | 5.934010 | 2.249254 |
| Mg | 14.255060 | 3.033660 | 2.261970 |
| Mg | 16.586740 | 4.484400 | 4.520030 |
| Mg | 18.960290 | 1.494800 | 5.174280 |
| Mg | 16.625864 | 0.044097 | 7.433913 |
| Mg | 16.630482 | 2.939702 | 7.433169 |
| Mg | 14.432413 | 4.466941 | 9.286826 |
| Si | 16.973513 | 4.484191 | 1.231711 |
| Si | 16.260858 | 1.492211 | 3.310133 |
| Si | 18.637210 | 4.484400 | 6.382630 |
| Si | 14.596270 | 1.494800 | 8.472360 |
| O | 17.672963 | 5.842388 | 0.561161 |
| O | 17.674485 | 3.128238 | 0.559509 |
| O | 15.349310 | 4.484400 | 1.386100 |

O 15.470890 1.494800 1.760420

O 17.713830 4.484400 2.799080

O 17.910830 1.494800 3.191290

O 15.504330 0.166960 4.002710

O 15.504330 2.822640 4.002710

O 17.877110 5.812340 5.692940

O 17.877110 3.156450 5.692940

O 15.515235 4.496341 6.517309

O 15.331264 1.501508 6.903026

O 17.837336 4.484236 7.926304

O 17.731060 1.495435 8.302553

O 15.294056 0.137077 9.138771

O 15.310993 2.844855 9.146345

Mg 16.802545 7.476416 0.406255

Mg 14.252080 11.914394 2.260961

Mg 14.255060 9.012860 2.261970

Mg 16.586740 10.463600 4.520030

Mg 18.960300 7.474000 5.174280

Mg 16.630077 6.025447 7.432051

Mg 16.630192 8.924543 7.438182

Mg 14.431811 10.474225 9.266339

Si 16.973126 10.465063 1.232205

Si 16.263550 7.475925 3.305515

Si 18.637210 10.463600 6.382630

Si 14.596270 7.474000 8.472360

O 17.675326 11.819330 0.558696

O 17.674683 9.107884 0.560569

O 15.349310 10.463600 1.386100

O 15.470890 7.474000 1.760420

O 17.713840 10.463600 2.799080

O 17.910830 7.474000 3.191290

O 15.504330 6.146160 4.002710

O 15.504330 8.801840 4.002710

O 17.877120 11.791540 5.692940

O 17.877110 9.135650 5.692940

O 15.528082 10.455468 6.518419

O 15.313631 7.468450 6.907442

O 17.843324 10.461945 7.930359

O 17.720508 7.469120 8.305964

O 15.272935 6.115462 9.148141

O 15.293919 8.826513 9.144879

Mg 16.804666 13.453769 0.412261

Mg 14.252503 17.891161 2.255651

Mg 14.255060 14.992060 2.261970

Mg 16.586740 16.442800 4.520030

Mg 18.960300 13.453200 5.174280

Mg 16.634715 12.011197 7.440517

Mg 16.630581 14.899873 7.440128

Mg 14.420155 16.436362 9.282258

Si 16.973465 16.442387 1.232254

Si 16.260711 13.453361 3.311182

Si 18.637220 16.442800 6.382630

Si 14.596270 13.453200 8.472360

O 17.675848 17.798293 0.559501

O 17.675840 15.087642 0.559081
O 15.349310 16.442800 1.386100
O 15.470890 13.453200 1.760420
O 17.713840 16.442800 2.799080
O 17.910830 13.453200 3.191290
O 15.504330 12.125360 4.002710
O 15.504330 14.781030 4.002710
O 17.877120 17.770750 5.692940
O 17.877120 15.114850 5.692940
O 15.530426 16.446694 6.504773
O 15.342090 13.453593 6.902190
O 17.844593 16.444853 7.928935
O 17.738052 13.455049 8.313259
O 15.314456 12.097626 9.138375
O 15.306882 14.810472 9.139634
C 10.350165 8.541954 10.334935
C 9.568053 9.750098 10.337705
C 8.303832 9.734083 10.991197
C 7.772138 8.502580 11.513574
C 8.608574 7.328656 11.607765
C 9.934225 7.418790 11.150524
C 8.040006 6.094189 12.106186
C 6.691515 6.002842 12.388782
C 5.835049 7.169781 12.318603
C 4.446876 7.110275 12.601656
C 3.657554 8.278575 12.584540
C 4.238710 9.538695 12.339207
C 5.623170 9.641972 12.017742
C 6.415570 8.436468 11.961163
C 6.248913 10.899217 11.675302
C 7.522201 10.937178 11.120387
H 11.423292 8.591071 10.079162
H 9.956513 10.703647 9.869055
H 10.635408 6.594353 11.305303
H 8.696109 5.222130 12.244946
H 6.261061 5.051700 12.726005
H 3.987678 6.145882 12.849584
H 2.583511 8.208339 12.785834
H 3.630388 10.449773 12.408713
H 5.681092 11.831075 11.783551
H 7.932260 11.880637 10.741951

**Pyrene V$_{MgO}$-[010]-fo (⊥)**

Number of atoms: 360

Mg 2.543480 1.494800 0.388370
Mg 0.008060 5.935140 2.261970
Mg 0.008060 3.033660 2.261970
Mg 2.341706 4.486552 4.518813
Mg 4.714427 1.490120 5.179027
Mg 2.374423 0.043228 7.440107
Mg 2.371749 2.943532 7.434662
Mg 0.174232 4.486693 9.281662
Si 2.727970 4.484400 1.221250
Si 2.018610 1.494800 3.313620

Si 4.375006 4.482005 6.391970
Si 0.348359 1.495923 8.466913
O 3.434710 5.847120 0.556660
O 3.434700 3.121670 0.556660
O 1.102310 4.484400 1.386100
O 1.223890 1.494800 1.760420
O 3.466830 4.484400 2.799080
O 3.663830 1.494800 3.191290
O 1.257330 0.166960 4.002710
O 1.257330 2.822640 4.002710
O 3.630897 5.807269 5.699885
O 3.625198 3.160777 5.696334
O 1.263700 4.489025 6.519726
O 1.085196 1.494251 6.899959
O 3.582500 4.480098 7.934464
O 3.476312 1.493939 8.304645
O 1.050113 0.141946 9.140190
O 1.048073 2.853767 9.134949
Mg 2.543480 7.474000 0.388370
Mg 0.008060 11.914340 2.261970
Mg 0.008060 9.012860 2.261970
Mg 2.349941 10.464389 4.529018
Mg 4.724514 7.482027 5.189807
Mg 2.378531 6.030761 7.445080
Mg 2.380521 8.926610 7.447932
Mg 0.182869 10.466884 9.294472
Si 2.727970 10.463600 1.221250
Si 2.018610 7.474000 3.313620
Si 4.394702 10.460973 6.409584
Si 0.347634 7.480549 8.474194
O 3.434710 11.826320 0.556660
O 3.434710 9.100870 0.556660
O 1.102310 10.463600 1.386100
O 1.223890 7.474000 1.760420
O 3.466830 10.463600 2.799080
O 3.663830 7.474000 3.191290
O 1.257330 6.146160 4.002710
O 1.257330 8.801840 4.002710
O 3.640764 11.779537 5.707994
O 3.638146 9.144412 5.709478
O 1.279714 10.466388 6.522423
O 1.086535 7.477409 6.908215
O 3.597223 10.464242 7.951685
O 3.482778 7.480603 8.311506
O 1.044867 6.120824 9.143428
O 1.053379 8.831637 9.148813
Mg 2.543480 13.453200 0.388370
Mg 0.008060 17.893540 2.261970
Mg 0.008060 14.992060 2.261970
Mg 2.342134 16.439864 4.522209
Mg 4.723607 13.440689 5.187722
Mg 2.386687 12.003266 7.451614
Mg 2.386965 14.895096 7.451706
Mg 0.184084 16.441922 9.283787

| | | | |
|---|---|---|---|
| Si | 2.727970 | 16.442800 | 1.221250 |
| Si | 2.018610 | 13.453200 | 3.313620 |
| Si | 4.378357 | 16.438206 | 6.396234 |
| Si | 0.356269 | 13.452783 | 8.477040 |
| O | 3.434710 | 17.805520 | 0.556660 |
| O | 3.434710 | 15.080070 | 0.556660 |
| O | 1.102310 | 16.442800 | 1.386100 |
| O | 1.223890 | 13.453200 | 1.760420 |
| O | 3.466830 | 16.442800 | 2.799080 |
| O | 3.663830 | 13.453200 | 3.191290 |
| O | 1.257330 | 12.125360 | 4.002710 |
| O | 1.257330 | 14.781030 | 4.002710 |
| O | 3.627696 | 17.758302 | 5.699276 |
| O | 3.634120 | 15.111664 | 5.706347 |
| O | 1.271393 | 16.437972 | 6.523142 |
| O | 1.096192 | 13.451205 | 6.910810 |
| O | 3.590582 | 16.442682 | 7.942289 |
| O | 3.495354 | 13.446431 | 8.324313 |
| O | 1.058826 | 12.099061 | 9.151266 |
| O | 1.060727 | 14.810296 | 9.145786 |
| Mg | 7.292480 | 1.494800 | 0.388370 |
| Mg | 4.757060 | 5.935140 | 2.261970 |
| Mg | 4.757060 | 3.033660 | 2.261970 |
| Mg | 7.090971 | 4.483762 | 4.517852 |
| Mg | 9.466850 | 1.490703 | 5.179793 |
| Mg | 7.131293 | 0.033017 | 7.452253 |
| Mg | 7.121688 | 2.928287 | 7.437354 |
| Mg | 4.907613 | 4.470538 | 9.294542 |
| Si | 7.476970 | 4.484400 | 1.221250 |
| Si | 6.767610 | 1.494800 | 3.313620 |
| Si | 9.120961 | 4.482061 | 6.408727 |
| Si | 5.096945 | 1.486355 | 8.470040 |
| O | 8.183700 | 5.847120 | 0.556660 |
| O | 8.183700 | 3.121670 | 0.556660 |
| O | 5.851310 | 4.484400 | 1.386100 |
| O | 5.972890 | 1.494800 | 1.760420 |
| O | 8.215830 | 4.484400 | 2.799080 |
| O | 8.412830 | 1.494800 | 3.191290 |
| O | 6.006330 | 0.166960 | 4.002710 |
| O | 6.006330 | 2.822640 | 4.002710 |
| O | 8.384042 | 5.812716 | 5.718698 |
| O | 8.381774 | 3.164960 | 5.697024 |
| O | 6.018955 | 4.472508 | 6.506572 |
| O | 5.836865 | 1.481166 | 6.903733 |
| O | 8.324811 | 4.465547 | 7.947583 |
| O | 8.233311 | 1.482830 | 8.311850 |
| O | 5.798755 | 0.132322 | 9.145115 |
| O | 5.798381 | 2.845126 | 9.137652 |
| Mg | 7.292480 | 7.474000 | 0.388370 |
| Mg | 4.757060 | 11.914340 | 2.261970 |
| Mg | 4.757060 | 9.012860 | 2.261970 |
| Mg | 7.112834 | 10.460513 | 4.537600 |
| Mg | 9.445558 | 7.487985 | 5.202790 |
| Mg | 7.091595 | 6.000053 | 7.438183 |

Mg 7.177669 8.928058 7.481640

Mg 4.934490 10.458753 9.300497

Si 7.476970 10.463600 1.221250

Si 6.767610 7.474000 3.313620

Si 9.162804 10.443627 6.412640

Si 5.105478 7.476786 8.479491

O 8.183700 11.826320 0.556660

O 8.183700 9.100870 0.556660

O 5.851310 10.463600 1.386100

O 5.972890 7.474000 1.760420

O 8.215830 10.463600 2.799080

O 8.412830 7.474000 3.191290

O 6.006330 6.146160 4.002710

O 6.006330 8.801840 4.002710

O 8.401264 11.762792 5.688250

O 8.382030 9.129656 5.706541

O 6.036674 10.454015 6.552013

O 5.842802 7.488632 6.914403

O 8.474367 10.458762 7.952599

O 8.152604 7.397756 8.368179

O 5.777055 6.101603 9.141003

O 5.811985 8.822286 9.175223

Mg 7.292480 13.453200 0.388370

Mg 4.757060 17.893540 2.261970

Mg 4.757060 14.992060 2.261970

Mg 7.093619 16.444003 4.518685

Mg 9.442112 13.441702 5.142598

Mg 7.167691 11.964123 7.463209

Mg 7.108466 14.900097 7.411722

Mg 4.930988 16.431841 9.287705

Si 7.476970 16.442800 1.221250

Si 6.767610 13.453200 3.313620

Si 9.132185 16.436282 6.389230

Si 5.118960 13.436708 8.484157

O 8.183700 17.805520 0.556660

O 8.183700 15.080070 0.556660

O 5.851310 16.442800 1.386100

O 5.972890 13.453200 1.760420

O 8.215830 16.442800 2.799080

O 8.412830 13.453200 3.191290

O 6.006330 12.125360 4.002710

O 6.006330 14.781030 4.002710

O 8.379879 17.763842 5.706895

O 8.374555 15.123214 5.684426

O 6.021869 16.439786 6.515711

O 5.843198 13.415094 6.914062

O 8.350455 16.421442 7.939543

O 8.215638 13.473917 8.257274

O 5.823346 12.093300 9.188149

O 5.816227 14.806442 9.136605

Mg 12.041480 1.494800 0.388370

Mg 9.506060 5.935140 2.261970

Mg 9.506060 3.033660 2.261970

Mg 11.841134 4.486387 4.523336

Mg 14.211740 1.508945 5.183997

Mg 11.881144 0.039664 7.434049

Mg 11.870018 2.948201 7.457426

Mg 9.637487 4.412943 9.318804

Si 12.225970 4.484400 1.221250

Si 11.516610 1.494800 3.313620

Si 13.856112 4.517153 6.414717

Si 9.854046 1.462270 8.474638

O 12.932710 5.847120 0.556660

O 12.932710 3.121670 0.556660

O 10.600310 4.484400 1.386100

O 10.721890 1.494800 1.760420

O 12.964830 4.484400 2.799080

O 13.161830 1.494800 3.191290

O 10.755330 0.166960 4.002710

O 10.755330 2.822640 4.002710

O 13.098430 5.843294 5.729919

O 13.119967 3.190758 5.709470

O 10.761017 4.509170 6.538622

O 10.588892 1.492422 6.906674

O 13.067417 4.502854 7.961413

O 12.972623 1.494798 8.310420

O 10.552737 0.096990 9.131715

O 10.553461 2.809854 9.164239

Mg 12.041480 7.474000 0.388370

Mg 9.506060 11.914340 2.261970

Mg 9.506060 9.012860 2.261970

Mg 11.846825 10.473771 4.502362

Mg 14.232508 7.471818 5.165268

Mg 11.823475 6.039538 7.506368

Mg 11.942184 8.933574 7.265618

Si 12.225112 10.459152 1.228063

Si 11.516610 7.474000 3.313620

Si 13.918586 10.480687 6.375919

Si 9.742936 7.415921 8.683932

O 12.927437 11.816802 0.557796

O 12.932710 9.100870 0.556660

O 10.600310 10.463600 1.386100

O 10.721890 7.474000 1.760420

O 12.964840 10.463600 2.799080

O 13.161830 7.474000 3.191290

O 10.755330 6.146160 4.002710

O 10.755330 8.801840 4.002710

O 13.155382 11.790604 5.668592

O 13.175935 9.153693 5.649670

O 10.819174 10.436774 6.407553

O 10.519506 7.596289 7.135688

O 13.092567 10.454775 7.893983

O 12.951582 7.510009 8.297821

O 10.543694 6.036575 9.200945

Mg 12.054959 13.451836 0.410431

Mg 9.503791 17.893597 2.256567

Mg 9.506060 14.992060 2.261970

Mg 11.843192 16.441175 4.521311

Mg 14.226776 13.462683 5.172736
Mg 11.869741 11.975578 7.340904
Mg 11.902970 14.888671 7.409423
Mg 9.706175 16.383736 9.278983
Si 12.223619 16.442496 1.231012
Si 11.510760 13.454095 3.310232
Si 13.888101 16.449384 6.395640
Si 9.826010 13.467616 8.351655
O 12.921519 17.800951 0.558115
O 12.927740 15.086003 0.560545
O 10.600310 16.442800 1.386100
O 10.721890 13.453200 1.760420
O 12.964840 16.442800 2.799080
O 13.161830 13.453200 3.191290
O 10.755330 12.125360 4.002710
O 10.755330 14.781030 4.002710
O 13.132415 17.770534 5.703731
O 13.138706 15.130412 5.691640
O 10.773732 16.438463 6.511627
O 10.598499 13.477891 6.822520
O 13.088244 16.434960 7.936515
O 12.950537 13.407877 8.283918
O 10.435587 11.973187 8.904984
O 10.578253 14.736180 9.108572
Mg 16.804280 1.494167 0.410611
Mg 14.253764 5.933449 2.251943
Mg 14.255060 3.033660 2.261970
Mg 16.588815 4.486699 4.522081
Mg 18.959023 1.496324 5.179231
Mg 16.621294 0.042932 7.444967
Mg 16.616191 2.948651 7.441628
Mg 14.431939 4.493061 9.283598
Si 16.973503 4.484212 1.232101
Si 16.261095 1.492866 3.310589
Si 18.618857 4.493520 6.397055
Si 14.593483 1.490954 8.476062
O 17.673758 5.841470 0.560191
O 17.674601 3.128303 0.559295
O 15.349310 4.484400 1.386100
O 15.470890 1.494800 1.760420
O 17.713830 4.484400 2.799080
O 17.910830 1.494800 3.191290
O 15.504330 0.166960 4.002710
O 15.504330 2.822640 4.002710
O 17.869145 5.814769 5.701634
O 17.867694 3.171686 5.700576
O 15.499701 4.505472 6.534800
O 15.330614 1.495965 6.908450
O 17.823111 4.493899 7.941536
O 17.723306 1.498207 8.305052
O 15.282036 0.125574 9.143087
O 15.293297 2.851298 9.144954
Mg 16.803094 7.474863 0.407718
Mg 14.254612 11.912883 2.256305

Mg 14.255060 9.012860 2.261970

Mg 16.595680 10.466659 4.529451

Mg 18.963392 7.482863 5.183368

Mg 16.618455 6.044275 7.444627

Mg 16.612543 8.952803 7.462409

Mg 14.279699 10.497882 9.412713

Si 16.973665 10.463878 1.232105

Si 16.262961 7.475395 3.307018

Si 18.632742 10.470642 6.399952

Si 14.590045 7.512269 8.464480

O 17.674332 11.819420 0.559257

O 17.674223 9.107795 0.559485

O 15.349310 10.463600 1.386100

O 15.470890 7.474000 1.760420

O 17.713840 10.463600 2.799080

O 17.910830 7.474000 3.191290

O 15.504330 6.146160 4.002710

O 15.504330 8.801840 4.002710

O 17.885981 11.793853 5.706123

O 17.883779 9.147758 5.706334

O 15.555188 10.473750 6.472816

O 15.327202 7.491736 6.904119

O 17.845655 10.472725 7.945431

O 17.720553 7.488298 8.315761

O 15.268477 6.148988 9.146475

O 15.247573 8.885734 9.124789

Mg 16.804737 13.453580 0.410556

Mg 14.253608 17.892639 2.256547

Mg 14.255060 14.992060 2.261970

Mg 16.592609 16.440526 4.526110

Mg 18.967533 13.452368 5.185701

Mg 16.615764 12.001809 7.458304

Mg 16.616884 14.898273 7.457325

Mg 14.395472 16.429053 9.316513

Si 16.973570 16.442915 1.232100

Si 16.261522 13.453137 3.310155

Si 18.625537 16.437464 6.399529

Si 14.576523 13.430525 8.463667

O 17.675218 17.798223 0.559007

O 17.674865 15.087242 0.559551

O 15.349310 16.442800 1.386100

O 15.470890 13.453200 1.760420

O 17.713840 16.442800 2.799080

O 17.910830 13.453200 3.191290

O 15.504330 12.125360 4.002710

O 15.504330 14.781030 4.002710

O 17.878525 17.761655 5.703405

O 17.876959 15.115552 5.706011

O 15.528366 16.444218 6.515851

O 15.329533 13.463196 6.901215

O 17.832971 16.440267 7.945441

O 17.730832 13.456370 8.320516

O 15.280639 12.066878 9.102342

O 15.261902 14.790042 9.155088

C 10.135767 8.705808 10.021410

C 11.457847 8.819803 10.519004

C 11.879887 9.970650 11.233495

C 10.932639 11.020650 11.487394

C 9.533889 10.779266 11.236909

C 9.159295 9.613429 10.514145

C 8.595236 11.850400 11.490363

C 9.035670 13.107074 11.864471

C 10.442809 13.398054 12.019322

C 10.933362 14.707589 12.270430

C 12.318093 14.965899 12.340457

C 13.253547 13.920684 12.197070

C 12.806602 12.591892 11.971732

C 11.393415 12.327102 11.856709

C 13.727888 11.476775 11.892914

C 13.274245 10.198035 11.574691

H 12.186943 8.045486 10.255038

H 8.103236 9.453746 10.258582

H 7.530183 11.696884 11.272236

H 8.307782 13.918030 11.992776

H 10.218177 15.523372 12.445709

H 12.665696 15.983975 12.556419

H 14.329053 14.118128 12.277371

H 14.787274 11.655847 12.114646

H 13.966678 9.340725 11.582903

H 9.710064 11.288413 8.756335

**Coronene [010]-fo**

Number of atoms: 372

Mg 2.543480 1.494800 0.388370

Mg 0.008060 5.935140 2.261970

Mg 0.008060 3.033660 2.261970

Mg 2.352889 4.482263 4.520173

Mg 4.722399 1.498797 5.181263

Mg 2.384236 0.046218 7.433501

Mg 2.395038 2.943711 7.438729

Mg 0.196991 4.480333 9.270540

Si 2.727970 4.484400 1.221250

Si 2.018610 1.494800 3.313620

Si 4.394121 4.482874 6.390608

Si 0.363629 1.496447 8.468601

O 3.434710 5.847120 0.556660

O 3.434700 3.121670 0.556660

O 1.102310 4.484400 1.386100

O 1.223890 1.494800 1.760420

O 3.466830 4.484400 2.799080

O 3.663830 1.494800 3.191290

O 1.257330 0.166960 4.002710

O 1.257330 2.822640 4.002710

O 3.630807 5.802090 5.694026

O 3.629192 3.163389 5.698538

O 1.273430 4.492929 6.535923

O 1.095392 1.499707 6.900890

O 3.607704 4.484441 7.935450

| | | | |
|---|---|---|---|
| O | 3.479409 | 1.488294 | 8.306692 |
| O | 1.061663 | 0.140374 | 9.141019 |
| O | 1.068413 | 2.847716 | 9.147180 |
| Mg | 2.543480 | 7.474000 | 0.388370 |
| Mg | 0.008060 | 11.914340 | 2.261970 |
| Mg | 0.008060 | 9.012860 | 2.261970 |
| Mg | 2.355522 | 10.466169 | 4.522239 |
| Mg | 4.709098 | 7.476032 | 5.144823 |
| Mg | 2.401868 | 6.024061 | 7.434192 |
| Mg | 2.401685 | 8.921194 | 7.433475 |
| Mg | 0.202655 | 10.462372 | 9.270605 |
| Si | 2.727970 | 10.463600 | 1.221250 |
| Si | 2.018610 | 7.474000 | 3.313620 |
| Si | 4.395688 | 10.465344 | 6.398769 |
| Si | 0.372974 | 7.468115 | 8.518282 |
| O | 3.434710 | 11.826320 | 0.556660 |
| O | 3.434710 | 9.100870 | 0.556660 |
| O | 1.102310 | 10.463600 | 1.386100 |
| O | 1.223890 | 7.474000 | 1.760420 |
| O | 3.466830 | 10.463600 | 2.799080 |
| O | 3.663830 | 7.474000 | 3.191290 |
| O | 1.257330 | 6.146160 | 4.002710 |
| O | 1.257330 | 8.801840 | 4.002710 |
| O | 3.631278 | 11.785564 | 5.704839 |
| O | 3.630183 | 9.147775 | 5.697797 |
| O | 1.271976 | 10.455547 | 6.543543 |
| O | 1.091697 | 7.473152 | 6.946389 |
| O | 3.604305 | 10.461123 | 7.941463 |
| O | 3.507164 | 7.472836 | 8.288250 |
| O | 1.086809 | 6.120146 | 9.216195 |
| O | 1.082253 | 8.814892 | 9.224533 |
| Mg | 2.543480 | 13.453200 | 0.388370 |
| Mg | 0.008060 | 17.893540 | 2.261970 |
| Mg | 0.008060 | 14.992060 | 2.261970 |
| Mg | 2.346904 | 16.442825 | 4.517595 |
| Mg | 4.723835 | 13.448392 | 5.180988 |
| Mg | 2.395118 | 12.004311 | 7.442057 |
| Mg | 2.381248 | 14.902380 | 7.431560 |
| Mg | 0.190520 | 16.444005 | 9.277115 |
| Si | 2.727970 | 16.442800 | 1.221250 |
| Si | 2.018610 | 13.453200 | 3.313620 |
| Si | 4.384752 | 16.441585 | 6.389517 |
| Si | 0.364439 | 13.451397 | 8.475618 |
| O | 3.434710 | 17.805520 | 0.556660 |
| O | 3.434710 | 15.080070 | 0.556660 |
| O | 1.102310 | 16.442800 | 1.386100 |
| O | 1.223890 | 13.453200 | 1.760420 |
| O | 3.466830 | 16.442800 | 2.799080 |
| O | 3.663830 | 13.453200 | 3.191290 |
| O | 1.257330 | 12.125360 | 4.002710 |
| O | 1.257330 | 14.781030 | 4.002710 |
| O | 3.634119 | 17.763708 | 5.694921 |
| O | 3.633298 | 15.120363 | 5.694770 |
| O | 1.271587 | 16.441681 | 6.516251 |

O 1.092348 13.446517 6.905626

O 3.589994 16.441731 7.933168

O 3.478881 13.463322 8.301665

O 1.070348 12.099080 9.154950

O 1.062214 14.810034 9.144090

Mg 7.292480 1.494800 0.388370

Mg 4.757060 5.935140 2.261970

Mg 4.757060 3.033660 2.261970

Mg 7.101395 4.480407 4.525672

Mg 9.472231 1.496586 5.191416

Mg 7.132533 0.049920 7.448567

Mg 7.129903 2.951488 7.446285

Mg 4.913371 4.526650 9.334987

Si 7.476970 4.484400 1.221250

Si 6.767610 1.494800 3.313620

Si 9.149768 4.471627 6.403789

Si 5.099845 1.500095 8.474815

O 8.183700 5.847120 0.556660

O 8.183700 3.121670 0.556660

O 5.851310 4.484400 1.386100

O 5.972890 1.494800 1.760420

O 8.215830 4.484400 2.799080

O 8.412830 1.494800 3.191290

O 6.006330 0.166960 4.002710

O 6.006330 2.822640 4.002710

O 8.399298 5.786164 5.683818

O 8.388502 3.151312 5.709128

O 6.035669 4.470753 6.476787

O 5.839037 1.495482 6.910409

O 8.350530 4.485114 7.936254

O 8.222911 1.494969 8.328401

O 5.800645 0.145716 9.157208

O 5.782331 2.862343 9.152308

Mg 7.292480 7.474000 0.388370

Mg 4.757060 11.914340 2.261970

Mg 4.757060 9.012860 2.261970

Mg 7.101510 10.467233 4.528678

Mg 9.467840 7.472166 5.166232

Mg 7.144942 6.021284 7.380234

Mg 7.147874 8.932575 7.392366

Mg 4.898613 10.420425 9.354703

Si 7.476970 10.463600 1.221250

Si 6.767610 7.474000 3.313620

Si 9.152527 10.472679 6.407142

Si 5.139658 7.476514 8.364240

O 8.183700 11.826320 0.556660

O 8.183700 9.100870 0.556660

O 5.851310 10.463600 1.386100

O 5.972890 7.474000 1.760420

O 8.215830 10.463600 2.799080

O 8.412830 7.474000 3.191290

O 6.006330 6.146160 4.002710

O 6.006330 8.801840 4.002710

O 8.390570 11.793372 5.713055

O 8.401200 9.155869 5.691807

O 6.037957 10.475849 6.479711

O 5.860231 7.480152 6.804903

O 8.364241 10.463900 7.946289

O 8.239600 7.473458 8.257284

O 5.849584 6.117828 9.007192

O 5.843295 8.833546 9.018852

Mg 7.292480 13.453200 0.388370

Mg 4.757060 17.893540 2.261970

Mg 4.757060 14.992060 2.261970

Mg 7.095943 16.441704 4.522010

Mg 9.472194 13.447620 5.188415

Mg 7.132630 11.992199 7.453515

Mg 7.136941 14.892826 7.451733

Mg 4.940325 16.439372 9.265031

Si 7.476970 16.442800 1.221250

Si 6.767610 13.453200 3.313620

Si 9.129104 16.439175 6.400193

Si 5.099546 13.445814 8.463394

O 8.183700 17.805520 0.556660

O 8.183700 15.080070 0.556660

O 5.851310 16.442800 1.386100

O 5.972890 13.453200 1.760420

O 8.215830 16.442800 2.799080

O 8.412830 13.453200 3.191290

O 6.006330 12.125360 4.002710

O 6.006330 14.781030 4.002710

O 8.383005 17.761759 5.704403

O 8.383219 15.115306 5.706506

O 6.025860 16.440609 6.528009

O 5.845588 13.453698 6.903297

O 8.339090 16.440811 7.945904

O 8.226814 13.449116 8.340801

O 5.774003 12.075478 9.127285

O 5.801104 14.796233 9.154147

Mg 12.041480 1.494800 0.388370

Mg 9.506060 5.935140 2.261970

Mg 9.506060 3.033660 2.261970

Mg 11.837627 4.484459 4.526174

Mg 14.212693 1.493344 5.180880

Mg 11.874266 0.057826 7.458546

Mg 11.863517 2.962329 7.464280

Mg 9.583086 4.515040 9.401802

Si 12.225970 4.484400 1.221250

Si 11.516610 1.494800 3.313620

Si 13.884697 4.477944 6.392558

Si 9.843565 1.516412 8.500087

O 12.932710 5.847120 0.556660

O 12.932710 3.121670 0.556660

O 10.600310 4.484400 1.386100

O 10.721890 1.494800 1.760420

O 12.964830 4.484400 2.799080

O 13.161830 1.494800 3.191290

O 10.755330 0.166960 4.002710

O 10.755330 2.822640 4.002710

O 13.142311 5.801381 5.697630

O 13.135554 3.156123 5.703755

O 10.793340 4.473487 6.487089

O 10.584303 1.505406 6.932065

O 13.097448 4.484910 7.939127

O 12.978323 1.507827 8.328611

O 10.549835 0.158138 9.172758

O 10.513226 2.887453 9.171812

Mg 12.041480 7.474000 0.388370

Mg 9.506060 11.914340 2.261970

Mg 9.506060 9.012860 2.261970

Mg 11.838203 10.462846 4.526884

Mg 14.217975 7.472601 5.182771

Mg 11.874435 6.013265 7.435652

Mg 11.877235 8.925942 7.431825

Mg 9.620449 10.433881 9.400563

Si 12.225970 10.463600 1.221250

Si 11.516610 7.474000 3.313620

Si 13.885176 10.467845 6.395285

Si 9.858060 7.469467 8.407339

O 12.932710 11.826320 0.556660

O 12.932710 9.100870 0.556660

O 10.600310 10.463600 1.386100

O 10.721890 7.474000 1.760420

O 12.964840 10.463600 2.799080

O 13.161830 7.474000 3.191290

O 10.755330 6.146160 4.002710

O 10.755330 8.801840 4.002710

O 13.137636 11.791109 5.705444

O 13.143895 9.143320 5.700096

O 10.796607 10.471683 6.487189

O 10.612855 7.468115 6.853728

O 13.096290 10.461368 7.940867

O 12.983176 7.469777 8.317092

O 10.542245 6.104588 9.077045

O 10.553489 8.830793 9.073422

Mg 12.041480 13.453200 0.388370

Mg 9.506060 17.893540 2.261970

Mg 9.506060 14.992060 2.261970

Mg 11.842049 16.442594 4.523297

Mg 14.215049 13.454266 5.183452

Mg 11.867105 11.989889 7.460712

Mg 11.875044 14.894501 7.456492

Mg 9.697067 16.446180 9.272461

Si 12.225970 16.442800 1.221250

Si 11.516610 13.453200 3.313620

Si 13.874039 16.443160 6.404055

Si 9.849403 13.436280 8.497338

O 12.932710 17.805520 0.556660

O 12.932710 15.080070 0.556660

O 10.600310 16.442800 1.386100

O 10.721890 13.453200 1.760420

O 12.964840 16.442800 2.799080

O 13.161830 13.453200 3.191290

O 10.755330 12.125360 4.002710

O 10.755330 14.781030 4.002710

O 13.124740 17.765208 5.709875

O 13.126288 15.119537 5.710059

O 10.769410 16.443629 6.540015

O 10.584562 13.448615 6.927047

O 13.082532 16.444202 7.950334

O 12.979560 13.445807 8.325324

O 10.532628 12.070261 9.163752

O 10.551224 14.797975 9.169634

Mg 16.790480 1.494800 0.388370

Mg 14.255060 5.935140 2.261970

Mg 14.255060 3.033660 2.261970

Mg 16.589460 4.485606 4.521371

Mg 18.966912 1.494527 5.178265

Mg 16.627990 0.047683 7.444371

Mg 16.630437 2.944098 7.442102

Mg 14.441208 4.484889 9.272867

Si 16.974970 4.484400 1.221250

Si 16.265610 1.494800 3.313620

Si 18.630444 4.488087 6.393800

Si 14.601032 1.499908 8.476371

O 17.681710 5.847120 0.556660

O 17.681710 3.121670 0.556660

O 15.349310 4.484400 1.386100

O 15.470890 1.494800 1.760420

O 17.713830 4.484400 2.799080

O 17.910830 1.494800 3.191290

O 15.504330 0.166960 4.002710

O 15.504330 2.822640 4.002710

O 17.881438 5.813057 5.705598

O 17.881704 3.167892 5.698498

O 15.528627 4.484368 6.525760

O 15.335494 1.495715 6.908549

O 17.843171 4.489344 7.941828

O 17.738457 1.498082 8.306313

O 15.303745 0.143735 9.147901

O 15.313489 2.850697 9.148573

Mg 16.790480 7.474000 0.388370

Mg 14.255060 11.914340 2.261970

Mg 14.255060 9.012860 2.261970

Mg 16.590011 10.462944 4.522033

Mg 18.970790 7.474727 5.187746

Mg 16.634274 6.028055 7.451763

Mg 16.635274 8.918965 7.454488

Mg 14.443005 10.464065 9.271461

Si 16.974970 10.463600 1.221250

Si 16.265610 7.474000 3.313620

Si 18.629416 10.462182 6.397916

Si 14.602103 7.472727 8.479349

O 17.681710 11.826320 0.556660

O 17.681710 9.100870 0.556660

O 15.349310 10.463600 1.386100

O 15.470890 7.474000 1.760420

O 17.713840 10.463600 2.799080

O 17.910830 7.474000 3.191290

O 15.504330 6.146160 4.002710

O 15.504330 8.801840 4.002710

O 17.881921 11.783971 5.704177

O 17.881926 9.137392 5.708204

O 15.529262 10.464580 6.529279

O 15.347313 7.474233 6.915309

O 17.843260 10.459441 7.946927

O 17.749040 7.471041 8.344863

O 15.310529 6.121454 9.157489

O 15.308174 8.824522 9.159378

Mg 16.790480 13.453200 0.388370

Mg 14.255060 17.893540 2.261970

Mg 14.255060 14.992060 2.261970

Mg 16.589337 16.442117 4.521150

Mg 18.967124 13.452165 5.179641

Mg 16.631523 12.005470 7.448040

Mg 16.629346 14.899817 7.448420

Mg 14.434444 16.445289 9.282602

Si 16.974970 16.442800 1.221250

Si 16.265610 13.453200 3.313620

Si 18.626573 16.440221 6.393841

Si 14.601348 13.452230 8.477845

O 17.681710 17.805520 0.556660

O 17.681710 15.080070 0.556660

O 15.349310 16.442800 1.386100

O 15.470890 13.453200 1.760420

O 17.713840 16.442800 2.799080

O 17.910830 13.453200 3.191290

O 15.504330 12.125360 4.002710

O 15.504330 14.781030 4.002710

O 17.877027 17.763146 5.700467

O 17.875967 15.115899 5.704581

O 15.517366 16.442396 6.527937

O 15.338274 13.452993 6.911332

O 17.838473 16.442443 7.941440

O 17.738755 13.450085 8.316022

O 15.310993 12.100510 9.152465

O 15.303695 14.809436 9.148612

C 3.578670 8.554762 11.487335

C 4.144804 9.821615 11.675006

C 5.567655 9.984555 11.866617

C 6.400053 8.809399 11.869840

C 5.803341 7.504022 11.802245

C 4.386509 7.372684 11.604490

C 3.805899 6.058385 11.547732

C 4.601793 4.917271 11.711366

C 6.042318 5.021311 11.815396

C 6.636310 6.331034 11.850900

C 8.063130 6.469520 11.919846

C 8.902276 5.297833 11.955614

C 8.284354 4.001247 11.859522

C 6.895573 3.866672 11.791778

C 10.327223 5.462025 12.074042

C 10.901378 6.728628 12.085057

C 10.089629 7.907403 12.016421

C 8.661786 7.774495 11.952714

C 7.827873 8.945064 11.919243

C 8.424301 10.256892 11.923085

C 7.571925 11.412551 11.920513

C 6.186558 11.280276 11.918712

C 9.864411 10.366365 11.956755

C 10.663452 9.222529 11.999141

H 2.514371 8.455481 11.230743

H 3.498988 10.712514 11.692426

H 2.724305 5.977264 11.369966

H 4.136216 3.922558 11.776616

H 8.919212 3.104609 11.867193

H 6.450023 2.873601 11.666853

H 10.956512 4.565653 12.144821

H 11.989925 6.841255 12.134958

H 8.023403 12.410811 11.912666

H 5.557235 12.176139 11.907499

H 10.323440 11.364149 11.947793

H 11.754790 9.316475 12.029757

**Coronene Fe-[010]-fo**

Number of atoms: 372

Mg 2.543480 1.494800 0.388370

Mg 0.008060 5.935140 2.261970

Mg 0.008060 3.033660 2.261970

Mg 2.355657 4.482056 4.525323

Mg 4.722180 1.502452 5.182184

Mg 2.380462 0.046806 7.430410

Mg 2.394916 2.946010 7.444225

Mg 0.203735 4.487961 9.277032

Si 2.727970 4.484400 1.221250

Si 2.018610 1.494800 3.313620

Si 4.396519 4.481205 6.402510

Si 0.362259 1.498931 8.469318

O 3.434710 5.847120 0.556660

O 3.434700 3.121670 0.556660

O 1.102310 4.484400 1.386100

O 1.223890 1.494800 1.760420

O 3.466830 4.484400 2.799080

O 3.663830 1.494800 3.191290

O 1.257330 0.166960 4.002710

O 1.257330 2.822640 4.002710

O 3.629845 5.795736 5.697254

O 3.629448 3.162635 5.706501

O 1.272619 4.493839 6.539837

O 1.093133 1.502293 6.901211

O 3.608972 4.485947 7.946439

O 3.478255 1.483772 8.300934

O 1.059291 0.140824 9.139159

O 1.066593 2.849728 9.148901

Mg 2.543480 7.474000 0.388370

Mg 0.008060 11.914340 2.261970

Mg 0.008060 9.012860 2.261970

Mg 2.353259 10.465041 4.524964

Mg 4.701347 7.468784 5.141239

Mg 2.400788 6.024752 7.438413

Mg 2.398827 8.923478 7.444470

Mg 0.202103 10.463662 9.280095

Si 2.727970 10.463600 1.221250

Si 2.018610 7.474000 3.313620

Si 4.393043 10.464050 6.400030

Si 0.376665 7.474759 8.519278

O 3.434710 11.826320 0.556660

O 3.434710 9.100870 0.556660

O 1.102310 10.463600 1.386100

O 1.223890 7.474000 1.760420

O 3.466830 10.463600 2.799080

O 3.663830 7.474000 3.191290

O 1.257330 6.146160 4.002710

O 1.257330 8.801840 4.002710

O 3.631142 11.785130 5.705231

O 3.630275 9.147048 5.699773

O 1.274461 10.457603 6.539320

O 1.091579 7.473336 6.944002

O 3.610460 10.465285 7.946216

O 3.505763 7.474458 8.283639

O 1.089475 6.127188 9.213881

O 1.087494 8.826113 9.206986

Mg 2.543480 13.453200 0.388370

Mg 0.008060 17.893540 2.261970

Mg 0.008060 14.992060 2.261970

Mg 2.345370 16.443789 4.517306

Mg 4.715867 13.449906 5.177234

Mg 2.393047 12.003846 7.444912

Mg 2.379667 14.903910 7.430909

Mg 0.187045 16.445703 9.277742

Si 2.727970 16.442800 1.221250

Si 2.018610 13.453200 3.313620

Si 4.383066 16.443847 6.387015

Si 0.361690 13.453529 8.471344

O 3.434710 17.805520 0.556660

O 3.434710 15.080070 0.556660

O 1.102310 16.442800 1.386100

O 1.223890 13.453200 1.760420

O 3.466830 16.442800 2.799080

O 3.663830 13.453200 3.191290

O 1.257330 12.125360 4.002710

O 1.257330 14.781030 4.002710

O 3.632898 17.764714 5.691492

O 3.631342 15.122056 5.692931

O 1.270239 16.442383 6.513979

O 1.092072 13.448525 6.902375

O 3.589423 16.442698 7.930363

O 3.475594 13.464472 8.305243

O 1.065329 12.101406 9.148463
O 1.059041 14.812467 9.139433
Mg 7.292480 1.494800 0.388370
Mg 4.757060 5.935140 2.261970
Mg 4.757060 3.033660 2.261970
Mg 7.100226 4.478855 4.530134
Mg 9.470824 1.496990 5.187417
Mg 7.134179 0.051406 7.449595
Mg 7.132421 2.953511 7.456705
Mg 4.900398 4.530253 9.371337
Si 7.476970 4.484400 1.221250
Si 6.767610 1.494800 3.313620
Si 9.153539 4.468198 6.408769
Si 5.098781 1.502710 8.463692
O 8.183700 5.847120 0.556660
O 8.183700 3.121670 0.556660
O 5.851310 4.484400 1.386100
O 5.972890 1.494800 1.760420
O 8.215830 4.484400 2.799080
O 8.412830 1.494800 3.191290
O 6.006330 0.166960 4.002710
O 6.006330 2.822640 4.002710
O 8.401735 5.781763 5.686512
O 8.391486 3.148501 5.713915
O 6.039495 4.464491 6.471619
O 5.845189 1.494111 6.903148
O 8.366032 4.482135 7.945311
O 8.230599 1.491587 8.332329
O 5.801500 0.149164 9.148959
O 5.773921 2.870682 9.129131
Mg 7.292480 7.474000 0.388370
Mg 4.757060 11.914340 2.261970
Mg 4.757060 9.012860 2.261970
Mg 7.097010 10.465710 4.524648
Mg 9.465855 7.464977 5.161670
Mg 7.141432 6.006791 7.381117
Mg 7.135347 8.918881 7.366743
Mg 4.919264 10.413473 9.351941
Si 7.476970 10.463600 1.221250
Si 6.767610 7.474000 3.313620
Si 9.164214 10.470458 6.401050
Si 5.136643 7.467151 8.349486
O 8.183700 11.826320 0.556660
O 8.183700 9.100870 0.556660
O 5.851310 10.463600 1.386100
O 5.972890 7.474000 1.760420
O 8.215830 10.463600 2.799080
O 8.412830 7.474000 3.191290
O 6.006330 6.146160 4.002710
O 6.006330 8.801840 4.002710
O 8.392405 11.791147 5.719357
O 8.403736 9.157292 5.694111
O 6.039160 10.475768 6.471628
O 5.856540 7.461310 6.786922

O 8.360590 10.464840 7.947067

O 8.227621 7.474173 8.240587

O 5.843645 6.109642 8.994209

O 5.850600 8.829352 8.978068

Mg 7.292480 13.453200 0.388370

Mg 4.757060 17.893540 2.261970

Mg 4.757060 14.992060 2.261970

Mg 7.092931 16.444234 4.521239

Mg 9.466706 13.448711 5.177183

Mg 7.114415 11.993657 7.433146

Mg 7.123164 14.898333 7.443730

Mg 4.937532 16.445263 9.265146

Si 7.476970 16.442800 1.221250

Si 6.767610 13.453200 3.313620

Si 9.127976 16.437476 6.398779

Si 5.095186 13.448182 8.465149

O 8.183700 17.805520 0.556660

O 8.183700 15.080070 0.556660

O 5.851310 16.442800 1.386100

O 5.972890 13.453200 1.760420

O 8.215830 16.442800 2.799080

O 8.412830 13.453200 3.191290

O 6.006330 12.125360 4.002710

O 6.006330 14.781030 4.002710

O 8.384044 17.759089 5.703148

O 8.382026 15.112071 5.707028

O 6.025262 16.442629 6.522148

O 5.835089 13.454893 6.899342

O 8.338519 16.435480 7.945237

O 8.216580 13.440876 8.322435

O 5.780567 12.082103 9.130418

O 5.801487 14.802433 9.144540

Mg 12.041480 1.494800 0.388370

Mg 9.506060 5.935140 2.261970

Mg 9.506060 3.033660 2.261970

Mg 11.837481 4.484643 4.526336

Mg 14.213910 1.496195 5.180621

Mg 11.877068 0.054810 7.452494

Mg 11.865677 2.961919 7.456657

Mg 9.612595 4.514238 9.414347

Si 12.225970 4.484400 1.221250

Si 11.516610 1.494800 3.313620

Si 13.885090 4.484005 6.392788

Si 9.852758 1.513195 8.490013

O 12.932710 5.847120 0.556660

O 12.932710 3.121670 0.556660

O 10.600310 4.484400 1.386100

O 10.721890 1.494800 1.760420

O 12.964830 4.484400 2.799080

O 13.161830 1.494800 3.191290

O 10.755330 0.166960 4.002710

O 10.755330 2.822640 4.002710

O 13.142499 5.807721 5.697118

O 13.138488 3.161307 5.700951

O 10.798155 4.477310 6.480382

O 10.588167 1.497712 6.919596

O 13.093900 4.491348 7.936248

O 12.979799 1.504309 8.319784

O 10.557166 0.151478 9.163177

O 10.534186 2.880783 9.151711

Mg 12.041480 7.474000 0.388370

Mg 9.506060 11.914340 2.261970

Mg 9.506060 9.012860 2.261970

Mg 11.842680 10.460691 4.521153

Mg 14.221379 7.473538 5.183952

Mg 11.872735 6.024490 7.428145

Mg 11.870356 8.941990 7.427768

Fe 9.504895 10.477918 9.553951

Si 12.225970 10.463600 1.221250

Si 11.516610 7.474000 3.313620

Si 13.890938 10.465806 6.393892

Si 9.842044 7.477509 8.392900

O 12.932710 11.826320 0.556660

O 12.932710 9.100870 0.556660

O 10.600310 10.463600 1.386100

O 10.721890 7.474000 1.760420

O 12.964840 10.463600 2.799080

O 13.161830 7.474000 3.191290

O 10.755330 6.146160 4.002710

O 10.755330 8.801840 4.002710

O 13.142449 11.790023 5.702681

O 13.145551 9.145230 5.697822

O 10.804398 10.478543 6.469557

O 10.611202 7.487515 6.847878

O 13.096240 10.464648 7.938424

O 12.973120 7.486401 8.319898

O 10.540151 6.122488 9.057681

O 10.505169 8.870845 9.053562

Mg 12.041480 13.453200 0.388370

Mg 9.506060 17.893540 2.261970

Mg 9.506060 14.992060 2.261970

Mg 11.842339 16.444218 4.521213

Mg 14.219094 13.453798 5.180433

Mg 11.876027 11.983967 7.436303

Mg 11.880512 14.891054 7.449399

Mg 9.697847 16.444831 9.269975

Si 12.225970 16.442800 1.221250

Si 11.516610 13.453200 3.313620

Si 13.875254 16.441589 6.401898

Si 9.833278 13.446565 8.473096

O 12.932710 17.805520 0.556660

O 12.932710 15.080070 0.556660

O 10.600310 16.442800 1.386100

O 10.721890 13.453200 1.760420

O 12.964840 16.442800 2.799080

O 13.161830 13.453200 3.191290

O 10.755330 12.125360 4.002710

O 10.755330 14.781030 4.002710

O 13.125639 17.762449 5.706793
O 13.126177 15.116386 5.709864
O 10.768480 16.434359 6.540148
O 10.584970 13.438179 6.922229
O 13.082379 16.440536 7.948472
O 12.969849 13.436008 8.326637
O 10.513560 12.056195 9.151755
O 10.542688 14.785172 9.165722
Mg 16.790480 1.494800 0.388370
Mg 14.255060 5.935140 2.261970
Mg 14.255060 3.033660 2.261970
Mg 16.591489 4.486665 4.523959
Mg 18.965967 1.498900 5.179720
Mg 16.629751 0.049873 7.442573
Mg 16.630946 2.947883 7.445399
Mg 14.431285 4.483945 9.276398
Si 16.974970 4.484400 1.221250
Si 16.265610 1.494800 3.313620
Si 18.629841 4.492567 6.400579
Si 14.601612 1.500081 8.473172
O 17.681710 5.847120 0.556660
O 17.681710 3.121670 0.556660
O 15.349310 4.484400 1.386100
O 15.470890 1.494800 1.760420
O 17.713830 4.484400 2.799080
O 17.910830 1.494800 3.191290
O 15.504330 0.166960 4.002710
O 15.504330 2.822640 4.002710
O 17.879478 5.816484 5.711232
O 17.880980 3.173082 5.702131
O 15.528635 4.488559 6.528146
O 15.337635 1.497739 6.906329
O 17.844086 4.491752 7.949551
O 17.736900 1.499352 8.307115
O 15.304623 0.143860 9.144442
O 15.308469 2.852091 9.147833
Mg 16.790480 7.474000 0.388370
Mg 14.255060 11.914340 2.261970
Mg 14.255060 9.012860 2.261970
Mg 16.593570 10.461876 4.524440
Mg 18.972399 7.475049 5.193321
Mg 16.632826 6.030640 7.461406
Mg 16.637127 8.917672 7.462833
Mg 14.434815 10.461119 9.279705
Si 16.974970 10.463600 1.221250
Si 16.265610 7.474000 3.313620
Si 18.631002 10.461825 6.402556
Si 14.593898 7.476207 8.483558
O 17.681710 11.826320 0.556660
O 17.681710 9.100870 0.556660
O 15.349310 10.463600 1.386100
O 15.470890 7.474000 1.760420
O 17.713840 10.463600 2.799080
O 17.910830 7.474000 3.191290

| | | | |
|---|---|---|---|
| O | 15.504330 | 6.146160 | 4.002710 |
| O | 15.504330 | 8.801840 | 4.002710 |
| O | 17.881544 | 11.782572 | 5.706403 |
| O | 17.881708 | 9.137520 | 5.713739 |
| O | 15.532792 | 10.463299 | 6.530203 |
| O | 15.343249 | 7.475941 | 6.921480 |
| O | 17.844452 | 10.461309 | 7.951406 |
| O | 17.750118 | 7.473182 | 8.351643 |
| O | 15.297037 | 6.123062 | 9.162713 |
| O | 15.304874 | 8.825223 | 9.164224 |
| Mg | 16.790480 | 13.453200 | 0.388370 |
| Mg | 14.255060 | 17.893540 | 2.261970 |
| Mg | 14.255060 | 14.992060 | 2.261970 |
| Mg | 16.590836 | 16.443242 | 4.520418 |
| Mg | 18.966571 | 13.451164 | 5.179643 |
| Mg | 16.631568 | 12.004299 | 7.450698 |
| Mg | 16.625947 | 14.899371 | 7.447161 |
| Mg | 14.433785 | 16.447586 | 9.282327 |
| Si | 16.974970 | 16.442800 | 1.221250 |
| Si | 16.265610 | 13.453200 | 3.313620 |
| Si | 18.625264 | 16.440361 | 6.393181 |
| Si | 14.595125 | 13.448005 | 8.479259 |
| O | 17.681710 | 17.805520 | 0.556660 |
| O | 17.681710 | 15.080070 | 0.556660 |
| O | 15.349310 | 16.442800 | 1.386100 |
| O | 15.470890 | 13.453200 | 1.760420 |
| O | 17.713840 | 16.442800 | 2.799080 |

| | | | |
|---|---|---|---|
| O | 17.910830 | 13.453200 | 3.191290 |
| O | 15.504330 | 12.125360 | 4.002710 |
| O | 15.504330 | 14.781030 | 4.002710 |
| O | 17.876444 | 17.763738 | 5.699510 |
| O | 17.874189 | 15.116632 | 5.702501 |
| O | 15.517440 | 16.440961 | 6.526609 |
| O | 15.332798 | 13.450469 | 6.913726 |
| O | 17.835748 | 16.442024 | 7.940197 |
| O | 17.735535 | 13.452992 | 8.312521 |
| O | 15.304204 | 12.097477 | 9.154556 |
| O | 15.293168 | 14.806697 | 9.149648 |
| C | 4.099439 | 8.871861 | 11.637091 |
| C | 4.894586 | 10.017687 | 11.728741 |
| C | 6.346073 | 9.927473 | 11.721258 |
| C | 6.949767 | 8.611956 | 11.758440 |
| C | 6.123274 | 7.437804 | 11.760186 |
| C | 4.690890 | 7.556481 | 11.649016 |
| C | 3.888678 | 6.372988 | 11.596728 |
| C | 4.475440 | 5.102194 | 11.729539 |
| C | 5.908966 | 4.951539 | 11.832853 |
| C | 6.731688 | 6.134511 | 11.812774 |
| C | 8.162535 | 6.010954 | 11.853062 |
| C | 8.773128 | 4.701377 | 11.869191 |
| C | 7.927221 | 3.538945 | 11.852457 |
| C | 6.540870 | 3.660758 | 11.855629 |
| C | 10.211880 | 4.603477 | 11.945297 |
| C | 10.999980 | 5.751932 | 12.001204 |

C 10.418846 7.064065 11.974529

C 8.989056 7.185431 11.881524

C 8.377884 8.483967 11.809747

C 9.217448 9.659203 11.803114

C 8.590437 10.953190 11.585308

C 7.176210 11.075177 11.617909

C 10.636410 9.510860 11.976892

C 11.220962 8.248452 12.039565

H 3.005672 8.946948 11.559903

H 4.430539 11.009895 11.833893

H 2.805304 6.467964 11.436261

H 3.838509 4.209602 11.819812

H 8.389390 2.544937 11.846920

H 5.920236 2.759420 11.829392

H 10.672675 3.607205 11.943794

H 12.090853 5.666229 12.071022

H 9.205536 11.859180 11.681436

H 6.719586 12.064045 11.500654

H 11.253683 10.416371 12.039137

H 12.307906 8.145832 12.132766

**Coronene Ni-[010]-fo**

Number of atoms: 372

Mg 2.543480 1.494800 0.388370

Mg 0.008060 5.935140 2.261970

Mg 0.008060 3.033660 2.261970

Mg 2.342244 4.483517 4.516869

Mg 4.722779 1.491183 5.181500

Mg 2.375534 0.043956 7.442276

Mg 2.377605 2.938880 7.432303

Mg 0.188834 4.482940 9.271617

Si 2.727970 4.484400 1.221250

Si 2.018610 1.494800 3.313620

Si 4.383045 4.478519 6.389060

Si 0.351829 1.497128 8.473290

O 3.434710 5.847120 0.556660

O 3.434700 3.121670 0.556660

O 1.102310 4.484400 1.386100

O 1.223890 1.494800 1.760420

O 3.466830 4.484400 2.799080

O 3.663830 1.494800 3.191290

O 1.257330 0.166960 4.002710

O 1.257330 2.822640 4.002710

O 3.634756 5.800275 5.693463

O 3.633773 3.157556 5.694770

O 1.272007 4.478525 6.512941

O 1.085725 1.491391 6.904869

O 3.582869 4.481166 7.929457

O 3.483971 1.493800 8.305833

O 1.052017 0.140398 9.145608

O 1.062636 2.849680 9.142521

Mg 2.543480 7.474000 0.388370

Mg 0.008060 11.914340 2.261970

Mg 0.008060 9.012860 2.261970

Mg 2.340250 10.463311 4.517340
Mg 4.718928 7.469126 5.177256
Mg 2.374857 6.019778 7.428903
Mg 2.376074 8.919937 7.432384
Mg 0.191715 10.458756 9.272299
Si 2.727970 10.463600 1.221250
Si 2.018610 7.474000 3.313620
Si 4.379371 10.462250 6.389529
Si 0.355476 7.470497 8.463872
O 3.434710 11.826320 0.556660
O 3.434710 9.100870 0.556660
O 1.102310 10.463600 1.386100
O 1.223890 7.474000 1.760420
O 3.466830 10.463600 2.799080
O 3.663830 7.474000 3.191290
O 1.257330 6.146160 4.002710
O 1.257330 8.801840 4.002710
O 3.633122 11.784912 5.696857
O 3.634913 9.139385 5.695058
O 1.275594 10.464915 6.514963
O 1.091696 7.470045 6.896314
O 3.585413 10.461445 7.933337
O 3.474221 7.467522 8.304189
O 1.060158 6.117104 9.139321
O 1.061645 8.823119 9.139861
Mg 2.543480 13.453200 0.388370
Mg 0.008060 17.893540 2.261970
Mg 0.008060 14.992060 2.261970
Mg 2.341856 16.442255 4.520592
Mg 4.718183 13.452496 5.179896
Mg 2.377300 12.003754 7.438691
Mg 2.377880 14.898561 7.446241
Mg 0.183141 16.441745 9.282165
Si 2.727970 16.442800 1.221250
Si 2.018610 13.453200 3.313620
Si 4.379141 16.440821 6.396463
Si 0.353323 13.448187 8.477414
O 3.434710 17.805520 0.556660
O 3.434710 15.080070 0.556660
O 1.102310 16.442800 1.386100
O 1.223890 13.453200 1.760420
O 3.466830 16.442800 2.799080
O 3.663830 13.453200 3.191290
O 1.257330 12.125360 4.002710
O 1.257330 14.781030 4.002710
O 3.628888 17.762286 5.702303
O 3.629490 15.117323 5.704215
O 1.264780 16.439049 6.527094
O 1.087981 13.452699 6.908580
O 3.586335 16.440651 7.941551
O 3.483346 13.446938 8.317837
O 1.062420 12.094445 9.146708
O 1.054094 14.806308 9.147920
Mg 7.292480 1.494800 0.388370

Mg 4.757060 5.935140 2.261970
Mg 4.757060 3.033660 2.261970
Mg 7.104933 4.481668 4.529891
Mg 9.470477 1.500734 5.188221
Mg 7.137155 0.044587 7.448463
Mg 7.146344 2.940642 7.460372
Mg 4.922593 4.471021 9.265194
Si 7.476970 4.484400 1.221250
Si 6.767610 1.494800 3.313620
Si 9.138484 4.484531 6.420742
Si 5.105129 1.492166 8.472985
O 8.183700 5.847120 0.556660
O 8.183700 3.121670 0.556660
O 5.851310 4.484400 1.386100
O 5.972890 1.494800 1.760420
O 8.215830 4.484400 2.799080
O 8.412830 1.494800 3.191290
O 6.006330 0.166960 4.002710
O 6.006330 2.822640 4.002710
O 8.376070 5.799327 5.714480
O 8.379450 3.163905 5.721817
O 6.022870 4.480631 6.537711
O 5.845538 1.495351 6.910026
O 8.359346 4.486027 7.971267
O 8.236386 1.484360 8.324791
O 5.803948 0.138213 9.149938
O 5.805875 2.843789 9.156662
Mg 7.292480 7.474000 0.388370
Mg 4.757060 11.914340 2.261970
Mg 4.757060 9.012860 2.261970
Mg 7.110313 10.464203 4.527406
Mg 9.455032 7.473940 5.156183
Mg 7.134026 6.016100 7.449702
Mg 7.134560 8.917606 7.436122
Mg 4.941984 10.469211 9.252042
Si 7.476970 10.463600 1.221250
Si 6.767610 7.474000 3.313620
Si 9.159516 10.460926 6.412257
Si 5.092257 7.464295 8.474718
O 8.183700 11.826320 0.556660
O 8.183700 9.100870 0.556660
O 5.851310 10.463600 1.386100
O 5.972890 7.474000 1.760420
O 8.215830 10.463600 2.799080
O 8.412830 7.474000 3.191290
O 6.006330 6.146160 4.002710
O 6.006330 8.801840 4.002710
O 8.384212 11.781195 5.719009
O 8.379467 9.148529 5.712187
O 6.020528 10.460914 6.546137
O 5.833635 7.466533 6.916014
O 8.359466 10.456786 7.952577
O 8.219484 7.474550 8.301607
O 5.791434 6.114378 9.174083

O 5.790916 8.812677 9.176816

Mg 7.292480 13.453200 0.388370

Mg 4.757060 17.893540 2.261970

Mg 4.757060 14.992060 2.261970

Mg 7.095347 16.443882 4.519833

Mg 9.465064 13.447670 5.167592

Mg 7.139864 12.002128 7.440907

Mg 7.122442 14.901342 7.435869

Mg 4.928592 16.444532 9.282792

Si 7.476970 16.442800 1.221250

Si 6.767610 13.453200 3.313620

Si 9.129995 16.437633 6.392245

Si 5.104292 13.452901 8.483076

O 8.183700 17.805520 0.556660

O 8.183700 15.080070 0.556660

O 5.851310 16.442800 1.386100

O 5.972890 13.453200 1.760420

O 8.215830 16.442800 2.799080

O 8.412830 13.453200 3.191290

O 6.006330 12.125360 4.002710

O 6.006330 14.781030 4.002710

O 8.382045 17.762140 5.700709

O 8.376543 15.117358 5.696611

O 6.021312 16.441770 6.522235

O 5.832905 13.441894 6.913892

O 8.340685 16.433311 7.938863

O 8.217977 13.456359 8.303869

O 5.815641 12.109546 9.182030

O 5.803538 14.812586 9.147948

Mg 12.041480 1.494800 0.388370

Mg 9.506060 5.935140 2.261970

Mg 9.506060 3.033660 2.261970

Mg 11.841525 4.484092 4.525404

Mg 14.216579 1.501930 5.188575

Mg 11.880576 0.051750 7.447416

Mg 11.868609 2.957881 7.446943

Mg 9.667719 4.517162 9.389922

Si 12.225970 4.484400 1.221250

Si 11.516610 1.494800 3.313620

Si 13.883766 4.486170 6.399058

Si 9.858706 1.498350 8.485563

O 12.932710 5.847120 0.556660

O 12.932710 3.121670 0.556660

O 10.600310 4.484400 1.386100

O 10.721890 1.494800 1.760420

O 12.964830 4.484400 2.799080

O 13.161830 1.494800 3.191290

O 10.755330 0.166960 4.002710

O 10.755330 2.822640 4.002710

O 13.134270 5.803917 5.690376

O 13.131413 3.162676 5.704798

O 10.780071 4.481494 6.490799

O 10.587429 1.493575 6.913088

O 13.083941 4.498657 7.934456

O 12.967003 1.501386 8.322723

O 10.557173 0.135607 9.157114

O 10.545760 2.864154 9.146586

Mg 12.041480 7.474000 0.388370

Mg 9.506060 11.914340 2.261970

Mg 9.506060 9.012860 2.261970

Mg 11.846845 10.463304 4.525183

Mg 14.211802 7.477172 5.167907

Mg 11.874269 6.035082 7.406700

Mg 11.872155 8.949137 7.404560

Ni 9.517918 10.447748 9.521251

Si 12.225970 10.463600 1.221250

Si 11.516610 7.474000 3.313620

Si 13.896740 10.467718 6.403705

Si 9.844459 7.489655 8.388872

O 12.932710 11.826320 0.556660

O 12.932710 9.100870 0.556660

O 10.600310 10.463600 1.386100

O 10.721890 7.474000 1.760420

O 12.964840 10.463600 2.799080

O 13.161830 7.474000 3.191290

O 10.755330 6.146160 4.002710

O 10.755330 8.801840 4.002710

O 13.141850 11.790029 5.704732

O 13.145737 9.150161 5.692775

O 10.798994 10.469123 6.440721

O 10.590405 7.486966 6.837010

O 13.103594 10.461819 7.937558

O 12.973819 7.490145 8.295203

O 10.559858 6.140235 9.048450

O 10.534409 8.878111 9.030069

Mg 12.041480 13.453200 0.388370

Mg 9.506060 17.893540 2.261970

Mg 9.506060 14.992060 2.261970

Mg 11.844607 16.441896 4.521024

Mg 14.220468 13.447458 5.187816

Mg 11.869789 11.980897 7.422940

Mg 11.889380 14.890150 7.446934

Mg 9.706346 16.422471 9.256291

Si 12.225970 16.442800 1.221250

Si 11.516610 13.453200 3.313620

Si 13.877418 16.443132 6.402052

Si 9.839550 13.430933 8.425364

O 12.932710 17.805520 0.556660

O 12.932710 15.080070 0.556660

O 10.600310 16.442800 1.386100

O 10.721890 13.453200 1.760420

O 12.964840 16.442800 2.799080

O 13.161830 13.453200 3.191290

O 10.755330 12.125360 4.002710

O 10.755330 14.781030 4.002710

O 13.129817 17.764786 5.706083

O 13.132575 15.119887 5.705914

O 10.769574 16.438823 6.537514

O 10.592853 13.472914 6.875546
O 13.084826 16.441335 7.948019
O 12.962432 13.438069 8.334345
O 10.524862 12.031724 9.026304
O 10.539635 14.757502 9.169187
Mg 16.790480 1.494800 0.388370
Mg 14.255060 5.935140 2.261970
Mg 14.255060 3.033660 2.261970
Mg 16.585126 4.485117 4.524039
Mg 18.958936 1.491987 5.179072
Mg 16.620106 0.057598 7.455831
Mg 16.609093 2.961593 7.458419
Mg 14.345105 4.528293 9.359003
Si 16.974970 4.484400 1.221250
Si 16.265610 1.494800 3.313620
Si 18.625288 4.478437 6.388660
Si 14.586827 1.517704 8.494597
O 17.681710 5.847120 0.556660
O 17.681710 3.121670 0.556660
O 15.349310 4.484400 1.386100
O 15.470890 1.494800 1.760420
O 17.713830 4.484400 2.799080
O 17.910830 1.494800 3.191290
O 15.504330 0.166960 4.002710
O 15.504330 2.822640 4.002710
O 17.880011 5.800427 5.693110
O 17.874336 3.155423 5.701797
O 15.527713 4.483393 6.499716
O 15.328327 1.506102 6.927320
O 17.840039 4.486243 7.936731
O 17.725731 1.507298 8.321109
O 15.293926 0.160513 9.168157
O 15.256130 2.893089 9.162195
Mg 16.790480 7.474000 0.388370
Mg 14.255060 11.914340 2.261970
Mg 14.255060 9.012860 2.261970
Mg 16.585251 10.464325 4.526090
Mg 18.959114 7.472631 5.175059
Mg 16.620182 6.020604 7.437627
Mg 16.621748 8.928065 7.432504
Mg 14.345237 10.433553 9.393818
Si 16.974970 10.463600 1.221250
Si 16.265610 7.474000 3.313620
Si 18.628105 10.468209 6.390219
Si 14.599047 7.481889 8.432175
O 17.681710 11.826320 0.556660
O 17.681710 9.100870 0.556660
O 15.349310 10.463600 1.386100
O 15.470890 7.474000 1.760420
O 17.713840 10.463600 2.799080
O 17.910830 7.474000 3.191290
O 15.504330 6.146160 4.002710
O 15.504330 8.801840 4.002710
O 17.879958 11.792900 5.704182

| | | | |
|---|---|---|---|
| O | 17.884653 | 9.144166 | 5.694709 |
| O | 15.541183 | 10.468278 | 6.488296 |
| O | 15.341078 | 7.469303 | 6.873448 |
| O | 17.844508 | 10.460751 | 7.937340 |
| O | 17.732965 | 7.472133 | 8.301851 |
| O | 15.302941 | 6.122647 | 9.111622 |
| O | 15.312499 | 8.841334 | 9.087620 |
| Mg | 16.790480 | 13.453200 | 0.388370 |
| Mg | 14.255060 | 17.893540 | 2.261970 |
| Mg | 14.255060 | 14.992060 | 2.261970 |
| Mg | 16.590729 | 16.442076 | 4.523095 |
| Mg | 18.960945 | 13.452891 | 5.181248 |
| Mg | 16.610381 | 11.986677 | 7.463677 |
| Mg | 16.618801 | 14.889704 | 7.459214 |
| Mg | 14.444254 | 16.446651 | 9.274223 |
| Si | 16.974970 | 16.442800 | 1.221250 |
| Si | 16.265610 | 13.453200 | 3.313620 |
| Si | 18.618421 | 16.438504 | 6.403438 |
| Si | 14.586836 | 13.429534 | 8.494564 |
| O | 17.681710 | 17.805520 | 0.556660 |
| O | 17.681710 | 15.080070 | 0.556660 |
| O | 15.349310 | 16.442800 | 1.386100 |
| O | 15.470890 | 13.453200 | 1.760420 |
| O | 17.713840 | 16.442800 | 2.799080 |
| O | 17.910830 | 13.453200 | 3.191290 |
| O | 15.504330 | 12.125360 | 4.002710 |
| O | 15.504330 | 14.781030 | 4.002710 |
| O | 17.872210 | 17.761931 | 5.708243 |
| O | 17.870593 | 15.114909 | 5.710611 |
| O | 15.518323 | 16.443840 | 6.541251 |
| O | 15.327367 | 13.445134 | 6.926910 |
| O | 17.826949 | 16.442022 | 7.949985 |
| O | 17.727615 | 13.442385 | 8.325803 |
| O | 15.271356 | 12.063038 | 9.160373 |
| O | 15.287867 | 14.792730 | 9.166226 |
| C | 7.350636 | 10.065385 | 11.663539 |
| C | 8.212853 | 11.158379 | 11.588193 |
| C | 9.642436 | 10.975082 | 11.738647 |
| C | 10.154566 | 9.627280 | 11.835526 |
| C | 9.250174 | 8.504601 | 11.855714 |
| C | 7.833083 | 8.721552 | 11.803863 |
| C | 6.946912 | 7.597400 | 11.837977 |
| C | 7.445627 | 6.303210 | 11.894690 |
| C | 8.856819 | 6.054245 | 11.896792 |
| C | 9.768165 | 7.166120 | 11.879701 |
| C | 11.186758 | 6.938505 | 11.851152 |
| C | 11.703615 | 5.597568 | 11.828509 |
| C | 10.780997 | 4.496740 | 11.844118 |
| C | 9.397599 | 4.719176 | 11.885303 |
| C | 13.130404 | 5.397822 | 11.817457 |
| C | 14.005940 | 6.485534 | 11.804296 |
| C | 13.507346 | 7.833505 | 11.805968 |
| C | 12.090316 | 8.059452 | 11.834882 |
| C | 11.575955 | 9.402247 | 11.853008 |

C 12.471353 10.525152 11.855414
C 11.937539 11.857772 11.844703
C 10.570251 12.078616 11.773399
C 13.891243 10.273269 11.848725
C 14.391762 8.965403 11.813817
H 6.268443 10.230109 11.616305
H 7.800503 12.160480 11.411187
H 5.868215 7.771410 11.787032
H 6.758853 5.449987 11.921181
H 11.162217 3.469053 11.793722
H 8.710378 3.864468 11.961535
H 13.516869 4.372437 11.933549
H 15.090400 6.331322 11.794594
H 12.627074 12.709038 11.832566
H 10.185978 13.101279 11.692821
H 14.578849 11.129745 11.914874
H 15.471996 8.787475 11.787627

**Coronene V$_{MgO}$-[010]-fo (=)**

Number of atoms: 370

Mg 2.543480 1.494800 0.388370
Mg 0.008060 5.935140 2.261970
Mg 0.008060 3.033660 2.261970
Mg 2.340479 4.487194 4.513337
Mg 4.702964 1.480284 5.175311
Mg 2.369044 0.043884 7.430823
Mg 2.371410 2.945254 7.418711
Mg 0.169560 4.481728 9.284116
Si 2.727970 4.484400 1.221250
Si 2.018610 1.494800 3.313620
Si 4.380125 4.498510 6.388801
Si 0.352943 1.498735 8.465711
O 3.434710 5.847120 0.556660
O 3.434700 3.121670 0.556660
O 1.102310 4.484400 1.386100
O 1.223890 1.494800 1.760420
O 3.466830 4.484400 2.799080
O 3.663830 1.494800 3.191290
O 1.257330 0.166960 4.002710
O 1.257330 2.822640 4.002710
O 3.637061 5.816660 5.689888
O 3.648819 3.172786 5.686409
O 1.282984 4.482967 6.493538
O 1.085542 1.497702 6.895505
O 3.580768 4.486518 7.922297
O 3.465235 1.501738 8.297512
O 1.052535 0.142401 9.136037
O 1.060339 2.857296 9.130796
Mg 2.543480 7.474000 0.388370
Mg 0.008060 11.914340 2.261970
Mg 0.008060 9.012860 2.261970
Mg 2.354489 10.469973 4.533019
Mg 4.713020 7.508999 5.197941
Mg 2.352005 6.027035 7.419388

Mg 2.359251 8.940256 7.447477

Mg 0.169573 10.471243 9.281158

Si 2.727970 10.463600 1.221250

Si 2.018610 7.474000 3.313620

Si 4.385379 10.473300 6.430367

Si 0.335810 7.476595 8.465408

O 3.434710 11.826320 0.556660

O 3.434710 9.100870 0.556660

O 1.102310 10.463600 1.386100

O 1.223890 7.474000 1.760420

O 3.466830 10.463600 2.799080

O 3.663830 7.474000 3.191290

O 1.257330 6.146160 4.002710

O 1.257330 8.801840 4.002710

O 3.631256 11.787413 5.719120

O 3.628726 9.153126 5.722612

O 1.280888 10.468750 6.514932

O 1.071549 7.480819 6.897923

O 3.590950 10.468946 7.974622

O 3.415795 7.467579 8.302121

O 1.038565 6.114248 9.130323

O 1.030925 8.828514 9.147773

Mg 2.543480 13.453200 0.388370

Mg 0.008060 17.893540 2.261970

Mg 0.008060 14.992060 2.261970

Mg 2.344694 16.435478 4.519334

Mg 4.743499 13.437147 5.208346

Mg 2.387556 12.011819 7.455976

Mg 2.381332 14.899882 7.443669

Mg 0.178265 16.443664 9.282135

Si 2.727970 16.442800 1.221250

Si 2.018610 13.453200 3.313620

Si 4.377874 16.440275 6.394321

Si 0.352880 13.451869 8.470984

O 3.434710 17.805520 0.556660

O 3.434710 15.080070 0.556660

O 1.102310 16.442800 1.386100

O 1.223890 13.453200 1.760420

O 3.466830 16.442800 2.799080

O 3.663830 13.453200 3.191290

O 1.257330 12.125360 4.002710

O 1.257330 14.781030 4.002710

O 3.624941 17.762421 5.695503

O 3.637948 15.115003 5.702917

O 1.280717 16.443406 6.504444

O 1.090750 13.454430 6.904851

O 3.583376 16.454097 7.936890

O 3.485441 13.467544 8.311753

O 1.056895 12.098755 9.147223

O 1.052275 14.812323 9.141013

Mg 7.292480 1.494800 0.388370

Mg 4.757060 5.935140 2.261970

Mg 4.757060 3.033660 2.261970

Mg 7.023619 4.427818 4.475464

| | | | |
|---|---|---|---|
| Mg | 9.477310 | 1.505421 | 5.169280 |
| Mg | 7.121536 | 0.052819 | 7.424307 |
| Mg | 7.091132 | 2.953087 | 7.469447 |
| Mg | 4.827141 | 4.460221 | 9.350699 |
| Si | 7.476970 | 4.484400 | 1.221250 |
| Si | 6.767610 | 1.494800 | 3.313620 |
| Si | 9.198875 | 4.462370 | 6.576381 |
| Si | 5.082987 | 1.497826 | 8.463379 |
| O | 8.183700 | 5.847120 | 0.556660 |
| O | 8.183700 | 3.121670 | 0.556660 |
| O | 5.851310 | 4.484400 | 1.386100 |
| O | 5.972890 | 1.494800 | 1.760420 |
| O | 8.215830 | 4.484400 | 2.799080 |
| O | 8.412830 | 1.494800 | 3.191290 |
| O | 6.006330 | 0.166960 | 4.002710 |
| O | 6.006330 | 2.822640 | 4.002710 |
| O | 8.686941 | 6.091684 | 6.280775 |
| O | 8.376219 | 3.346054 | 5.673388 |
| O | 6.040013 | 4.507959 | 6.489277 |
| O | 5.826771 | 1.513046 | 6.896764 |
| O | 8.316883 | 4.424704 | 8.069956 |
| O | 8.227214 | 1.512188 | 8.264372 |
| O | 5.794879 | 0.156185 | 9.136926 |
| O | 5.765297 | 2.865652 | 9.140393 |
| Mg | 7.292480 | 7.474000 | 0.388370 |
| Mg | 4.757060 | 11.914340 | 2.261970 |
| Mg | 4.757060 | 9.012860 | 2.261970 |
| Mg | 7.139749 | 10.487498 | 4.598203 |
| Mg | 9.478880 | 7.637720 | 5.079657 |
| Mg | 6.979172 | 5.970280 | 7.592230 |
| Mg | 7.113400 | 8.964378 | 7.527778 |
| Mg | 4.938869 | 10.487460 | 9.343059 |
| Si | 7.476970 | 10.463600 | 1.221250 |
| Si | 6.767610 | 7.474000 | 3.313620 |
| Si | 9.134451 | 10.466530 | 6.566319 |
| Si | 5.026264 | 7.485350 | 8.475110 |
| O | 8.183700 | 11.826320 | 0.556660 |
| O | 8.183700 | 9.100870 | 0.556660 |
| O | 5.851310 | 10.463600 | 1.386100 |
| O | 5.972890 | 7.474000 | 1.760420 |
| O | 8.215830 | 10.463600 | 2.799080 |
| O | 8.412830 | 7.474000 | 3.191290 |
| O | 6.006330 | 6.146160 | 4.002710 |
| O | 6.006330 | 8.801840 | 4.002710 |
| O | 8.402711 | 11.781177 | 5.775570 |
| O | 8.379370 | 9.149043 | 5.757649 |
| O | 6.018332 | 10.468737 | 6.562836 |
| O | 5.813198 | 7.461904 | 6.936325 |
| O | 8.364712 | 10.415280 | 8.032227 |
| O | 8.126308 | 7.488991 | 8.447049 |
| O | 5.686658 | 6.104705 | 9.178809 |
| O | 5.727001 | 8.810616 | 9.166631 |
| Mg | 7.292480 | 13.453200 | 0.388370 |
| Mg | 4.757060 | 17.893540 | 2.261970 |

| | | | |
|---|---|---|---|
| Mg | 4.757060 | 14.992060 | 2.261970 |
| Mg | 7.096376 | 16.434920 | 4.520198 |
| Mg | 9.453325 | 13.437853 | 5.205510 |
| Mg | 7.206513 | 11.994427 | 7.590041 |
| Mg | 7.130893 | 14.911457 | 7.435843 |
| Mg | 4.917512 | 16.459549 | 9.287003 |
| Si | 7.476970 | 16.442800 | 1.221250 |
| Si | 6.767610 | 13.453200 | 3.313620 |
| Si | 9.130165 | 16.460791 | 6.381697 |
| Si | 5.108309 | 13.468009 | 8.487896 |
| O | 8.183700 | 17.805520 | 0.556660 |
| O | 8.183700 | 15.080070 | 0.556660 |
| O | 5.851310 | 16.442800 | 1.386100 |
| O | 5.972890 | 13.453200 | 1.760420 |
| O | 8.215830 | 16.442800 | 2.799080 |
| O | 8.412830 | 13.453200 | 3.191290 |
| O | 6.006330 | 12.125360 | 4.002710 |
| O | 6.006330 | 14.781030 | 4.002710 |
| O | 8.376087 | 17.793611 | 5.689731 |
| O | 8.371408 | 15.151323 | 5.677864 |
| O | 6.018781 | 16.454641 | 6.512118 |
| O | 5.860302 | 13.427886 | 6.936367 |
| O | 8.336776 | 16.470632 | 7.922227 |
| O | 8.222267 | 13.541919 | 8.351255 |
| O | 5.788407 | 12.138337 | 9.224335 |
| O | 5.796050 | 14.839891 | 9.142812 |
| Mg | 12.041480 | 1.494800 | 0.388370 |
| Mg | 9.506060 | 5.935140 | 2.261970 |
| Mg | 9.506060 | 3.033660 | 2.261970 |
| Mg | 11.850582 | 4.474698 | 4.527192 |
| Mg | 14.217744 | 1.493615 | 5.181406 |
| Mg | 11.875972 | 0.049998 | 7.425459 |
| Mg | 11.893040 | 2.954041 | 7.466552 |
| Mg | 9.613170 | 4.438907 | 9.543170 |
| Si | 12.225970 | 4.484400 | 1.221250 |
| Si | 11.516610 | 1.494800 | 3.313620 |
| Si | 13.899614 | 4.482982 | 6.400089 |
| Si | 9.848817 | 1.501837 | 8.450066 |
| O | 12.932710 | 5.847120 | 0.556660 |
| O | 12.932710 | 3.121670 | 0.556660 |
| O | 10.600310 | 4.484400 | 1.386100 |
| O | 10.721890 | 1.494800 | 1.760420 |
| O | 12.964830 | 4.484400 | 2.799080 |
| O | 13.161830 | 1.494800 | 3.191290 |
| O | 10.755330 | 0.166960 | 4.002710 |
| O | 10.755330 | 2.822640 | 4.002710 |
| O | 13.136919 | 5.811378 | 5.708778 |
| O | 13.143771 | 3.168780 | 5.702101 |
| O | 10.829089 | 4.459302 | 6.498733 |
| O | 10.586354 | 1.522418 | 6.882361 |
| O | 13.117047 | 4.495532 | 7.946527 |
| O | 12.974282 | 1.491671 | 8.311930 |
| O | 10.536083 | 0.146776 | 9.122621 |
| O | 10.544347 | 2.861447 | 9.111290 |

Mg 12.041480 7.474000 0.388370

Mg 9.506060 11.914340 2.261970

Mg 9.506060 9.012860 2.261970

Mg 11.824295 10.492462 4.541457

Mg 14.230170 7.475570 5.163147

Mg 11.956430 6.051239 7.435865

Mg 11.935415 8.961047 7.324382

Si 12.226845 10.461078 1.231547

Si 11.516610 7.474000 3.313620

Si 13.888210 10.463600 6.382630

Si 9.492196 6.952562 7.829420

O 12.930177 11.814482 0.555317

O 12.932710 9.100870 0.556660

O 10.600310 10.463600 1.386100

O 10.721890 7.474000 1.760420

O 12.964840 10.463600 2.799080

O 13.161830 7.474000 3.191290

O 10.755330 6.146160 4.002710

O 10.755330 8.801840 4.002710

O 13.128110 11.791550 5.692940

O 13.147168 9.138774 5.664776

O 10.794886 10.434035 6.481457

O 10.627189 7.621161 6.831001

O 13.098177 10.448704 7.915596

O 12.945065 7.523975 8.343304

O 10.426131 6.019311 8.805697

Mg 12.060950 13.450738 0.420037

Mg 9.512436 17.885438 2.259652

Mg 9.506060 14.992060 2.261970

Mg 11.837740 16.442800 4.520030

Mg 14.211300 13.453200 5.174280

Mg 11.823293 11.990423 7.466725

Mg 11.875275 14.902992 7.452635

Mg 9.677732 16.434508 9.266426

Si 12.224500 16.441245 1.231860

Si 11.509489 13.453815 3.318014

Si 13.888220 16.442800 6.382630

Si 9.847270 13.453200 8.472360

O 12.920399 17.801858 0.558451

O 12.929313 15.088484 0.557675

O 10.600310 16.442800 1.386100

O 10.721890 13.453200 1.760420

O 12.964840 16.442800 2.799080

O 13.161830 13.453200 3.191290

O 10.755330 12.125360 4.002710

O 10.755330 14.781030 4.002710

O 13.128120 17.770750 5.692940

O 13.128120 15.114850 5.692940

O 10.770486 16.470703 6.506453

O 10.572645 13.520901 6.876199

O 13.095654 16.452850 7.927417

O 12.979980 13.464947 8.313528

O 10.539403 12.068460 9.005554

O 10.569402 14.816092 9.171121

Mg 16.804521 1.493409 0.411867

Mg 14.257534 5.933739 2.256914

Mg 14.255060 3.033660 2.261970

Mg 16.586740 4.484400 4.520030

Mg 18.960290 1.494800 5.174280

Mg 16.629008 0.047379 7.437628

Mg 16.620264 2.952036 7.442991

Mg 14.383088 4.496265 9.367423

Si 16.973534 4.484494 1.232035

Si 16.260460 1.493940 3.311118

Si 18.637210 4.484400 6.382630

Si 14.596270 1.494800 8.472360

O 17.675031 5.841055 0.559692

O 17.675861 3.127918 0.560115

O 15.349310 4.484400 1.386100

O 15.470890 1.494800 1.760420

O 17.713830 4.484400 2.799080

O 17.910830 1.494800 3.191290

O 15.504330 0.166960 4.002710

O 15.504330 2.822640 4.002710

O 17.877110 5.812340 5.692940

O 17.877110 3.156450 5.692940

O 15.539652 4.485899 6.493995

O 15.335749 1.500081 6.907488

O 17.844099 4.484362 7.926494

O 17.728293 1.504039 8.310560

O 15.301661 0.141589 9.143059

O 15.275829 2.865891 9.144876

Mg 16.804019 7.476105 0.410514

Mg 14.254322 11.912924 2.265748

Mg 14.255060 9.012860 2.261970

Mg 16.586740 10.463600 4.520030

Mg 18.960300 7.474000 5.174280

Mg 16.620995 6.022370 7.434916

Mg 16.603774 8.934997 7.441478

Mg 14.312372 10.453410 9.396305

Si 16.973321 10.464337 1.232669

Si 16.262228 7.476153 3.309626

Si 18.637210 10.463600 6.382630

Si 14.596270 7.474000 8.472360

O 17.676771 11.818514 0.558235

O 17.676487 9.108169 0.559382

O 15.349310 10.463600 1.386100

O 15.470890 7.474000 1.760420

O 17.713840 10.463600 2.799080

O 17.910830 7.474000 3.191290

O 15.504330 6.146160 4.002710

O 15.504330 8.801840 4.002710

O 17.877120 11.791540 5.692940

O 17.877110 9.135650 5.692940

O 15.532637 10.456438 6.469124

O 15.311377 7.456777 6.899981

O 17.841248 10.460960 7.926254

O 17.711445 7.471454 8.304595

O 15.290945 6.112897 9.138829
O 15.287571 8.848169 9.106240
Mg 16.805512 13.452715 0.414113
Mg 14.253595 17.891225 2.257083
Mg 14.255060 14.992060 2.261970
Mg 16.586740 16.442800 4.520030
Mg 18.960300 13.453200 5.174280
Mg 16.604314 11.988410 7.449126
Mg 16.632381 14.892801 7.438234
Mg 14.433943 16.436095 9.272828
Si 16.973467 16.442151 1.232143
Si 16.260452 13.452059 3.312521
Si 18.637220 16.442800 6.382630
Si 14.596270 13.453200 8.472360
O 17.676811 17.798394 0.559748
O 17.676349 15.087870 0.558346
O 15.349310 16.442800 1.386100
O 15.470890 13.453200 1.760420
O 17.713840 16.442800 2.799080
O 17.910830 13.453200 3.191290
O 15.504330 12.125360 4.002710
O 15.504330 14.781030 4.002710
O 17.877120 17.770750 5.692940
O 17.877120 15.114850 5.692940
O 15.529970 16.447725 6.509132
O 15.338476 13.457884 6.901550
O 17.844777 16.444470 7.928563
O 17.729213 13.443178 8.312302
O 15.266110 12.067478 9.122842
O 15.315857 14.806226 9.144423
C 6.428235 9.544605 11.942521
C 7.107894 10.743367 11.769898
C 8.533558 10.763360 11.559576
C 9.244879 9.511133 11.517469
C 8.534605 8.274747 11.670448
C 7.114459 8.279520 11.882369
C 6.433718 7.040112 12.081242
C 7.115715 5.826045 12.058267
C 8.534639 5.793412 11.873628
C 9.256014 7.031326 11.703277
C 10.691911 7.025698 11.629512
C 11.422400 5.785201 11.714618
C 10.687882 4.558828 11.842332
C 9.284066 4.561637 11.919054
C 12.858304 5.817107 11.714669
C 13.549667 7.028095 11.656125
C 12.842879 8.272983 11.563258
C 11.406433 8.272490 11.521237
C 10.677020 9.513335 11.444947
C 11.384536 10.765564 11.380656
C 10.641271 11.993950 11.278732
C 9.251057 11.984214 11.472442
C 12.815085 10.747936 11.493408
C 13.527855 9.542042 11.592999

H 5.355638 9.540442 12.183664

H 6.575588 11.702192 11.796215

H 5.353717 7.058100 12.268225

H 6.587702 4.881535 12.241702

H 11.226515 3.603793 11.879996

H 8.749841 3.618020 12.096396

H 13.401201 4.874894 11.900106

H 14.644785 7.039487 11.687161

H 11.186079 12.942586 11.220334

H 8.689946 12.925429 11.483586

H 13.346138 11.708088 11.574253

H 14.611757 9.537473 11.774560

**Coronene $V_{MgO}$-[010]-fo (⊥)**

Number of atoms: 370

Mg 2.543480 1.494800 0.388370

Mg 0.008060 5.935140 2.261970

Mg 0.008060 3.033660 2.261970

Mg 2.345472 4.484020 4.519718

Mg 4.722515 1.490355 5.184853

Mg 2.372714 0.040767 7.442005

Mg 2.373030 2.936228 7.436688

Mg 0.180483 4.481282 9.277746

Si 2.727970 4.484400 1.221250

Si 2.018610 1.494800 3.313620

Si 4.384098 4.478629 6.398617

Si 0.345846 1.486548 8.475110

O 3.434710 5.847120 0.556660

O 3.434700 3.121670 0.556660

O 1.102310 4.484400 1.386100

O 1.223890 1.494800 1.760420

O 3.466830 4.484400 2.799080

O 3.663830 1.494800 3.191290

O 1.257330 0.166960 4.002710

O 1.257330 2.822640 4.002710

O 3.639107 5.803028 5.703022

O 3.633548 3.159858 5.699106

O 1.261893 4.476930 6.519821

O 1.080817 1.489917 6.906272

O 3.582480 4.475714 7.937692

O 3.478079 1.488907 8.305013

O 1.054218 0.134317 9.146299

O 1.047181 2.844998 9.142209

Mg 2.543480 7.474000 0.388370

Mg 0.008060 11.914340 2.261970

Mg 0.008060 9.012860 2.261970

Mg 2.347379 10.462555 4.520538

Mg 4.734336 7.475898 5.196816

Mg 2.380658 6.021728 7.438319

Mg 2.374377 8.919233 7.422732

Mg 0.183065 10.459617 9.275675

Si 2.727970 10.463600 1.221250

Si 2.018610 7.474000 3.313620

Si 4.390928 10.457879 6.394915

Si 0.352485 7.469449 8.461009

O 3.434710 11.826320 0.556660

O 3.434710 9.100870 0.556660

O 1.102310 10.463600 1.386100

O 1.223890 7.474000 1.760420

O 3.466830 10.463600 2.799080

O 3.663830 7.474000 3.191290

O 1.257330 6.146160 4.002710

O 1.257330 8.801840 4.002710

O 3.641920 11.779074 5.694814

O 3.641744 9.138210 5.695139

O 1.281852 10.461007 6.501015

O 1.090296 7.464362 6.895188

O 3.577309 10.458580 7.928731

O 3.481782 7.479996 8.295405

O 1.053561 6.114365 9.135778

O 1.053370 8.823667 9.133662

Mg 2.543480 13.453200 0.388370

Mg 0.008060 17.893540 2.261970

Mg 0.008060 14.992060 2.261970

Mg 2.341206 16.442253 4.519898

Mg 4.714653 13.449976 5.171444

Mg 2.374965 12.001689 7.426780

Mg 2.369479 14.900465 7.434118

Mg 0.184340 16.435535 9.281802

Si 2.727970 16.442800 1.221250

Si 2.018610 13.453200 3.313620

Si 4.377490 16.441985 6.393939

Si 0.351129 13.446541 8.470363

O 3.434710 17.805520 0.556660

O 3.434710 15.080070 0.556660

O 1.102310 16.442800 1.386100

O 1.223890 13.453200 1.760420

O 3.466830 16.442800 2.799080

O 3.663830 13.453200 3.191290

O 1.257330 12.125360 4.002710

O 1.257330 14.781030 4.002710

O 3.628569 17.764572 5.701509

O 3.629955 15.121550 5.695135

O 1.266891 16.439977 6.520879

O 1.084387 13.450122 6.900573

O 3.585183 16.436310 7.938152

O 3.473638 13.454572 8.292625

O 1.059679 12.091893 9.140114

O 1.055572 14.801445 9.144472

Mg 7.292480 1.494800 0.388370

Mg 4.757060 5.935140 2.261970

Mg 4.757060 3.033660 2.261970

Mg 7.094396 4.484138 4.528267

Mg 9.472555 1.492007 5.199921

Mg 7.144512 0.035937 7.472863

Mg 7.128120 2.937956 7.462915

Mg 4.903978 4.469037 9.299921

Si 7.476970 4.484400 1.221250

Si 6.767610 1.494800 3.313620

Si 9.122712 4.502744 6.437931

Si 5.098618 1.484623 8.474756

O 8.183700 5.847120 0.556660

O 8.183700 3.121670 0.556660

O 5.851310 4.484400 1.386100

O 5.972890 1.494800 1.760420

O 8.215830 4.484400 2.799080

O 8.412830 1.494800 3.191290

O 6.006330 0.166960 4.002710

O 6.006330 2.822640 4.002710

O 8.389765 5.839122 5.751952

O 8.393211 3.188113 5.714539

O 6.027965 4.482910 6.525835

O 5.845148 1.486279 6.913164

O 8.334430 4.484592 7.979760

O 8.226312 1.495631 8.333410

O 5.796057 0.129037 9.153881

O 5.792441 2.843514 9.150911

Mg 7.292480 7.474000 0.388370

Mg 4.757060 11.914340 2.261970

Mg 4.757060 9.012860 2.261970

Mg 7.109545 10.459574 4.533134

Mg 9.434303 7.495721 5.190428

Mg 7.114580 6.019539 7.486499

Mg 7.188321 8.933398 7.493268

Mg 4.898583 10.451615 9.278828

Si 7.476970 10.463600 1.221250

Si 6.767610 7.474000 3.313620

Si 9.165452 10.446490 6.399213

Si 5.100372 7.474843 8.484944

O 8.183700 11.826320 0.556660

O 8.183700 9.100870 0.556660

O 5.851310 10.463600 1.386100

O 5.972890 7.474000 1.760420

O 8.215830 10.463600 2.799080

O 8.412830 7.474000 3.191290

O 6.006330 6.146160 4.002710

O 6.006330 8.801840 4.002710

O 8.397522 11.759246 5.671188

O 8.381045 9.125740 5.708540

O 6.032421 10.448515 6.544151

O 5.862415 7.492114 6.932375

O 8.468938 10.470785 7.936918

O 8.161655 7.429790 8.461662

O 5.769573 6.104244 9.157520

O 5.792600 8.822034 9.196175

Mg 7.292480 13.453200 0.388370

Mg 4.757060 17.893540 2.261970

Mg 4.757060 14.992060 2.261970

Mg 7.105015 16.447051 4.528925

Mg 9.432164 13.450617 5.121327

Mg 7.151075 11.969327 7.433255

Mg 7.115357 14.909439 7.413434

Mg 4.930900 16.425772 9.275373

Si 7.476970 16.442800 1.221250

Si 6.767610 13.453200 3.313620

Si 9.142973 16.443610 6.422308

Si 5.094023 13.444170 8.443764

O 8.183700 17.805520 0.556660

O 8.183700 15.080070 0.556660

O 5.851310 16.442800 1.386100

O 5.972890 13.453200 1.760420

O 8.215830 16.442800 2.799080

O 8.412830 13.453200 3.191290

O 6.006330 12.125360 4.002710

O 6.006330 14.781030 4.002710

O 8.381529 17.769331 5.734191

O 8.378992 15.136939 5.698536

O 6.019808 16.445486 6.530785

O 5.836307 13.444544 6.888413

O 8.366482 16.418697 7.973927

O 8.211998 13.465074 8.225622

O 5.780109 12.082763 9.118725

O 5.803608 14.784298 9.156900

Mg 12.041480 1.494800 0.388370

Mg 9.506060 5.935140 2.261970

Mg 9.506060 3.033660 2.261970

Mg 11.846460 4.485962 4.528345

Mg 14.210494 1.506249 5.200684

Mg 11.867686 0.027846 7.461083

Mg 11.866319 2.946253 7.488903

Mg 9.672140 4.455062 9.335136

Si 12.225970 4.484400 1.221250

Si 11.516610 1.494800 3.313620

Si 13.839796 4.523380 6.438062

Si 9.846859 1.459577 8.503708

O 12.932710 5.847120 0.556660

O 12.932710 3.121670 0.556660

O 10.600310 4.484400 1.386100

O 10.721890 1.494800 1.760420

O 12.964830 4.484400 2.799080

O 13.161830 1.494800 3.191290

O 10.755330 0.166960 4.002710

O 10.755330 2.822640 4.002710

O 13.085711 5.849061 5.749986

O 13.108012 3.194102 5.731457

O 10.759390 4.540844 6.600427

O 10.585226 1.497606 6.933370

O 13.056948 4.514208 7.991224

O 12.959536 1.490411 8.342538

O 10.516505 0.075718 9.147402

O 10.542683 2.818940 9.188011

Mg 12.041480 7.474000 0.388370

Mg 9.506060 11.914340 2.261970

Mg 9.506060 9.012860 2.261970

Mg 11.848683 10.476860 4.500756

Mg 14.227442 7.464488 5.161209
Mg 11.815596 6.070128 7.570782
Mg 11.958729 8.943620 7.266686
Si 12.225991 10.459785 1.228565
Si 11.516610 7.474000 3.313620
Si 13.927410 10.477075 6.371194
Si 9.750686 7.500058 8.817703
O 12.928538 11.816067 0.557401
O 12.932710 9.100870 0.556660
O 10.600310 10.463600 1.386100
O 10.721890 7.474000 1.760420
O 12.964840 10.463600 2.799080
O 13.161830 7.474000 3.191290
O 10.755330 6.146160 4.002710
O 10.755330 8.801840 4.002710
O 13.160149 11.782455 5.661724
O 13.177101 9.149302 5.650078
O 10.821650 10.431129 6.395093
O 10.512828 7.648070 7.257729
O 13.108078 10.453364 7.897649
O 12.967514 7.536927 8.315902
O 10.553113 6.115067 9.345784
Mg 12.057804 13.451845 0.413577
Mg 9.505693 17.893092 2.257938
Mg 9.506060 14.992060 2.261970
Mg 11.843059 16.444764 4.530937
Mg 14.222048 13.467338 5.165220
Mg 11.879284 11.971724 7.324335
Mg 11.897384 14.892143 7.383831
Mg 9.671036 16.340901 9.412457
Si 12.223968 16.442526 1.231200
Si 11.510993 13.454181 3.312087
Si 13.890578 16.452397 6.411302
Si 9.825679 13.467255 8.275927
O 12.921532 17.801246 0.557747
O 12.928146 15.087035 0.559148
O 10.600310 16.442800 1.386100
O 10.721890 13.453200 1.760420
O 12.964840 16.442800 2.799080
O 13.161830 13.453200 3.191290
O 10.755330 12.125360 4.002710
O 10.755330 14.781030 4.002710
O 13.133052 17.775004 5.721283
O 13.144411 15.136902 5.689540
O 10.785636 16.447234 6.486792
O 10.609730 13.459771 6.761196
O 13.089413 16.427066 7.946471
O 12.947998 13.405104 8.269541
O 10.412705 11.963152 8.841516
O 10.585243 14.734386 9.007460
Mg 16.804665 1.493993 0.411461
Mg 14.255050 5.933492 2.252636
Mg 14.255060 3.033660 2.261970
Mg 16.587706 4.481335 4.520842

Mg 18.956159 1.485177 5.181123

Mg 16.604974 0.020409 7.468125

Mg 16.609230 2.925924 7.458778

Mg 14.451171 4.478284 9.283715

Si 16.973531 4.484407 1.231940

Si 16.260483 1.492371 3.310949

Si 18.615939 4.471192 6.398360

Si 14.580987 1.462996 8.508705

O 17.674156 5.841182 0.559714

O 17.675126 3.128334 0.559587

O 15.349310 4.484400 1.386100

O 15.470890 1.494800 1.760420

O 17.713830 4.484400 2.799080

O 17.910830 1.494800 3.191290

O 15.504330 0.166960 4.002710

O 15.504330 2.822640 4.002710

O 17.867415 5.790951 5.697261

O 17.859249 3.150584 5.708507

O 15.483991 4.490046 6.560593

O 15.319139 1.477399 6.938494

O 17.822248 4.475360 7.944847

O 17.719092 1.477750 8.324297

O 15.244677 0.082704 9.170180

O 15.291600 2.824451 9.174415

Mg 16.803982 7.475449 0.409637

Mg 14.256345 11.912493 2.260171

Mg 14.255060 9.012860 2.261970

Mg 16.593503 10.467138 4.522406

Mg 18.961628 7.466712 5.176360

Mg 16.620611 6.023596 7.439119

Mg 16.627920 8.933621 7.426271

Mg 14.327980 10.451478 9.404075

Si 16.973712 10.464149 1.232537

Si 16.262306 7.476830 3.308759

Si 18.635176 10.463522 6.381863

Si 14.610087 7.506976 8.447247

O 17.675530 11.819179 0.558899

O 17.675302 9.108407 0.559024

O 15.349310 10.463600 1.386100

O 15.470890 7.474000 1.760420

O 17.713840 10.463600 2.799080

O 17.910830 7.474000 3.191290

O 15.504330 6.146160 4.002710

O 15.504330 8.801840 4.002710

O 17.886988 11.785768 5.691723

O 17.889067 9.137226 5.689860

O 15.562787 10.461283 6.452378

O 15.331265 7.463178 6.882295

O 17.851605 10.461810 7.925454

O 17.728417 7.467439 8.294576

O 15.272397 6.143670 9.147381

O 15.321133 8.872832 9.065092

Mg 16.805484 13.453605 0.412752

Mg 14.254205 17.891482 2.258429

Mg 14.255060 14.992060 2.261970
Mg 16.586486 16.441183 4.530640
Mg 18.959352 13.452695 5.177336
Mg 16.617158 11.991237 7.441806
Mg 16.606178 14.894593 7.453575
Mg 14.335742 16.381513 9.395416
Si 16.973492 16.442605 1.232108
Si 16.260951 13.452867 3.311765
Si 18.619479 16.434703 6.399405
Si 14.576146 13.418937 8.432025
O 17.675677 17.798185 0.559158
O 17.676101 15.087645 0.559081
O 15.349310 16.442800 1.386100
O 15.470890 13.453200 1.760420
O 17.713840 16.442800 2.799080
O 17.910830 13.453200 3.191290
O 15.504330 12.125360 4.002710
O 15.504330 14.781030 4.002710
O 17.873749 17.760174 5.709460
O 17.875102 15.113602 5.702591
O 15.533161 16.441869 6.502609
O 15.333301 13.448631 6.873812
O 17.833404 16.430053 7.946850
O 17.726713 13.445074 8.314918
O 15.275151 12.050919 9.064831
O 15.265765 14.780964 9.111633
C 7.667463 13.043897 11.555317
C 7.614968 11.666184 11.357341
C 8.801449 10.880396 11.181819
C 10.056945 11.523772 11.416154
C 10.128925 12.937822 11.659640
C 8.924605 13.719738 11.675787
C 9.033965 15.150798 11.818781
C 10.284389 15.780055 11.915916
C 11.502544 15.009177 11.882163
C 11.408625 13.579378 11.776085
C 12.596978 12.770715 11.817728
C 13.876690 13.381791 12.035605
C 13.953034 14.817080 12.106933
C 12.803787 15.609949 12.018419
C 15.025599 12.539846 12.210013
C 14.926713 11.154824 12.115003
C 13.679506 10.521171 11.763031
C 12.503966 11.343894 11.635295
C 11.246950 10.739482 11.299156
C 11.191785 9.392739 10.781810
C 9.961288 8.896434 10.176101
C 8.788799 9.590232 10.532758
C 12.368510 8.586328 10.944766
C 13.550741 9.110251 11.482462
H 6.742704 13.631966 11.547806
H 6.638595 11.186168 11.249912
H 8.113137 15.741096 11.924241
H 10.346891 16.863000 12.093692

H 14.929672 15.278506 12.299286

H 12.871355 16.698007 12.158674

H 15.995550 13.004067 12.417732

H 15.811577 10.526887 12.273997

H 7.814501 9.182804 10.223848

H 12.300492 7.530891 10.660452

H 14.418781 8.458413 11.633487

H 9.670828 11.288420 8.685120

**Benzocoronene V$_{MgO}$-[010]-fo (=)**

Number of atoms: 376

Mg 2.543480 1.494800 0.388370

Mg 0.008060 5.935140 2.261970

Mg 0.008060 3.033660 2.261970

Mg 2.349413 4.484034 4.513511

Mg 4.718764 1.484945 5.178752

Mg 2.387395 0.039511 7.434756

Mg 2.385024 2.933246 7.426984

Mg 0.180197 4.476707 9.275670

Si 2.727970 4.484400 1.221250

Si 2.018610 1.494800 3.313620

Si 4.383111 4.474139 6.381228

Si 0.357810 1.492104 8.460214

O 3.434710 5.847120 0.556660

O 3.434700 3.121670 0.556660

O 1.102310 4.484400 1.386100

O 1.223890 1.494800 1.760420

O 3.466830 4.484400 2.799080

O 3.663830 1.494800 3.191290

O 1.257330 0.166960 4.002710

O 1.257330 2.822640 4.002710

O 3.635066 5.791557 5.680735

O 3.631511 3.148391 5.692466

O 1.278133 4.478375 6.505766

O 1.092679 1.487334 6.893773

O 3.583735 4.472678 7.921772

O 3.479975 1.487007 8.308330

O 1.055560 0.138599 9.136486

O 1.059047 2.848266 9.129103

Mg 2.543480 7.474000 0.388370

Mg 0.008060 11.914340 2.261970

Mg 0.008060 9.012860 2.261970

Mg 2.367377 10.463880 4.537506

Mg 4.721262 7.487188 5.175313

Mg 2.375518 6.016300 7.421154

Mg 2.384946 8.918481 7.435449

Mg 0.216160 10.460009 9.296340

Si 2.727970 10.463600 1.221250

Si 2.018610 7.474000 3.313620

Si 4.402591 10.456773 6.435574

Si 0.348383 7.466627 8.466974

O 3.434710 11.826320 0.556660

O 3.434710 9.100870 0.556660

O 1.102310 10.463600 1.386100

O 1.223890 7.474000 1.760420

O 3.466830 10.463600 2.799080

O 3.663830 7.474000 3.191290

O 1.257330 6.146160 4.002710

O 1.257330 8.801840 4.002710

O 3.638968 11.773012 5.717969

O 3.634850 9.142710 5.718943

O 1.278413 10.462337 6.511966

O 1.075722 7.463122 6.900066

O 3.588537 10.460251 7.966732

O 3.463818 7.448218 8.266273

O 1.044865 6.114749 9.158986

O 1.058915 8.807631 9.150904

Mg 2.543480 13.453200 0.388370

Mg 0.008060 17.893540 2.261970

Mg 0.008060 14.992060 2.261970

Mg 2.350884 16.439082 4.517557

Mg 4.730355 13.425806 5.181262

Mg 2.392590 12.003958 7.439056

Mg 2.389815 14.896806 7.428792

Mg 0.186295 16.441617 9.274630

Si 2.727970 16.442800 1.221250

Si 2.018610 13.453200 3.313620

Si 4.389671 16.432035 6.387063

Si 0.357841 13.458917 8.470226

O 3.434710 17.805520 0.556660

O 3.434710 15.080070 0.556660

O 1.102310 16.442800 1.386100

O 1.223890 13.453200 1.760420

O 3.466830 16.442800 2.799080

O 3.663830 13.453200 3.191290

O 1.257330 12.125360 4.002710

O 1.257330 14.781030 4.002710

O 3.635865 17.756273 5.695718

O 3.638829 15.112730 5.691963

O 1.279239 16.440161 6.511910

O 1.085621 13.460855 6.903071

O 3.595044 16.437877 7.931734

O 3.493086 13.463803 8.275136

O 1.061975 12.110612 9.144514

O 1.063838 14.807295 9.165386

Mg 7.292480 1.494800 0.388370

Mg 4.757060 5.935140 2.261970

Mg 4.757060 3.033660 2.261970

Mg 7.087554 4.486966 4.518663

Mg 9.465040 1.489428 5.175790

Mg 7.126164 0.022380 7.447892

Mg 7.120161 2.926127 7.436017

Mg 4.909903 4.470958 9.275433

Si 7.476970 4.484400 1.221250

Si 6.767610 1.494800 3.313620

Si 9.123575 4.484409 6.407574

Si 5.100088 1.475775 8.476461

O 8.183700 5.847120 0.556660

O 8.183700 3.121670 0.556660

O 5.851310 4.484400 1.386100

O 5.972890 1.494800 1.760420

O 8.215830 4.484400 2.799080

O 8.412830 1.494800 3.191290

O 6.006330 0.166960 4.002710

O 6.006330 2.822640 4.002710

O 8.393875 5.818482 5.722031

O 8.389453 3.166032 5.695526

O 6.026907 4.461296 6.500634

O 5.839568 1.474719 6.908814

O 8.323698 4.465141 7.944697

O 8.234448 1.476756 8.306613

O 5.794418 0.114174 9.147689

O 5.797962 2.838039 9.144492

Mg 7.292480 7.474000 0.388370

Mg 4.757060 11.914340 2.261970

Mg 4.757060 9.012860 2.261970

Mg 7.120210 10.461136 4.571366

Mg 9.428166 7.502557 5.183285

Mg 7.106362 6.008016 7.439730

Mg 7.145955 8.939517 7.489029

Mg 4.843989 10.451881 9.439832

Si 7.476970 10.463600 1.221250

Si 6.767610 7.474000 3.313620

Si 9.157022 10.427244 6.494456

Si 5.085403 7.470466 8.408540

O 8.183700 11.826320 0.556660

O 8.183700 9.100870 0.556660

O 5.851310 10.463600 1.386100

O 5.972890 7.474000 1.760420

O 8.215830 10.463600 2.799080

O 8.412830 7.474000 3.191290

O 6.006330 6.146160 4.002710

O 6.006330 8.801840 4.002710

O 8.421184 11.756031 5.740442

O 8.401347 9.130001 5.709445

O 6.043236 10.449407 6.490726

O 5.857583 7.456145 6.863437

O 8.433666 10.396842 7.987793

O 8.170152 7.417611 8.406932

O 5.775694 6.103086 9.094604

O 5.751964 8.843262 9.043608

Mg 7.292480 13.453200 0.388370

Mg 4.757060 17.893540 2.261970

Mg 4.757060 14.992060 2.261970

Mg 7.090047 16.435904 4.519169

Mg 9.447159 13.405520 5.180421

Mg 7.176064 11.925068 7.508332

Mg 7.133611 14.875502 7.417365

Mg 4.929981 16.403128 9.281487

Si 7.476970 16.442800 1.221250

Si 6.767610 13.453200 3.313620

Si 9.134755 16.425899 6.391329

Si 5.118092 13.422358 8.409086

O 8.183700 17.805520 0.556660

O 8.183700 15.080070 0.556660

O 5.851310 16.442800 1.386100

O 5.972890 13.453200 1.760420

O 8.215830 16.442800 2.799080

O 8.412830 13.453200 3.191290

O 6.006330 12.125360 4.002710

O 6.006330 14.781030 4.002710

O 8.390612 17.752009 5.702191

O 8.396449 15.100610 5.698347

O 6.033683 16.435779 6.502128

O 5.881685 13.421014 6.863083

O 8.349012 16.418424 7.937500

O 8.241241 13.453189 8.335365

O 5.782164 12.050089 9.056312

O 5.828645 14.787679 9.081459

Mg 12.041480 1.494800 0.388370

Mg 9.506060 5.935140 2.261970

Mg 9.506060 3.033660 2.261970

Mg 11.840052 4.488612 4.523686

Mg 14.211283 1.512131 5.179033

Mg 11.883553 0.038667 7.433541

Mg 11.871164 2.948483 7.454189

Mg 9.645965 4.430985 9.299099

Si 12.225970 4.484400 1.221250

Si 11.516610 1.494800 3.313620

Si 13.857706 4.517617 6.418507

Si 9.854646 1.468955 8.468023

O 12.932710 5.847120 0.556660

O 12.932710 3.121670 0.556660

O 10.600310 4.484400 1.386100

O 10.721890 1.494800 1.760420

O 12.964830 4.484400 2.799080

O 13.161830 1.494800 3.191290

O 10.755330 0.166960 4.002710

O 10.755330 2.822640 4.002710

O 13.097916 5.846029 5.742330

O 13.118598 3.193237 5.712774

O 10.763541 4.515959 6.555595

O 10.592842 1.491993 6.901710

O 13.071159 4.500867 7.968326

O 12.975627 1.494320 8.310849

O 10.560085 0.108465 9.129124

O 10.552966 2.819617 9.154938

Mg 12.041480 7.474000 0.388370

Mg 9.506060 11.914340 2.261970

Mg 9.506060 9.012860 2.261970

Mg 11.822935 10.473993 4.511462

Mg 14.230515 7.465158 5.160301

Mg 11.827550 6.041720 7.531567

Mg 11.946603 8.926124 7.267857

Si 12.225341 10.459287 1.229149

Si 11.516610 7.474000 3.313620
Si 13.888210 10.463600 6.382630
Si 9.762745 7.468196 8.686813
O 12.928020 11.816518 0.557633
O 12.932710 9.100870 0.556660
O 10.600310 10.463600 1.386100
O 10.721890 7.474000 1.760420
O 12.964840 10.463600 2.799080
O 13.161830 7.474000 3.191290
O 10.755330 6.146160 4.002710
O 10.755330 8.801840 4.002710
O 13.128110 11.791550 5.692940
O 13.163321 9.141536 5.646344
O 10.823840 10.436573 6.454731
O 10.532418 7.608483 7.150600
O 13.095733 10.432736 7.896635
O 12.952413 7.513074 8.339887
O 10.538729 6.066580 9.220904
Mg 12.055556 13.450994 0.410947
Mg 9.504109 17.892766 2.256309
Mg 9.506060 14.992060 2.261970
Mg 11.837740 16.442800 4.520030
Mg 14.211300 13.453200 5.174280
Mg 11.886393 11.953218 7.389422
Mg 11.902931 14.878384 7.435850
Mg 9.693553 16.407367 9.273380
Si 12.223576 16.442034 1.231037
Si 11.510708 13.454283 3.310341
Si 13.888220 16.442800 6.382630
Si 9.847270 13.453200 8.472360
O 12.921479 17.801236 0.558922
O 12.927545 15.086157 0.560235
O 10.600310 16.442800 1.386100
O 10.721890 13.453200 1.760420
O 12.964840 16.442800 2.799080
O 13.161830 13.453200 3.191290
O 10.755330 12.125360 4.002710
O 10.755330 14.781030 4.002710
O 13.128120 17.770750 5.692940
O 13.128120 15.114850 5.692940
O 10.776992 16.416669 6.536775
O 10.596515 13.413844 6.943508
O 13.095987 16.433054 7.931622
O 12.968889 13.420374 8.312327
O 10.554594 12.006459 9.091444
O 10.570983 14.753451 9.217877
Mg 16.803950 1.494298 0.409748
Mg 14.254095 5.934527 2.251625
Mg 14.255060 3.033660 2.261970
Mg 16.586740 4.484400 4.520030
Mg 18.960290 1.494800 5.174280
Mg 16.632004 0.049950 7.433813
Mg 16.625867 2.945461 7.433405
Mg 14.429860 4.476320 9.295090

Si 16.973516 4.484532 1.231954
Si 16.261262 1.492590 3.310001
Si 18.637210 4.484400 6.382630
Si 14.596270 1.494800 8.472360
O 17.673481 5.841351 0.559909
O 17.674452 3.128125 0.559733
O 15.349310 4.484400 1.386100
O 15.470890 1.494800 1.760420
O 17.713830 4.484400 2.799080
O 17.910830 1.494800 3.191290
O 15.504330 0.166960 4.002710
O 15.504330 2.822640 4.002710
O 17.877110 5.812340 5.692940
O 17.877110 3.156450 5.692940
O 15.502460 4.499720 6.528333
O 15.330302 1.498011 6.902874
O 17.838859 4.485285 7.926619
O 17.732075 1.498202 8.298875
O 15.302719 0.141011 9.140415
O 15.303244 2.848064 9.143198
Mg 16.803385 7.475431 0.408095
Mg 14.254618 11.912054 2.257746
Mg 14.255060 9.012860 2.261970
Mg 16.586740 10.463600 4.520030
Mg 18.960300 7.474000 5.174280
Mg 16.629488 6.028967 7.433360
Mg 16.625608 8.935427 7.451621
Mg 14.344185 10.464367 9.327984
Si 16.973699 10.464011 1.232341
Si 16.263008 7.476287 3.307362
Si 18.637210 10.463600 6.382630
Si 14.596270 7.474000 8.472360
O 17.674572 11.819458 0.559059
O 17.674341 9.108107 0.559133
O 15.349310 10.463600 1.386100
O 15.470890 7.474000 1.760420
O 17.713840 10.463600 2.799080
O 17.910830 7.474000 3.191290
O 15.504330 6.146160 4.002710
O 15.504330 8.801840 4.002710
O 17.877120 11.791540 5.692940
O 17.877110 9.135650 5.692940
O 15.530038 10.465090 6.498732
O 15.321026 7.475660 6.909165
O 17.859171 10.464324 7.939929
O 17.719672 7.472083 8.309549
O 15.282570 6.119623 9.149571
O 15.268780 8.843466 9.144415
Mg 16.804715 13.453268 0.410883
Mg 14.253904 17.892031 2.255542
Mg 14.255060 14.992060 2.261970
Mg 16.586740 16.442800 4.520030
Mg 18.960300 13.453200 5.174280
Mg 16.628352 11.997189 7.453412

Mg 16.634243 14.897381 7.434766

Mg 14.432087 16.445712 9.281512

Si 16.973593 16.442650 1.232027

Si 16.261458 13.452040 3.310398

Si 18.637220 16.442800 6.382630

Si 14.596270 13.453200 8.472360

O 17.674983 17.798557 0.559499

O 17.674797 15.087440 0.559186

O 15.349310 16.442800 1.386100

O 15.470890 13.453200 1.760420

O 17.713840 16.442800 2.799080

O 17.910830 13.453200 3.191290

O 15.504330 12.125360 4.002710

O 15.504330 14.781030 4.002710

O 17.877120 17.770750 5.692940

O 17.877120 15.114850 5.692940

O 15.527324 16.442095 6.511198

O 15.332708 13.456006 6.907607

O 17.842141 16.442205 7.929372

O 17.729938 13.458367 8.315174

O 15.273593 12.079219 9.136271

O 15.291890 14.805956 9.150106

C 11.727809 10.568363 10.631754

C 11.564591 9.228380 10.457390

C 10.213606 8.602171 10.299362

C 9.113374 9.485961 10.833073

C 9.261250 10.885020 10.900539

C 10.577341 11.538162 10.477361

C 8.178626 11.714939 11.377383

C 8.272900 13.143719 11.511022

C 7.162154 13.927594 11.799630

C 5.857256 13.352805 11.925472

C 4.660830 14.144080 11.977548

C 3.396018 13.565388 11.927293

C 3.249886 12.138721 11.927156

C 1.964345 11.498580 11.970253

C 1.847070 10.110606 11.979465

C 3.006463 9.262370 11.932208

C 2.916849 7.832137 11.924401

C 4.068276 7.053986 11.981883

C 5.375846 7.637859 11.947101

C 6.568646 6.853021 11.853198

C 7.780584 7.440698 11.521856

C 7.913999 8.860076 11.343089

C 6.785552 9.678882 11.677488

C 5.499771 9.064130 11.898730

C 4.310849 9.878671 11.931849

C 4.434186 11.319538 11.933162

C 5.742719 11.925828 11.891171

C 6.910097 11.107221 11.677811

H 12.693062 10.995414 10.962394

H 12.414582 8.537392 10.523889

H 10.278285 7.698167 10.938400

H 10.774508 12.421043 11.114718

H 9.238369 13.642589 11.367639

H 7.267815 15.016222 11.885528

H 4.758542 15.237631 12.047134

H 2.494811 14.187923 11.871806

H 1.072060 12.132733 12.019277

H 0.863310 9.631293 12.074093

H 1.931564 7.357369 11.860057

H 3.979562 5.959554 12.023496

H 6.504545 5.766575 11.989852

H 8.656998 6.795158 11.403146

**Benzocoronene V$_{MgO}$-[010]-fo (⊥)**

Number of atoms: 376

Mg 2.543480 1.494800 0.388370

Mg 0.008060 5.935140 2.261970

Mg 0.008060 3.033660 2.261970

Mg 2.341925 4.482783 4.514004

Mg 4.713055 1.477656 5.174084

Mg 2.388902 0.035878 7.449381

Mg 2.373756 2.933854 7.429038

Mg 0.179043 4.475052 9.278932

Si 2.727970 4.484400 1.221250

Si 2.018610 1.494800 3.313620

Si 4.372388 4.468551 6.378021

Si 0.355688 1.487179 8.474750

O 3.434710 5.847120 0.556660

O 3.434700 3.121670 0.556660

O 1.102310 4.484400 1.386100

O 1.223890 1.494800 1.760420

O 3.466830 4.484400 2.799080

O 3.663830 1.494800 3.191290

O 1.257330 0.166960 4.002710

O 1.257330 2.822640 4.002710

O 3.625101 5.792679 5.685734

O 3.617573 3.148016 5.688283

O 1.258610 4.481850 6.519873

O 1.086363 1.479999 6.906546

O 3.582435 4.472169 7.922483

O 3.479085 1.496274 8.302323

O 1.057023 0.137366 9.159658

O 1.055939 2.845470 9.140741

Mg 2.543480 7.474000 0.388370

Mg 0.008060 11.914340 2.261970

Mg 0.008060 9.012860 2.261970

Mg 2.366762 10.463215 4.539236

Mg 4.712526 7.480784 5.174691

Mg 2.374642 6.018798 7.431040

Mg 2.393012 8.919731 7.459981

Mg 0.183957 10.450598 9.290323

Si 2.727970 10.463600 1.221250

Si 2.018610 7.474000 3.313620

Si 4.404890 10.452147 6.437581

Si 0.349272 7.468845 8.473649

O 3.434710 11.826320 0.556660

| | | | |
|---|---|---|---|
| O | 3.434710 | 9.100870 | 0.556660 |
| O | 1.102310 | 10.463600 | 1.386100 |
| O | 1.223890 | 7.474000 | 1.760420 |
| O | 3.466830 | 10.463600 | 2.799080 |
| O | 3.663830 | 7.474000 | 3.191290 |
| O | 1.257330 | 6.146160 | 4.002710 |
| O | 1.257330 | 8.801840 | 4.002710 |
| O | 3.636436 | 11.766002 | 5.726976 |
| O | 3.631899 | 9.141520 | 5.725584 |
| O | 1.272594 | 10.465494 | 6.557290 |
| O | 1.084028 | 7.476824 | 6.908414 |
| O | 3.621549 | 10.448703 | 7.986357 |
| O | 3.474126 | 7.454010 | 8.298856 |
| O | 1.045270 | 6.109940 | 9.140977 |
| O | 1.051101 | 8.814985 | 9.161257 |
| Mg | 2.543480 | 13.453200 | 0.388370 |
| Mg | 0.008060 | 17.893540 | 2.261970 |
| Mg | 0.008060 | 14.992060 | 2.261970 |
| Mg | 2.356607 | 16.440167 | 4.528406 |
| Mg | 4.713633 | 13.437187 | 5.171004 |
| Mg | 2.404929 | 11.992149 | 7.472276 |
| Mg | 2.401149 | 14.890059 | 7.456808 |
| Mg | 0.187149 | 16.438520 | 9.274944 |
| Si | 2.727970 | 16.442800 | 1.221250 |
| Si | 2.018610 | 13.453200 | 3.313620 |
| Si | 4.388070 | 16.433382 | 6.413559 |
| Si | 0.360472 | 13.443410 | 8.518256 |
| O | 3.434710 | 17.805520 | 0.556660 |
| O | 3.434710 | 15.080070 | 0.556660 |
| O | 1.102310 | 16.442800 | 1.386100 |
| O | 1.223890 | 13.453200 | 1.760420 |
| O | 3.466830 | 16.442800 | 2.799080 |
| O | 3.663830 | 13.453200 | 3.191290 |
| O | 1.257330 | 12.125360 | 4.002710 |
| O | 1.257330 | 14.781030 | 4.002710 |
| O | 3.624219 | 17.752275 | 5.711474 |
| O | 3.627800 | 15.114207 | 5.712017 |
| O | 1.265694 | 16.425517 | 6.550896 |
| O | 1.089587 | 13.441913 | 6.952451 |
| O | 3.605100 | 16.438766 | 7.959155 |
| O | 3.496954 | 13.444852 | 8.305661 |
| O | 1.061114 | 12.092122 | 9.216459 |
| O | 1.062680 | 14.793972 | 9.218788 |
| Mg | 7.292480 | 1.494800 | 0.388370 |
| Mg | 4.757060 | 5.935140 | 2.261970 |
| Mg | 4.757060 | 3.033660 | 2.261970 |
| Mg | 7.090845 | 4.478872 | 4.512203 |
| Mg | 9.468357 | 1.476668 | 5.180712 |
| Mg | 7.125112 | 0.017885 | 7.443518 |
| Mg | 7.130025 | 2.913034 | 7.429050 |
| Mg | 4.919151 | 4.460449 | 9.267930 |
| Si | 7.476970 | 4.484400 | 1.221250 |
| Si | 6.767610 | 1.494800 | 3.313620 |
| Si | 9.125710 | 4.467772 | 6.399478 |

Si 5.101977 1.477211 8.448348

O 8.183700 5.847120 0.556660

O 8.183700 3.121670 0.556660

O 5.851310 4.484400 1.386100

O 5.972890 1.494800 1.760420

O 8.215830 4.484400 2.799080

O 8.412830 1.494800 3.191290

O 6.006330 0.166960 4.002710

O 6.006330 2.822640 4.002710

O 8.389342 5.792492 5.698255

O 8.387626 3.149203 5.693469

O 6.016108 4.455556 6.494946

O 5.839506 1.484504 6.884209

O 8.323145 4.454587 7.935378

O 8.228553 1.473897 8.329205

O 5.799507 0.117108 9.101425

O 5.803783 2.830955 9.135049

Mg 7.292480 7.474000 0.388370

Mg 4.757060 11.914340 2.261970

Mg 4.757060 9.012860 2.261970

Mg 7.107926 10.461855 4.542806

Mg 9.440009 7.479780 5.197265

Mg 7.084693 5.979956 7.404582

Mg 7.166535 8.923011 7.458399

Mg 4.937148 10.462989 9.413602

Si 7.476970 10.463600 1.221250

Si 6.767610 7.474000 3.313620

Si 9.165067 10.439854 6.420613

Si 5.097971 7.457927 8.434518

O 8.183700 11.826320 0.556660

O 8.183700 9.100870 0.556660

O 5.851310 10.463600 1.386100

O 5.972890 7.474000 1.760420

O 8.215830 10.463600 2.799080

O 8.412830 7.474000 3.191290

O 6.006330 6.146160 4.002710

O 6.006330 8.801840 4.002710

O 8.411249 11.755681 5.685925

O 8.394175 9.125021 5.704433

O 6.049609 10.438022 6.505417

O 5.834696 7.460936 6.875562

O 8.479253 10.450127 7.956833

O 8.160486 7.364556 8.296135

O 5.781740 6.095743 9.119016

O 5.803855 8.809182 9.104179

Mg 7.292480 13.453200 0.388370

Mg 4.757060 17.893540 2.261970

Mg 4.757060 14.992060 2.261970

Mg 7.095667 16.442271 4.526864

Mg 9.441969 13.442120 5.152546

Mg 7.160737 11.955555 7.445916

Mg 7.106070 14.907207 7.383391

Mg 4.895119 16.417309 9.393646

Si 7.476970 16.442800 1.221250

Si 6.767610 13.453200 3.313620

Si 9.143983 16.436551 6.404432

Si 5.129233 13.436691 8.402553

O 8.183700 17.805520 0.556660

O 8.183700 15.080070 0.556660

O 5.851310 16.442800 1.386100

O 5.972890 13.453200 1.760420

O 8.215830 16.442800 2.799080

O 8.412830 13.453200 3.191290

O 6.006330 12.125360 4.002710

O 6.006330 14.781030 4.002710

O 8.388290 17.761561 5.711962

O 8.390117 15.126161 5.683328

O 6.031809 16.446477 6.470332

O 5.853388 13.421394 6.838482

O 8.353997 16.420099 7.938590

O 8.197116 13.473128 8.253312

O 5.829656 12.078072 9.055779

O 5.817229 14.816723 9.036019

Mg 12.041480 1.494800 0.388370

Mg 9.506060 5.935140 2.261970

Mg 9.506060 3.033660 2.261970

Mg 11.838123 4.484449 4.527725

Mg 14.208710 1.505911 5.184177

Mg 11.862963 0.018650 7.450318

Mg 11.866521 2.936108 7.462374

Mg 9.640240 4.393009 9.298351

Si 12.225970 4.484400 1.221250

Si 11.516610 1.494800 3.313620

Si 13.855022 4.515120 6.426416

Si 9.851601 1.438841 8.478505

O 12.932710 5.847120 0.556660

O 12.932710 3.121670 0.556660

O 10.600310 4.484400 1.386100

O 10.721890 1.494800 1.760420

O 12.964830 4.484400 2.799080

O 13.161830 1.494800 3.191290

O 10.755330 0.166960 4.002710

O 10.755330 2.822640 4.002710

O 13.098826 5.850633 5.755785

O 13.121220 3.192939 5.714786

O 10.765117 4.505832 6.547344

O 10.587818 1.485867 6.910085

O 13.066598 4.496887 7.974487

O 12.976247 1.483401 8.313192

O 10.534512 0.062112 9.120375

O 10.555971 2.787117 9.173512

Mg 12.041480 7.474000 0.388370

Mg 9.506060 11.914340 2.261970

Mg 9.506060 9.012860 2.261970

Mg 11.833194 10.477394 4.498898

Mg 14.232240 7.464848 5.165953

Mg 11.837251 6.041630 7.534066

Mg 11.944959 8.931142 7.257466

Si 12.225114 10.459780 1.228047
Si 11.516610 7.474000 3.313620
Si 13.908945 10.487119 6.360093
Si 9.742632 7.396528 8.661211
O 12.928012 11.816614 0.557201
O 12.932710 9.100870 0.556660
O 10.600310 10.463600 1.386100
O 10.721890 7.474000 1.760420
O 12.964840 10.463600 2.799080
O 13.161830 7.474000 3.191290
O 10.755330 6.146160 4.002710
O 10.755330 8.801840 4.002710
O 13.148087 11.797615 5.671812
O 13.178707 9.152459 5.647057
O 10.825718 10.435594 6.418991
O 10.542411 7.589005 7.134080
O 13.087119 10.453515 7.892591
O 12.932174 7.550744 8.381791
O 10.537708 6.025029 9.198613
Mg 12.055464 13.452219 0.412127
Mg 9.503850 17.892430 2.259422
Mg 9.506060 14.992060 2.261970
Mg 11.833494 16.441035 4.527859
Mg 14.220251 13.467142 5.171539
Mg 11.847249 11.973963 7.375856
Mg 11.871410 14.900836 7.431454
Mg 9.577562 16.388736 9.418999

Si 12.223522 16.442324 1.231265
Si 11.509923 13.455493 3.312003
Si 13.882132 16.443914 6.394400
Si 9.803796 13.479441 8.371337
O 12.922021 17.801221 0.558133
O 12.928793 15.086396 0.559950
O 10.600310 16.442800 1.386100
O 10.721890 13.453200 1.760420
O 12.964840 16.442800 2.799080
O 13.161830 13.453200 3.191290
O 10.755330 12.125360 4.002710
O 10.755330 14.781030 4.002710
O 13.135292 17.768318 5.705734
O 13.138260 15.120786 5.698545
O 10.789669 16.436314 6.480869
O 10.593330 13.460661 6.845706
O 13.090966 16.429447 7.938455
O 12.942044 13.410110 8.308478
O 10.392206 11.986753 8.973539
O 10.539358 14.772102 9.098258
Mg 16.803813 1.493837 0.410317
Mg 14.253686 5.933479 2.250627
Mg 14.255060 3.033660 2.261970
Mg 16.588769 4.483451 4.521599
Mg 18.963441 1.492379 5.181651
Mg 16.626604 0.045072 7.442430
Mg 16.620177 2.945299 7.445294

Mg 14.421607 4.477538 9.304563

Si 16.973446 4.484135 1.232051

Si 16.260627 1.492710 3.310550

Si 18.614619 4.491467 6.398810

Si 14.596711 1.491633 8.472073

O 17.673656 5.841770 0.560847

O 17.674477 3.128284 0.559424

O 15.349310 4.484400 1.386100

O 15.470890 1.494800 1.760420

O 17.713830 4.484400 2.799080

O 17.910830 1.494800 3.191290

O 15.504330 0.166960 4.002710

O 15.504330 2.822640 4.002710

O 17.863322 5.813636 5.705283

O 17.865571 3.168400 5.702897

O 15.498547 4.504731 6.541010

O 15.334200 1.497683 6.905809

O 17.822111 4.490424 7.945285

O 17.730420 1.493820 8.308942

O 15.303315 0.138615 9.144327

O 15.297568 2.848852 9.145665

Mg 16.802967 7.475187 0.407400

Mg 14.253332 11.913712 2.256803

Mg 14.255060 9.012860 2.261970

Mg 16.601737 10.468329 4.525256

Mg 18.966449 7.478742 5.183374

Mg 16.607716 6.033871 7.455233

Mg 16.621039 8.929394 7.457270

Mg 14.378459 10.469747 9.284870

Si 16.973392 10.464154 1.232024

Si 16.262799 7.475670 3.306687

Si 18.630311 10.466858 6.411164

Si 14.575621 7.499051 8.502080

O 17.674387 11.819600 0.559516

O 17.674156 9.107930 0.559774

O 15.349310 10.463600 1.386100

O 15.470890 7.474000 1.760420

O 17.713840 10.463600 2.799080

O 17.910830 7.474000 3.191290

O 15.504330 6.146160 4.002710

O 15.504330 8.801840 4.002710

O 17.884571 11.790908 5.715793

O 17.878978 9.146268 5.713145

O 15.542918 10.472834 6.511929

O 15.300227 7.486671 6.933327

O 17.833595 10.467406 7.955472

O 17.720168 7.474921 8.314274

O 15.254971 6.133862 9.170001

O 15.287001 8.849860 9.167719

Mg 16.804095 13.453798 0.410163

Mg 14.252507 17.892375 2.256505

Mg 14.255060 14.992060 2.261970

Mg 16.591734 16.441009 4.524531

Mg 18.977406 13.449562 5.197782

| | | | |
|---|---|---|---|
| Mg | 16.628094 | 12.009834 | 7.465375 |
| Mg | 16.617453 | 14.892594 | 7.460371 |
| Mg | 14.424378 | 16.445451 | 9.283228 |
| Si | 16.973405 | 16.442872 | 1.232066 |
| Si | 16.261006 | 13.453350 | 3.309972 |
| Si | 18.623698 | 16.430102 | 6.403571 |
| Si | 14.566951 | 13.439825 | 8.473926 |
| O | 17.675522 | 17.798210 | 0.559146 |
| O | 17.675092 | 15.087066 | 0.559776 |
| O | 15.349310 | 16.442800 | 1.386100 |
| O | 15.470890 | 13.453200 | 1.760420 |
| O | 17.713840 | 16.442800 | 2.799080 |
| O | 17.910830 | 13.453200 | 3.191290 |
| O | 15.504330 | 12.125360 | 4.002710 |
| O | 15.504330 | 14.781030 | 4.002710 |
| O | 17.879862 | 17.754364 | 5.705171 |
| O | 17.875346 | 15.106631 | 5.711470 |
| O | 15.524978 | 16.438356 | 6.525135 |
| O | 15.325091 | 13.446838 | 6.917377 |
| O | 17.829195 | 16.433927 | 7.948576 |
| O | 17.733662 | 13.452939 | 8.345455 |
| O | 15.275693 | 12.089672 | 9.151704 |
| O | 15.264675 | 14.794136 | 9.154499 |
| C | 11.072544 | 9.277046 | 10.607406 |
| C | 9.897548 | 8.682788 | 10.053167 |
| C | 8.657605 | 9.108789 | 10.574216 |
| C | 8.543567 | 10.265263 | 11.395401 |
| C | 9.720721 | 11.032692 | 11.686395 |
| C | 10.986138 | 10.429381 | 11.401888 |
| C | 9.570010 | 12.444374 | 12.029144 |
| C | 10.696141 | 13.310557 | 12.195967 |
| C | 10.560596 | 14.695073 | 12.201059 |
| C | 9.277117 | 15.307158 | 12.047777 |
| C | 9.115911 | 16.729024 | 11.845696 |
| C | 7.844635 | 17.293781 | 11.699149 |
| C | 6.668475 | 16.479571 | 11.829924 |
| C | 5.351890 | 17.059002 | 11.800197 |
| C | 4.210245 | 16.247875 | 11.811237 |
| C | 4.320959 | 14.811451 | 11.862770 |
| C | 3.169482 | 13.967563 | 11.733239 |
| C | 3.301544 | 12.586267 | 11.772024 |
| C | 4.592030 | 11.970996 | 11.905674 |
| C | 4.767614 | 10.543642 | 11.880486 |
| C | 6.051338 | 9.978863 | 11.776873 |
| C | 7.223900 | 10.799214 | 11.727565 |
| C | 7.080385 | 12.203944 | 11.934039 |
| C | 5.769960 | 12.796243 | 11.977327 |
| C | 5.628232 | 14.222928 | 11.972953 |
| C | 6.802737 | 15.055978 | 11.960028 |
| C | 8.112240 | 14.462025 | 12.023650 |
| C | 8.261472 | 13.033406 | 12.012908 |
| H | 12.050208 | 8.837377 | 10.360456 |
| H | 9.668867 | 11.301077 | 8.792145 |
| H | 7.741110 | 8.620615 | 10.218616 |

H 11.907713 10.914614 11.744573

H 11.701440 12.884587 12.260215

H 11.449053 15.331808 12.282359

H 10.002844 17.379813 11.824292

H 7.724330 18.362512 11.483966

H 5.257896 18.148570 11.749880

H 3.213590 16.709505 11.807392

H 2.190243 14.417411 11.529473

H 2.412703 11.962545 11.618191

H 3.888340 9.890949 11.975957

H 6.156666 8.891420 11.717067

**Fullerene [010]-fo**

Number of atoms: 396

Mg 2.543480 1.494800 0.388370

Mg 0.008060 5.935140 2.261970

Mg 0.008060 3.033660 2.261970

Mg 2.345523 4.483200 4.520274

Mg 4.723886 1.493349 5.182685

Mg 2.387721 0.046267 7.441655

Mg 2.387926 2.943896 7.441769

Mg 0.186226 4.482952 9.280512

Si 2.727970 4.484400 1.221250

Si 2.018610 1.494800 3.313620

Si 4.386657 4.483420 6.393490

Si 0.361860 1.495291 8.468204

O 3.434710 5.847120 0.556660

O 3.434700 3.121670 0.556660

O 1.102310 4.484400 1.386100

O 1.223890 1.494800 1.760420

O 3.466830 4.484400 2.799080

O 3.663830 1.494800 3.191290

O 1.257330 0.166960 4.002710

O 1.257330 2.822640 4.002710

O 3.634143 5.804621 5.699625

O 3.635525 3.162562 5.700480

O 1.278908 4.483050 6.516511

O 1.098823 1.495914 6.902055

O 3.599685 4.487633 7.941454

O 3.489411 1.496587 8.310914

O 1.063622 0.140889 9.142176

O 1.063048 2.851407 9.140956

Mg 2.543480 7.474000 0.388370

Mg 0.008060 11.914340 2.261970

Mg 0.008060 9.012860 2.261970

Mg 2.346474 10.464329 4.523548

Mg 4.713270 7.474069 5.170357

Mg 2.381270 6.021209 7.439154

Mg 2.383029 8.921966 7.442498

Mg 0.188603 10.461164 9.282086

Si 2.727970 10.463600 1.221250

Si 2.018610 7.474000 3.313620

Si 4.387305 10.462220 6.401577

Si 0.359752 7.472109 8.470923

O 3.434710 11.826320 0.556660
O 3.434710 9.100870 0.556660
O 1.102310 10.463600 1.386100
O 1.223890 7.474000 1.760420
O 3.466830 10.463600 2.799080
O 3.663830 7.474000 3.191290
O 1.257330 6.146160 4.002710
O 1.257330 8.801840 4.002710
O 3.635413 11.783186 5.707397
O 3.633638 9.141392 5.706411
O 1.278366 10.463139 6.522354
O 1.096293 7.472337 6.903464
O 3.601080 10.457337 7.950142
O 3.482260 7.469955 8.316704
O 1.061083 6.116318 9.143592
O 1.062891 8.826435 9.145282
Mg 2.543480 13.453200 0.388370
Mg 0.008060 17.893540 2.261970
Mg 0.008060 14.992060 2.261970
Mg 2.346428 16.442346 4.522386
Mg 4.725032 13.450324 5.185108
Mg 2.387970 12.002005 7.447528
Mg 2.387252 14.898826 7.443683
Mg 0.191412 16.443390 9.279882
Si 2.727970 16.442800 1.221250
Si 2.018610 13.453200 3.313620
Si 4.387503 16.440094 6.396385
Si 0.361740 13.452031 8.473523
O 3.434710 17.805520 0.556660
O 3.434710 15.080070 0.556660
O 1.102310 16.442800 1.386100
O 1.223890 13.453200 1.760420
O 3.466830 16.442800 2.799080
O 3.663830 13.453200 3.191290
O 1.257330 12.125360 4.002710
O 1.257330 14.781030 4.002710
O 3.635813 17.762047 5.702239
O 3.636122 15.117607 5.703140
O 1.276508 16.441007 6.520315
O 1.097547 13.450329 6.906259
O 3.594948 16.440700 7.941347
O 3.488579 13.449269 8.315951
O 1.062621 12.095261 9.145960
O 1.063451 14.808504 9.144730
Mg 7.292480 1.494800 0.388370
Mg 4.757060 5.935140 2.261970
Mg 4.757060 3.033660 2.261970
Mg 7.105193 4.479862 4.525714
Mg 9.473260 1.496707 5.189709
Mg 7.137663 0.045114 7.446765
Mg 7.144962 2.941444 7.449688
Mg 4.943403 4.480549 9.284922
Si 7.476970 4.484400 1.221250
Si 6.767610 1.494800 3.313620

| | | | |
|---|---|---|---|
| Si | 9.146481 | 4.478533 | 6.409852 |
| Si | 5.110685 | 1.494225 | 8.475159 |
| O | 8.183700 | 5.847120 | 0.556660 |
| O | 8.183700 | 3.121670 | 0.556660 |
| O | 5.851310 | 4.484400 | 1.386100 |
| O | 5.972890 | 1.494800 | 1.760420 |
| O | 8.215830 | 4.484400 | 2.799080 |
| O | 8.412830 | 1.494800 | 3.191290 |
| O | 6.006330 | 0.166960 | 4.002710 |
| O | 6.006330 | 2.822640 | 4.002710 |
| O | 8.385727 | 5.790837 | 5.695786 |
| O | 8.384228 | 3.158551 | 5.714625 |
| O | 6.027327 | 4.482441 | 6.523538 |
| O | 5.845757 | 1.495866 | 6.908631 |
| O | 8.354885 | 4.487552 | 7.952818 |
| O | 8.233099 | 1.489130 | 8.322649 |
| O | 5.807683 | 0.138144 | 9.148759 |
| O | 5.813178 | 2.847692 | 9.152334 |
| Mg | 7.292480 | 7.474000 | 0.388370 |
| Mg | 4.757060 | 11.914340 | 2.261970 |
| Mg | 4.757060 | 9.012860 | 2.261970 |
| Mg | 7.105053 | 10.467567 | 4.527067 |
| Mg | 9.458861 | 7.470852 | 5.155076 |
| Mg | 7.137625 | 6.016711 | 7.417290 |
| Mg | 7.139773 | 8.925952 | 7.413747 |
| Mg | 4.935142 | 10.465857 | 9.307641 |
| Si | 7.476970 | 10.463600 | 1.221250 |

| | | | |
|---|---|---|---|
| Si | 6.767610 | 7.474000 | 3.313620 |
| Si | 9.146543 | 10.463918 | 6.410561 |
| Si | 5.101703 | 7.467897 | 8.460580 |
| O | 8.183700 | 11.826320 | 0.556660 |
| O | 8.183700 | 9.100870 | 0.556660 |
| O | 5.851310 | 10.463600 | 1.386100 |
| O | 5.972890 | 7.474000 | 1.760420 |
| O | 8.215830 | 10.463600 | 2.799080 |
| O | 8.412830 | 7.474000 | 3.191290 |
| O | 6.006330 | 6.146160 | 4.002710 |
| O | 6.006330 | 8.801840 | 4.002710 |
| O | 8.384289 | 11.784659 | 5.713764 |
| O | 8.386685 | 9.147714 | 5.699854 |
| O | 6.028396 | 10.462130 | 6.522279 |
| O | 5.834128 | 7.471807 | 6.901538 |
| O | 8.355785 | 10.457345 | 7.952021 |
| O | 8.246464 | 7.465995 | 8.240334 |
| O | 5.816391 | 6.122869 | 9.152815 |
| O | 5.809688 | 8.822186 | 9.151003 |
| Mg | 7.292480 | 13.453200 | 0.388370 |
| Mg | 4.757060 | 17.893540 | 2.261970 |
| Mg | 4.757060 | 14.992060 | 2.261970 |
| Mg | 7.097288 | 16.441961 | 4.523349 |
| Mg | 9.471362 | 13.447000 | 5.186138 |
| Mg | 7.145344 | 12.000515 | 7.452051 |
| Mg | 7.137865 | 14.897830 | 7.447571 |
| Mg | 4.936605 | 16.441088 | 9.284676 |

| | | | |
|---|---|---|---|
| Si | 7.476970 | 16.442800 | 1.221250 |
| Si | 6.767610 | 13.453200 | 3.313620 |
| Si | 9.134454 | 16.440486 | 6.396929 |
| Si | 5.110305 | 13.449717 | 8.478715 |
| O | 8.183700 | 17.805520 | 0.556660 |
| O | 8.183700 | 15.080070 | 0.556660 |
| O | 5.851310 | 16.442800 | 1.386100 |
| O | 5.972890 | 13.453200 | 1.760420 |
| O | 8.215830 | 16.442800 | 2.799080 |
| O | 8.412830 | 13.453200 | 3.191290 |
| O | 6.006330 | 12.125360 | 4.002710 |
| O | 6.006330 | 14.781030 | 4.002710 |
| O | 8.384208 | 17.762806 | 5.702108 |
| O | 8.383718 | 15.117982 | 5.703423 |
| O | 6.029153 | 16.439705 | 6.522647 |
| O | 5.845422 | 13.445919 | 6.912512 |
| O | 8.346578 | 16.441225 | 7.943489 |
| O | 8.235116 | 13.455760 | 8.319498 |
| O | 5.811137 | 12.094867 | 9.156868 |
| O | 5.808352 | 14.807236 | 9.149699 |
| Mg | 12.041480 | 1.494800 | 0.388370 |
| Mg | 9.506060 | 5.935140 | 2.261970 |
| Mg | 9.506060 | 3.033660 | 2.261970 |
| Mg | 11.839825 | 4.482552 | 4.522561 |
| Mg | 14.215080 | 1.492316 | 5.180032 |
| Mg | 11.879848 | 0.054426 | 7.450026 |
| Mg | 11.868667 | 2.959619 | 7.454659 |
| Mg | 9.616495 | 4.513062 | 9.402584 |
| Si | 12.225970 | 4.484400 | 1.221250 |
| Si | 11.516610 | 1.494800 | 3.313620 |
| Si | 13.889308 | 4.474413 | 6.386649 |
| Si | 9.853867 | 1.510399 | 8.491757 |
| O | 12.932710 | 5.847120 | 0.556660 |
| O | 12.932710 | 3.121670 | 0.556660 |
| O | 10.600310 | 4.484400 | 1.386100 |
| O | 10.721890 | 1.494800 | 1.760420 |
| O | 12.964830 | 4.484400 | 2.799080 |
| O | 13.161830 | 1.494800 | 3.191290 |
| O | 10.755330 | 0.166960 | 4.002710 |
| O | 10.755330 | 2.822640 | 4.002710 |
| O | 13.144249 | 5.796769 | 5.688339 |
| O | 13.134480 | 3.154639 | 5.698904 |
| O | 10.789020 | 4.477034 | 6.484023 |
| O | 10.589380 | 1.502643 | 6.921416 |
| O | 13.100429 | 4.483974 | 7.931002 |
| O | 12.979613 | 1.505900 | 8.319954 |
| O | 10.559192 | 0.152283 | 9.161011 |
| O | 10.525950 | 2.885779 | 9.152410 |
| Mg | 12.041480 | 7.474000 | 0.388370 |
| Mg | 9.506060 | 11.914340 | 2.261970 |
| Mg | 9.506060 | 9.012860 | 2.261970 |
| Mg | 11.838380 | 10.465406 | 4.523183 |
| Mg | 14.218570 | 7.473412 | 5.176927 |
| Mg | 11.889935 | 6.015055 | 7.411496 |

Mg 11.884493 8.932607 7.409775

Mg 9.623160 10.449669 9.404898

Si 12.225970 10.463600 1.221250

Si 11.516610 7.474000 3.313620

Si 13.888543 10.471651 6.387079

Si 9.872430 7.477007 8.372548

O 12.932710 11.826320 0.556660

O 12.932710 9.100870 0.556660

O 10.600310 10.463600 1.386100

O 10.721890 7.474000 1.760420

O 12.964840 10.463600 2.799080

O 13.161830 7.474000 3.191290

O 10.755330 6.146160 4.002710

O 10.755330 8.801840 4.002710

O 13.135820 11.792606 5.698659

O 13.144490 9.148005 5.688715

O 10.789440 10.469855 6.474950

O 10.616951 7.466214 6.828616

O 13.098232 10.463203 7.930323

O 12.981975 7.473256 8.293479

O 10.565215 6.108437 9.047944

O 10.554716 8.848314 9.027090

Mg 12.041480 13.453200 0.388370

Mg 9.506060 17.893540 2.261970

Mg 9.506060 14.992060 2.261970

Mg 11.842938 16.442508 4.523313

Mg 14.216271 13.455146 5.181425

Mg 11.867887 11.990416 7.449246

Mg 11.879584 14.896262 7.446301

Mg 9.697266 16.445360 9.279065

Si 12.225970 16.442800 1.221250

Si 11.516610 13.453200 3.313620

Si 13.879205 16.443346 6.399277

Si 9.856661 13.441503 8.480317

O 12.932710 17.805520 0.556660

O 12.932710 15.080070 0.556660

O 10.600310 16.442800 1.386100

O 10.721890 13.453200 1.760420

O 12.964840 16.442800 2.799080

O 13.161830 13.453200 3.191290

O 10.755330 12.125360 4.002710

O 10.755330 14.781030 4.002710

O 13.129001 17.764766 5.704923

O 13.130243 15.121347 5.704077

O 10.775078 16.444838 6.525722

O 10.590301 13.451249 6.909610

O 13.087686 16.443487 7.944458

O 12.980047 13.445430 8.313626

O 10.537777 12.069863 9.133730

O 10.558824 14.802084 9.149740

Mg 16.790480 1.494800 0.388370

Mg 14.255060 5.935140 2.261970

Mg 14.255060 3.033660 2.261970

Mg 16.591255 4.484462 4.521401

Mg 18.967574 1.493137 5.179047

Mg 16.628330 0.045501 7.443610

Mg 16.631293 2.942110 7.439370

Mg 14.431636 4.485354 9.280618

Si 16.974970 4.484400 1.221250

Si 16.265610 1.494800 3.313620

Si 18.633141 4.483110 6.390729

Si 14.601584 1.499152 8.473789

O 17.681710 5.847120 0.556660

O 17.681710 3.121670 0.556660

O 15.349310 4.484400 1.386100

O 15.470890 1.494800 1.760420

O 17.713830 4.484400 2.799080

O 17.910830 1.494800 3.191290

O 15.504330 0.166960 4.002710

O 15.504330 2.822640 4.002710

O 17.882964 5.804597 5.696438

O 17.882946 3.161746 5.697003

O 15.531988 4.479895 6.513785

O 15.337376 1.494441 6.906147

O 17.843254 4.483588 7.936589

O 17.737458 1.494698 8.306124

O 15.303091 0.142119 9.144675

O 15.309910 2.852955 9.143678

Mg 16.790480 7.474000 0.388370

Mg 14.255060 11.914340 2.261970

Mg 14.255060 9.012860 2.261970

Mg 16.591503 10.464012 4.523041

Mg 18.965758 7.474219 5.178447

Mg 16.630983 6.023994 7.439178

Mg 16.629870 8.922991 7.440786

Mg 14.429149 10.461585 9.279547

Si 16.974970 10.463600 1.221250

Si 16.265610 7.474000 3.313620

Si 18.632702 10.465188 6.395287

Si 14.600840 7.473065 8.465014

O 17.681710 11.826320 0.556660

O 17.681710 9.100870 0.556660

O 15.349310 10.463600 1.386100

O 15.470890 7.474000 1.760420

O 17.713840 10.463600 2.799080

O 17.910830 7.474000 3.191290

O 15.504330 6.146160 4.002710

O 15.504330 8.801840 4.002710

O 17.883536 11.788570 5.703926

O 17.883588 9.143159 5.700197

O 15.531523 10.467727 6.515262

O 15.344059 7.472026 6.901076

O 17.842575 10.462938 7.941236

O 17.735848 7.473170 8.308761

O 15.304284 6.119627 9.142404

O 15.302874 8.826850 9.142198

Mg 16.790480 13.453200 0.388370

Mg 14.255060 17.893540 2.261970

Mg 14.255060 14.992060 2.261970
Mg 16.591651 16.441858 4.522340
Mg 18.968218 13.453311 5.181118
Mg 16.631342 12.005503 7.444443
Mg 16.629220 14.900741 7.445690
Mg 14.432678 16.443477 9.283950
Si 16.974970 16.442800 1.221250
Si 16.265610 13.453200 3.313620
Si 18.631000 16.440759 6.395597
Si 14.600729 13.450280 8.471718
O 17.681710 17.805520 0.556660
O 17.681710 15.080070 0.556660
O 15.349310 16.442800 1.386100
O 15.470890 13.453200 1.760420
O 17.713840 16.442800 2.799080
O 17.910830 13.453200 3.191290
O 15.504330 12.125360 4.002710
O 15.504330 14.781030 4.002710
O 17.880906 17.762789 5.701050
O 17.880016 15.117724 5.704030
O 15.522435 16.442247 6.522262
O 15.340120 13.453085 6.905839
O 17.840883 16.441989 7.941980
O 17.737190 13.453154 8.313109
O 15.305873 12.095334 9.144233
O 15.302851 14.807815 9.142852
C 10.013985 9.746091 12.063324
C 10.892584 8.579974 12.004731
C 10.379087 7.342873 11.561990
C 8.989086 7.230947 11.147051
C 8.140096 8.350035 11.187827
C 8.657015 9.647379 11.635793
C 7.595865 10.294221 12.421596
C 7.930503 11.022566 13.583583
C 9.326768 11.131299 14.009275
C 9.360849 11.084490 15.470656
C 10.412928 10.412823 16.130832
C 11.471711 9.766794 15.355191
C 11.441110 9.816171 13.944499
C 10.353022 10.514906 13.261133
C 11.773247 8.622417 13.166848
C 12.127127 7.422751 13.824984
C 11.611013 6.140713 13.343134
C 10.757969 6.100094 12.219794
C 9.595008 5.204047 12.203179
C 8.496410 5.903295 11.518942
C 7.165055 5.775794 11.991090
C 6.286179 6.945463 12.047493
C 6.763539 8.213562 11.644127
C 6.427204 9.411738 12.415818
C 5.633088 9.292746 13.581383
C 5.975064 10.053449 14.783026
C 7.104532 10.900945 14.784107
C 7.987460 10.941350 15.949183

C 7.712516 10.131720 17.072725

C 8.801506 9.436332 17.756625

C 10.128641 9.574872 17.294410

C 11.008452 8.408372 17.235564

C 11.838385 8.527396 16.037313

C 12.158294 7.375091 15.284337

C 11.664217 6.064651 15.706044

C 11.324364 5.301260 14.505887

C 10.198559 4.447400 14.502364

C 9.315373 4.399279 13.336552

C 7.942254 4.266539 13.815270

C 6.886637 4.938517 13.154836

C 5.832361 5.589590 13.933675

C 5.867557 5.545595 15.343146

C 5.531609 6.735660 16.122613

C 5.178251 7.933107 15.461933

C 5.139556 7.982266 14.001702

C 5.467910 6.830713 13.249436

C 5.691588 9.213609 15.946148

C 6.544778 9.251560 17.070606

C 6.912305 8.012207 17.753363

C 6.414588 6.775765 17.286774

C 7.294650 5.609408 17.228831

C 6.957508 4.848540 16.027136

C 7.975364 4.220115 15.277985

C 9.369651 4.332693 15.701795

C 9.696684 5.070660 16.860709

C 8.641890 5.719190 17.637837

C 9.156785 6.999775 18.120977

C 8.307097 8.125973 18.177249

C 10.530696 7.143300 17.642069

C 10.864030 5.951296 16.863313

**Fullerene Fe-[010]-fo**

Number of atoms: 396

Mg 2.543480 1.494800 0.388370

Mg 0.008060 5.935140 2.261970

Mg 0.008060 3.033660 2.261970

Mg 2.344001 4.485905 4.522125

Mg 4.718628 1.497223 5.181856

Mg 2.381869 0.047615 7.440785

Mg 2.381429 2.948519 7.439553

Mg 0.181010 4.487023 9.289290

Si 2.727970 4.484400 1.221250

Si 2.018610 1.494800 3.313620

Si 4.382835 4.488796 6.395110

Si 0.358364 1.498841 8.467587

O 3.434710 5.847120 0.556660

O 3.434700 3.121670 0.556660

O 1.102310 4.484400 1.386100

O 1.223890 1.494800 1.760420

O 3.466830 4.484400 2.799080

O 3.663830 1.494800 3.191290

O 1.257330 0.166960 4.002710

| | | | |
|---|---|---|---|
| O | 1.257330 | 2.822640 | 4.002710 |
| O | 3.630648 | 5.809666 | 5.700717 |
| O | 3.632066 | 3.167832 | 5.698545 |
| O | 1.272952 | 4.490710 | 6.519545 |
| O | 1.094424 | 1.498902 | 6.900703 |
| O | 3.592687 | 4.488477 | 7.939710 |
| O | 3.483833 | 1.498046 | 8.305725 |
| O | 1.058320 | 0.142221 | 9.138156 |
| O | 1.058377 | 2.855401 | 9.138405 |
| Mg | 2.543480 | 7.474000 | 0.388370 |
| Mg | 0.008060 | 11.914340 | 2.261970 |
| Mg | 0.008060 | 9.012860 | 2.261970 |
| Mg | 2.347133 | 10.464607 | 4.530544 |
| Mg | 4.714140 | 7.477363 | 5.180711 |
| Mg | 2.379141 | 6.029837 | 7.446428 |
| Mg | 2.385426 | 8.928652 | 7.460019 |
| Mg | 0.182290 | 10.466817 | 9.300125 |
| Si | 2.727970 | 10.463600 | 1.221250 |
| Si | 2.018610 | 7.474000 | 3.313620 |
| Si | 4.388773 | 10.467206 | 6.413843 |
| Si | 0.353615 | 7.481123 | 8.478413 |
| O | 3.434710 | 11.826320 | 0.556660 |
| O | 3.434710 | 9.100870 | 0.556660 |
| O | 1.102310 | 10.463600 | 1.386100 |
| O | 1.223890 | 7.474000 | 1.760420 |
| O | 3.466830 | 10.463600 | 2.799080 |
| O | 3.663830 | 7.474000 | 3.191290 |
| O | 1.257330 | 6.146160 | 4.002710 |
| O | 1.257330 | 8.801840 | 4.002710 |
| O | 3.637907 | 11.788085 | 5.716018 |
| O | 3.636410 | 9.147636 | 5.715813 |
| O | 1.281374 | 10.466816 | 6.528354 |
| O | 1.092407 | 7.481357 | 6.911539 |
| O | 3.610852 | 10.466985 | 7.963908 |
| O | 3.483427 | 7.473774 | 8.322177 |
| O | 1.052169 | 6.121685 | 9.146167 |
| O | 1.058484 | 8.834116 | 9.153040 |
| Mg | 2.543480 | 13.453200 | 0.388370 |
| Mg | 0.008060 | 17.893540 | 2.261970 |
| Mg | 0.008060 | 14.992060 | 2.261970 |
| Mg | 2.344904 | 16.442465 | 4.524058 |
| Mg | 4.718999 | 13.453847 | 5.185641 |
| Mg | 2.387533 | 12.003989 | 7.460265 |
| Mg | 2.384714 | 14.901095 | 7.449546 |
| Mg | 0.184768 | 16.446436 | 9.287348 |
| Si | 2.727970 | 16.442800 | 1.221250 |
| Si | 2.018610 | 13.453200 | 3.313620 |
| Si | 4.385535 | 16.443573 | 6.399036 |
| Si | 0.357125 | 13.453001 | 8.478175 |
| O | 3.434710 | 17.805520 | 0.556660 |
| O | 3.434710 | 15.080070 | 0.556660 |
| O | 1.102310 | 16.442800 | 1.386100 |
| O | 1.223890 | 13.453200 | 1.760420 |
| O | 3.466830 | 16.442800 | 2.799080 |

| | | | |
|---|---|---|---|
| O | 3.663830 | 13.453200 | 3.191290 |
| O | 1.257330 | 12.125360 | 4.002710 |
| O | 1.257330 | 14.781030 | 4.002710 |
| O | 3.634232 | 17.764122 | 5.702677 |
| O | 3.634762 | 15.120591 | 5.705537 |
| O | 1.275818 | 16.441350 | 6.519368 |
| O | 1.096729 | 13.451788 | 6.912162 |
| O | 3.594452 | 16.443664 | 7.943513 |
| O | 3.490365 | 13.455145 | 8.326847 |
| O | 1.060452 | 12.098538 | 9.151795 |
| O | 1.057476 | 14.812307 | 9.145338 |
| Mg | 7.292480 | 1.494800 | 0.388370 |
| Mg | 4.757060 | 5.935140 | 2.261970 |
| Mg | 4.757060 | 3.033660 | 2.261970 |
| Mg | 7.092654 | 4.482883 | 4.521831 |
| Mg | 9.468879 | 1.494302 | 5.181615 |
| Mg | 7.134552 | 0.044673 | 7.448279 |
| Mg | 7.133357 | 2.944224 | 7.444817 |
| Mg | 4.930080 | 4.484827 | 9.289070 |
| Si | 7.476970 | 4.484400 | 1.221250 |
| Si | 6.767610 | 1.494800 | 3.313620 |
| Si | 9.132311 | 4.483115 | 6.390305 |
| Si | 5.104571 | 1.496518 | 8.469258 |
| O | 8.183700 | 5.847120 | 0.556660 |
| O | 8.183700 | 3.121670 | 0.556660 |
| O | 5.851310 | 4.484400 | 1.386100 |
| O | 5.972890 | 1.494800 | 1.760420 |
| O | 8.215830 | 4.484400 | 2.799080 |
| O | 8.412830 | 1.494800 | 3.191290 |
| O | 6.006330 | 0.166960 | 4.002710 |
| O | 6.006330 | 2.822640 | 4.002710 |
| O | 8.376413 | 5.799877 | 5.691628 |
| O | 8.380638 | 3.160471 | 5.699606 |
| O | 6.025157 | 4.483654 | 6.516383 |
| O | 5.843251 | 1.494563 | 6.903371 |
| O | 8.346555 | 4.489210 | 7.937649 |
| O | 8.236844 | 1.495087 | 8.309279 |
| O | 5.805275 | 0.140626 | 9.141654 |
| O | 5.805908 | 2.852746 | 9.139913 |
| Mg | 7.292480 | 7.474000 | 0.388370 |
| Mg | 4.757060 | 11.914340 | 2.261970 |
| Mg | 4.757060 | 9.012860 | 2.261970 |
| Mg | 7.094480 | 10.464214 | 4.525974 |
| Mg | 9.458224 | 7.468969 | 5.161051 |
| Mg | 7.123867 | 6.021162 | 7.434880 |
| Mg | 7.123776 | 8.920625 | 7.434106 |
| Mg | 4.951542 | 10.462457 | 9.326830 |
| Si | 7.476970 | 10.463600 | 1.221250 |
| Si | 6.767610 | 7.474000 | 3.313620 |
| Si | 9.154696 | 10.463320 | 6.389476 |
| Si | 5.105216 | 7.473181 | 8.475532 |
| O | 8.183700 | 11.826320 | 0.556660 |
| O | 8.183700 | 9.100870 | 0.556660 |
| O | 5.851310 | 10.463600 | 1.386100 |

O 5.972890 7.474000 1.760420

O 8.215830 10.463600 2.799080

O 8.412830 7.474000 3.191290

O 6.006330 6.146160 4.002710

O 6.006330 8.801840 4.002710

O 8.383271 11.786198 5.715726

O 8.382056 9.145071 5.711393

O 6.034172 10.466067 6.521464

O 5.833086 7.472566 6.903025

O 8.356002 10.462108 7.949059

O 8.230070 7.474212 8.284647

O 5.809066 6.115378 9.140041

O 5.815627 8.826336 9.142805

Mg 7.292480 13.453200 0.388370

Mg 4.757060 17.893540 2.261970

Mg 4.757060 14.992060 2.261970

Mg 7.093695 16.443812 4.523682

Mg 9.459413 13.455533 5.161362

Mg 7.127444 12.003127 7.440615

Mg 7.130856 14.902164 7.444457

Mg 4.933789 16.443523 9.291009

Si 7.476970 16.442800 1.221250

Si 6.767610 13.453200 3.313620

Si 9.134565 16.440815 6.395893

Si 5.111623 13.452614 8.485639

O 8.183700 17.805520 0.556660

O 8.183700 15.080070 0.556660

O 5.851310 16.442800 1.386100

O 5.972890 13.453200 1.760420

O 8.215830 16.442800 2.799080

O 8.412830 13.453200 3.191290

O 6.006330 12.125360 4.002710

O 6.006330 14.781030 4.002710

O 8.382893 17.762601 5.704141

O 8.379708 15.119508 5.702819

O 6.028466 16.441888 6.521022

O 5.841002 13.452643 6.912885

O 8.350780 16.435720 7.945352

O 8.245690 13.442572 8.299666

O 5.822105 12.098909 9.156041

O 5.815767 14.812793 9.147505

Mg 12.041480 1.494800 0.388370

Mg 9.506060 5.935140 2.261970

Mg 9.506060 3.033660 2.261970

Mg 11.843757 4.485382 4.519854

Mg 14.217893 1.498085 5.178850

Mg 11.882390 0.048842 7.441376

Mg 11.880346 2.946559 7.439697

Mg 9.693851 4.504555 9.278773

Si 12.225970 4.484400 1.221250

Si 11.516610 1.494800 3.313620

Si 13.883626 4.487106 6.392379

Si 9.856859 1.496266 8.473814

O 12.932710 5.847120 0.556660

O 12.932710 3.121670 0.556660

O 10.600310 4.484400 1.386100

O 10.721890 1.494800 1.760420

O 12.964830 4.484400 2.799080

O 13.161830 1.494800 3.191290

O 10.755330 0.166960 4.002710

O 10.755330 2.822640 4.002710

O 13.134137 5.807010 5.694403

O 13.132172 3.165749 5.697939

O 10.773565 4.483783 6.515844

O 10.592444 1.495945 6.905042

O 13.089714 4.488699 7.936503

O 12.981881 1.498596 8.308390

O 10.554933 0.134788 9.141706

O 10.553557 2.858664 9.140624

Mg 12.041480 7.474000 0.388370

Mg 9.506060 11.914340 2.261970

Mg 9.506060 9.012860 2.261970

Mg 11.849261 10.463192 4.512693

Mg 14.223639 7.477133 5.180041

Mg 11.886398 6.029040 7.426201

Mg 11.875229 8.936504 7.382632

Fe 9.576175 10.507912 9.478910

Si 12.225970 10.463600 1.221250

Si 11.516610 7.474000 3.313620

Si 13.901001 10.463556 6.392150

Si 9.849224 7.479352 8.414215

O 12.932710 11.826320 0.556660

O 12.932710 9.100870 0.556660

O 10.600310 10.463600 1.386100

O 10.721890 7.474000 1.760420

O 12.964840 10.463600 2.799080

O 13.161830 7.474000 3.191290

O 10.755330 6.146160 4.002710

O 10.755330 8.801840 4.002710

O 13.149917 11.784494 5.684491

O 13.146135 9.147174 5.684068

O 10.793493 10.469277 6.438844

O 10.593250 7.453699 6.856993

O 13.090768 10.466152 7.918911

O 12.962452 7.488452 8.291736

O 10.554653 6.151559 9.133861

O 10.541170 8.890889 8.992461

Mg 12.041480 13.453200 0.388370

Mg 9.506060 17.893540 2.261970

Mg 9.506060 14.992060 2.261970

Mg 11.845176 16.443623 4.520318

Mg 14.227247 13.454215 5.180285

Mg 11.893398 11.999624 7.369610

Mg 11.895174 14.906356 7.424771

Mg 9.697367 16.426710 9.289279

Si 12.225970 16.442800 1.221250

Si 11.516610 13.453200 3.313620

Si 13.886893 16.447758 6.393809

Si 9.864692 13.458211 8.417466

O 12.932710 17.805520 0.556660

O 12.932710 15.080070 0.556660

O 10.600310 16.442800 1.386100

O 10.721890 13.453200 1.760420

O 12.964840 16.442800 2.799080

O 13.161830 13.453200 3.191290

O 10.755330 12.125360 4.002710

O 10.755330 14.781030 4.002710

O 13.132644 17.767090 5.699699

O 13.137870 15.125230 5.696670

O 10.775942 16.443653 6.518569

O 10.599204 13.474864 6.862655

O 13.093751 16.445544 7.939104

O 12.968822 13.447801 8.291042

O 10.590002 12.049375 9.005494

O 10.566100 14.781940 9.140861

Mg 16.790480 1.494800 0.388370

Mg 14.255060 5.935140 2.261970

Mg 14.255060 3.033660 2.261970

Mg 16.593368 4.487444 4.523468

Mg 18.964615 1.499347 5.180325

Mg 16.631018 0.048341 7.441927

Mg 16.629153 2.949916 7.441702

Mg 14.430424 4.494293 9.282987

Si 16.974970 4.484400 1.221250

Si 16.265610 1.494800 3.313620

Si 18.627446 4.495753 6.398883

Si 14.603213 1.499747 8.469661

O 17.681710 5.847120 0.556660

O 17.681710 3.121670 0.556660

O 15.349310 4.484400 1.386100

O 15.470890 1.494800 1.760420

O 17.713830 4.484400 2.799080

O 17.910830 1.494800 3.191290

O 15.504330 0.166960 4.002710

O 15.504330 2.822640 4.002710

O 17.876649 5.817166 5.704980

O 17.879001 3.173789 5.700665

O 15.524515 4.492426 6.521146

O 15.339765 1.499048 6.902209

O 17.835413 4.494115 7.944370

O 17.733912 1.499818 8.305010

O 15.304165 0.142490 9.139332

O 15.302846 2.858105 9.138582

Mg 16.790480 7.474000 0.388370

Mg 14.255060 11.914340 2.261970

Mg 14.255060 9.012860 2.261970

Mg 16.594914 10.464783 4.529355

Mg 18.966650 7.482003 5.185761

Mg 16.622685 6.040777 7.451777

Mg 16.618197 8.941088 7.461104

Mg 14.328019 10.474846 9.367242

Si 16.974970 10.463600 1.221250

| | | | |
|---|---|---|---|
| Si | 16.265610 | 7.474000 | 3.313620 |
| Si | 18.634124 | 10.469009 | 6.403443 |
| Si | 14.582458 | 7.496682 | 8.473600 |
| O | 17.681710 | 11.826320 | 0.556660 |
| O | 17.681710 | 9.100870 | 0.556660 |
| O | 15.349310 | 10.463600 | 1.386100 |
| O | 15.470890 | 7.474000 | 1.760420 |
| O | 17.713840 | 10.463600 | 2.799080 |
| O | 17.910830 | 7.474000 | 3.191290 |
| O | 15.504330 | 6.146160 | 4.002710 |
| O | 15.504330 | 8.801840 | 4.002710 |
| O | 17.885207 | 11.791435 | 5.708824 |
| O | 17.884450 | 9.147616 | 5.708633 |
| O | 15.541727 | 10.467756 | 6.497530 |
| O | 15.334815 | 7.489197 | 6.912327 |
| O | 17.844536 | 10.468658 | 7.949217 |
| O | 17.727659 | 7.487468 | 8.321998 |
| O | 15.284968 | 6.138657 | 9.145937 |
| O | 15.258474 | 8.863612 | 9.146562 |
| Mg | 16.790480 | 13.453200 | 0.388370 |
| Mg | 14.255060 | 17.893540 | 2.261970 |
| Mg | 14.255060 | 14.992060 | 2.261970 |
| Mg | 16.594321 | 16.441613 | 4.523089 |
| Mg | 18.969238 | 13.451657 | 5.185822 |
| Mg | 16.619935 | 11.996044 | 7.460553 |
| Mg | 16.627828 | 14.895656 | 7.449756 |
| Mg | 14.434187 | 16.443550 | 9.286110 |
| Si | 16.974970 | 16.442800 | 1.221250 |
| Si | 16.265610 | 13.453200 | 3.313620 |
| Si | 18.630320 | 16.439646 | 6.397404 |
| Si | 14.588712 | 13.443564 | 8.473922 |
| O | 17.681710 | 17.805520 | 0.556660 |
| O | 17.681710 | 15.080070 | 0.556660 |
| O | 15.349310 | 16.442800 | 1.386100 |
| O | 15.470890 | 13.453200 | 1.760420 |
| O | 17.713840 | 16.442800 | 2.799080 |
| O | 17.910830 | 13.453200 | 3.191290 |
| O | 15.504330 | 12.125360 | 4.002710 |
| O | 15.504330 | 14.781030 | 4.002710 |
| O | 17.881558 | 17.762800 | 5.701410 |
| O | 17.879776 | 15.116844 | 5.704645 |
| O | 15.527855 | 16.442446 | 6.519589 |
| O | 15.338353 | 13.448034 | 6.912066 |
| O | 17.838651 | 16.440918 | 7.943285 |
| O | 17.731391 | 13.449438 | 8.321046 |
| O | 15.266850 | 12.078521 | 9.147318 |
| O | 15.290528 | 14.800382 | 9.146615 |
| C | 11.201259 | 12.441170 | 12.087597 |
| C | 11.955076 | 11.171407 | 12.045684 |
| C | 11.274600 | 9.966713 | 11.776107 |
| C | 9.833493 | 9.966872 | 11.450612 |
| C | 9.088269 | 11.223793 | 11.443442 |
| C | 9.815332 | 12.454137 | 11.834473 |
| C | 8.926840 | 13.269722 | 12.643748 |

C 9.452152 14.063458 13.691644
C 10.884065 14.046521 13.964700
C 11.067382 14.107620 15.412868
C 12.104003 13.370418 16.027608
C 12.994449 12.542318 15.214199
C 12.817963 12.479026 13.814876
C 11.741522 13.239398 13.177620
C 12.949871 11.199721 13.117649
C 13.239125 10.022414 13.851920
C 12.540823 8.777984 13.545060
C 11.568683 8.752178 12.519496
C 10.318309 8.016499 12.704957
C 9.244031 8.769429 12.071056
C 7.965318 8.827672 12.654826
C 7.216486 10.098177 12.682900
C 7.774701 11.265801 12.119249
C 7.673450 12.537779 12.827180
C 6.991097 12.613552 14.062388
C 7.538440 13.424530 15.150708
C 8.744464 14.136460 14.969629
C 9.744873 14.165275 16.035145
C 9.504360 13.481560 17.247158
C 10.577521 12.719747 17.882917
C 11.854842 12.665379 17.282318
C 12.593276 11.402404 17.247244
C 13.298300 11.327028 15.970214
C 13.418124 10.088760 15.300565
C 12.831787 8.882813 15.886524
C 12.289233 8.069649 14.799942
C 11.080862 7.360934 14.981606
C 10.078464 7.328202 13.915759
C 8.756557 7.387625 14.530232
C 7.720422 8.128266 13.910922
C 6.831523 8.958345 14.722259
C 7.004926 9.018705 16.122167
C 6.882643 10.297898 16.819908
C 6.589764 11.472381 16.091491
C 6.405119 11.409950 14.643226
C 6.523625 10.173009 13.966234
C 7.289337 12.717877 16.405837
C 8.254756 12.745881 17.436319
C 8.556296 11.529752 18.189475
C 7.881069 10.327052 17.885770
C 8.619247 9.064046 17.849505
C 8.076699 8.255180 16.760954
C 8.937277 7.454138 15.978423
C 10.373156 7.436819 16.258301
C 10.897884 8.221830 17.308827
C 10.005694 9.048857 18.119123
C 10.704070 10.294651 18.432175
C 9.991733 11.513623 18.466098
C 12.027257 10.238319 17.813209
C 12.148022 8.957035 17.119897

**Fullerene Ni-[010]-fo**

Number of atoms: 396

Mg 2.543480 1.494800 0.388370

Mg 0.008060 5.935140 2.261970

Mg 0.008060 3.033660 2.261970

Mg 2.344995 4.483084 4.520549

Mg 4.719597 1.492002 5.181421

Mg 2.383890 0.044927 7.441283

Mg 2.384034 2.944074 7.441212

Mg 0.182784 4.483676 9.283004

Si 2.727970 4.484400 1.221250

Si 2.018610 1.494800 3.313620

Si 4.385751 4.481410 6.391930

Si 0.358184 1.493930 8.467372

O 3.434710 5.847120 0.556660

O 3.434700 3.121670 0.556660

O 1.102310 4.484400 1.386100

O 1.223890 1.494800 1.760420

O 3.466830 4.484400 2.799080

O 3.663830 1.494800 3.191290

O 1.257330 0.166960 4.002710

O 1.257330 2.822640 4.002710

O 3.631933 5.800097 5.694747

O 3.633111 3.159952 5.699945

O 1.275113 4.482754 6.515459

O 1.095206 1.494469 6.901033

O 3.597843 4.486166 7.938438

O 3.484564 1.495735 8.308898

O 1.059140 0.139005 9.140321

O 1.057427 2.851261 9.137681

Mg 2.543480 7.474000 0.388370

Mg 0.008060 11.914340 2.261970

Mg 0.008060 9.012860 2.261970

Mg 2.353265 10.464719 4.533645

Mg 4.710344 7.473901 5.168900

Mg 2.378986 6.021104 7.434413

Mg 2.388541 8.925072 7.452452

Mg 0.184907 10.459328 9.289813

Si 2.727970 10.463600 1.221250

Si 2.018610 7.474000 3.313620

Si 4.395491 10.463875 6.425438

Si 0.358771 7.473320 8.469159

O 3.434710 11.826320 0.556660

O 3.434710 9.100870 0.556660

O 1.102310 10.463600 1.386100

O 1.223890 7.474000 1.760420

O 3.466830 10.463600 2.799080

O 3.663830 7.474000 3.191290

O 1.257330 6.146160 4.002710

O 1.257330 8.801840 4.002710

O 3.638672 11.781893 5.723838

O 3.640651 9.145226 5.721128

O 1.281233 10.463709 6.528524

O 1.095459 7.476272 6.902980

| | | | |
|---|---|---|---|
| O | 3.615046 | 10.461105 | 7.975162 |
| O | 3.482744 | 7.462957 | 8.302840 |
| O | 1.058591 | 6.115437 | 9.138030 |
| O | 1.061618 | 8.826019 | 9.146663 |
| Mg | 2.543480 | 13.453200 | 0.388370 |
| Mg | 0.008060 | 17.893540 | 2.261970 |
| Mg | 0.008060 | 14.992060 | 2.261970 |
| Mg | 2.344824 | 16.441588 | 4.522990 |
| Mg | 4.718242 | 13.448244 | 5.184145 |
| Mg | 2.391598 | 12.000595 | 7.461827 |
| Mg | 2.383578 | 14.898236 | 7.447686 |
| Mg | 0.185887 | 16.442689 | 9.284947 |
| Si | 2.727970 | 16.442800 | 1.221250 |
| Si | 2.018610 | 13.453200 | 3.313620 |
| Si | 4.383889 | 16.439000 | 6.396683 |
| Si | 0.358155 | 13.448989 | 8.478086 |
| O | 3.434710 | 17.805520 | 0.556660 |
| O | 3.434710 | 15.080070 | 0.556660 |
| O | 1.102310 | 16.442800 | 1.386100 |
| O | 1.223890 | 13.453200 | 1.760420 |
| O | 3.466830 | 16.442800 | 2.799080 |
| O | 3.663830 | 13.453200 | 3.191290 |
| O | 1.257330 | 12.125360 | 4.002710 |
| O | 1.257330 | 14.781030 | 4.002710 |
| O | 3.632750 | 17.760774 | 5.701483 |
| O | 3.632074 | 15.117238 | 5.703345 |
| O | 1.273631 | 16.438957 | 6.520830 |
| O | 1.096677 | 13.446702 | 6.912409 |
| O | 3.593321 | 16.441465 | 7.941890 |
| O | 3.486073 | 13.455997 | 8.323788 |
| O | 1.058632 | 12.093834 | 9.154059 |
| O | 1.056937 | 14.807692 | 9.147388 |
| Mg | 7.292480 | 1.494800 | 0.388370 |
| Mg | 4.757060 | 5.935140 | 2.261970 |
| Mg | 4.757060 | 3.033660 | 2.261970 |
| Mg | 7.095792 | 4.482680 | 4.521969 |
| Mg | 9.470169 | 1.491488 | 5.183897 |
| Mg | 7.132617 | 0.044395 | 7.446341 |
| Mg | 7.135794 | 2.943059 | 7.445859 |
| Mg | 4.940344 | 4.493932 | 9.284706 |
| Si | 7.476970 | 4.484400 | 1.221250 |
| Si | 6.767610 | 1.494800 | 3.313620 |
| Si | 9.137454 | 4.480837 | 6.395725 |
| Si | 5.105465 | 1.497735 | 8.471287 |
| O | 8.183700 | 5.847120 | 0.556660 |
| O | 8.183700 | 3.121670 | 0.556660 |
| O | 5.851310 | 4.484400 | 1.386100 |
| O | 5.972890 | 1.494800 | 1.760420 |
| O | 8.215830 | 4.484400 | 2.799080 |
| O | 8.412830 | 1.494800 | 3.191290 |
| O | 6.006330 | 0.166960 | 4.002710 |
| O | 6.006330 | 2.822640 | 4.002710 |
| O | 8.383655 | 5.797195 | 5.693021 |
| O | 8.384535 | 3.158689 | 5.705003 |

O 6.027909 4.480158 6.517445

O 5.842905 1.495451 6.904767

O 8.348268 4.489213 7.942459

O 8.234586 1.493105 8.311807

O 5.805533 0.141824 9.143995

O 5.806657 2.855317 9.141546

Mg 7.292480 7.474000 0.388370

Mg 4.757060 11.914340 2.261970

Mg 4.757060 9.012860 2.261970

Mg 7.095504 10.466442 4.521541

Mg 9.464050 7.469845 5.163179

Mg 7.135161 6.020676 7.427640

Mg 7.131233 8.926415 7.400460

Mg 4.922921 10.463693 9.388316

Si 7.476970 10.463600 1.221250

Si 6.767610 7.474000 3.313620

Si 9.168917 10.466703 6.368462

Si 5.103332 7.466472 8.436637

O 8.183700 11.826320 0.556660

O 8.183700 9.100870 0.556660

O 5.851310 10.463600 1.386100

O 5.972890 7.474000 1.760420

O 8.215830 10.463600 2.799080

O 8.412830 7.474000 3.191290

O 6.006330 6.146160 4.002710

O 6.006330 8.801840 4.002710

O 8.389017 11.785243 5.692371

O 8.392949 9.145958 5.687407

O 6.042393 10.466067 6.502363

O 5.840965 7.455638 6.876096

O 8.388551 10.466985 7.910648

O 8.229484 7.482849 8.275004

O 5.815025 6.130484 9.144489

O 5.806017 8.838209 9.074623

Mg 7.292480 13.453200 0.388370

Mg 4.757060 17.893540 2.261970

Mg 4.757060 14.992060 2.261970

Mg 7.093990 16.441856 4.522636

Mg 9.466887 13.451748 5.173808

Mg 7.130660 11.998435 7.432731

Mg 7.126313 14.899999 7.442060

Mg 4.934572 16.443298 9.286433

Si 7.476970 16.442800 1.221250

Si 6.767610 13.453200 3.313620

Si 9.130550 16.436775 6.397648

Si 5.108518 13.450203 8.476933

O 8.183700 17.805520 0.556660

O 8.183700 15.080070 0.556660

O 5.851310 16.442800 1.386100

O 5.972890 13.453200 1.760420

O 8.215830 16.442800 2.799080

O 8.412830 13.453200 3.191290

O 6.006330 12.125360 4.002710

O 6.006330 14.781030 4.002710

O 8.383334 17.760073 5.702897
O 8.379980 15.115670 5.702266
O 6.026551 16.441911 6.518859
O 5.837844 13.451789 6.904909
O 8.343057 16.436670 7.943703
O 8.218773 13.445287 8.308999
O 5.808777 12.091721 9.140885
O 5.809313 14.810169 9.143422
Mg 12.041480 1.494800 0.388370
Mg 9.506060 5.935140 2.261970
Mg 9.506060 3.033660 2.261970
Mg 11.843630 4.484292 4.520803
Mg 14.215021 1.490070 5.180378
Mg 11.878735 0.042662 7.447289
Mg 11.878426 2.944589 7.445189
Mg 9.678847 4.507704 9.302544
Si 12.225970 4.484400 1.221250
Si 11.516610 1.494800 3.313620
Si 13.882739 4.480478 6.393088
Si 9.854260 1.496563 8.478887
O 12.932710 5.847120 0.556660
O 12.932710 3.121670 0.556660
O 10.600310 4.484400 1.386100
O 10.721890 1.494800 1.760420
O 12.964830 4.484400 2.799080
O 13.161830 1.494800 3.191290
O 10.755330 0.166960 4.002710
O 10.755330 2.822640 4.002710
O 13.133865 5.801260 5.695965
O 13.130203 3.158676 5.701539
O 10.778178 4.483659 6.517386
O 10.591736 1.493862 6.910757
O 13.090759 4.485183 7.939231
O 12.979594 1.494288 8.315160
O 10.552389 0.135884 9.148522
O 10.544420 2.863001 9.145321
Mg 12.041480 7.474000 0.388370
Mg 9.506060 11.914340 2.261970
Mg 9.506060 9.012860 2.261970
Mg 11.846352 10.464595 4.518206
Mg 14.218994 7.473628 5.177097
Mg 11.885224 6.027348 7.433997
Mg 11.875145 8.938252 7.409379
Ni 9.514473 10.454309 9.475073
Si 12.225970 10.463600 1.221250
Si 11.516610 7.474000 3.313620
Si 13.891526 10.464885 6.387034
Si 9.849854 7.491362 8.425547
O 12.932710 11.826320 0.556660
O 12.932710 9.100870 0.556660
O 10.600310 10.463600 1.386100
O 10.721890 7.474000 1.760420
O 12.964840 10.463600 2.799080
O 13.161830 7.474000 3.191290

| | | | |
|---|---|---|---|
| O | 10.755330 | 6.146160 | 4.002710 |
| O | 10.755330 | 8.801840 | 4.002710 |
| O | 13.141095 | 11.786890 | 5.694928 |
| O | 13.144762 | 9.142252 | 5.688171 |
| O | 10.808174 | 10.464046 | 6.437243 |
| O | 10.597275 | 7.462483 | 6.872107 |
| O | 13.095759 | 10.459118 | 7.926033 |
| O | 12.972811 | 7.482703 | 8.300465 |
| O | 10.546909 | 6.152008 | 9.140732 |
| O | 10.555964 | 8.880214 | 9.053839 |
| Mg | 12.041480 | 13.453200 | 0.388370 |
| Mg | 9.506060 | 17.893540 | 2.261970 |
| Mg | 9.506060 | 14.992060 | 2.261970 |
| Mg | 11.843044 | 16.440815 | 4.522832 |
| Mg | 14.218617 | 13.449739 | 5.181069 |
| Mg | 11.865387 | 11.976510 | 7.432644 |
| Mg | 11.876840 | 14.884814 | 7.444016 |
| Mg | 9.694002 | 16.426601 | 9.280097 |
| Si | 12.225970 | 16.442800 | 1.221250 |
| Si | 11.516610 | 13.453200 | 3.313620 |
| Si | 13.877719 | 16.434955 | 6.400520 |
| Si | 9.836222 | 13.430537 | 8.462933 |
| O | 12.932710 | 17.805520 | 0.556660 |
| O | 12.932710 | 15.080070 | 0.556660 |
| O | 10.600310 | 16.442800 | 1.386100 |
| O | 10.721890 | 13.453200 | 1.760420 |
| O | 12.964840 | 16.442800 | 2.799080 |

| | | | |
|---|---|---|---|
| O | 13.161830 | 13.453200 | 3.191290 |
| O | 10.755330 | 12.125360 | 4.002710 |
| O | 10.755330 | 14.781030 | 4.002710 |
| O | 13.128157 | 17.758318 | 5.706094 |
| O | 13.127540 | 15.113767 | 5.704868 |
| O | 10.771191 | 16.434837 | 6.527373 |
| O | 10.583610 | 13.441569 | 6.901967 |
| O | 13.084094 | 16.432832 | 7.945610 |
| O | 12.968176 | 13.430696 | 8.312993 |
| O | 10.529897 | 12.048326 | 9.114973 |
| O | 10.547029 | 14.775448 | 9.149863 |
| Mg | 16.790480 | 1.494800 | 0.388370 |
| Mg | 14.255060 | 5.935140 | 2.261970 |
| Mg | 14.255060 | 3.033660 | 2.261970 |
| Mg | 16.592250 | 4.484532 | 4.520912 |
| Mg | 18.965895 | 1.491640 | 5.179881 |
| Mg | 16.627435 | 0.042840 | 7.442925 |
| Mg | 16.628880 | 2.941155 | 7.439312 |
| Mg | 14.433080 | 4.483091 | 9.282877 |
| Si | 16.974970 | 4.484400 | 1.221250 |
| Si | 16.265610 | 1.494800 | 3.313620 |
| Si | 18.629493 | 4.482988 | 6.392695 |
| Si | 14.601418 | 1.493301 | 8.472627 |
| O | 17.681710 | 5.847120 | 0.556660 |
| O | 17.681710 | 3.121670 | 0.556660 |
| O | 15.349310 | 4.484400 | 1.386100 |
| O | 15.470890 | 1.494800 | 1.760420 |

O 17.713830 4.484400 2.799080
O 17.910830 1.494800 3.191290
O 15.504330 0.166960 4.002710
O 15.504330 2.822640 4.002710
O 17.879305 5.804392 5.697348
O 17.878915 3.161486 5.697609
O 15.524286 4.482104 6.518967
O 15.336012 1.492375 6.904719
O 17.838391 4.483860 7.938137
O 17.733733 1.492831 8.304440
O 15.301752 0.136011 9.142642
O 15.305711 2.848859 9.142005
Mg 16.790480 7.474000 0.388370
Mg 14.255060 11.914340 2.261970
Mg 14.255060 9.012860 2.261970
Mg 16.593981 10.464142 4.525560
Mg 18.966704 7.475309 5.180564
Mg 16.627700 6.026457 7.441242
Mg 16.627473 8.925036 7.443480
Mg 14.403795 10.456874 9.298651
Si 16.974970 10.463600 1.221250
Si 16.265610 7.474000 3.313620
Si 18.635170 10.464402 6.398808
Si 14.594949 7.477362 8.466712
O 17.681710 11.826320 0.556660
O 17.681710 9.100870 0.556660
O 15.349310 10.463600 1.386100
O 15.470890 7.474000 1.760420
O 17.713840 10.463600 2.799080
O 17.910830 7.474000 3.191290
O 15.504330 6.146160 4.002710
O 15.504330 8.801840 4.002710
O 17.884770 11.786770 5.704937
O 17.884813 9.143362 5.701774
O 15.534197 10.464014 6.509266
O 15.337623 7.474028 6.902790
O 17.842799 10.462322 7.943584
O 17.733992 7.474160 8.307357
O 15.296519 6.121056 9.139357
O 15.295958 8.831310 9.141308
Mg 16.790480 13.453200 0.388370
Mg 14.255060 17.893540 2.261970
Mg 14.255060 14.992060 2.261970
Mg 16.592351 16.440670 4.522899
Mg 18.969979 13.450383 5.185322
Mg 16.626928 11.999496 7.449117
Mg 16.625683 14.894592 7.449294
Mg 14.429886 16.438140 9.286570
Si 16.974970 16.442800 1.221250
Si 16.265610 13.453200 3.313620
Si 18.628354 16.436620 6.398032
Si 14.591308 13.440360 8.473996
O 17.681710 17.805520 0.556660
O 17.681710 15.080070 0.556660

O 15.349310 16.442800 1.386100

O 15.470890 13.453200 1.760420

O 17.713840 16.442800 2.799080

O 17.910830 13.453200 3.191290

O 15.504330 12.125360 4.002710

O 15.504330 14.781030 4.002710

O 17.878548 17.758918 5.702230

O 17.877991 15.113856 5.705415

O 15.520213 16.437054 6.523879

O 15.334280 13.447775 6.909948

O 17.837253 16.438205 7.943713

O 17.733149 13.448290 8.315267

O 15.294151 12.084061 9.143899

O 15.290913 14.798003 9.146863

C 9.847789 10.365632 11.599538

C 10.754168 9.335877 12.129136

C 10.364879 7.980320 12.162578

C 9.027954 7.585137 11.709244

C 8.119714 8.555850 11.244253

C 8.518621 9.971998 11.175037

C 7.352920 10.784502 11.555614

C 7.522368 11.945227 12.332964

C 8.862563 12.338688 12.787777

C 8.731411 12.873787 14.140535

C 9.731514 12.617880 15.105943

C 10.895041 11.808632 14.751931

C 11.014965 11.284751 13.444791

C 9.988950 11.561360 12.446101

C 11.487433 9.913610 13.247406

C 11.825248 9.113259 14.363351

C 11.426259 7.708812 14.397730

C 10.705119 7.154340 13.315287

C 9.585598 6.245239 13.570057

C 8.550038 6.503987 12.568992

C 7.182102 6.450988 12.934898

C 6.245662 7.458479 12.439513

C 6.716076 8.499961 11.613603

C 6.233978 9.875162 11.802114

C 5.311489 10.164581 12.837842

C 5.484690 11.368299 13.649517

C 6.573267 12.237814 13.402470

C 7.319499 12.813107 14.519758

C 6.956620 12.497087 15.846809

C 7.991020 12.231500 16.845201

C 9.354406 12.288573 16.479321

C 10.286749 11.276731 16.974273

C 11.238918 10.978684 15.905068

C 11.695695 9.655454 15.714338

C 11.216094 8.583542 16.585782

C 11.049477 7.380691 15.771205

C 9.967285 6.506772 16.016523

C 9.223824 5.928555 14.897683

C 7.812122 5.872046 15.274278

C 6.810141 6.128821 14.311666

C 5.646329 6.939200 14.670101

C 5.522858 7.462509 15.976384

C 5.047984 8.830089 16.175735

C 4.713198 9.629151 15.059435

C 4.833759 9.086438 13.708593

C 5.301473 7.766959 13.516158

C 5.113750 11.036288 15.023263

C 5.835977 11.592220 16.102829

C 6.179543 10.765918 17.257648

C 5.793543 9.407781 17.292734

C 6.726021 8.395680 17.786703

C 6.558941 7.193259 16.972638

C 7.683502 6.412629 16.626307

C 9.015595 6.805544 17.084555

C 9.177146 7.966604 17.872469

C 8.012640 8.776147 18.227828

C 8.412228 10.181986 18.192896

C 7.511998 11.159875 17.715419

C 9.823453 10.241206 17.815364

C 10.296544 8.871429 17.618068

**Fullerene $V_{MgO}$-[010]-fo**

Number of atoms: 394

Mg 2.543480 1.494800 0.388370

Mg 0.008060 5.935140 2.261970

Mg 0.008060 3.033660 2.261970

Mg 2.343011 4.484010 4.515914

Mg 4.714698 1.479007 5.177273

Mg 2.380126 0.031500 7.437662

Mg 2.376072 2.929958 7.430386

Mg 0.172719 4.475940 9.281006

Si 2.727970 4.484400 1.221250

Si 2.018610 1.494800 3.313620

Si 4.371520 4.466374 6.385819

Si 0.353321 1.488659 8.462673

O 3.434710 5.847120 0.556660

O 3.434700 3.121670 0.556660

O 1.102310 4.484400 1.386100

O 1.223890 1.494800 1.760420

O 3.466830 4.484400 2.799080

O 3.663830 1.494800 3.191290

O 1.257330 0.166960 4.002710

O 1.257330 2.822640 4.002710

O 3.631203 5.791386 5.691342

O 3.623561 3.143017 5.694414

O 1.279151 4.478477 6.503988

O 1.089774 1.482515 6.895472

O 3.583290 4.467753 7.929534

O 3.478844 1.481233 8.304727

O 1.050404 0.132853 9.136361

O 1.055165 2.847425 9.128416

Mg 2.543480 7.474000 0.388370

Mg 0.008060 11.914340 2.261970

Mg 0.008060 9.012860 2.261970

Mg 2.356520 10.463605 4.535485

Mg 4.712924 7.479302 5.181708

Mg 2.373783 6.018652 7.439092

Mg 2.382415 8.917388 7.454821

Mg 0.170842 10.459267 9.284735

Si 2.727970 10.463600 1.221250

Si 2.018610 7.474000 3.313620

Si 4.395751 10.450518 6.427160

Si 0.341777 7.473016 8.463605

O 3.434710 11.826320 0.556660

O 3.434710 9.100870 0.556660

O 1.102310 10.463600 1.386100

O 1.223890 7.474000 1.760420

O 3.466830 10.463600 2.799080

O 3.663830 7.474000 3.191290

O 1.257330 6.146160 4.002710

O 1.257330 8.801840 4.002710

O 3.643180 11.771444 5.723919

O 3.630614 9.138910 5.720101

O 1.281722 10.459381 6.518038

O 1.085173 7.469359 6.900403

O 3.609356 10.450630 7.975610

O 3.468785 7.456011 8.313907

O 1.036707 6.112173 9.133625

O 1.044204 8.824228 9.141015

Mg 2.543480 13.453200 0.388370

Mg 0.008060 17.893540 2.261970

Mg 0.008060 14.992060 2.261970

Mg 2.348947 16.436870 4.521769

Mg 4.741451 13.424351 5.204701

Mg 2.391439 11.991295 7.459026

Mg 2.389525 14.883393 7.441804

Mg 0.181736 16.433248 9.280729

Si 2.727970 16.442800 1.221250

Si 2.018610 13.453200 3.313620

Si 4.388869 16.423220 6.399323

Si 0.354408 13.442464 8.465845

O 3.434710 17.805520 0.556660

O 3.434710 15.080070 0.556660

O 1.102310 16.442800 1.386100

O 1.223890 13.453200 1.760420

O 3.466830 16.442800 2.799080

O 3.663830 13.453200 3.191290

O 1.257330 12.125360 4.002710

O 1.257330 14.781030 4.002710

O 3.638753 17.744155 5.699519

O 3.644315 15.097910 5.708679

O 1.281058 16.434333 6.506516

O 1.096258 13.439907 6.902185

O 3.592613 16.431403 7.941771

O 3.503165 13.441462 8.308540

O 1.054196 12.089284 9.144307

O 1.057317 14.801916 9.133511

Mg 7.292480 1.494800 0.388370

Mg 4.757060 5.935140 2.261970
Mg 4.757060 3.033660 2.261970
Mg 7.088477 4.479312 4.513474
Mg 9.466679 1.477813 5.177886
Mg 7.127644 0.009585 7.454586
Mg 7.120537 2.907120 7.426667
Mg 4.901500 4.447071 9.295165
Si 7.476970 4.484400 1.221250
Si 6.767610 1.494800 3.313620
Si 9.122880 4.465516 6.402129
Si 5.098959 1.466849 8.467850
O 8.183700 5.847120 0.556660
O 8.183700 3.121670 0.556660
O 5.851310 4.484400 1.386100
O 5.972890 1.494800 1.760420
O 8.215830 4.484400 2.799080
O 8.412830 1.494800 3.191290
O 6.006330 0.166960 4.002710
O 6.006330 2.822640 4.002710
O 8.385439 5.795602 5.709176
O 8.382246 3.145826 5.696583
O 6.016175 4.446942 6.491944
O 5.838179 1.458107 6.900823
O 8.317461 4.449140 7.938273
O 8.233329 1.463029 8.309096
O 5.798054 0.111877 9.144173
O 5.801831 2.827084 9.131519

Mg 7.292480 7.474000 0.388370
Mg 4.757060 11.914340 2.261970
Mg 4.757060 9.012860 2.261970
Mg 7.123591 10.462334 4.558585
Mg 9.424055 7.487523 5.166639
Mg 7.063802 5.967052 7.411052
Mg 7.146643 8.907539 7.456312
Mg 4.944334 10.437053 9.355433
Si 7.476970 10.463600 1.221250
Si 6.767610 7.474000 3.313620
Si 9.157028 10.420621 6.465826
Si 5.090436 7.450132 8.474595
O 8.183700 11.826320 0.556660
O 8.183700 9.100870 0.556660
O 5.851310 10.463600 1.386100
O 5.972890 7.474000 1.760420
O 8.215830 10.463600 2.799080
O 8.412830 7.474000 3.191290
O 6.006330 6.146160 4.002710
O 6.006330 8.801840 4.002710
O 8.413285 11.757537 5.734804
O 8.392937 9.125424 5.683280
O 6.035259 10.441344 6.538129
O 5.820861 7.460267 6.904530
O 8.421443 10.374424 7.955838
O 8.127877 7.358427 8.324343
O 5.773169 6.075992 9.132546

O 5.794877 8.790874 9.165268

Mg 7.292480 13.453200 0.388370

Mg 4.757060 17.893540 2.261970

Mg 4.757060 14.992060 2.261970

Mg 7.091606 16.435079 4.523726

Mg 9.448926 13.410722 5.174264

Mg 7.188521 11.922857 7.523479

Mg 7.133981 14.864064 7.452459

Mg 4.927603 16.411352 9.292401

Si 7.476970 16.442800 1.221250

Si 6.767610 13.453200 3.313620

Si 9.133653 16.414295 6.401258

Si 5.124842 13.416537 8.483459

O 8.183700 17.805520 0.556660

O 8.183700 15.080070 0.556660

O 5.851310 16.442800 1.386100

O 5.972890 13.453200 1.760420

O 8.215830 16.442800 2.799080

O 8.412830 13.453200 3.191290

O 6.006330 12.125360 4.002710

O 6.006330 14.781030 4.002710

O 8.387334 17.738923 5.708735

O 8.386561 15.089022 5.714575

O 6.032690 16.417128 6.520884

O 5.882432 13.390737 6.927982

O 8.353117 16.404614 7.952154

O 8.238907 13.427415 8.390021

O 5.814045 12.074566 9.189662

O 5.817955 14.788969 9.138521

Mg 12.041480 1.494800 0.388370

Mg 9.506060 5.935140 2.261970

Mg 9.506060 3.033660 2.261970

Mg 11.838611 4.483061 4.521897

Mg 14.212880 1.508172 5.179505

Mg 11.885613 0.036005 7.435678

Mg 11.868217 2.946464 7.451168

Mg 9.602220 4.394035 9.334552

Si 12.225970 4.484400 1.221250

Si 11.516610 1.494800 3.313620

Si 13.864828 4.506253 6.407416

Si 9.853693 1.455753 8.471115

O 12.932710 5.847120 0.556660

O 12.932710 3.121670 0.556660

O 10.600310 4.484400 1.386100

O 10.721890 1.494800 1.760420

O 12.964830 4.484400 2.799080

O 13.161830 1.494800 3.191290

O 10.755330 0.166960 4.002710

O 10.755330 2.822640 4.002710

O 13.102078 5.834640 5.726502

O 13.121685 3.185132 5.702343

O 10.764696 4.499124 6.515397

O 10.590737 1.485133 6.905211

O 13.076259 4.493004 7.953600

O 12.974692 1.493699 8.308835

O 10.558971 0.095644 9.130833

O 10.544530 2.807284 9.161023

Mg 12.041480 7.474000 0.388370

Mg 9.506060 11.914340 2.261970

Mg 9.506060 9.012860 2.261970

Mg 11.827571 10.469548 4.509029

Mg 14.232080 7.465273 5.163650

Mg 11.842534 6.029068 7.479666

Mg 11.954679 8.925634 7.277172

Si 12.225839 10.459302 1.228990

Si 11.516610 7.474000 3.313620

Si 13.888210 10.463600 6.382630

Si 9.713104 7.367383 8.594884

O 12.927873 11.816399 0.557582

O 12.932710 9.100870 0.556660

O 10.600310 10.463600 1.386100

O 10.721890 7.474000 1.760420

O 12.964840 10.463600 2.799080

O 13.161830 7.474000 3.191290

O 10.755330 6.146160 4.002710

O 10.755330 8.801840 4.002710

O 13.128110 11.791550 5.692940

O 13.157002 9.143106 5.648167

O 10.820597 10.434169 6.445492

O 10.522041 7.615839 7.109585

O 13.103871 10.439524 7.911344

O 12.948140 7.495508 8.315460

O 10.533999 5.996134 9.097186

Mg 12.056407 13.451009 0.411030

Mg 9.505102 17.892906 2.255300

Mg 9.506060 14.992060 2.261970

Mg 11.837740 16.442800 4.520030

Mg 14.211300 13.453200 5.174280

Mg 11.905854 11.972154 7.354351

Mg 11.899210 14.886450 7.435312

Mg 9.690608 16.398228 9.303442

Si 12.223930 16.442402 1.231022

Si 11.511589 13.454384 3.309857

Si 13.888220 16.442800 6.382630

Si 9.847270 13.453200 8.472360

O 12.921250 17.801089 0.557995

O 12.927195 15.086266 0.560370

O 10.600310 16.442800 1.386100

O 10.721890 13.453200 1.760420

O 12.964840 16.442800 2.799080

O 13.161830 13.453200 3.191290

O 10.755330 12.125360 4.002710

O 10.755330 14.781030 4.002710

O 13.128120 17.770750 5.692940

O 13.128120 15.114850 5.692940

O 10.775829 16.416142 6.530859

O 10.581898 13.430243 6.932340

O 13.097517 16.435187 7.931651

O 12.968393 13.435819 8.300707

O 10.611143 12.008103 9.004984

O 10.570739 14.749229 9.211977

Mg 16.804303 1.494265 0.410585

Mg 14.254833 5.934271 2.254089

Mg 14.255060 3.033660 2.261970

Mg 16.586740 4.484400 4.520030

Mg 18.960290 1.494800 5.174280

Mg 16.630275 0.048065 7.435356

Mg 16.623520 2.946995 7.433998

Mg 14.418483 4.481483 9.298988

Si 16.973504 4.484535 1.232095

Si 16.261139 1.493127 3.310536

Si 18.637210 4.484400 6.382630

Si 14.596270 1.494800 8.472360

O 17.674213 5.841073 0.559583

O 17.674899 3.128185 0.559677

O 15.349310 4.484400 1.386100

O 15.470890 1.494800 1.760420

O 17.713830 4.484400 2.799080

O 17.910830 1.494800 3.191290

O 15.504330 0.166960 4.002710

O 15.504330 2.822640 4.002710

O 17.877110 5.812340 5.692940

O 17.877110 3.156450 5.692940

O 15.508690 4.496367 6.516584

O 15.330805 1.497401 6.903246

O 17.837632 4.485274 7.925659

O 17.728431 1.498632 8.301086

O 15.301203 0.140424 9.140365

O 15.297107 2.852940 9.140404

Mg 16.803729 7.475254 0.409478

Mg 14.256697 11.911609 2.259226

Mg 14.255060 9.012860 2.261970

Mg 16.586740 10.463600 4.520030

Mg 18.960300 7.474000 5.174280

Mg 16.629270 6.030284 7.432478

Mg 16.601835 8.943518 7.448040

Mg 14.297092 10.472678 9.445142

Si 16.973854 10.463866 1.232468

Si 16.262566 7.476642 3.308568

Si 18.637210 10.463600 6.382630

Si 14.596270 7.474000 8.472360

O 17.675193 11.819090 0.558680

O 17.674926 9.108130 0.558889

O 15.349310 10.463600 1.386100

O 15.470890 7.474000 1.760420

O 17.713840 10.463600 2.799080

O 17.910830 7.474000 3.191290

O 15.504330 6.146160 4.002710

O 15.504330 8.801840 4.002710

O 17.877120 11.791540 5.692940

O 17.877110 9.135650 5.692940

O 15.530394 10.464779 6.462341

O 15.319909 7.467080 6.904773

O 17.839662 10.463926 7.928010

O 17.714744 7.481548 8.302608

O 15.283019 6.119571 9.150272

O 15.246139 8.861854 9.118700

Mg 16.805649 13.453228 0.412393

Mg 14.254316 17.892295 2.256387

Mg 14.255060 14.992060 2.261970

Mg 16.586740 16.442800 4.520030

Mg 18.960300 13.453200 5.174280

Mg 16.605593 11.988393 7.448020

Mg 16.635021 14.893493 7.435008

Mg 14.433165 16.442526 9.284689

Si 16.973656 16.442687 1.232085

Si 16.261370 13.452003 3.311520

Si 18.637220 16.442800 6.382630

Si 14.596270 13.453200 8.472360

O 17.675284 17.798424 0.559291

O 17.675474 15.087718 0.559017

O 15.349310 16.442800 1.386100

O 15.470890 13.453200 1.760420

O 17.713840 16.442800 2.799080

O 17.910830 13.453200 3.191290

O 15.504330 12.125360 4.002710

O 15.504330 14.781030 4.002710

O 17.877120 17.770750 5.692940

O 17.877120 15.114850 5.692940

O 15.527613 16.443348 6.508620

O 15.330401 13.461931 6.902051

O 17.842477 16.441859 7.929089

O 17.728364 13.443854 8.306376

O 15.259735 12.067409 9.115432

O 15.297686 14.805501 9.148078

C 10.530411 11.445158 10.323820

C 11.610062 10.363973 10.531187

C 11.336592 8.995228 10.507085

C 9.946114 8.434936 10.253731

C 8.909466 9.479140 10.550025

C 9.175719 10.842107 10.600223

C 8.168904 11.500816 11.427256

C 8.520872 12.584158 12.243945

C 9.931417 13.037650 12.261606

C 10.261507 13.396989 13.631469

C 11.559749 13.145641 14.140302

C 12.553056 12.512445 13.288404

C 12.209154 12.163448 11.959620

C 10.898652 12.453829 11.432132

C 12.658728 10.875363 11.405861

C 13.440951 9.991652 12.202675

C 13.150575 8.564059 12.171350

C 12.095226 8.093992 11.354539

C 11.196898 7.059369 11.845747

C 9.878570 7.323226 11.313816

C 8.748322 7.077088 12.106250

C 7.622415 8.037867 12.125952
C 7.707262 9.205858 11.356315
C 7.236478 10.478656 11.900959
C 6.738672 10.546702 13.223299
C 7.129207 11.654953 14.093358
C 8.012482 12.651824 13.607317
C 9.084837 13.156675 14.464221
C 9.242043 12.655322 15.775066
C 10.581965 12.378778 16.294764
C 11.719059 12.621313 15.493486
C 12.822582 11.658669 15.477285
C 13.336753 11.588651 14.113305
C 13.779942 10.357343 13.583182
C 13.697907 9.145931 14.395972
C 13.312003 8.037925 13.523323
C 12.436320 7.035011 14.001785
C 11.368934 6.522011 13.145182
C 10.200596 6.266351 13.971555
C 8.912583 6.559263 13.457808
C 7.907039 7.188474 14.312192
C 8.229918 7.526914 15.645043
C 7.782183 8.802816 16.201424
C 7.023370 9.690276 15.407689
C 6.671751 9.333880 14.035527
C 7.114295 8.102470 13.491745
C 7.307306 11.127587 15.442862
C 8.337603 11.622135 16.273768
C 9.123113 10.700324 17.095916
C 8.849681 9.315539 17.060453
C 9.950388 8.355401 17.037516
C 9.567027 7.246636 16.167287
C 10.534244 6.627519 15.345680
C 11.921683 7.098818 15.366194
C 12.291382 8.171160 16.207858
C 11.290361 8.808602 17.061869
C 11.572074 10.239463 17.097693
C 10.505941 11.169339 17.111157
C 12.748050 10.488169 16.265178
C 13.194039 9.209891 15.716710
